# The elusive abnormal CO_2_ insertion enabled by metal-ligand cooperative photochemical selectivity inversion

**DOI:** 10.1038/s41467-018-03239-3

**Published:** 2018-03-21

**Authors:** Felix Schneck, Jennifer Ahrens, Markus Finger, A. Claudia Stückl, Christian Würtele, Dirk Schwarzer, Sven Schneider

**Affiliations:** 1 Universität Göttingen, Institut für Anorganische Chemie, Tammannstr. 4, 37077 Göttingen, Germany; 2 Department of Dynamics at Surfaces, Max Planck Institute for Biophysical Chemistry, Am Fassberg 11, 37077 Göttingen, Germany

## Abstract

Direct hydrogenation of CO_2_ to CO, the reverse water–gas shift reaction, is an attractive route to CO_2_ utilization. However, the use of molecular catalysts is impeded by the general reactivity of metal hydrides with CO_2_. Insertion into M–H bonds results in formates (MO(O)CH), whereas the abnormal insertion to the hydroxycarbonyl isomer (MC(O)OH), which is the key intermediate for CO-selective catalysis, has never been directly observed. We here report that the selectivity of CO_2_ insertion into a Ni–H bond can be inverted from normal to abnormal insertion upon switching from thermal to photochemical conditions. Mechanistic examination for abnormal insertion indicates photochemical N–H reductive elimination as the pivotal step that leads to an umpolung of the hydride ligand. This study conceptually introduces metal-ligand cooperation for selectivity control in photochemical transformations.

## Abstract (web summary)

The development of molecular catalysts for the reverse water–gas shift reaction is impeded by the general selectivity of CO_2_ insertion into M–H bonds to formates. Here, the authors report that the selectivity of CO_2_ insertion into a Ni–H bond can be inverted from normal to abnormal insertion upon switching from thermal to photochemical conditions.

## Introduction

Selectivity control is a key issue for CO_2_ functionalization to added value products. The products from CO_2_ reduction can range from oxalic acid (1-electron) to carbon monoxide or formic acid (2-electron), formaldehyde (4-electron), and methanol (6-electron) all the way to hydrocarbons^1^. The primary industrial C_1_ building block CO is a particularly attractive target. In nature, CO_2_ reduction to CO is catalyzed by the enzyme [NiFe] carbon monoxide dehydrogenase ([NiFe]-CODH), which contains a nickel capped [Fe_4_S_4_Ni]-cluster as active site^1^. The proposed mechanism comprises CO_2_ binding to nickel followed by formation of a hydroxycarbonyl bridged {Ni(CO_2_H)Fe} intermediate as selectivity deriving step upon electron and proton transfer. Alternatively, CO_2_ insertion into a Ni–H bond has also been discussed^2^. In analogy, metallocarboxylate (MCO_2_H/M) species are also proposed as the key intermediates for synthetic CO-selective molecular catalysis, yet rarely detected^3–8^. Such systems are generally electro- or photochemically driven^1,9–12^ as direct hydrogenation by reverse water–gas shift (RWGS) is endothermic (see equation 1).

1 $${\mathrm{CO}}_{\mathrm{2}} + {\mathrm{H}}_{\mathrm{2}} \to {\mathrm{CO}} + {\mathrm{H}}_{\mathrm{2}}{\mathrm{O}}\,({\mathrm{\Delta }}_{\mathrm{r}}{\mathrm{H}}^0 = + 42.2\,{\mathrm{kJ}} \cdot {\mathrm{mol}}^{ - 1})$$

Photo-driven direct RWGS would be a highly desirable alternative to (photo)electrocatalysis. But this route also faces a major kinetic challenge, which disfavors CO formation: CO_2_ hydrogenation via molecular hydride catalysts generally leads to formic acid (or methanol) instead of CO^13–16^. This selectivity is a consequence of the normal CO_2_ insertion into M–H bonds giving formates (MO_2_CH)^17^. In contrast, the alternative formation of the hydroxycarbonyl (MCO_2_H) isomer has never been directly observed and is therefore referred to as abnormal CO_2_ insertion^13^.

Nickel pincer complexes were recently studied both as [NiFe]-CODH models and in catalytic CO_2_ hydrogenation to formate^18–21^. Inspired by the biological and synthetic precedence, we here report the photo-driven inversion of the CO_2_ insertion selectivity into a Ni–H bond representing an example of abnormal CO_2_ insertion. The selectivity is defined by photoinduced *trans* N–H reductive elimination as an application of metal-ligand cooperativity in photocatalysis.

## Results

### Thermal vs. photochemical CO_2_ insertion

**Fig. 1:**
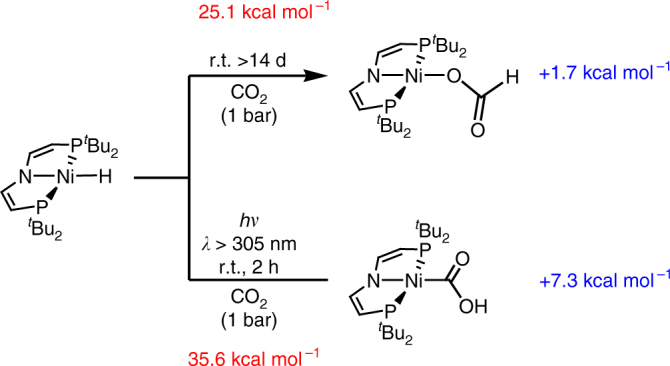
Experimental conditions for normal vs. abnormal CO_2_ insertion. Reactivity of **1** with CO_2_ under thermal and photochemical conditions, respectively, with computed (D3(BJ)-RI-*J*-PBE/def2-SVP//D3(BJ)-TPSS/def2-TZVP(Cosmo:THF)) free reaction enthalpies (Δ_r_*G*^0^(298 K), blue) and effective reaction barriers (Δ*G*^‡^_eff_(298 K) red) for the respective thermal insertions

**Fig. 2:**
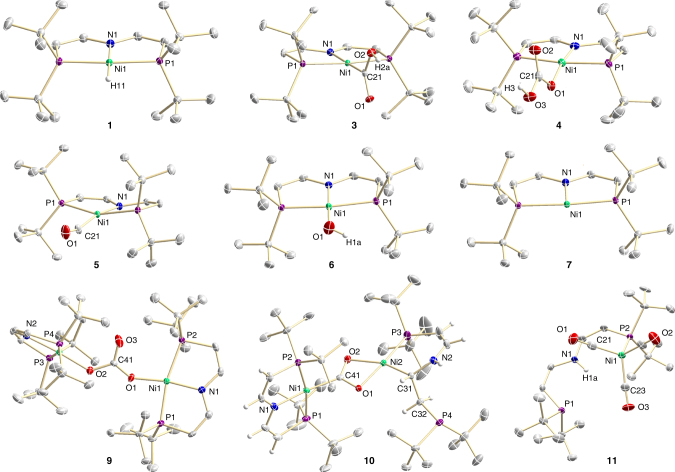
Molecular structures of nickel compounds relevant to CO_2_ activation. Structures of **1**, **3**–**7,** and **9**–**11** in the solid state derived by single-crystal X-ray diffraction. For crystallographic details see Supplementary Figs. 48–56 and Supplementary Tables 19–47

**Fig. 3:**
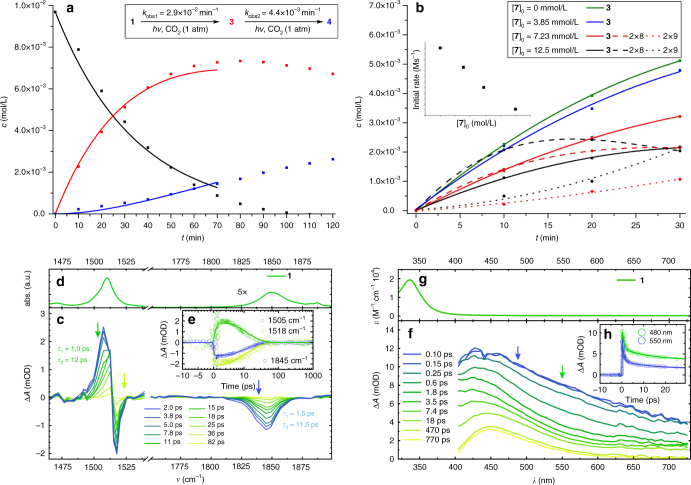
Selected kinetic and spectroscopic data. **a** Kinetic data for bulk photolysis of **1** (*λ* > 305 nm, [**7**]_0_ = 0, THF) and global fit over three half-lives of **1** (solid lines). **b** Initial kinetic data for bulk photolysis of **1** (*λ* > 305 nm, THF) with varying amounts of added **7** (lines are polynomial splines as guide for the eye). **c** Transient IR difference spectra generated by 400 nm excitation of a 11 mM solution of **1** in THF-d_8_ (1750–1900 cm^−1^) and THF (1470–1540 cm^–1^), respectively, for selected pump-probe delays. **d** Stationary FTIR spectrum of **1** in THF-d_8_. **e** Time traces with biexponential fits (time constants: *τ*_1_ = 1.3 ± 0.2 ps, *τ*_2_ = 12 ± 0.5 ps). **f** Transient UV/Vis difference spectra generated by 385 nm excitation of a 6 mM solution of **1** in THF for selected pump-probe delays. **g** Stationary absorption spectrum of **1** in THF. **h** Time traces with triexponential fits (time constants: *τ*_1_ = 0.9 ± 0.2 ps, *τ*_2_ = 13 ± 1 ps, *τ*_3_ ≫ 1 ns)

**Fig. 4:**
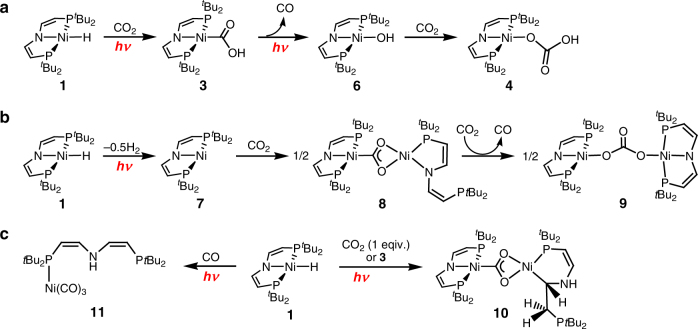
Control experiments for mechanistic rationalization. **a** Photochemical formation of hydrocarbonate **4**. **b** Photochemical formation of nickel(I) and reaction with CO_2_. **c** Photoproduct trapping experiments

Nickel(II) hydride complex [NiH(PNP)] (**1**, PNP = N(CHCHP*t*Bu_2_)_2_) can be prepared from the previously reported bromide [NiBr(PNP)] with LiAlH_4_^22^. As expected, the reaction of **1** with CO_2_ (1–10 bar) at room temperature (r.t.) gives formate complex [Ni(O_2_CH)(PNP)] (**2**, Fig. 1) as the product of normal insertion. However, the reaction is sluggish and takes several days for completion even at *p*(CO_2_) = 10 bar. In contrast to this thermal reactivity, bulk photolysis of **1** (*λ*_exc_ > 305 nm, Supplementary Fig. 15) in benzene or THF under CO_2_ (1 bar) at r.t. results in full conversion of **1** within a few hours (Fig. 1). A quantum yield around *Φ*_P_ = 5% was estimated actinometrically using a ferrioxalate assay (Supplementary Fig. 37). Importantly, the abnormal insertion product [Ni^II^(CO_2_H)(PNP)] (**3**) is formed as the main product in ~ 70% spectroscopic and isolated yield with no indication for formate **2**. In the solid state, the bond metrics of the {NiCO_2_H} entity (Fig. 2) resemble the other previously reported nickel hydroxycarbonyl complex, which was not obtained directly from CO_2_^18^. Besides **3**, small amounts (ca. 20%) of the hydrocarbonate complex [Ni(OCO_2_H)(PNP)] (**4**, Fig. 2) and trace quantities of nickel(I) complex [Ni(CO)(PNP)] (**5**, Fig. 2) are observed by nuclear magnetic resonance (NMR) and electron paramagnetic resonance (EPR) spectroscopy (Supplementary Figs. 28, 29), respectively, as the only side products. Kinetic monitoring of the photolysis (Fig. 3a) indicates slow underlying conversion of **3** to **4** by CO extrusion and CO_2_ insertion as the source of hydrocarbonate and carbonyl side products. Accordingly, photolysis of isolated **3** under argon gives the hydroxy complex [Ni(OH)(PNP)] (**6**, Fig. 2) in high yield within competitive timescales and **6** selectively inserts CO_2_ to hydrocarbonate **4** (Fig. 4a, Supplementary Fig. 16).

### Mechanistic examinations

In agreement with the experimental findings, thermal normal insertion was computed to be slow and strongly favored over endergonic abnormal insertion both thermodynamically and kinetically (Fig. 1). Mechanistic examinations were conducted to rationalize this photochemical selectivity inversion. Photolysis of the [NiD(PNP)] isotopologue under CO_2_ results in almost quantitative transfer of the deuterium label to the hydroxy proton of **3**, thus confirming the hydride ligand as the primary hydrogen source (Supplementary Fig. 25). The reaction rate exhibits first-order dependence in **1** and photon flux, respectively, but is independent from CO_2_ pressure between 1 and 10 bar (Supplementary Figs. 20–25). Therefore, the photochemistry of **1** in the absence of CO_2_ was first studied. Hydride H/D exchange with TEMPO-D (2,2,6,6-tetramethylpiperidinyldeuteroxide; 10 equiv.) is observed only under photolytic conditions (Supplementary Fig. 19). In contrast, H/D scrambling with *t*BuOD is obtained neither in the dark nor upon photolysis, suggesting homolytic rather than heterolytic Ni–H photoactivation. Accordingly, photolysis of **1** in benzene or THF under argon (Fig. 4b) gives [Ni(PNP)] (**7**) as the only detectable product by NMR and EPR spectroscopy upon comparison with an analytically pure sample prepared by reduction of [NiBr(PNP)] with Mg (Supplementary Figs. 17 and 30). Liberation of H_2_ was confirmed by gas chromatography headspace analysis (Supplementary Fig. 18). The rhombic *g*-tensor (*g*_x_ = 2.29, *g*_y_ = 2.25, *g*_z_ = 2.01) and ^14^N superhyperfine interaction (*A*_z_ = 34 MHz) of **7** are in agreement with nickel(I) and some spin delocalization onto the pincer ligand, which is confirmed by density functional theory (DFT) computations (*ρ*_Ni_ = 91%; Supplementary Fig. 42). In analogy to other nickel(I) complexes with similar spectroscopic features^20,23,24,25^, the molecular structure of **7** (Fig. 2) reveals T-shaped nickel coordination.

The observation of the photoreduction products **7** and **5** raises the question about the role of nickel(I) for abnormal insertion, e.g, via a radical chain mechanism. **7** reacts with equimolar amounts of CO_2_ in the dark and under photochemical conditions to one diamagnetic compound (Fig. 4b). NMR spectroscopic characterization of the ^13^CO_2_ isotopologue is in agreement with the formation of a dinuclear CO_2_-bridged (*δ*(^13^C) = 237 ppm) Ni^II^/Ni^II^ complex, in analogy with a related nickel(I) pincer complex^20^. In the current case, the product [(PNP)Ni^II^(1*κC*,2*κ*^2^*O*,*O′*-CO_2_)Ni^II^(*κ*^2^*P*,*N*-PNP)] (**8**) bears one tridentate (*δ*(^31^P) = 67.8 ppm) and one bidentate pincer ligand (*δ*(^31^P) = 72.2 and 4.8 ppm), respectively, presumably as a consequence of increased steric pressure. At 1 bar CO_2_, **8** subsequently eliminates CO and inserts CO_2_ (Fig. 4b) to the dinuclear carbonate bridged complex [(PNP)Ni^II^(OC(O)O)Ni^II^(PNP)] (**9**, Fig. 2). In the presence of **1** (without irradiation) this reactivity of **7** with CO_2_ remains unchanged. Importantly, **1** is not consumed under these conditions disfavoring a radical chain mechanism for abnormal insertion with photochemically produced **7** as chain propagator. Photolysis of mixtures of **1** and **7** ([**7**]_0_ = 0–1.3 [**1**]_0_) under CO_2_ (1 bar) gives rising amounts of binuclear (**9**) vs. mononuclear (**3**) products with increasing [**7**]_0_ (Fig. 3b). In fact, the consumption of **1** even slows with increasing [**7**]_0_. This rate retardation and the diverging selectivities strongly suggest that nickel(I) complex **7** is not an intermediate in abnormal CO_2_ insertion.

### Time-resolved spectroscopy

Transient spectroscopy was employed to further rationalize the photochemistry of **1**. Pump-probe IR-spectroscopy (*λ*_exc_ = 400 nm, THF) reveals an instantaneous bleach of the Ni–H stretching vibration at 1845 cm^–1^ and simultaneous shift of vibrational modes of the vinylic PNP ligand backbone ~1500–1520 cm^–1^ (Fig. 3c–e). The absence of other transient signals within the experimental window (1260–1900 cm^–1^) for both Ni–H/D isotopomers is consistent with the population of a dissociative state or at least strong weakening of the metal-hydride bond upon excitation (Supplementary Figs. 32, 33, 35). Ground state repopulation can be modeled with a biexponential fit. The immediately formed excited state shows a lifetime of *τ*_1_ = 1.3 ps. The timescale of the subsequent spectral evolution (*τ*_2_ = 12 ps) is consistent with vibrational cooling in the electronic ground state of **1**. Although full relaxation is observed for the IR spectrum, transient UV/Vis spectroscopy (Fig. 3f–h, Supplementary Fig. 34; *λ*_exc_ = 400 nm, THF) reveals the evolution of a band at around 450 nm within the same timescale (*τ*_2_ ≈ 13 ps) that is persistent on the timescale of the pump-probe experiment (*τ*_3_ ≫ 1 ns) and does not originate from nickel(I) complex **7** (Supplementary Fig. 31). The absence of IR-bands assignable to this product is attributed to low quantum yields (<5%). Notably, a weak feature at 450 nm is also observed in the stationary UV/Vis spectrum after bulk photolysis of **1** (Supplementary Fig. 31). Importantly, the rapid rate of all photoprocesses well below the timescale of diffusion exclude bimolecular reactivity of an excited state of **1** with CO_2_, which is in line with the rate independence of abnormal insertion from *p*(CO_2_). Instead, **1** apparently forms a photoproduct with low quantum yield that presumably is a common intermediate for photoreduction to **7** or, alternatively, abnormal insertion in the presence of CO_2_.

### Trapping experiments

The timescales of the photoprocesses and the retention of the Ni–D label upon reaction with CO_2_ support an intramolecular rearrangement to the photoproduct, yet not to nickel(I) as suggested by the kinetics. Further information about the photointermediate was obtained from variation of the reaction conditions. Irradiation of **1** in the presence of only one equivalent of CO_2_ gave a new product in ~20% isolated yield (Fig. 4c). Spectroscopic and crystallographic characterization reveal the formation of the unusual, dinuclear complex [(PNP)Ni^II^(1*κC*,2*κ*^2^*O*,*O’*-CO_2_)Ni^II^(*κ*^2^*P*,*C*-*t*Bu_2_PCHCHNHCHCH_2_P*t*Bu_2_)] (**10**, Fig. 2). This product is also obtained upon photolysis of solutions of **1** and **3** under argon suggesting trapping of the photointermediate by **3** at low CO_2_ concentrations. The formation of **10** can be rationalized by O–H oxidative addition of **3** to a nickel(0) photoproduct and subsequent olefin migrative insertion. Initial photoinduced N–H reductive elimination to nickel(0) is further supported by a trapping experiment with CO. Photolysis under CO gives the nickel(0) complex [(CO)_3_Ni{*κP*-HN(CHCHP*t*Bu_2_)_2_}] (**11**, Fig. 2) almost selectively (Fig. 4c). Thermal reductive elimination under CO is also observed^26^, yet on considerably slower timescales compared with the photolytic conditions. Reductive elimination photoreactivity is frequently observed for metal hydrides^27^. Surprisingly, the almost fully released pincer ligand of **11** favors the divinylamine over imine tautomer, in line with the selective transfer of the Ni–D deuteride label to the hydroxy group of **3**. In contrast, cooperative participation of the vinylic backbone, should lead to C–H/D scrambling, which is not observed^22^.

### Computational examinations

**Fig. 5:**
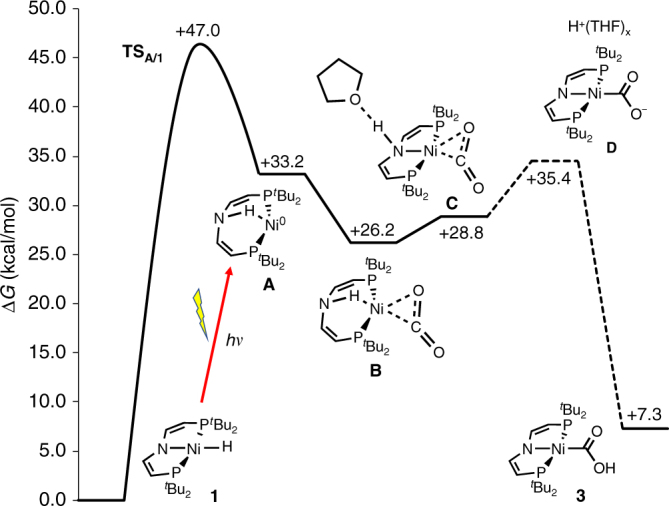
Computational examination of the proposed mechanism. Ground and transition state free energies (D3(BJ)-RI-*J*-PBE/def2-SVP//D3(BJ)-TPSS/def2-TZVP(Cosmo:THF)) for thermal reactivity in THF after photochemical N–H reductive elimination from **1** (red arrow). The dashed free energy for [H(THF)_x_][Ni(CO_2_)(PNP)] (**D**) ion pair formation is estimated from experimental p*K* derivation

This mechanistic picture was further probed by DFT computations (Fig. 5). The *trans*-NH reductive elimination product [Ni^0^(*H*PNP)] (**A**) was found at Δ*G* = 33.2 kcal mol^–1^ relative to parent **1** and close to a THF solvent adduct, [(THF)Ni^0^(*H*PNP)] (**A’**, Δ*G* = 35.9 kcal mol^–1^). In contrast to the Ni^II^ and Ni^I^ complexes **1** and **7**, respectively, TD-DFT predicts several electronic transitions with high oscillator strength in the range 400–450 nm both for **A** and **A’** (Supplementary Fig. 40), as was found by transient UV/Vis spectroscopy (Fig. 3f). Anagostic hydrogen bonding of the amine proton indicates high basicity of the metal ion. For the unproductive oxidative addition back to parent **1**, which fully proceeds on the singlet potential energy surface, a sizable barrier (**TS**_**A/1**_: Δ*G*^‡^ = 13.8 kcal mol^–1^, Supplementary Fig. 45) was found that is in agreement with a persistent photoproduct on the (ps) timescale of the transient spectroscopy experiments. Carbon dioxide binding stabilizes **A** by 7.0 kcal mol^–1^ giving side-on CO_2_ complex [Ni^0^(CO_2_)(*H*PNP)] (**B**). Alternatively, explicit solvent modeling produced intermediate [Ni^0^(CO_2_)(THF-*H*PNP)] (**C**) within 2.6 kcal mol^–1^. Anagostic N–H bonding (**B**) is in **C** replaced by a hydrogen bridge to THF. Such a structure could not be found for **A**, indicating reduced metal basicity upon binding of the π-acceptor CO_2_. Furthermore, nitrogen is directly bound to the metal in **C** more closely resembling the {Ni(PNP)} fragment of final product **3**. This observation suggests structural flexibility of the pincer at low energetic cost. Proton coupled electron transfer to the CO_2_ ligand would then complete the reaction sequence. Several intra- and intermolecular routes are conceivable and were found computationally with kinetically competitive barriers below unproductive N–H oxidative addition (**TS**_**A/1**_). However, such values should be treated with caution without experimental benchmarking owing to the known difficulties for DFT to reliably describe proton transfer reactions with considerable charge buildup and possible direct solute–solvent interactions^28^. We therefore prefer to correlate intermediates **B**/**C** with the free energy for deprotonation of **3** in THF, which is experimentally accessible upon reaction with a reference base (Supplementary Figs. 26, 27), defining an energetic upper limit for *N*-to-*O* proton transfer. Assuming that the proton mobility is sufficient within a [H(THF)_x_Ni(CO_2_)(PNP)] (**D**) ion pair the p*K*_ip_ of **3** (20.6) is used, placing **D** at 35.4 kcal mol^–1^ well below **TS**_**A/1**_, which further supports the proposed mechanism.

**Fig. 6:**
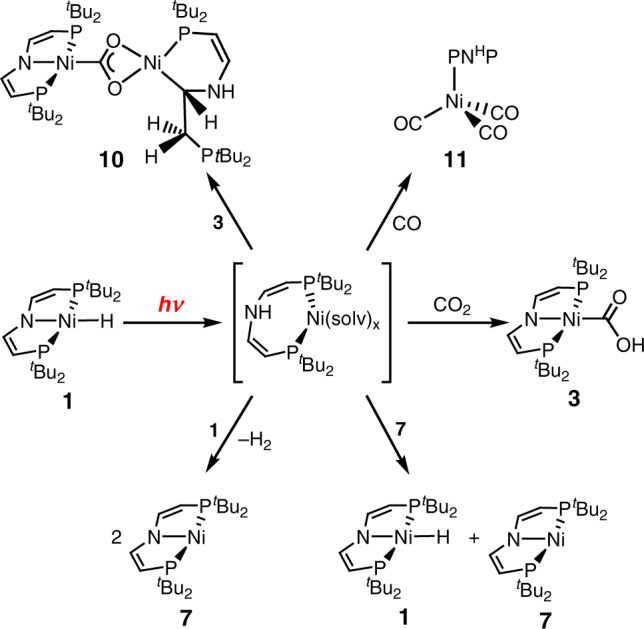
Proposed mechanism. Formation of a nickel(0) photoproduct from photochemical activation of **1** and reactivity with different substrates

In summary, a mechanism that evolved from kinetic, spectroscopic, labeling, and chemical trapping experiments supported by DFT calculations explains the photochemical selectivity inversion. Photochemical excitation populates a dissociative state leading to rate-determining conversion to a photoproduct with an overall quantum yield ~5%. Trapping with CO, **3** and **1**, respectively, the exclusion of nickel(I) as relevant intermediate, the retention of the Ni–D label, the photochemical timescales and the computed UV/Vis spectroscopic signatures all support the nickel(0) assignment for the immediate photoproduct as a result of intramolecular N–H reductive elimination. Hydroxycarbonyl formation presumably follows CO_2_ coordination to nickel(0) and proton coupled electron transfer. Hydrogen atom transfer of the photointermediate with **7** can explain the abnormal insertion rate retardation in the presence of added nickel(I) (Fig. 6).

This model implies the notion that the inversion of the CO_2_ insertion selectivity from normal to abnormal relies on the photochemical umpolung reaction of the hydride ligand to an N–H proton. As a consequence of this electron transfer, the reduced metal center becomes the most basic site and is subject to electrophilic attack by the substrate CO_2_. This mechanistic picture therefore in several ways expresses an application of metal-ligand cooperation (MLC), which is currently extensively exploited as a strategy for catalyst design^29^. On one hand, photochemical transfer of the two reducing equivalents stored within the Ni–H bond to the metal presumably drives the overall endergonic abnormal CO_2_ insertion. On the other hand, the pincer ligand acts as a proton relay and intramolecular proton coupled electron transfer from the amine likely facilitates the CO_2_ two-electron reduction to the hydroxycarbonyl isomer. Hence, MLC might similarly emerge as a valuable conception for photocatalysis.

## Methods

### Synthesis and characterization

[NiH{N(CHCHP*t*Bu_2_)_2_}] (**1**). [NiBr{N(CHCHP*t*Bu_2_)_2_}] (208 mg, 0.420 mmol, 1.00 eq) and LiAlH_4_ (16 mg, 0.422 mmol, 1.00 eq) are dissolved in 6 mL of THF. After stirring at room temperature for 30 mins, the solvent of the orange solution is removed in vacuo. The orange residue is extracted with pentanes and the resulting solution is filtered over Celite to yield a yellow solution. The solvent is removed in vacuo and the yellow solid is washed with 7 × 2 mL MeOH at 0 °C. The residue is dissolved in 10 mL of pentanes and filtered. After removal of the solvent in vacuo, the yellow solid is dissolved in a minimal amount of pentanes and recrystallized at −36 °C. The supernatant solution is decanted, and again recrystallized at −36 °C. The yellow crystalline material is dried in vacuo to yield 106 mg (0.255 mmol, 61%) of **1**. Crystals suitable for X-ray diffraction are obtained by crystallization from pentanes at −36 °C. ^31^P{^1^H} NMR (121 MHz, C_6_D_6_) δ: 86.2 ppm. ^1^H NMR (300 MHz, C_6_D_6_) δ: 7.20 (ABXX’B’A’, *N* = │^3^*J*_A-X_ + ^4^*J*_A-X’_│ = 18.3 Hz, ^3^*J*_A-B_ = 5.0 Hz, ^4^*J*_H-H_ = 1.9 Hz, Hz, 2 H, NC*H*), 4.09 (ABXX’B’A’, *N* = │^2^*J*_A-X_ + ^4^*J*_A-X’_│ = 1.8 Hz, ^3^*J*_A-B_ = 5.1 Hz, ^4^*J*_H-H_ = 1.8 Hz, 2 H, PC*H*), (A_18_XX’A’_18_, *N* = │^3^*J*_A-X_ + ^5^*J*_A-X_│ = 6.7 Hz, 36 H, P*tBu*), −18.52 (tp, ^2^*J*_P-H_ = 59.4 Hz, ^4^*J*_H-H_ = 2.0 Hz, 1 H, Ni*H*) ppm. ^13^C{^1^H} NMR (75 MHz, C_6_D_6_) δ: 160.8 (AXX’A’, *N* = │^2^*J*_A-X_ + ^3^*J*_A-X’_ │ = 11.8 Hz, N*C*H), 83.0 (AXX’A’, *N* = │^1^*J*_A-X_ + ^3^*J*_A-X’_│ = 15.8 Hz, P*C*H), 33.8 (A_2_XX’A’_2_, *N* = │^1^*J*_A-X_ + ^3^*J*_A-X’_ │ = 11.1 Hz, P*C*Me_3_), 29.8 (A_6_XX’A’_6_, *N* = │^2^*J*_A-X_ + ^4^*J*_A-X’_ │ = 2.9 Hz, PC*Me*_3_) ppm. IR (KBr): ṽ = 1834.3 cm^−1^. Anal. Calcd. For C_40_H_41_NNiP_2_ (416.20): C, 57.72; H, 9.93; N, 3.37. Found: C, 57.48 H, 9.80; N, 3.41. MS (LIFDI): *m*/*z* (%) 415.1 (100).

[NiD{N(CHCHP*t*Bu_2_)_2_}] (**1-D**). [NiBr{N(CHCHP*t*Bu_2_)_2_}] (150 mg, 0.303 mmol, 1.00 eq) and LiAlD_4_ (13 mg, 0.310 mmol, 1.02 eq) are dissolved in 6 mL of THF. After stirring at room temperature for 30 mins, the solvent of the orange solution is removed in vacuo. The orange residue is extracted with pentanes and the resulting solution is filtered over Celite to yield a yellow solution. The solvent is removed in vacuo and the yellow solid is washed with 7 × 2 mL MeOH at 0 °C. The residue is dissolved in 10 mL of pentanes and filtered. After removal of the solvent in vacuo, the yellow solid is dissolved in a minimal amount of pentanes and recrystallized at −36 °C. The supernatant solution is decanted, and again recrystallized at −36 °C. The yellow crystalline material is dried in vacuo to yield 69 mg (0.165 mmol, 55%) of **1-D**. ^2^H NMR (46 MHz, C_6_H_6_) δ: −17.50 (t, ^2^*J*_H-P_ = 9.0 Hz, Ni*D*) ppm. IR (THF-d_8_): ṽ = 1318, 1333 cm^−1^.

[Ni(OCHO){N(CHCHP*t*Bu_2_)_2_}] (**2**). Complex **1** (2.0 mg, 4.8 µmol, 1.00 eq) is dissolved in 0.3 mL of THF-d_8_ and filled into a medium wall precision pressure/vacuum valve NMR tube. The sample is degassed by three freeze–pump–thaw cycles. A total of 10 atm CO_2_ pressure (≥99.5% purity, no further purification) is applied. After 14 days, nearly complete conversion of **1** to **2** is detected. ^31^P{^1^H} NMR (203 MHz, THF-d_8_) δ: 56.1 ppm. ^1^H NMR (500 MHz, THF-d_8_) δ: 7.25 (t, ^4^*J*_H-P_ = 3.4 Hz, 1 H, OCO*H*), 6.49 (ABXX’B’A’, *N* = │^3^*J*_A-X_ + ^4^*J*_A-X’_│ = 19.1 Hz, ^3^*J*_A-B_ = 5.5 Hz, 2 H, NC*H*), 4.09 (ABXX’B’A’, *N* = │^2^*J*_A-X_ + ^4^*J*_A-X’_│ = 2.0 Hz, ^3^*J*_A-B_ = 5.5 Hz, 2 H, PC*H*), 1.51 (A_18_XX’A’_18_, *N* = │^3^*J*_A-X_ + ^5^*J*_A-X’_│ = 6.8 Hz, 36 H, P*tBu*) ppm. ^13^C{^1^H} NMR (126 MHz, THF-d_8_) δ: 168.3 (t, ^3^*J*_C-P_ = 1.1 Hz, O*C*OH), 162.9 (AXX’A’, *N* = │^2^*J*_A-X_ + ^3^*J*_A-X’_ │ = 11.0 Hz, N*C*H), 83.0 (dd, ^1^*J*_C-P_ = 19.6 Hz, ^3^*J*_C-P_ = 18.7 Hz, P*C*H), 35.8 (A_2_XX’A’_2_, *N* = │^1^*J*_A-X_ + ^3^*J*_A-X’_ │ = 8.5 Hz, P*C*Me_3_), 29.0 (A_6_XX’A’_6_, *N* = │^2^*J*_A-X_ + ^4^*J*_A-X’_ │ = 2.8 Hz, PC*Me*_3_) ppm.

[Ni(CO_2_H){N(CHCHP*t*Bu_2_)_2_}] (**3**). A solution of **1** (20 mg, 0.048 mmol, 1.00 eq) in benzene (6 mL) is degassed by three pump–freeze–thaw cycles and set under 1 atm of CO_2_ (≥99.5% purity, no further purification). The reaction mixture is photolyzed (*λ*_exc_ > 305 nm) for 8 hours, followed by washing with 5 × 2 mL of benzene and 5 × 2 mL of pentanes. The product is dissolved in THF and dried in vacuo to yield **3** (15 mg, 0.035 mmol, 73%) as a yellow solid. Crystals suitable for X-ray diffraction are obtained by diffusion of pentanes into a solution of **3** in THF. ^31^P{^1^H} NMR (162 MHz, THF-d_8_) δ: 66.2 ppm. ^1^H NMR (400 MHz, THF-d_8_) δ: 9.51 (br, 1 H, CO_2_*H*), 6.49 (ABXX’B’A’, *N* = │^3^*J*_A-X_ + ^4^*J*_A-X’_│ = 18.4 Hz, ^3^*J*_A-B_ = 5.3 Hz, 2 H, NC*H*), 4.02 (ABXX’B’A’, *N* = │^2^*J*_A-X_ + ^4^*J*_A-X’_│ = 2.0 Hz, ^3^*J*_A-B_ = 5.2 Hz, 2 H, PC*H*), 1.37 (A_18_XX’A’_18_, *N* = │^3^*J*_A-X_ + ^5^*J*_A-X’_│ = 6.7 Hz, 36 H, P*tBu*) ppm. ^13^C{^1^H} NMR (126 MHz, THF-d_8_) δ: 203.6 (t, ^2^*J*_C-P_ = 31.1 Hz, *C*O_2_H), 161.1 (AXX’A’, *N* = │^2^*J*_A-X_ + ^3^*J*_A-X’_ │ = 10.7 Hz, N*C*H), 82.4 (AXX’A’, *N* = │^1^*J*_A-X_ + ^3^*J*_A-X’_ │ = 18.6 Hz, P*C*H), 35.9 (A_2_XX’A’_2_, *N* = │^1^*J*_A-X_ + ^3^*J*_A-X’_ │ = 10.2 Hz, P*C*Me_3_), 29.3 (A_6_XX’A’_6_, *N* = │^2^*J*_A-X_ + ^4^*J*_A-X’_ │ = 2.7 Hz, PC*Me*_3_) ppm. IR (KBr): ṽ = 2645, 1584, 1565 cm^−1^. Anal. Calcd. For C_41_H_41_NO_2_NiP_2_ (460.20): C, 54.81; H, 8.98; N, 3.04. Found: C, 54.92 H, 8.98; N, 2.91. MS (LIFDI): *m*/*z* (%) 459.0 (100).

[Ni(OCO_2_H){N(CHCHP*t*Bu_2_)_2_}] (**4**). A solution of [Ni(OH){N(CHCHP*t*Bu_2_)_2_}] (**6**) in THF-d_8_ is degassed by three pump–freeze–thaw cycles set under 1 atm CO_2_ ( ≥ 99.9993% purity, purification by passing through P_4_O_10_, Drierite and cooling to −40 °C) in a J-Young NMR tube. Crystals of **4** suitable for X-ray diffraction are obtained by diffusion of pentanes on a THF solution. ^31^P{^1^H} NMR (162 MHz, THF-d_8_) δ: 54.6 ppm. ^1^H NMR (400 MHz, THF-d_8_) δ: 9.28 (br, 1 H, OCO_2_*H*), 6.47 (ABXX’B’A’, *N* = │^3^*J*_A-X_ + ^4^*J*_A-X’_│ = 19.1 Hz, ^3^*J*_A-B_ = 5.5 Hz, 2 H, NC*H*), 3.88 (ABXX’B’A’, *N* = │^2^*J*_A-X_ + ^4^*J*_A-X’_│ = 2.0 Hz, ^3^*J*_A-B_ = 5.4 Hz, 2 H, PC*H*), 1.48 (A_18_XX’A’_18_, *N* = │^3^*J*_A-X_ + ^5^*J*_A-X’_│ = 6.8 Hz, 36 H, P*tBu*) ppm. ^13^C{^1^H} NMR (126 MHz, THF-d_8_) δ: 163.0 (AXX’A’, *N* = │^2^*J*_A-X_ + ^3^*J*_A-X’_ │ = 11.1 Hz, N*C*H), 158.9 (s, O*C*O_2_H), 82.2 (AXX’A’, *N* = │^1^*J*_A-X_ + ^3^*J*_A-X’_ │ = 18.8 Hz, P*C*H), 35.7 (A_2_XX’A’_2_, *N* = │^1^*J*_A-X_ + ^3^*J*_A-X’_ │ = 8.6 Hz, P*C*Me_3_), 29.0 (A_6_XX’A’_6_, *N* = │^2^*J*_A-X_ + ^4^*J*_A-X’_ │ = 2.9 Hz, PC*Me*_3_) ppm. Evaporation of a solution of **4** results in CO_2_ elimination and clean reformation of **6**.

[Ni(CO){N(CHCHP*t*Bu_2_)_2_}] (**5**). A solution of [Ni{N(CHCHP*t*Bu_2_)_2_}] **7** (10 mg, 0.024 mmol, 1.00 eq) in THF (0.5 mL) is degassed by three freeze–pump–thaw cycles and set under 1 atm of CO in a J-Young NMR tube. The solution is shaken at r.t. for 30 mins and the solvent is evaporated. The black residue is dissolved in pentanes and filtrated. Evaporation of the solvent and drying in vacuo gives 9 mg (0.020 mmol, 83%) of **5** as black solid. Crystals suitable for X-ray diffraction are obtained by crystallization from Et_2_O at −36 °C. IR (KBr): ṽ = 1910.1 (CO) cm^−1^. MS (LIFDI): *m*/*z* (%) 414.1 (55), 442.1 (45). For EPR characterization see Supplementary Figs. 28, 29.

[Ni(OH){N(CHCHP*t*Bu_2_)_2_}] (**6**). [NiBr{N(CHCHP*t*Bu_2_)_2_}] (24 mg, 0.048 mmol, 1.00 eq), KOH (30 mg, 0.535 mmol, 11.15 eq) and 15-crown-5 (10 µL, 0.051 mmol, 1.06 eq) are filled in a J-Young NMR tube and dissolved in 1 mL THF. The solution is warmed to 70 °C for 2 days. After evaporation of the solvent, the orange solid is extracted with pentanes and filtered over celite. The solvent is evaporated and the orange powder is dissolved in pentanes and recrystallized at −36 °C. Removal of the solvent and drying in vacuo yields red, crystalline **6** (14 mg, 0.033 mmol, 69%). Crystals suitable for X-ray diffraction are obtained by crystallization from pentanes at −36 °C. ^31^P{^1^H} NMR (162 MHz, THF-d_8_) δ: 50.3 ppm. ^1^H NMR (400 MHz, THF-d_8_) δ: 6.62 (ABXX’B’A’, *N* = │^3^*J*_A-X_ + ^4^*J*_A-X’_│ = 18.8 Hz, ^3^*J*_A-B_ = 5.4 Hz, 2 H, NC*H*), 3.88 (ABXX’B’A’, *N* = │^2^*J*_A-X_ + ^4^*J*_A-X’_│ = 1.9 Hz, ^3^*J*_A-B_ = 5.3 Hz, 2 H, PC*H*), 1.37 (A_18_XX’A’_18_, *N* = │^3^*J*_A-X_ + ^5^*J*_A-X’_│ = 6.6 Hz, 36 H, P*tBu*), −4.88 (t, ^3^*J*_H-P_ = 5.6 Hz, O*H*) ppm. ^13^C{^1^H} NMR (126 MHz, THF-d_8_) δ: 162.8 (AXX’A’, *N* = │^2^*J*_A-X_ + ^3^*J*_A-X’_ │ = 11.8 Hz, N*C*H), 81.2 (AXX’A’, *N* = │^1^*J*_A-X_ + ^3^*J*_A-X’_ │ = 18.3 Hz, P*C*H), 35.2 (A_2_XX’A’_2_, *N* = │^1^*J*_A-X_ + ^3^*J*_A-X’_ │ = 8.6 Hz, P*C*Me_3_), 29.3 (A_6_XX’A’_6_, *N* = │^2^*J*_A-X_ + ^4^*J*_A-X’_ │ = 2.9 Hz, PC*Me*_3_) ppm. IR (Nujol): ṽ = 3643.8 cm^−1^. Anal. Calcd. For C_40_H_41_NONiP_2_ (132.19): C, 55.58; H, 9.56; N, 3.24. Found: C, 55.50 H, 9.46; N, 3.12. MS (LIFDI): *m*/*z* (%) 431.2 (100).

[Ni{N(CHCHP*t*Bu_2_)_2_}] (**7**). [NiBr{N(CHCHP*t*Bu_2_)_2_}] (80 mg, 0.162 mmol, 1.00 eq) and magnesium powder (78 mg, 3.21 mmol, 19.8 eq) are suspended in 5 mL THF and stirred for 30 mins at room temperature accompanied by a color change to orange. The solvent is evaporated, and the residue is dissolved in pentanes and filtered over celite. The solution is dried in vacuo and the orange powder is dissolved in a minimum amount of pentanes and recrystallized at –36 °C. The mother liquor is decanted after 3 days and this procedure is repeated two times, giving 42 mg (0.101 mmol, 62%) of **7** as orange crystals. Crystals suitable for X-ray diffraction are obtained by crystallization from pentanes at −36 °C. ^1^H NMR (400 MHz, C_6_D_6_) δ: 7 (br, *t*Bu), −62 (br, C*H*) ppm. Anal. Calcd. For C_20_H_40_NP_2_Ni (415.19): C, 57.86; H, 9.71; N, 3.37. Found: C, 57.59 H, 9.48; N, 3.27. MS (LIFDI): *m*/*z* (%) 414.1 (100).

[{N(CHCHP*t*Bu_2_)_2_}Ni(*1κC*,*2κ*^*2*^*O*,*O’-*^*13*^CO_2_)Ni{*κ*^*2*^*P*,*N-*N(CHCHP*t*Bu_2_)_2_}] (**8**). Complex **7** (5.0 mg, 0.012 mmol, 1.00 eq) is dissolved in 0.5 mL C_6_D_6_ in a J-Young NMR tube and 300 µL (0.012 mmol, 1.00 eq) ^13^CO_2_ are added with a syringe. The solution is kept at room temperature for 24 h. ^31^P{^1^H} NMR (162 MHz, C_6_D_6_) δ: 72.2 (dd, ^5^*J*_P-P_ = 8.7 Hz, ^3^*J*_P-C_ = 2.4 Hz, 1 P, (PNP)NiCO_2_Ni(*PN*)), 67.8 (d, ^2^*J*_P-C_ = 29.1 Hz, 2 P, (*PNP*)NiCO_2_Ni(PN)), 4.77 (d, ^5^*J*_P-P_ = 8.6 Hz, 1 P, non-coordinating pincer arm) ppm.^1^H NMR (400 MHz, C_6_D_6_) δ: 8.23 (ddd, ^3^*J*_H-P_ = 42.4 Hz, ^4^*J*_H-P_ = 5.5 Hz, ^3^*J*_H-H_ = 5.5 Hz, 1 H, NCH non-coordinating pincer arm), 6.86 (ABXX’B’A’, *N* = │^3^*J*_A-X_ + ^4^*J*_A-X’_│ = 18.4 Hz, ^3^*J*_A-B_ = 5.2 Hz, 2 H, NC*H*), 6.43 (dd, ^3^*J*_H-P_ = 20.8 Hz, ^2^*J*_H-H_ = 10.5 Hz, 1 H, NCH coordinating pincer arm), 4.69 (dd, ^2^*J*_H-H_ = 10.5 ppm, ^2^*J*_H-P_ = 6.4 Hz, 1 H, PCH coordinating pincer arm), 3.95 (m, 2 H, PC*H*), 3.40 (dd, ^2^*J*_H-P_ = 4.8 Hz, ^2^*J*_H-H_ = 4.8 Hz, 1 H, PCH non-coordinating pincer arm), 1.60 (m, 36 H, P*tBu*), 1.47 (d, ^3^*J*_H-P_ = 13.6 Hz, 18 H, P*t*Bu coordinating pincer arm), 1.25 (d, ^3^*J*_H-P_ = 10.9 Hz, 18 H, P*t*Bu non-coordinating pincer arm) ppm. ^13^C{^1^H} NMR (101 MHz, C_6_D_6_) δ: 236.7 (t, ^2^*J*_C-P_ = 29.1 Hz, ^3^*J*_C-P_ = 2.4 Hz, *C*O_2_), 165.8 (dd, ^2^*J*_C-P_ = 23.0 Hz, ^3^*J*_C-P_ = 13.7 Hz, NCH non-coordinating pincer arm), 160.9 (AXX’A’, *N* = │^2^*J*_A-X_ + ^3^*J*_A-X’_│ = 10.4 Hz, N*C*H), 149.8 (d, ^2^*J*_C-P_ = 14.1 Hz, N*C*H), 96.4 (d, ^1^*J*_C-P_ = 18.8 Hz, P*C*H), 82.3 (AXX’A’, *N* = │^1^*J*_A-X_ + ^3^*J*_A-X’_│ = 18.6 Hz, P*C*H), 74.9 (d, ^1^*J*_P-C_ = 47.5, PCH non-coordinating pincer arm), 36.0 (A_2_XX’A’_2_, *N* = │^1^*J*_A-X_ + ^3^*J*_A-X’_ │ = 10.3 Hz, P*C*Me_3_), 35.0 (d, ^1^*J*_C-P_ = 20.9 Hz, P*C*Me_3_), 32.4 (d, ^1^*J*_C-P_ = 19.5 Hz, P*C*Me_3_), 30.0 (d, ^2^*J*_C-P_ = 14.3 Hz, PC*Me*_3_), 29.7 (br, PC*Me*_3_), 28.9 (d, ^1^*J*_C-P_ = 4.1 Hz, P*C*Me_3_), 30.0 (d, ^2^*J*_C-P_ = 14.3 Hz, PC*Me*_3_) ppm. Assignment of signals is based on selectively decoupled ^1^H{^31^P} and 2D NMR spectra. MS (LIFDI): *m*/*z* (%) 872.3 (100).

[{N(CHCHP*t*Bu_2_)_2_}Ni(OC(O)O)Ni{N(CHCHP*t*Bu_2_)_2_}] (**9**). Complex **7** (3.2 mg, 0.008 mmol, 1.00 eq) and 3 µL O(SiMe_3_)_2_ as internal standard are dissolved in 0.5 mL THF-d_8_. The solution is degassed by three pump–freeze–thaw cycles and 1 atm CO_2_ (≥99.9993% purity, purification by passing through P_4_O_10_, Drierite and cooling to −40 °C) is applied. The solution is stirred at room temperature for 11 days. Crystals suitable for X-ray diffraction are obtained by crystallization from pentanes at −36 °C. ^31^P{^1^H} NMR (162 MHz, THF-d_8_) δ: 49.2 ppm. ^1^H NMR (400 MHz, THF-d_8_) δ: 6.31 (ABXX’B’A’, *N* = │^3^*J*_A-X_ + ^4^*J*_A-X’_│ = 17.9 Hz, ^3^*J*_A-B_ = 5.6 Hz, 4 H, NC*H*), 3.80 (d, ^3^*J*_H-H_ = 5.6 Hz, 4 H, PC*H*), 1.50 (A_18_XX’A’_18_, *N* = │^3^*J*_A-X_ + ^5^*J*_A-X’_│ = 6.7 Hz, 72 H, P*t*Bu), ppm. ^13^C{^1^H} NMR (126 MHz, C_6_D_6_) δ: 162.5 (*s*, O*C*(O)O) ppm. MS (LIFDI): *m*/*z* (%) 889.3 (100, ^13^CO_3_^2–^-isotopologue).

[{N(CHCHP*t*Bu_2_)_2_}Ni(*1κC*,*2 κ*^*2*^*O*,*O’-*CO_2_)Ni{*κ*^*2*^*P*,*C-t*Bu_2_PCHCHNHCHCH_2_P*t*Bu_2_)}] (**10**). Synthesis from **1** and CO_2_: Complex **1** (30 mg, 0.072 mmol, 1.00 eq) is dissolved in 3 mL benzene and 1.8 mL CO_2_ (0.074 mmol, 1.03 eq, ≥99.9993% purity, purification by passing through P_4_O_10_, Drierite and cooling to −40 °C) are added by syringe. The solution is photolyzed (*λ*_exc_ > 305 nm) over night and the solvent is evaporated. The residue is dissolved in pentanes, filtered, and the solvent is evaporated. The residue is suspended in 1 mL acetonitrile and diethyl ether is added dropwise until the solid is dissolved completely. Repeated crystallization at –36 °C and subsequent drying in vacuo gives orange **10** (7 mg, 0.008 mmol, 22%). Crystals suitable for X-ray diffraction are obtained by crystallization from pentanes at −36 °C. Synthesis from **1** and **3**: **3** (1.5 mg, 3.3 µmol, 1.00 eq) and **1** (1.4 mg, 3.4 µmol, 1.03 eq) are dissolved in 0.5 mL THF and filled in a J-Young NMR tube and the sample is photolyzed (*λ*_exc_ > 305 nm). ^31^P{^1^H} NMR (203 MHz, C_6_D_6_) δ: 66.2 (d. ^2^*J*_P-P_ = 197.3 Hz, 1 P, (*PNP*)NiCO_2_Ni(PC)), 65.0 (d. ^2^*J*_P-P_ = 197.1 Hz, 1 P, (*PNP*)NiCO_2_Ni(PC)), 36.8 (d, ^4^*J*_P-P_ = 7.4 Hz, 1 P, (PNP)NiCO_2_Ni(*PC*)), 17.1 (d, ^4^*J*_P-P_ = 7.2 Hz, 1 P, non-coordinating pincer arm) ppm. ^1^H NMR (500 MHz, C_6_D_6_) δ: 6.96 (m, 2 H, NC*H*), 6.48 (ddd, ^2^*J*_H-P_ = 27.2 Hz, ^3^*J*_H-H_ = 9.6 Hz, ^4^*J*_H-H_ = 7.5 Hz, 1 H, PC*H*), 4.37 (dd, ^4^*J*_H-P_ = 7.4 Hz, ^4^*J*_H-H_ = 7.4 Hz, 1 H, N*H*), 4.02 (m, 2 H, PC*H*), 3.52 (d, ^3^*J*_H-H_ = 9.6 Hz, 1 H, NCH), 2.92 (dddd, ^3^*J*_H-H_ = 12.4 Hz, ^3^*J*_H-P_ = 6.1 Hz, ^3^*J*_H-H_ = 1.9 Hz, ^3^*J*_H-H_ = 1.9 Hz, 1 H, NC*H*CH_2_), 2.72 (ddd, ^2^*J*_H-H_ = 14.3 Hz, ^2^*J*_H-P_ = 5.9 Hz, ^3^*J*_H-H_ = 2.1 Hz, 1 H, NCHC*H*H), 1.73 (m, 9 H, P*tBu*), 1.68 (m, 9 H, P*tBu*), 1.59 (m, 9 H, P*tBu*), 1.51 (m, 9 H, P*tBu*), 1.50 (d, ^3^*J*_H-P_ = 12.9 Hz, 9 H, P*tBu*), 1.47 (d, ^3^*J*_H-P_ = 13.1 Hz, 9 H, P*tBu*), 1.47 (NCHCH*H*, detected by ^1^H,^1^H COSY NMR), 1.32 (d, ^3^*J*_H-P_ = 10.7 Hz, 9 H, P*tBu*), 1.19 (d, ^3^*J*_H-P_ = 10.9 Hz, 9 H, P*tBu*) ppm. ^13^C{^1^H} NMR (126 MHz, C_6_D_6_) δ: 228.3 (dt, ^2^*J*_C-P_ = 28.5 Hz, ^3^*J*_C-P_ = 3.0 Hz, Ni*C*O_2_Ni), 160.7 (dd, ^3^*J*_C-P_ = 13.9 Hz, ^2^*J*_C-P_ = 7.3 Hz, N*C*H), 160.7 (dd, ^3^*J*_C-P_ = 14.0 Hz, ^2^*J*_C-P_ = 7.1 Hz, N*C*H), 149.0 (d, ^2^*J*_C-P_ = 7.3 Hz, N*C*H), 82.6 (dd, ^1^*J*_C-P_ = 33.3 Hz, ^3^*J*_C-P_ = 9.4 Hz, P*C*H), 82.4 (dd, ^1^*J*_C-P_ = 32.5 Hz, ^3^*J*_C-P_ = 9.5 Hz, P*C*H), 68.0 (dd, ^1^*J*_C-P_ = 50.7 Hz, P*C*H), 36.2 (dd, ^1^*J*_C-P_ = 13.8, ^3^*J*_C-P_ = 6.0 Hz, P*C*Me_3_), 35.9 (dd, ^1^*J*_C-P_ = 14.0, ^3^*J*_C-P_ = 5.8 Hz, P*C*Me_3_), 35.8 (dd, ^1^*J*_C-P_ = 14.2, ^3^*J*_C-P_ = 6.0 Hz, P*C*Me_3_), 35.4 (dd, ^1^*J*_C-P_ = 14.2, ^3^*J*_C-P_ = 6.4 Hz, P*C*Me_3_), 35.0 (d, ^1^*J*_C-P_ = 21.4, P*C*Me_3_), 34.6 (d, ^1^*J*_C-P_ = 24.4, P*C*Me_3_), 33.2 (dd, ^2^*J*_C-P_ = 30.4 Hz, ^2^*J*_C-P_ = 4.1 Hz, N*C*HNi), 32.6 (d, ^1^*J*_C-P_ = 22.9 Hz, P*C*Me_3_), 31.2 (d, ^1^*J*_C-P_ = 21.6 Hz, P*C*Me_3_), 30.5 (d, ^2^*J*_C-P_ = 12.7 Hz, PC*Me*_3_), 30.2 (dd, ^2^*J*_C-P_ = 3.9 Hz, ^4^*J*_C-P_ = 1.8 Hz, PC*Me*_3_), 30.1 (dd, ^2^*J*_C-P_ = 3.8 Hz, ^4^*J*_C-P_ = 1.8 Hz, PC*Me*_3_), 39.9 (d, ^1^*J*_C-P_ = 13.3 Hz, P*C*Me_3_), 29.7 (d, ^2^*J*_C-P_ = 5.0 Hz, 2xP*CMe*_3_), 29.7 (dd, ^2^*J*_C-P_ = 3.7 Hz, ^4^*J*_C-P_ = 1.8 Hz, PC*Me*_3_), 29.6 (dd, ^2^*J*_C-P_ = 3.7 Hz, ^4^*J*_C-P_ = 1.6 Hz, PC*Me*_3_), 29.4 (d, ^1^*J*_C-P_ = 26.9 Hz, *C*H_2_) ppm. IR (THF-d_8_): ṽ = 1526.4, 1611.3, 3333.5 cm^−1^. MS (LIFDI): *m*/*z* (%) 873.4 (50), 1392.5 (50).

[(CO)_3_Ni{*κP*-HN(CHCHP*t*Bu_2_)_2_}] (**11**). A solution of **1** (9.69 mM, 0.5 mL) in THF-d_8_ and O(SiMe_3_)_2_ as internal standard are filled into a J-Young NMR tube, degassed by three pump–freeze–haw cycles and 1 atm of CO applied. The sample is photolyzed (*λ*_exc_ > 305 nm) for 90 minutes. Crystals of **11** suitable for X-ray diffraction are obtained by crystallization from acetonitrile at −36 °C. ^31^P{^1^H} NMR (162 MHz, THF-d_8_) δ: 39.3 (*P*Ni(CO)_3_), −5.2 ppm. ^1^H NMR (400 MHz, THF-d_8_) δ: 8.10 (dd, ^3^*J*_H-H_ = 12.3 Hz, ^3^*J*_H-H_ = 12.3 Hz, 1 H, N*H*), 6.92 (dddd, ^3^*J*_H-P_ = 24.8 Hz, ^3^*J*_H-H_ = 13.0 Hz, ^3^*J*_H-H_ = 10.8 Hz, ^4^*J*_H-H_ = 2.4 Hz, 1 H, NC*H*CHPNi(CO)_3_), 6.88 (ddd, ^3^*J*_H-P_ = 18.1 Hz, ^3^*J*_H-H_ = 11.6 Hz, ^3^*J*_H-H_ = 9.5 Hz, 1 H, NC*H*), 4.74 (dd, ^3^*J*_H-H_ = 9.5 Hz, ^2^*J*_H-P_ = 4.8 Hz, 1 H, PC*H*), 4.46 (dd, ^3^*J*_H-H_ = 10.7 Hz, ^2^*J*_H-P_ = 2.4 Hz, 1 H, *H*CPNi(CO)_3_), 1.24 (d, ^3^*J*_H-P_ = 13.3 Hz, 18 H, *tBu*_2_PNi(CO)_3_), 1.23 (d, ^3^*J*_H-P_ = 12.3 Hz, 18 H, P*tBu*_*2*_) ppm. ^13^C{^1^H} NMR (101 MHz, THF-d_8_) δ: 198.5 (d, ^2^*J*_C-P_ = 3.8 Hz, Ni(*C*O)_3_), 145.5 (d, ^2^*J*_C-P_ = 5.8 Hz, N*C*H), 145.0 (d, ^2^*J*_C-P_ = 20.4 Hz, N*C*H), 95.3 (d, ^1^*J*_C-P_ = 17.1 Hz, P*C*H), 84.9 (d, ^1^*J*_C-P_ = 30.7 Hz, P*C*H), 35.9 (d, ^1^*J*_C-P_ = 35.9 Hz, P*C*Me_3_), 31.9 (d, ^1^*J*_C-P_ = 16.3 Hz, P*C*Me_3_), 29.7 (d, ^2^*J*_C-P_ = 13.8 Hz, P*CMe*_3_), 29.2 (d, ^2^*J*_C-P_ = 7.1 Hz, P*CMe*_3_) ppm. IR (Nujol): ṽ = 3316.5, 2062.7, 1987.9 cm^−1^. MS (LIFDI): *m*/*z* (%) 443.4 (100). Evaporation in vacuo results in CO loss and reformation of **1**.

### Data availability

Detailed descriptions of experimental and spectroscopic methods and results are available within this paper and its supplementary information files. For NMR spectra of the compounds in this article, see Supplementary Figs 1–14. For quantum chemical methods and results see Supplementary Figs. 38–47 and Supplementary Tables 1–18. The crystallographic data CCDC-1561988–1561994, CCDC-1574302, and CCDC-1574303 can be obtained free of charge from the Cambridge Crystallographic Data Centre (www.ccdc.cam.ac.uk/data_request/cif). All other data are available from the authors upon reasonable request.

## Electronic supplementary material

Supplementary Information(PDF 6698 kb)
