# Supplementary material for: The elusive abnormal CO2 insertion enabled by metal-ligand cooperative photochemical selectivity inversion
Source: Nat Commun. 2018 Mar 21;9:1161. doi: 10.1038/s41467-018-03239-3 (PMC5862843; doi:10.1038/s41467-018-03239-3)
Supplement: Supplementary file 1 — Supplementary Information(PDF 6698 kb) [file 41467_2018_3239_MOESM1_ESM.pdf]

The Elusive Abnormal CO_2 Insertion Enabled by Metal-Ligand Cooperative Photochemical Selectivity Inversion

Schneck et al.

Supplementary Methods

All experiments were performed under inert conditions using standard Schlenk and glove-box techniques (argon atmosphere). Solvents were purchased in HPLC quality (Sigma Aldrich) and dried using an MBraun Solvent Purification System. THF was additionally dried over Na/K. Deuterated solvents were obtained from Deutero GmbH and dried over Na/K (d_6-benzene, THF-d_8). Magnesium, potassium hydroxide, lithium aluminum hydride (95%), $^{13}CO_2$ and D_2 were purchased from Sigma Aldrich and used without further purification. Lithium aluminum deuteride (98% isotopic purity) was purchased from Strem chemicals and used without further purification. Hexamethyldisiloxane (HMDSO) was purchased from Sigma Aldrich, purified by distillation and stored over molecular sieves (3 Å). CO (≥99.997% purity) was purchased from Air Liquide. CO_2 was purchased from Linde. [NiBr{N(CHCHP*t*Bu$_2$)$_2$}] was prepared according to the literature.(*1*) TEMPO-D was prepared according to literature, but with use of deuterated acetone and water.(*2*) NMR spectra were recorded on Bruker Avance III 300, Avance III 400 or Avance 500 spectrometer with a Prodigy broadband cryoprobe. Spectra were calibrated to the residual solvent signals (C_6D_6: δ_H = 7.16 ppm, δ_C = 128.06 ppm; THF-d_8: δ_H = 3.58 ppm, δ_C = 67.21 ppm). ^{31}P-NMR data was referenced externally to phosphoric acid (δ = 0.0 ppm). The following abbreviations were used for signal multiplicities: s (singlet), d (doublet), t (triplet), p (pentet), m (multiplet), br (broad). LIFDI (Linden CMS) mass spectra were measured by the Zentrale Massenabteilung, Fakultät für Chemie, Georg-August-Universität. Elemental analyses were obtained from the Analytisches Labor, Georg-August-Universität using an Elementar Vario EL 3 analyzer. IR spectra were obtained as KBr pellets or in solution with a Thermo Science Nicolet iZ10. EPR spectra were measured using a Bruker ELEXSYS E500 spectrometer, equipped with the digital temperature control system ER 4131VT using nitrogen as coolant. All spectra at 150 K were recorded at about 9.4 GHz microwave frequency and 4 G field modulation amplitude, 100 kHz field modulation frequency, and around 10 mW microwave power. Photolysis experiments were performed using a 150 W

Hg(Xe) arc lamp with a lamp housing and arc lamp power supply from LOT-QuantumDesign GmbH. IR irradiation was cut off by a water filter and the photolyzed sample was kept at room temperature by cooling with a water bath. If not stated otherwise, a white-glass filter with a cut off wavelength of 305 nm was used. Gas phase analysis was performed by a Shimadzu GC-2014 equipped with a TCD detector and a molecular sieve 5Å, 80/100 column. Kinetic data analysis was performed using the program package COPASI.*(3)*

(a)

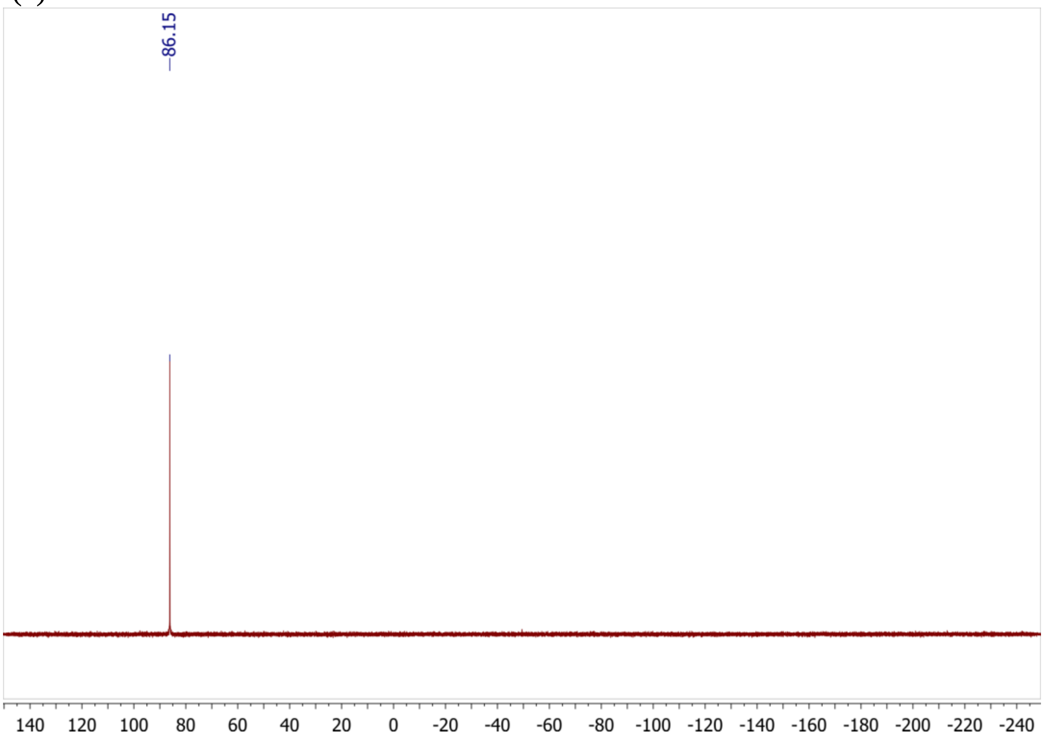

86.15
140 120 100 80 60 40 20 0 -20 -40 -60 -80 -100 -120 -140 -160 -180 -200 -220 -240

(b)

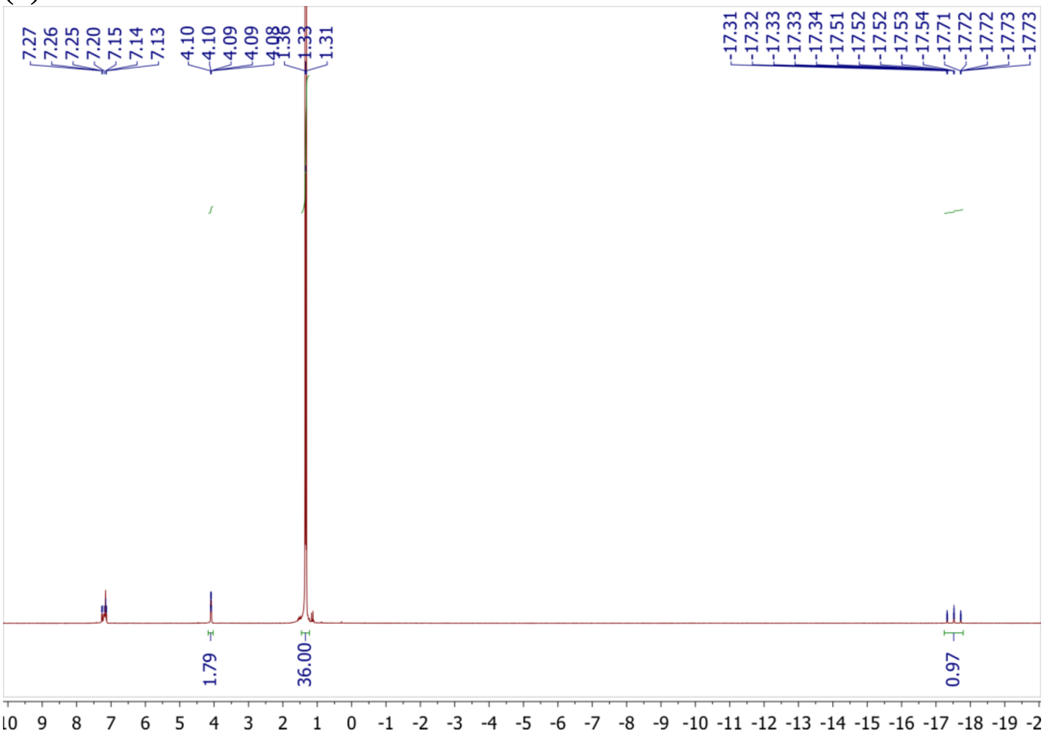

7.27 7.26 7.25 7.20 7.15 7.14 7.13
4.10 4.10 4.09 4.09 4.08 1.36 1.33 1.31
-17.31 -17.32 -17.33 -17.33 -17.34 -17.51 -17.52 -17.52 -17.53 -17.54 -17.71 -17.72 -17.72 -17.73 -17.73
1.79
36.00
0.97
10 9 8 7 6 5 4 3 2 1 0 -1 -2 -3 -4 -5 -6 -7 -8 -9 -10 -11 -12 -13 -14 -15 -16 -17 -18 -19 -2

(c)

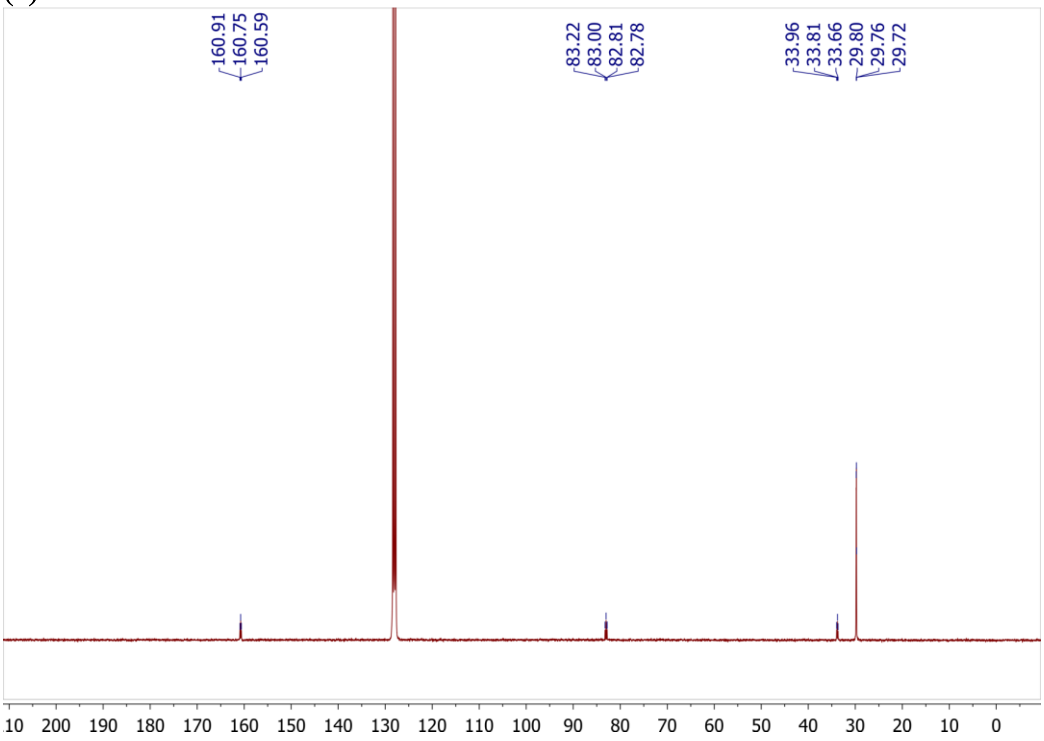

Supplementary Figure 1. (a) $^{31}P\{^1H\}$ (b) ^1H and (c) $^{13}C\{^1H\}$ NMR spectra of **1** in C_6D_6.

(a)

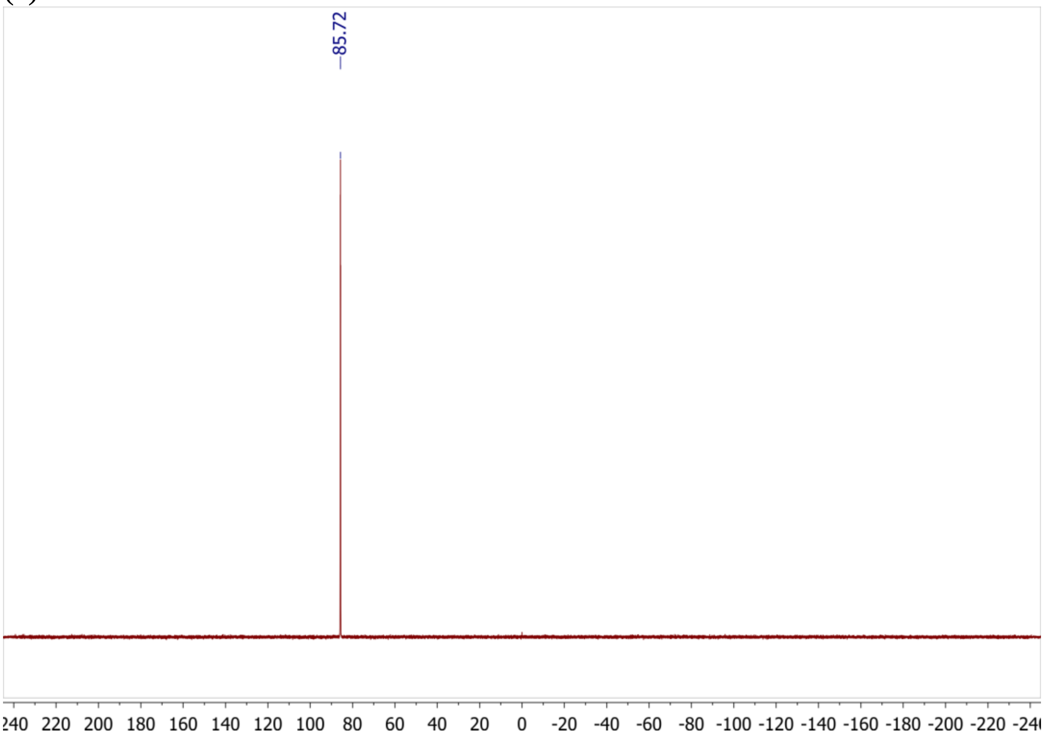

(b)

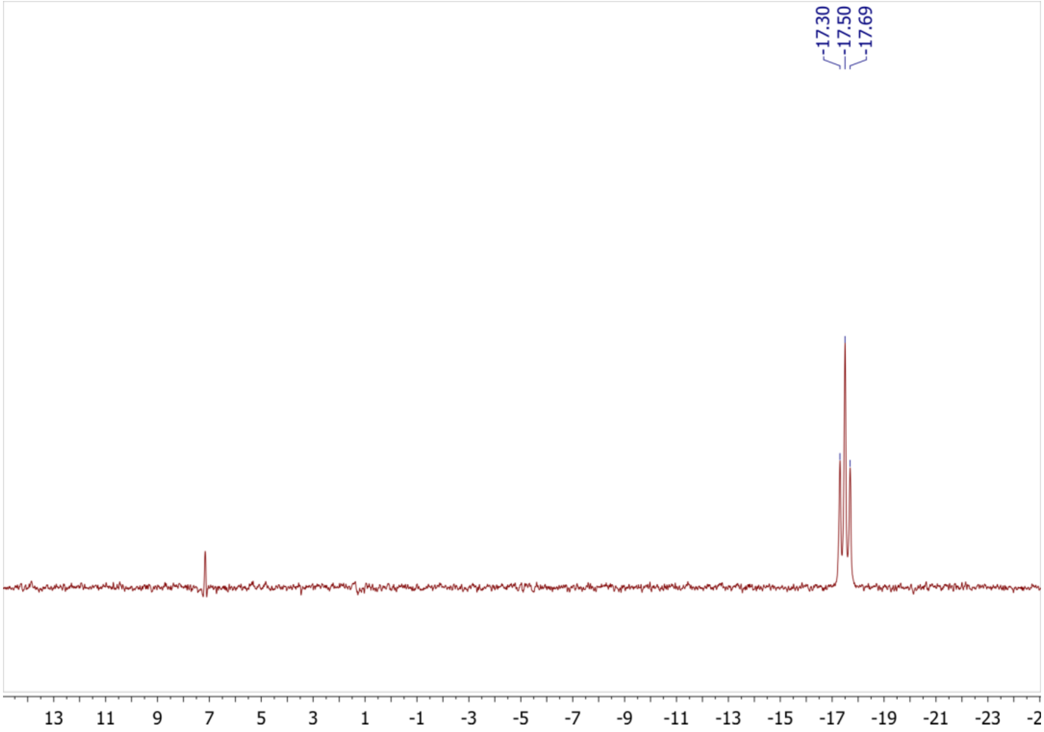

Supplementary Figure 2. (a) $^{31}P\{^{1}H\}$ and (b) ^{2}H NMR spectra of **1-D** in C_6H_6.

(a)

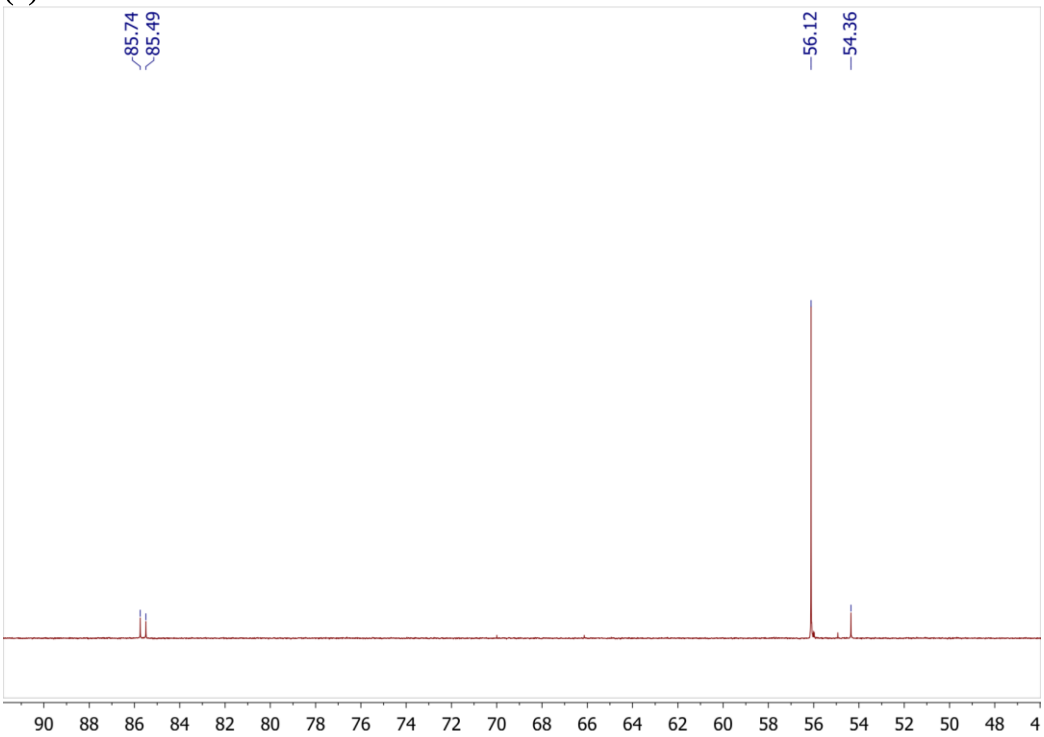

(b)

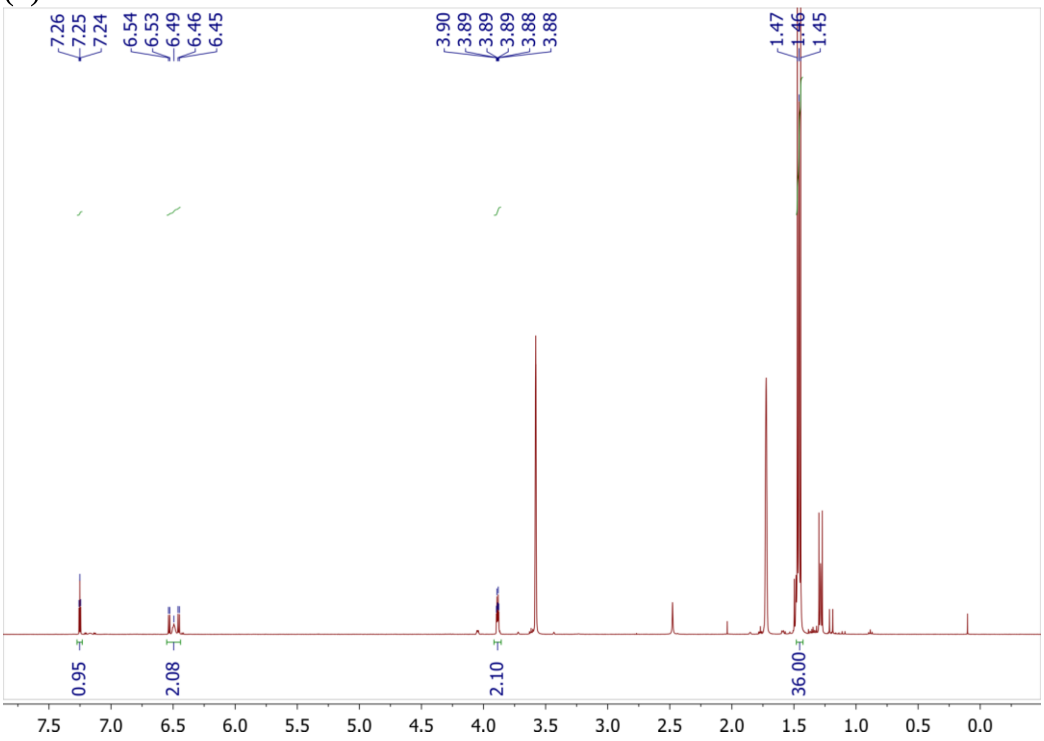

(c)

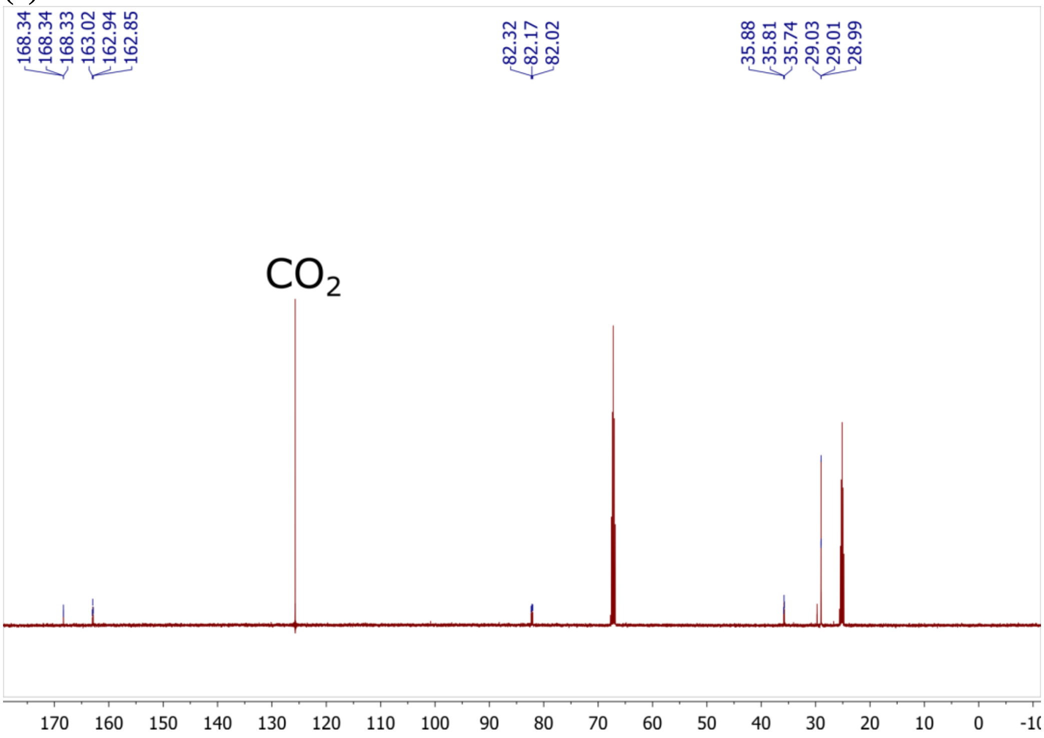

Supplementary Figure 3. (a) $^{31}P\{^1H\}$ (b) ^1H and (c) $^{13}C\{^1H\}$ NMR spectra of the reaction of **1** with 10 atm CO_2 (≥99.5% purity, no further purification) after 14 days in THF-d_8 showing formation of **2**.

(a)

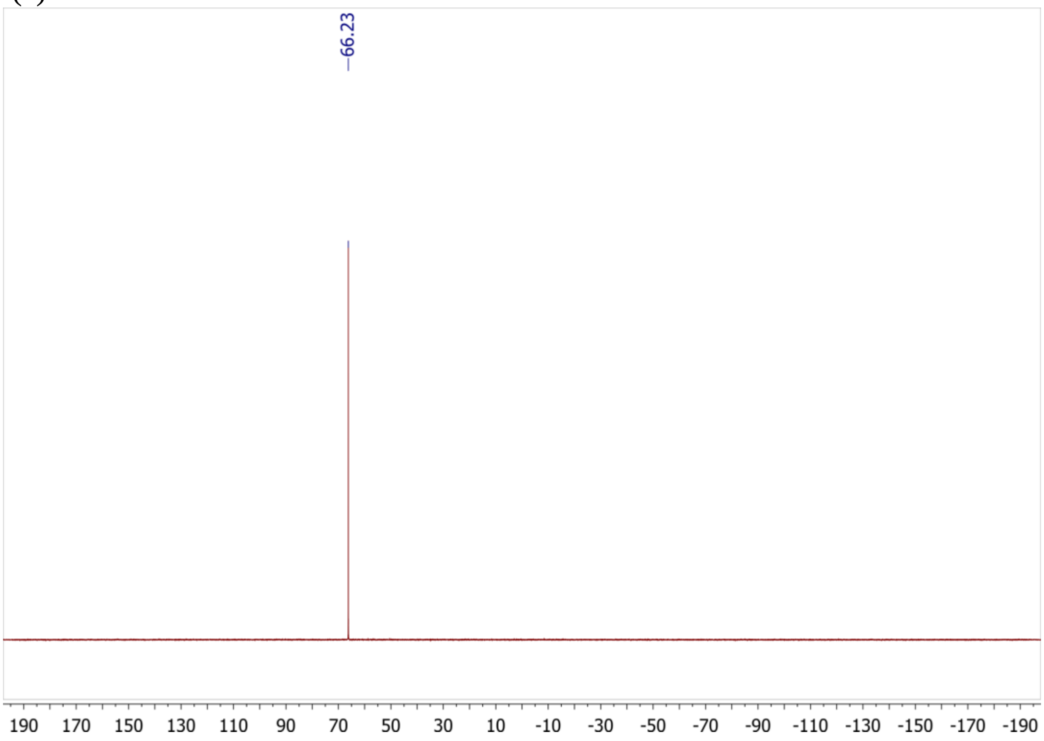
66.23
190 170 150 130 110 90 70 50 30 10 -10 -30 -50 -70 -90 -110 -130 -150 -170 -190

(b)

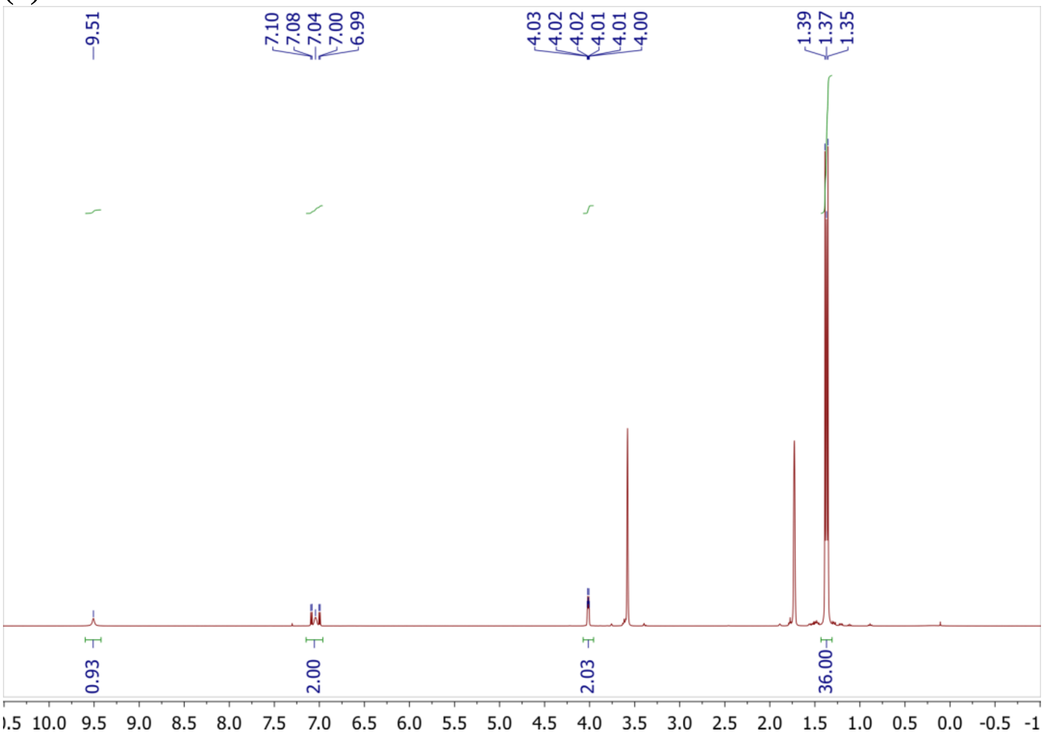
9.51
7.10 7.08 7.04 7.00 6.99
4.03 4.02 4.02 4.01 4.01 4.00
1.39 1.37 1.35
0.93
2.00
2.03
36.00
10.0 9.5 9.0 8.5 8.0 7.5 7.0 6.5 6.0 5.5 5.0 4.5 4.0 3.5 3.0 2.5 2.0 1.5 1.0 0.5 0.0 -0.5

(c)

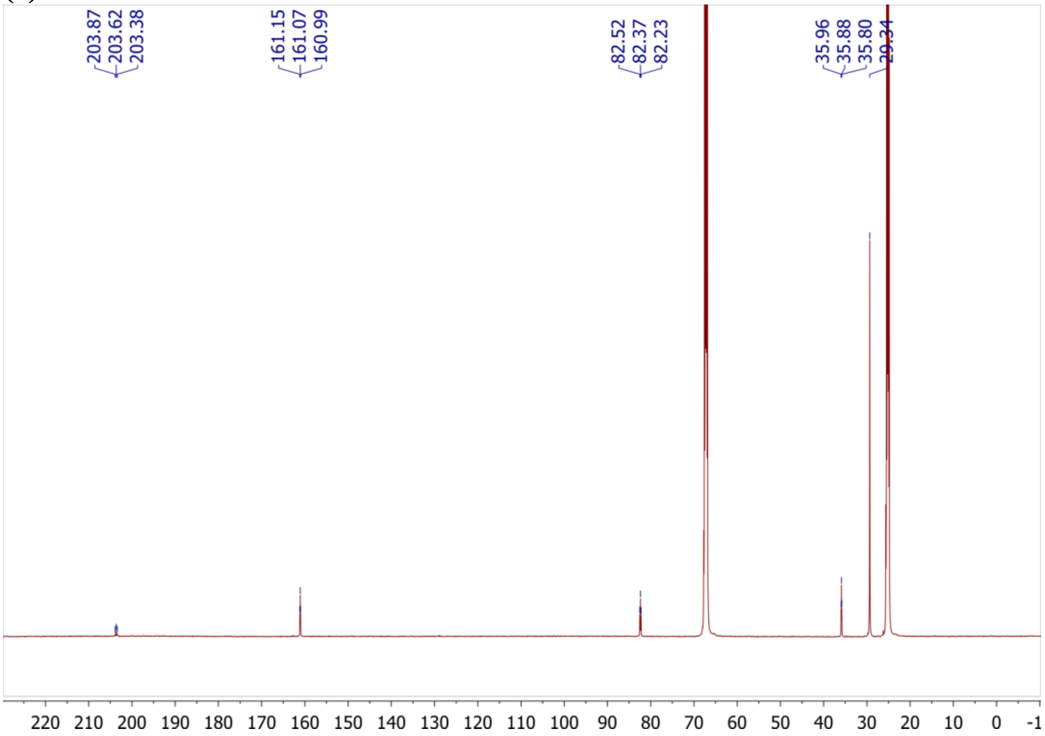

Supplementary Figure 4. (a) $^{31}P\{^{1}H\}$ (b) ^{1}H and (c) $^{13}C\{^{1}H\}$ NMR spectra of **3** in THF-d_8.

(a)

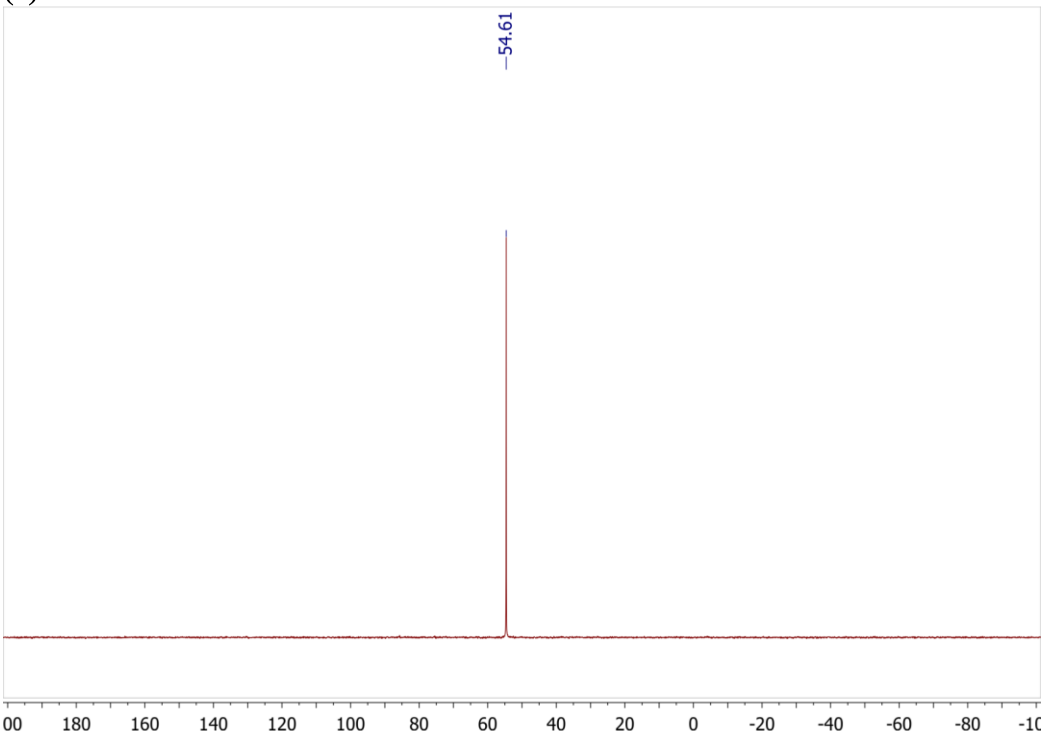

(b)

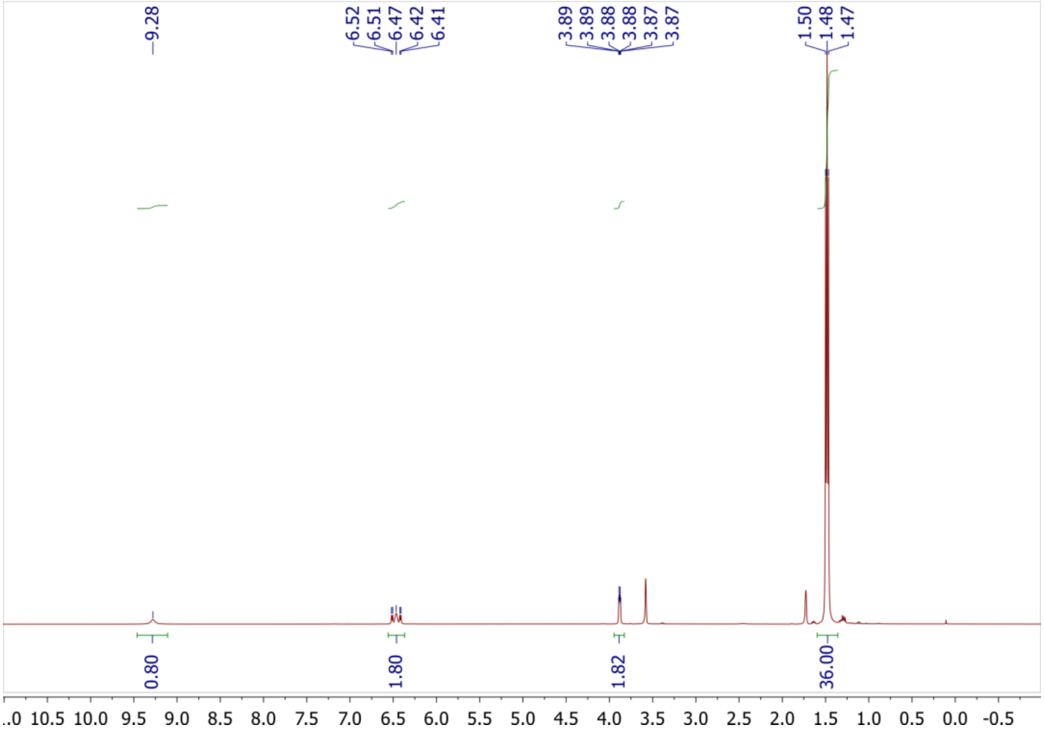

(c)

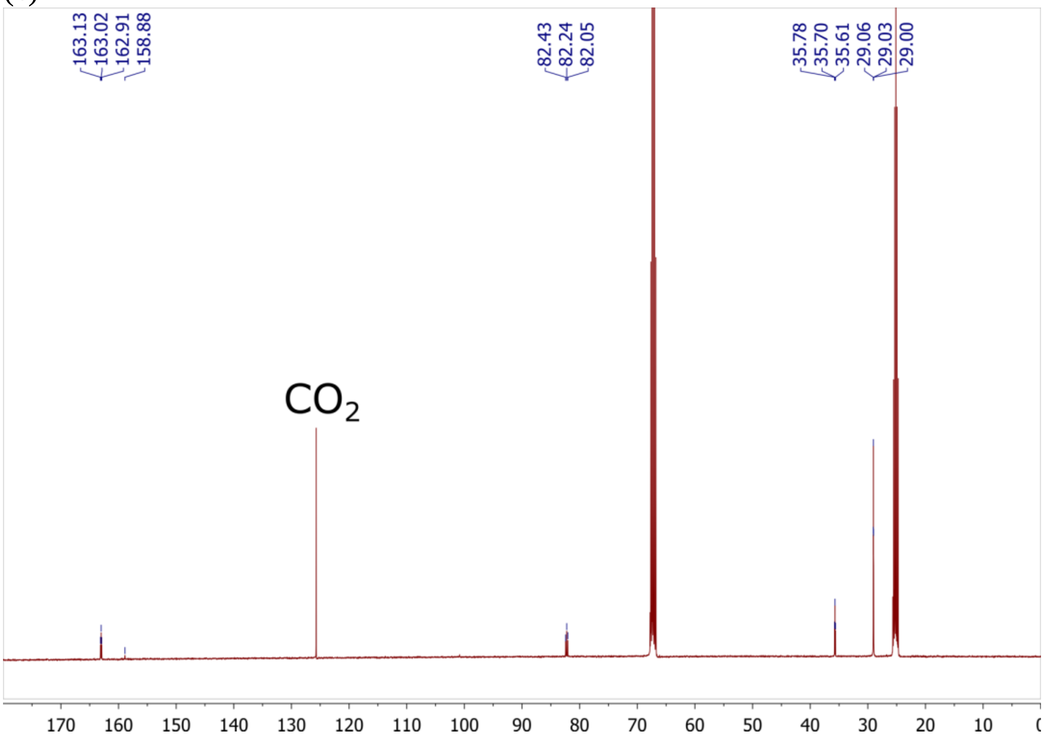

Supplementary Figure 5. (a) $^{31}P\{^{1}H\}$ NMR (b) ^{1}H NMR and (c) $^{13}C\{^{1}H\}$ NMR spectra of **4** in THF-d_8.

(a)

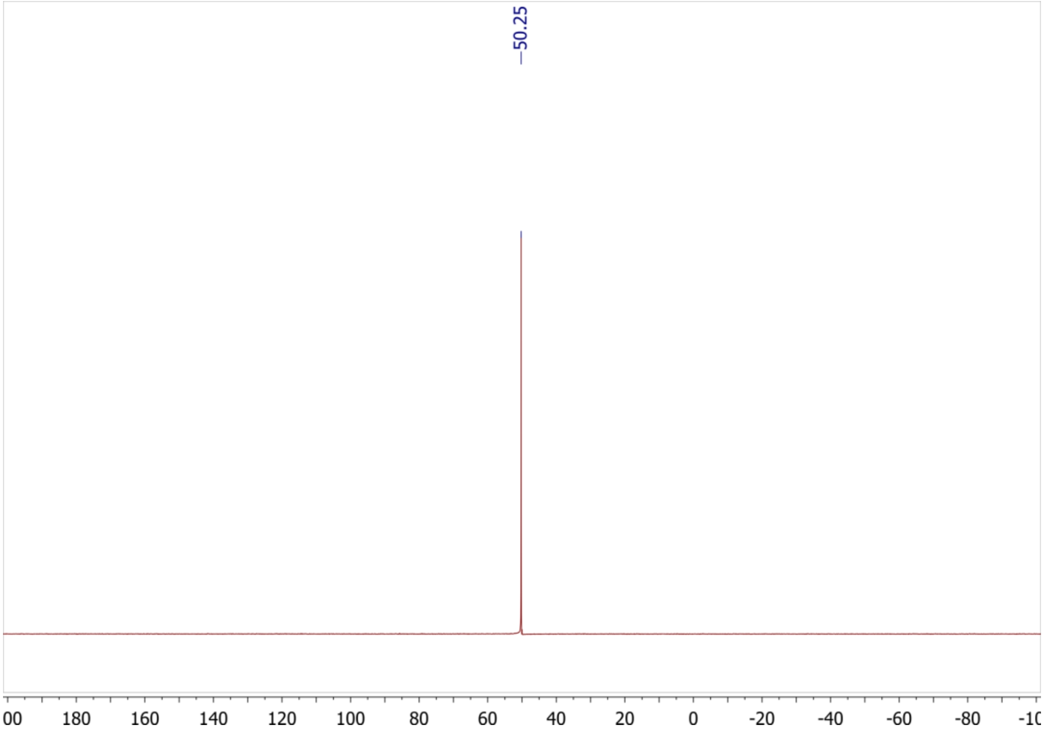
50.25
00
180
160
140
120
100
80
60
40
20
0
-20
-40
-60
-80
-10

(b)

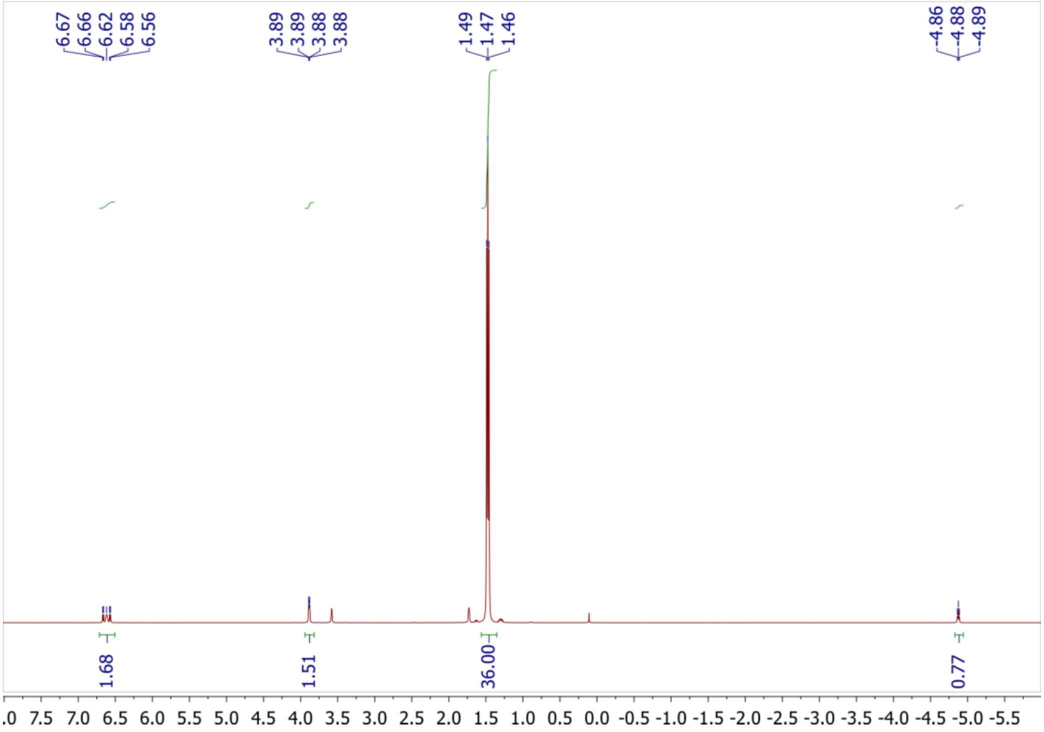
6.67
6.66
6.62
6.58
6.56
3.89
3.89
3.88
3.88
1.49
1.47
1.46
-4.86
-4.88
-4.89
1.68
1.51
36.00
0.77
.0 7.5 7.0 6.5 6.0 5.5 5.0 4.5 4.0 3.5 3.0 2.5 2.0 1.5 1.0 0.5 0.0 -0.5 -1.0 -1.5 -2.0 -2.5 -3.0 -3.5 -4.0 -4.5 -5.0 -5.5

(c)

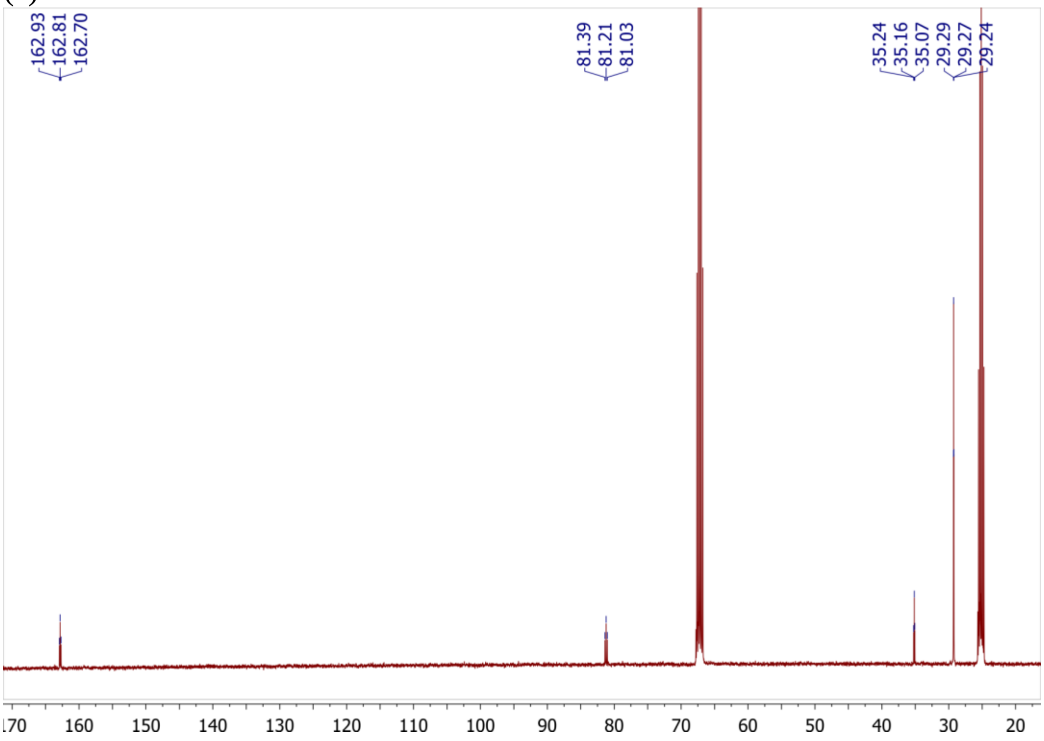

Supplementary Figure 6. (a) $^{31}P\{^1H\}$ (b) ^1H and (c) $^{13}C\{^1H\}$ NMR spectra of **6** in THF-d_8.

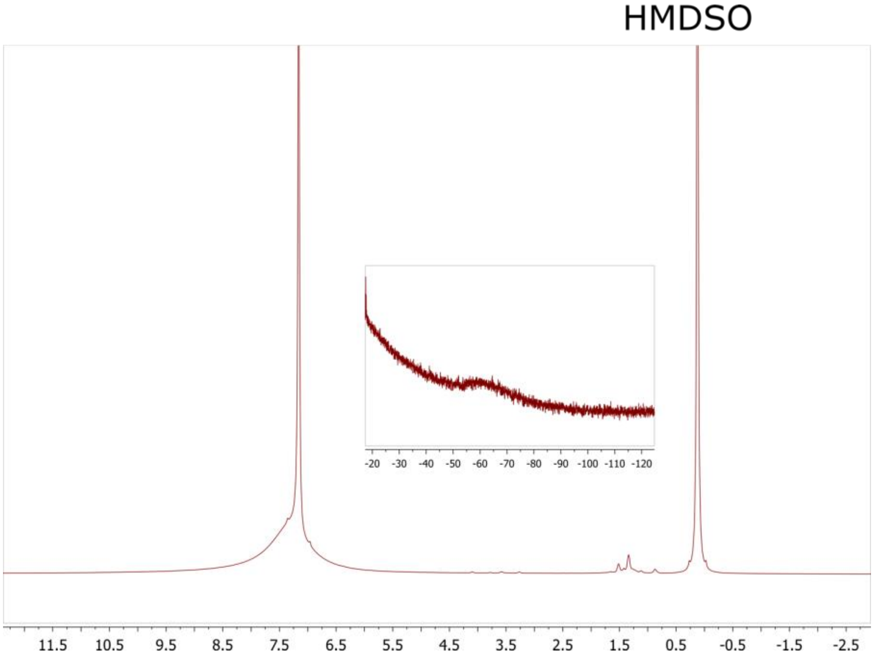

Supplementary Figure 7. ^1H NMR spectrum of **7** in C_6D_6.

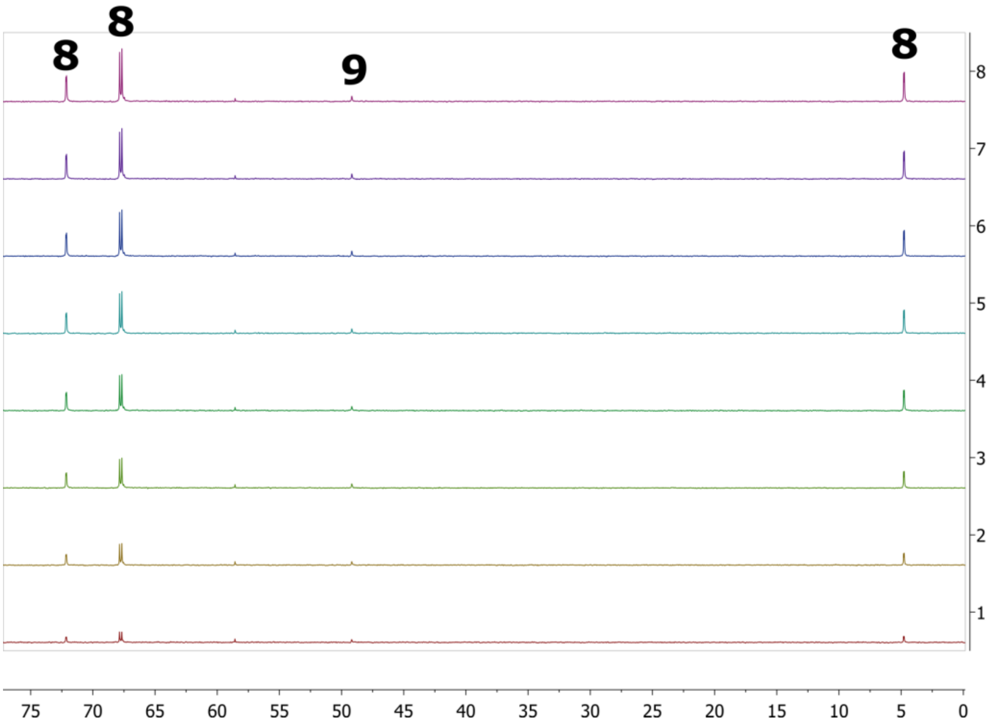

Supplementary Figure 8. $^{31}P\{^{1}H\}$ NMR spectra of the reaction of **7** with 1 eq $^{13}CO_2$ in C_6D_6 monitored over 15 h showing a constant ratio of **8** and **9** with increasing consumption of **7**.

(a)

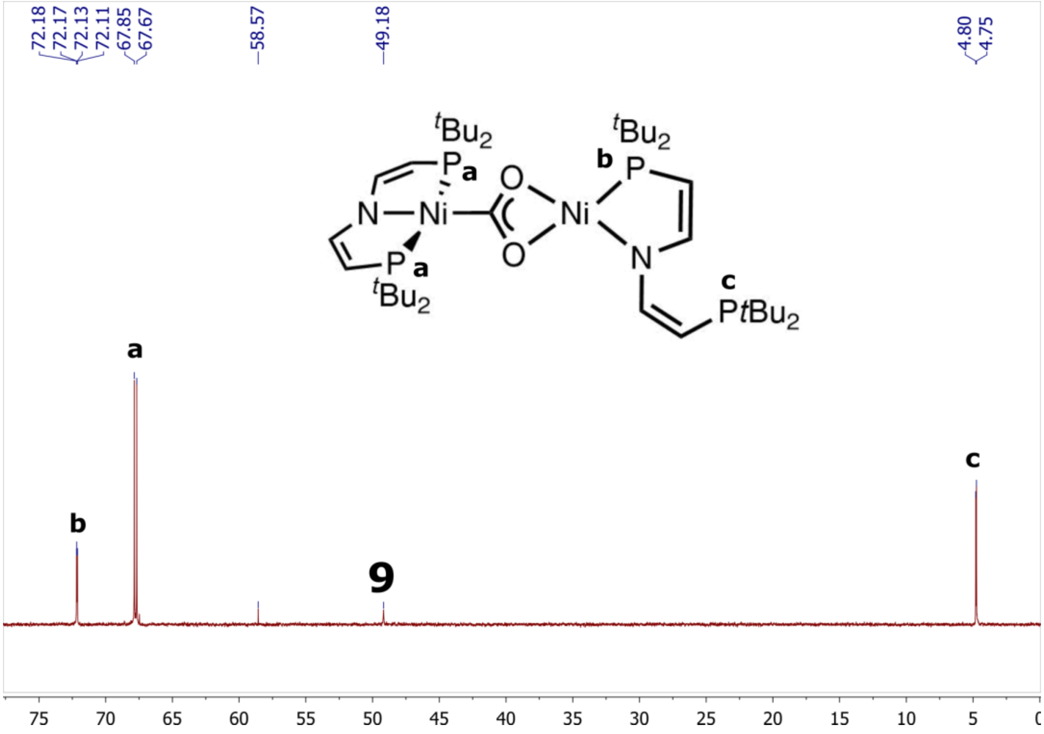
72.18
72.17
72.13
72.11
67.85
67.67
58.57
49.18
4.80
4.75
a
b
c
9
75 70 65 60 55 50 45 40 35 30 25 20 15 10 5

(b)

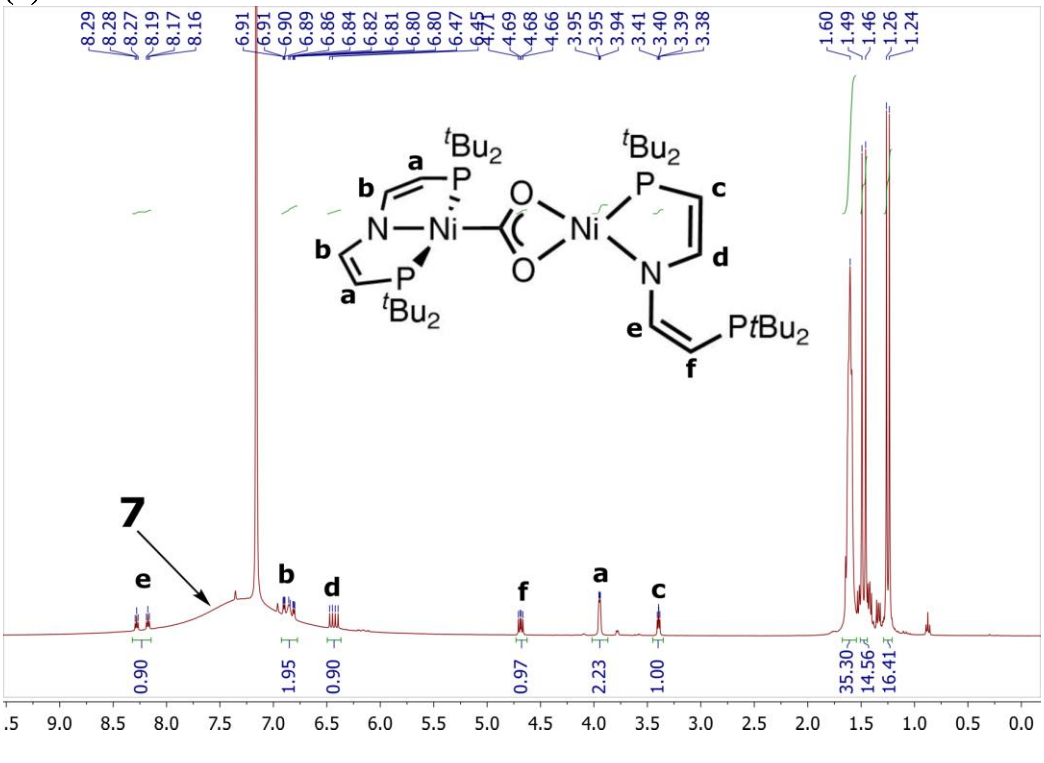
7
e
b
d
f
a
c
0.90
1.95
0.90
0.97
2.23
1.00
35.30
14.56
16.41
9.0 8.5 8.0 7.5 7.0 6.5 6.0 5.5 5.0 4.5 4.0 3.5 3.0 2.5 2.0 1.5 1.0 0.5 0.0

(c)

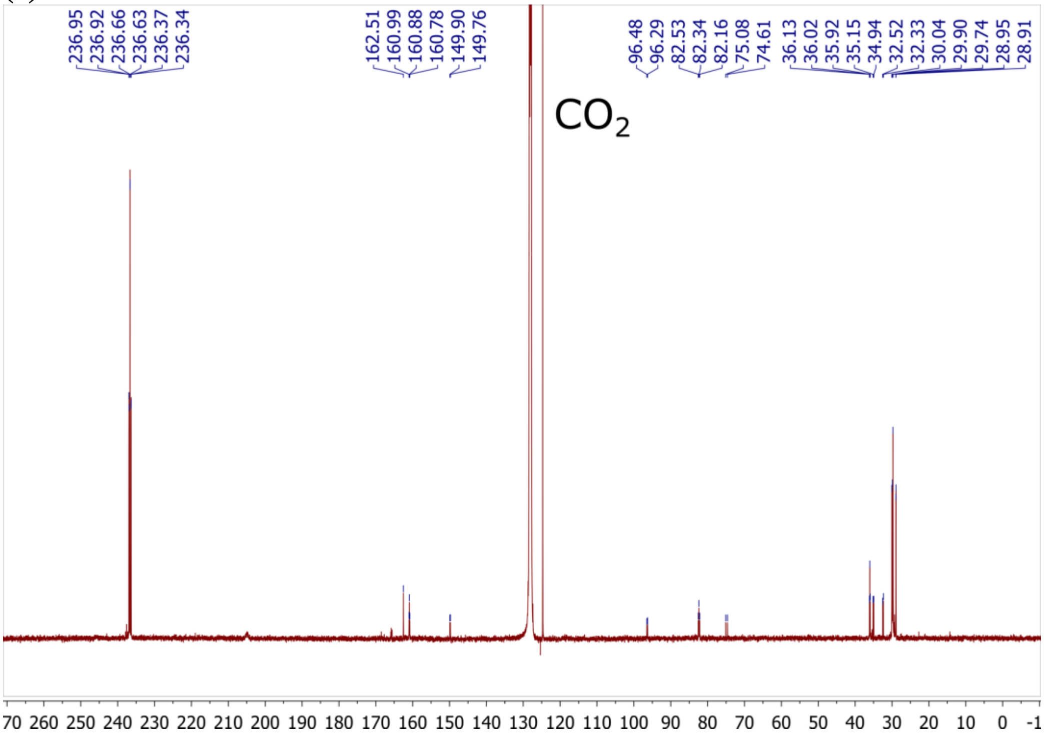

Supplementary Figure 9. (a) $^{31}P\{^1H\}$ (b) ^1H and (c) $^{13}C\{^1H\}$ NMR spectra of the reaction of **7** with 1 eq $^{13}CO_2$ in C_6D_6 after 16h.

(a)

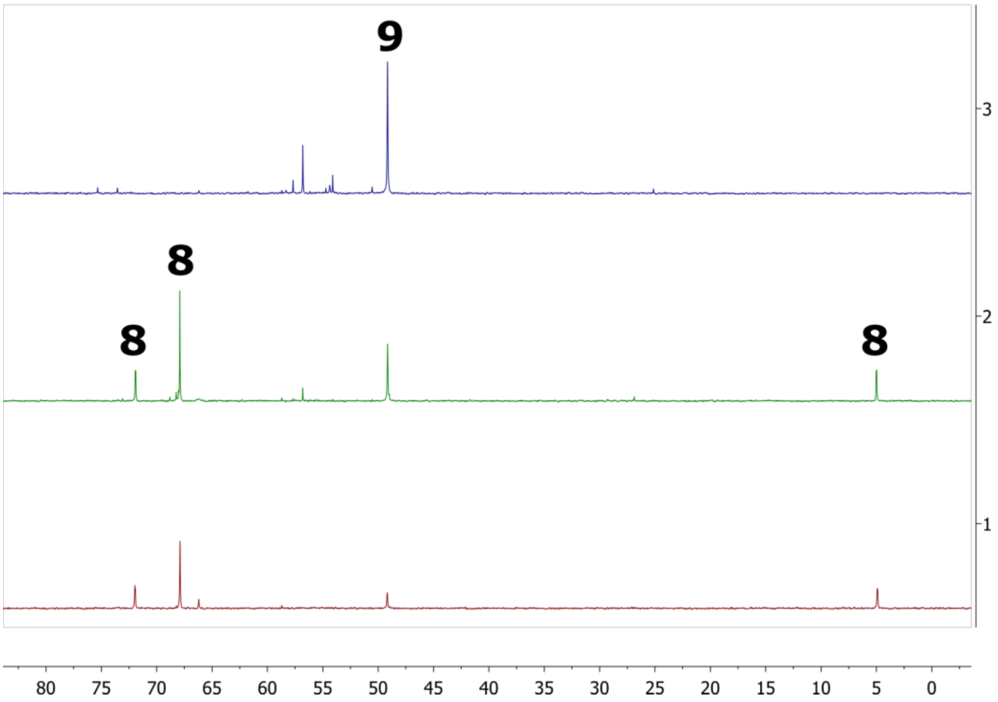

(b)

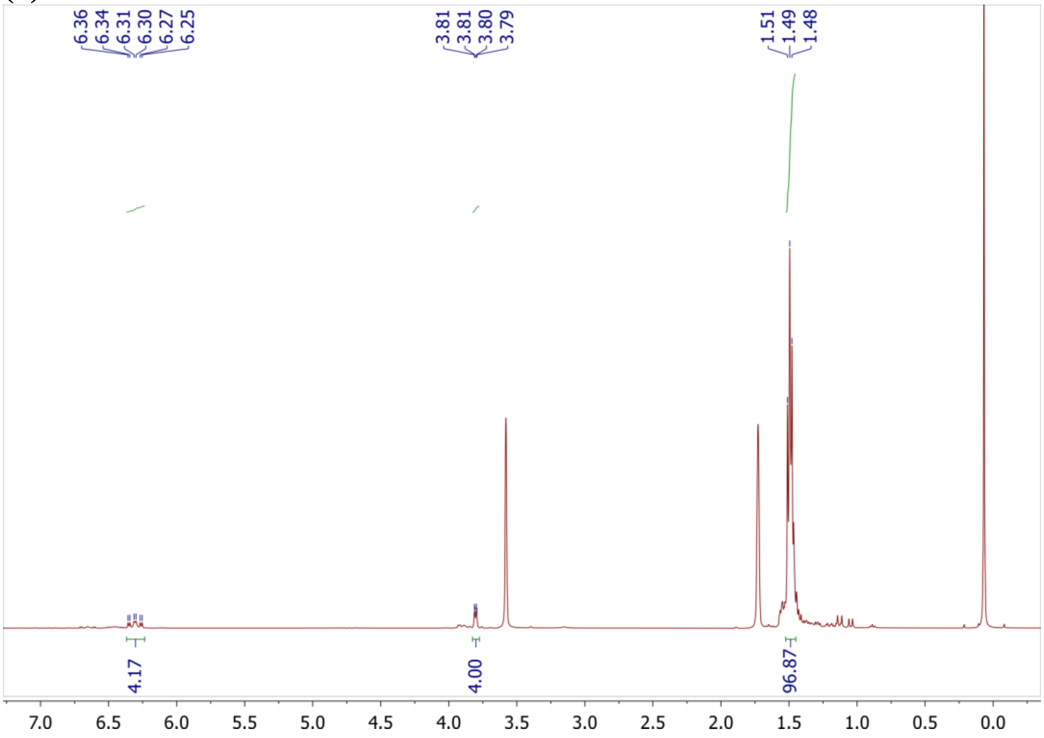

Supplementary Figure 10. (a) $^{31}P\{^1H\}$ NMR spectra of the reaction of **7** with 1 atm CO_2 in THF-d_8 after 1.5 h (bottom), 25 h (middle) and 11 d (top) showing conversion of **8** to **9**. (b) ^1H NMR spectrum of the reaction of **7** with 1 atm CO_2 in THF-d_8 after 11 days.

(a)

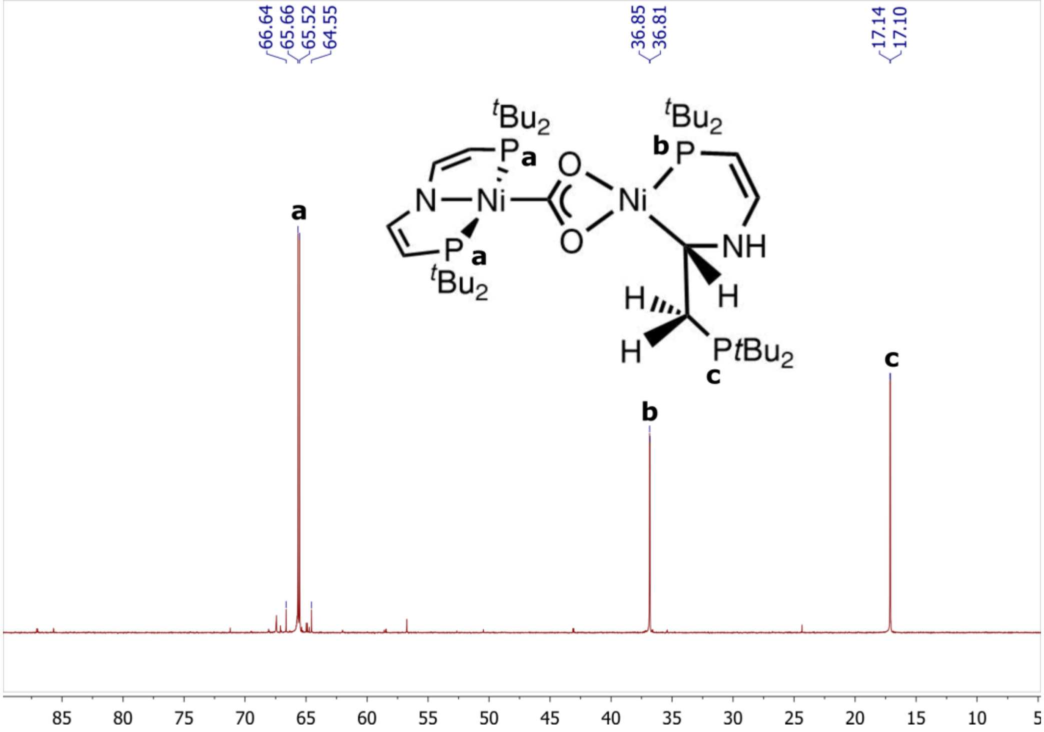

(b)

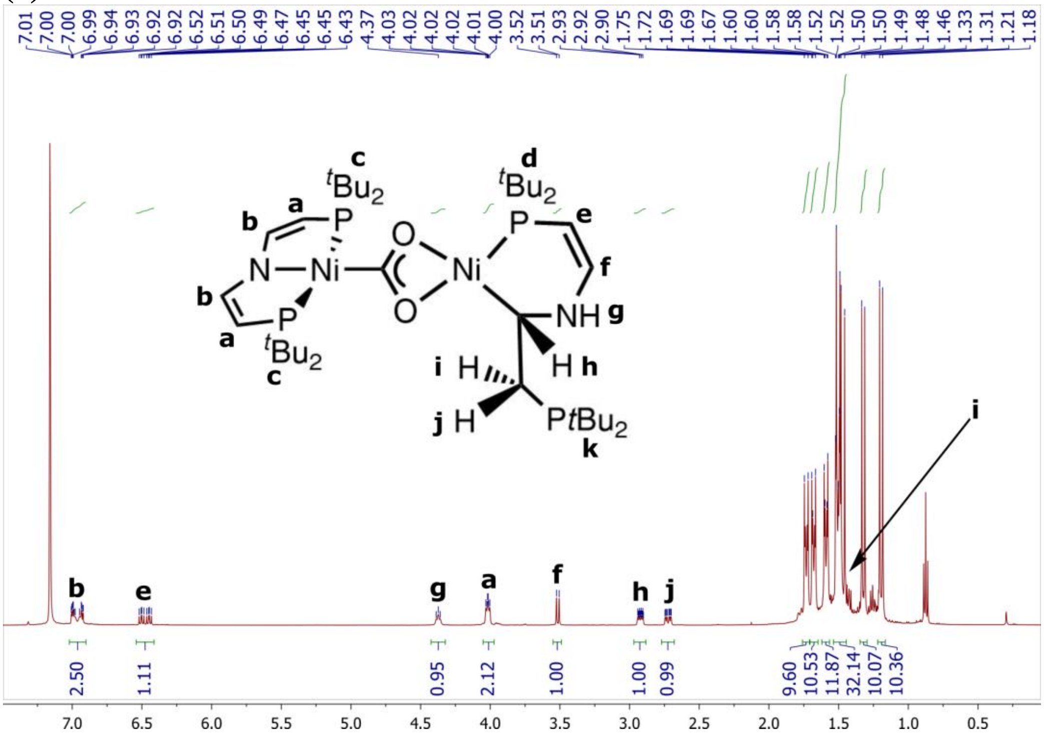
7.01 7.00 7.00 6.99 6.94 6.93 6.92 6.92 6.52 6.51 6.50 6.49 6.47 6.45 6.45 6.43 4.37 4.03 4.02 4.02 4.02 4.01 4.00 3.52 3.51 2.93 2.92 2.90 1.75 1.72 1.69 1.67 1.60 1.60 1.58 1.58 1.52 1.52 1.50 1.50 1.49 1.48 1.46 1.33 1.31 1.21 1.18
b
e
g
a
f
h
j
i
2.50
1.11
0.95
2.12
1.00
1.00
0.99
9.60
10.53
11.87
32.14
10.07
10.36
7.0 6.5 6.0 5.5 5.0 4.5 4.0 3.5 3.0 2.5 2.0 1.5 1.0 0.5

(c)

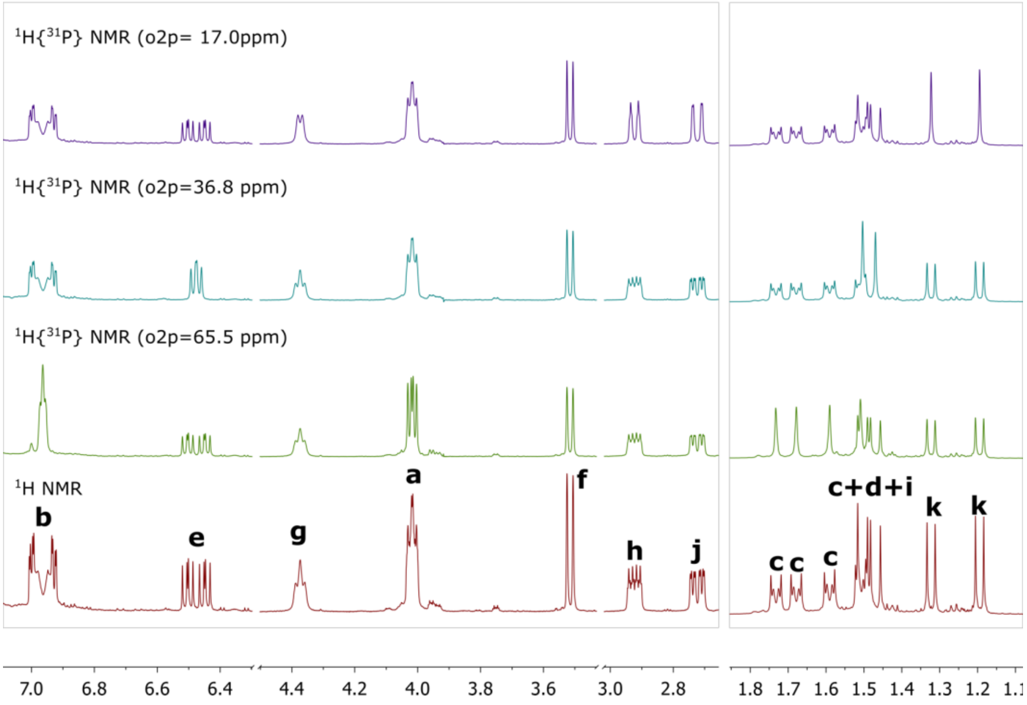
¹H{³¹P} NMR (o2p= 17.0ppm)
¹H{³¹P} NMR (o2p=36.8 ppm)
¹H{³¹P} NMR (o2p=65.5 ppm)
¹H NMR
b
e
g
a
f
h
j
c
c
c
c+d+i
k
k
7.0 6.8 6.6 6.4 4.4 4.2 4.0 3.8 3.6 3.0 2.8
1.8 1.7 1.6 1.5 1.4 1.3 1.2 1.1

(d)

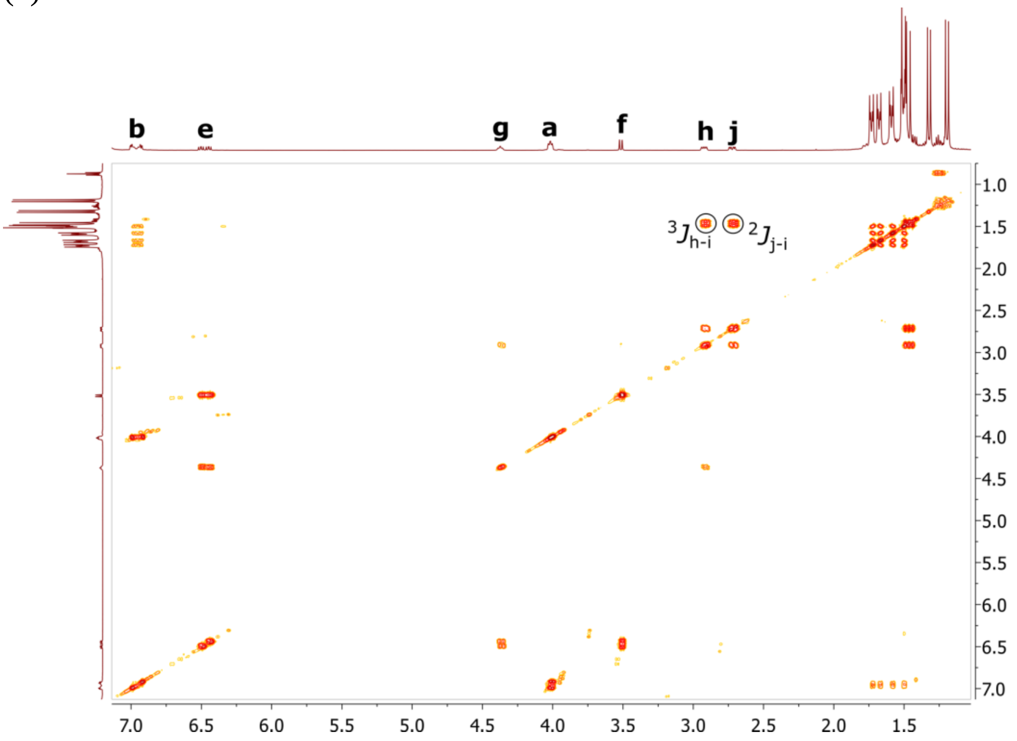

b
e
g
a
f
h
j
$^3J_{h-i}$
$^2J_{j-i}$
1.0
1.5
2.0
2.5
3.0
3.5
4.0
4.5
5.0
5.5
6.0
6.5
7.0
7.0
6.5
6.0
5.5
5.0
4.5
4.0
3.5
3.0
2.5
2.0
1.5

(e)

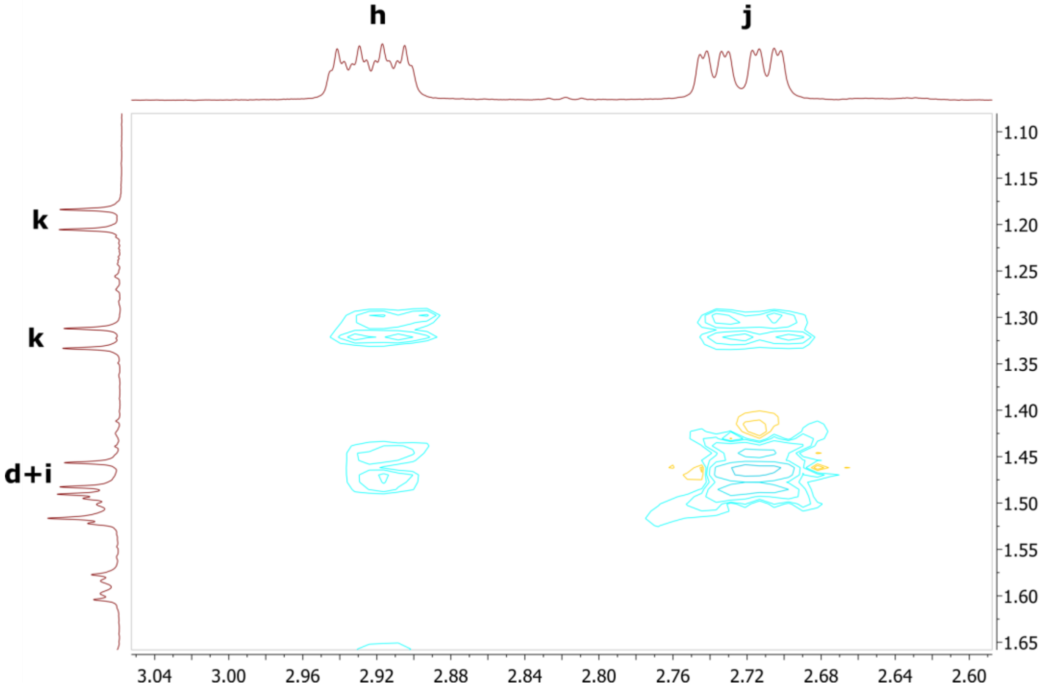

h
j
k
k
d+i
1.10
1.15
1.20
1.25
1.30
1.35
1.40
1.45
1.50
1.55
1.60
1.65
3.04
3.00
2.96
2.92
2.88
2.84
2.80
2.76
2.72
2.68
2.64
2.60

(f)

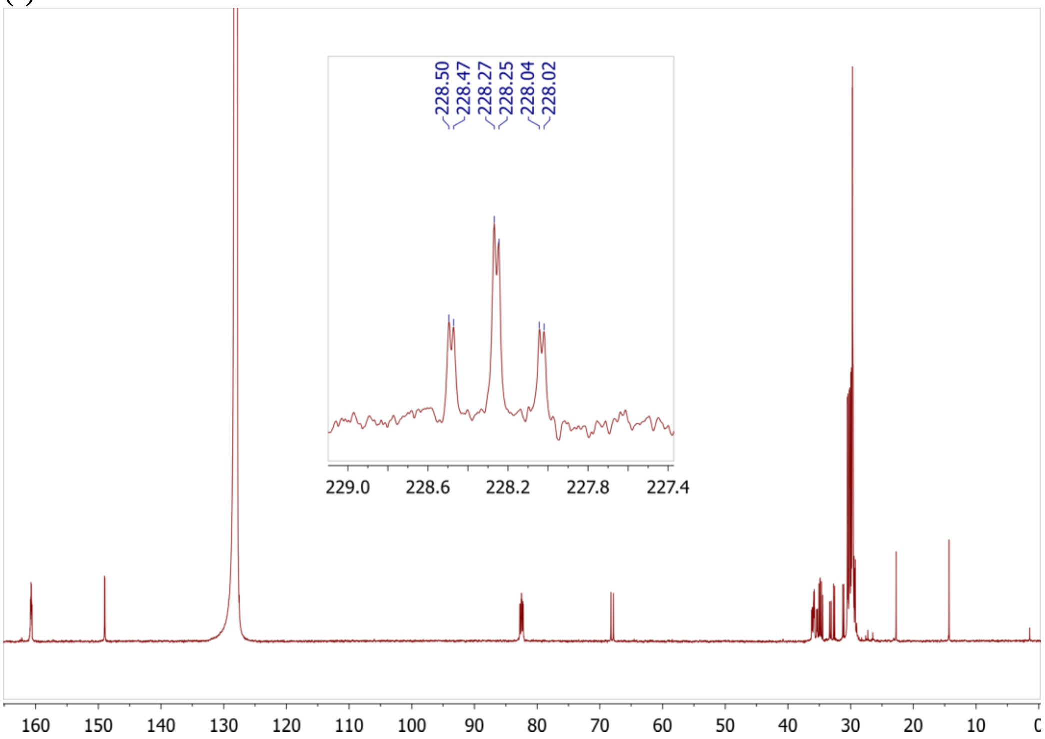

(g)

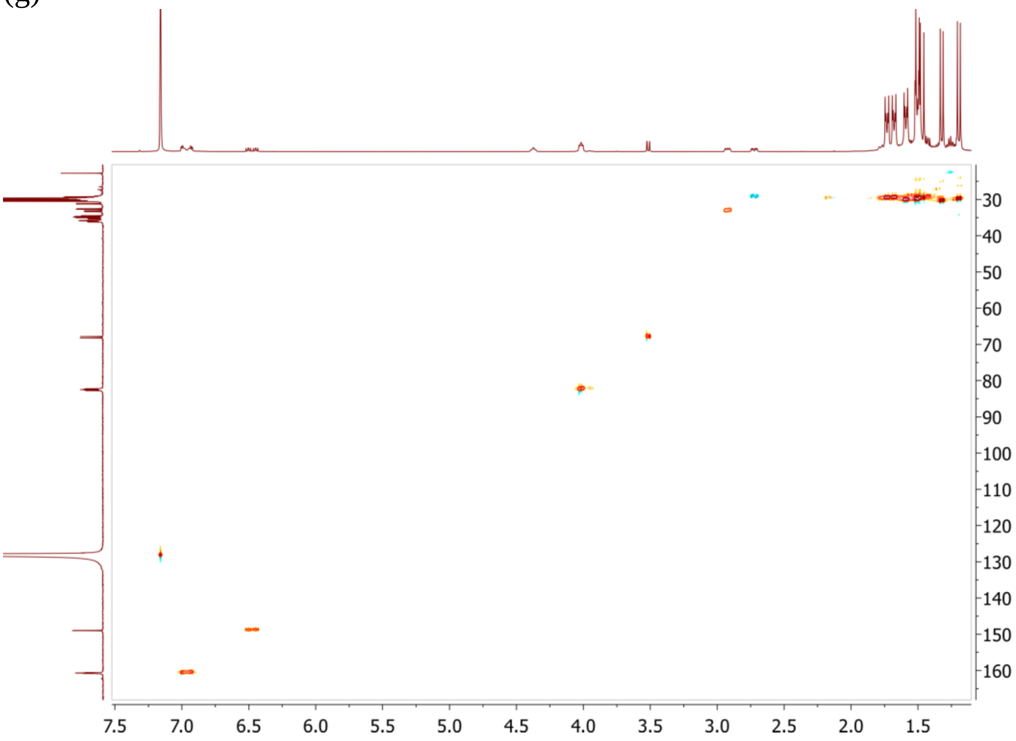

Supplementary Figure 11. (a) $^{31}P\{^{1}H\}$ (b) ^{1}H (c) $^{1}H\{^{31}P\}$ (d) $^{1}H,^{1}H$ COSY (e) $^{1}H,^{1}H$ NOESY (f) $^{13}C\{^{1}H\}$ and (g) $^{1}H,^{13}C$ HSQC NMR spectra of **10** in C_6D_6.

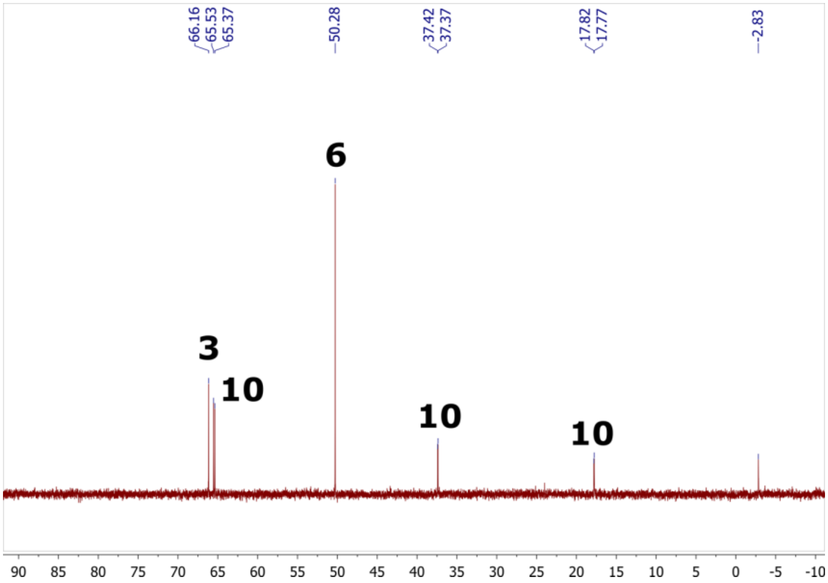

Supplementary Figure 12. $^{31}P\{^{1}H\}$ NMR spectrum of the reaction of **1** with **3** after 7 h of photolysis ($\lambda_{exc.}$ > 305 nm).

(a)

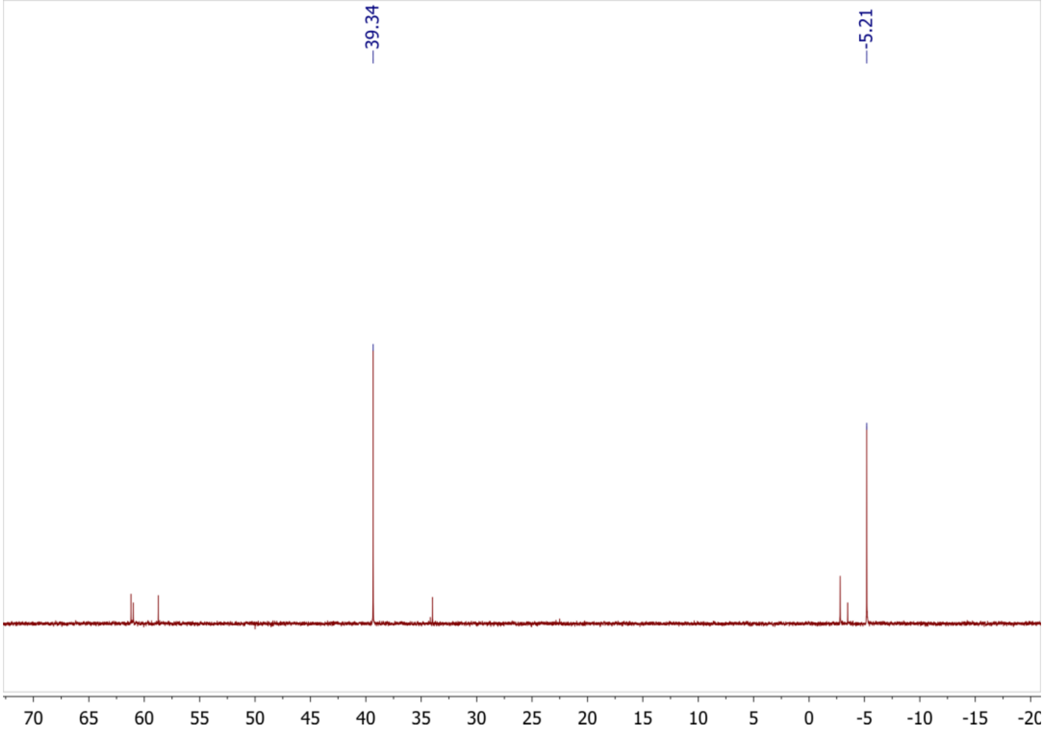

(b)

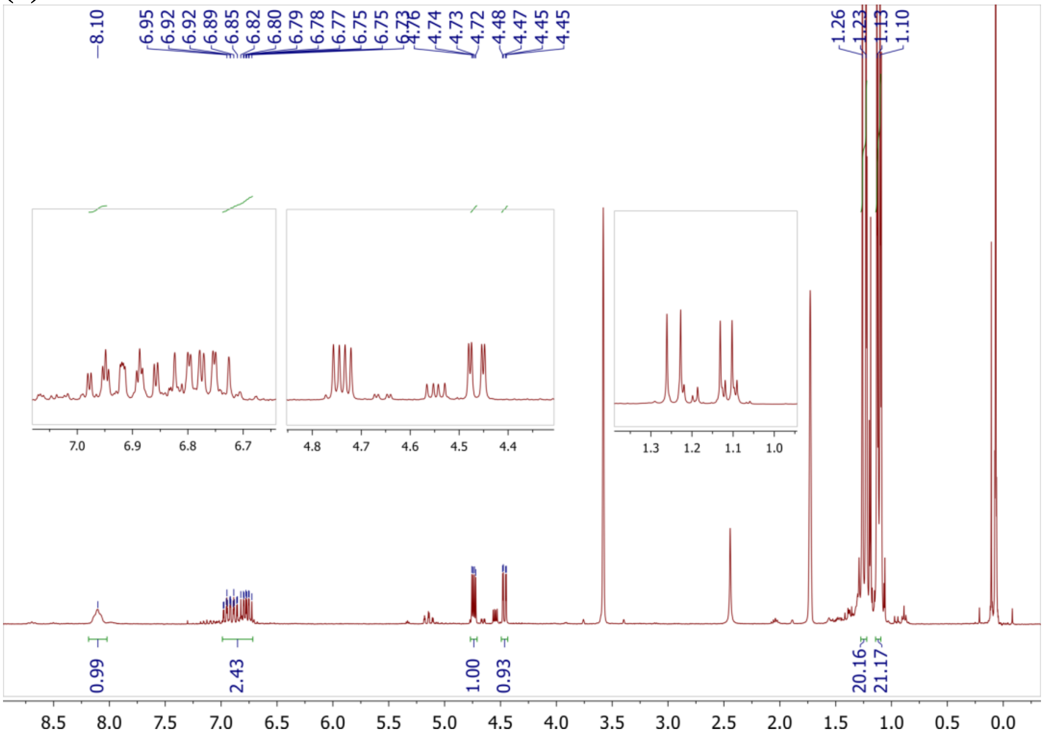

(c)

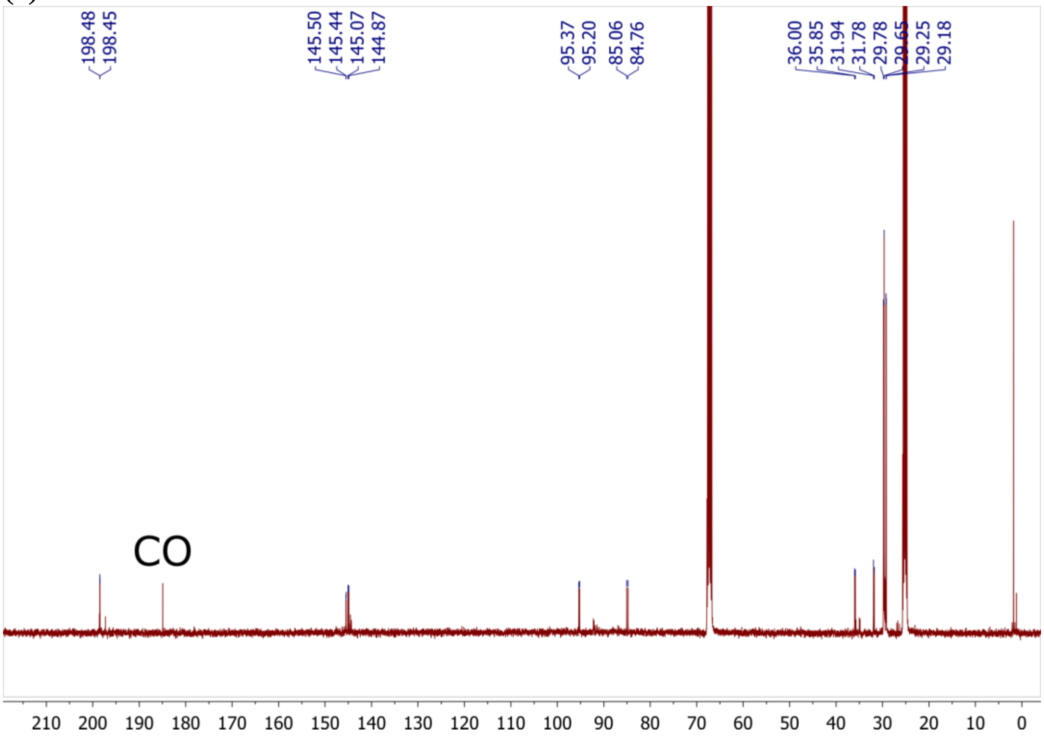

Supplementary Figure 13. (a) $^{31}P\{^{1}H\}$ (b) ^{1}H NMR and (c) $^{13}C\{^{1}H\}$ NMR spectra of **11** in THF-d_8.

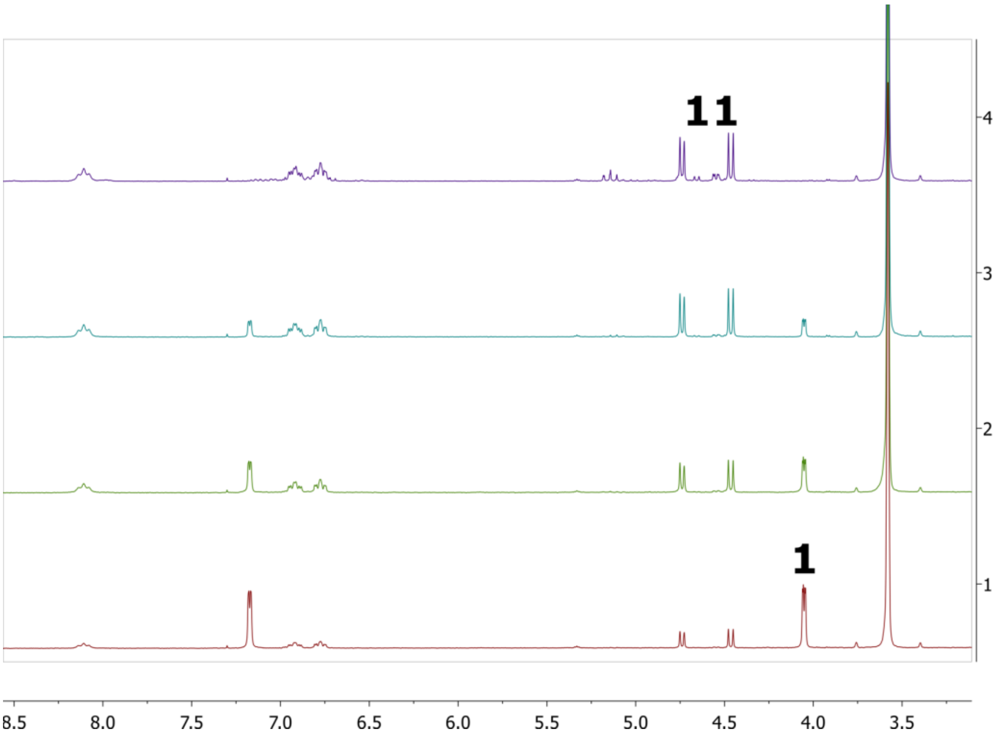

Supplementary Figure 14. $^{1}H\{^{31}P\}$ NMR spectra of the reaction of **1** with 1 atm CO in THF-d_8 after 10 min (bottom), 20 min (lower middle), 30 min (upper middle) and 90 min (top) of photolysis ($\lambda_{exc.}$ > 305 nm) showing conversion of **1** to **10**.

Wavelength dependence of photochemical CO_2 activation by **1**:

3.3 mg (0.0079 mmol, 1.00 eq) **1** and 2 µL HMDSO are dissolved in 0.5 mL THF-d_8 and filled into a J-Young NMR tube. The solution is degassed by three pump-freeze-thaw cycles, 1 atm CO_2 pressure (≥99.5% purity, no further purification) is applied and the sample is photolyzed using a white-glass ($\lambda_{exc.}$ > 305 nm) or green-glass filter (>420 nm).

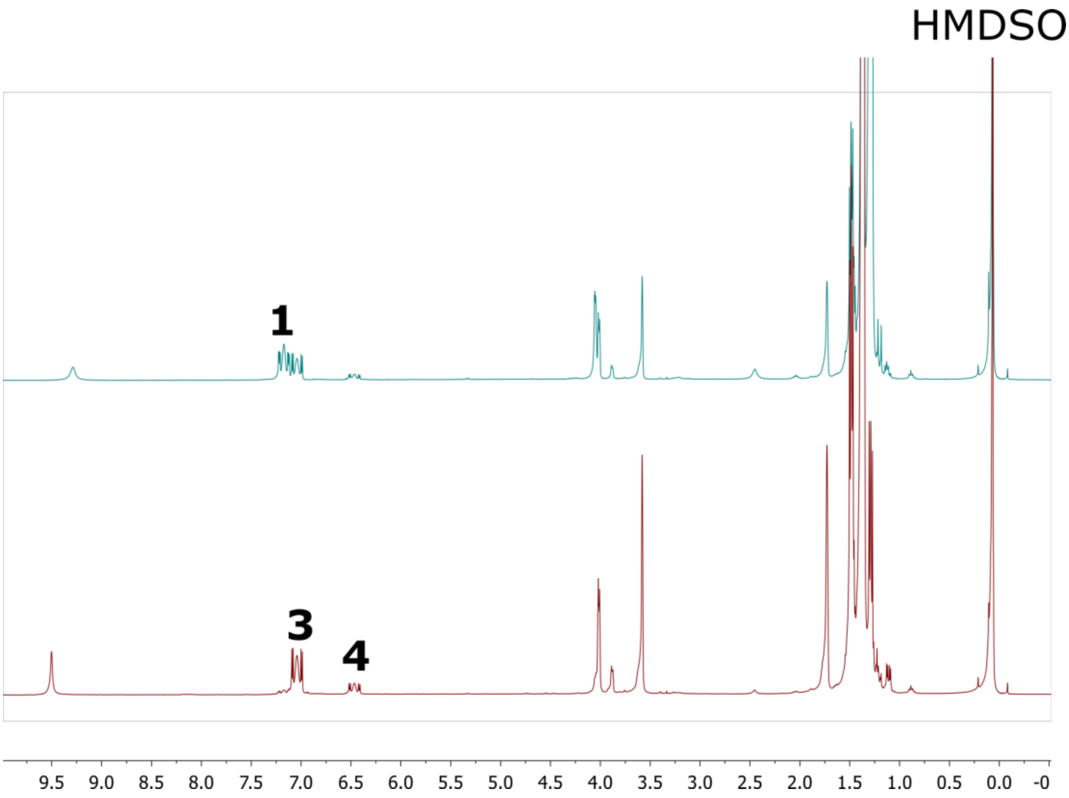

Supplementary Figure 15. ^{1}H NMR spectrum of the reaction of **1** with 1 atm CO_2 (≥99.5% purity, no further purification) in THF-d_8 showing different ratios of **1**, **3** and **4** after 4 h of photolysis (bottom: $\lambda_{exc.}$ > 305 nm; top: $\lambda_{exc.}$ > 420 nm).

Photochemical decarbonylation of **3**:

(a)

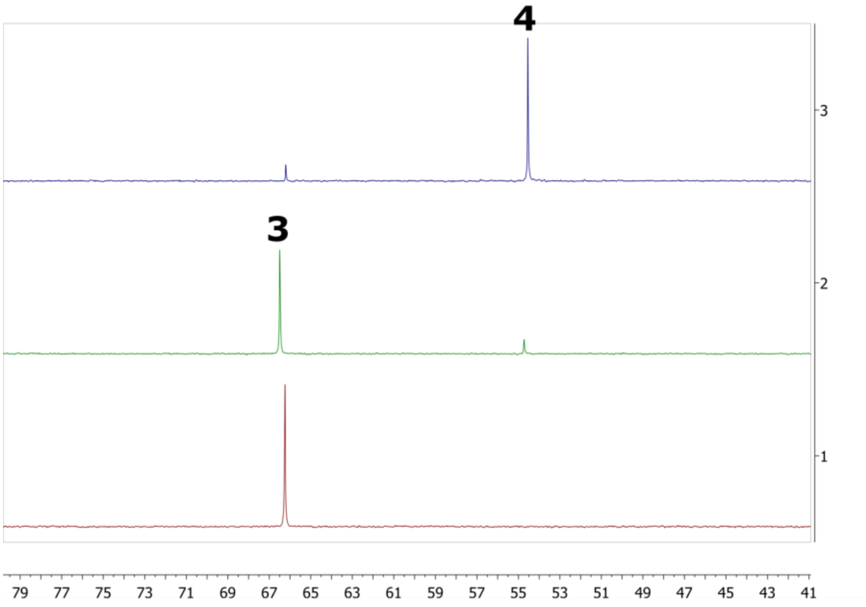

(b)

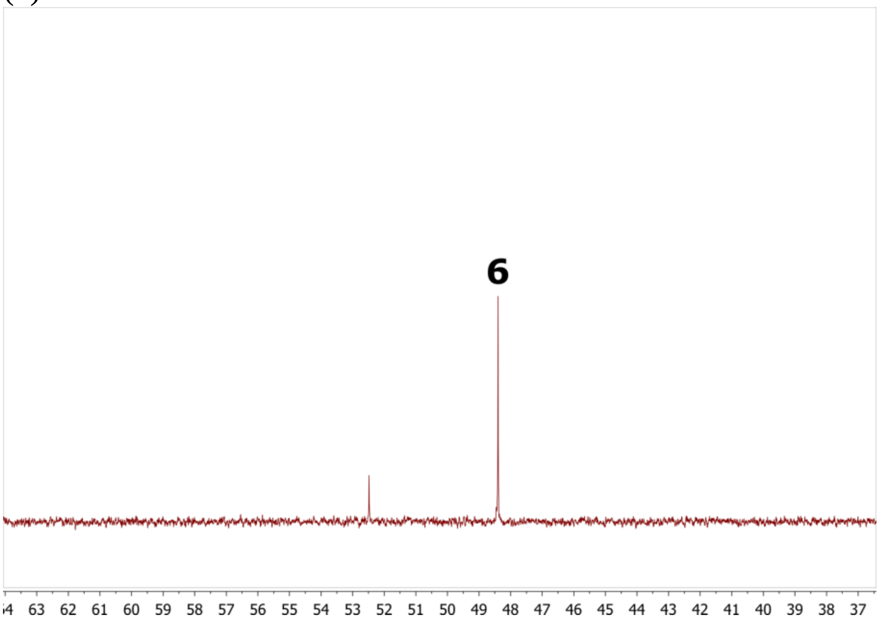

Supplementary Figure 16. (a) $^{31}P\{^{1}H\}$ NMR spectrum of the reaction of **3** with CO_2 (≥99.9993% purity, purification by passing through P_4O_{10}, Drierite and cooling to -40°C) in THF-d_8 showing different ratios of **3** and **4**. Bottom: **3** under Argon, middle: **3** after 18 h under 1 atm CO_2, top: **3** after 18 h of photolysis ($\lambda_{exc.}$ > 305 nm) under 1 atm CO_2. (b) $^{31}P\{^{1}H\}$ NMR spectrum of **3** after 3.5 h of photolysis ($\lambda_{exc.}$ > 305 nm) under Ar in THF-d_8 showing formation of **6**.

Photolysis of **1** under Ar:

4.5 mg (0.011 mmol, 1.00 eq) **1** are dissolved in 0.5 mL C_6D_6 and 3 µL HMDSO are added as internal standard. The sample is filled in a J-Young NMR tube and the sample is photolyzed ($\lambda_{exc.}$ > 305 nm). ^{1}H NMR spectroscopy is measured repetitively to determine the conversion of **1**.

(a)

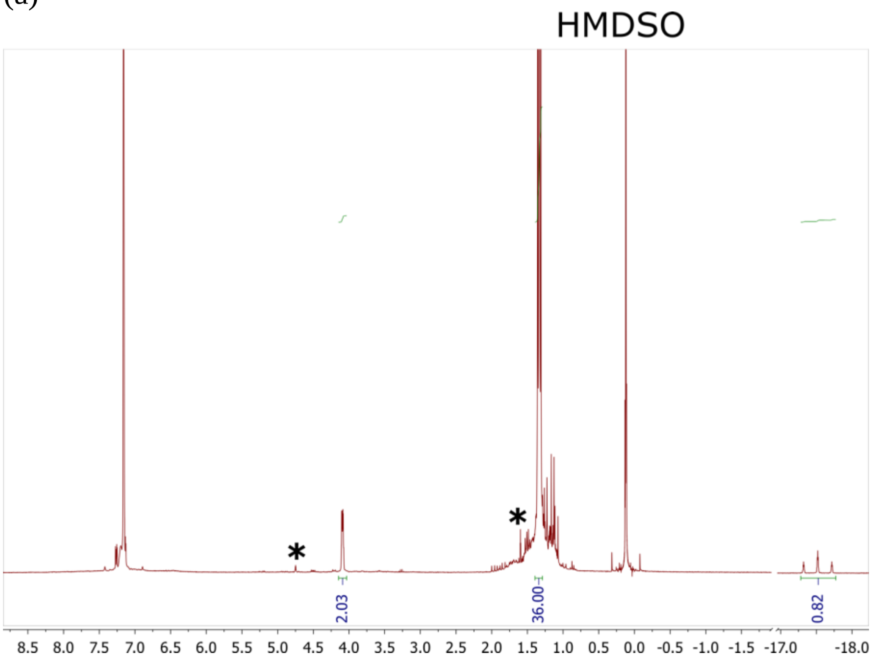

(b)

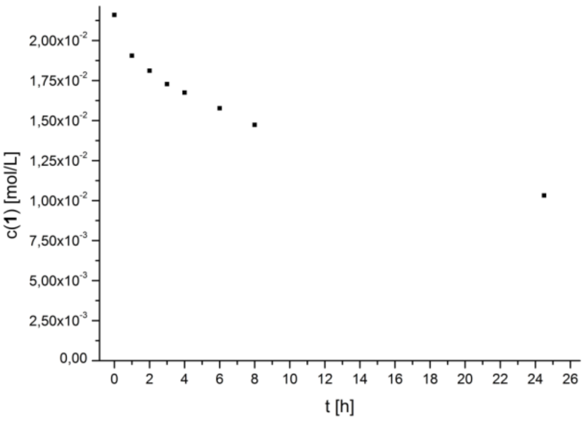

Supplementary Figure 17. (a) ^{1}H NMR spectrum of **1** in C_6D_6 after 24.5 h of photolysis ($\lambda_{exc.}$ > 305 nm, * Isobutene). (b) Conversion of **1** by photolysis ($\lambda_{exc.}$ > 305 nm) under Argon atmosphere.

3 mg (7.2 µmol, 1.00 eq) **1** are dissolved in 0.5 mL THF, filled in a J-Young NMR tube and photolyzed ($\lambda_{exc.}$ > 305 nm) overnight. 100 µL CH_4 is added as internal standard via a septum and 1 mL of the headspace is analyzed by TCD-GC showing formation of 1.2 µmol (0.33 eq) H_2.

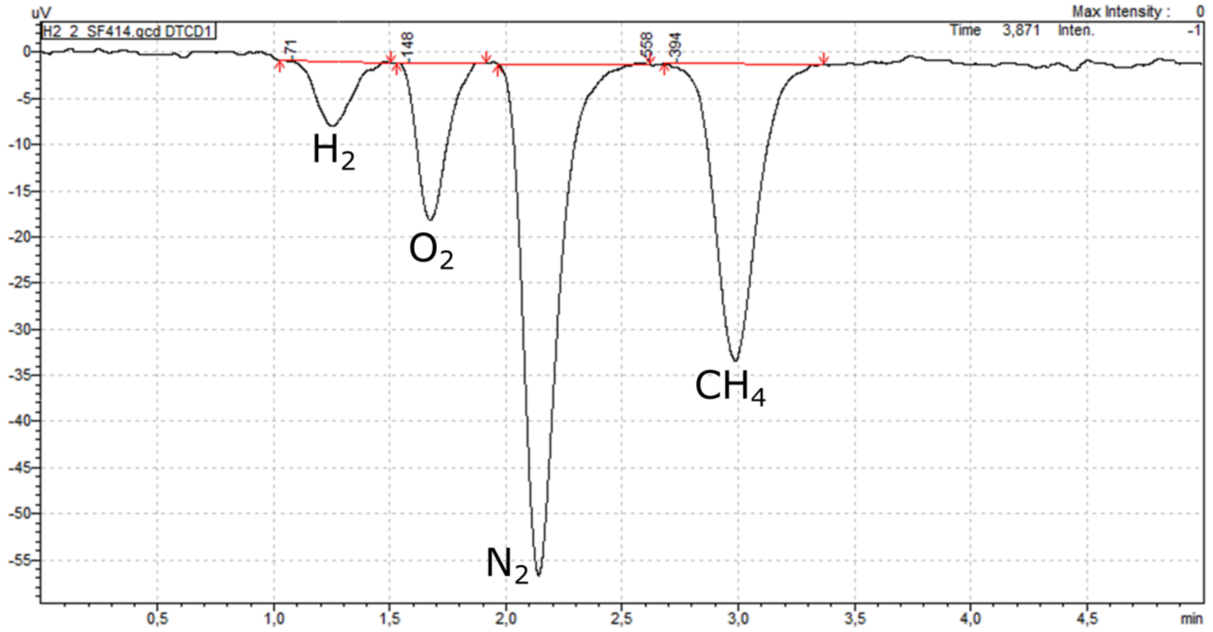

Supplementary Figure 18. Gas chromatogram of the headspace of the irradiation of **1** in THF.

H/D exchange with TEMPO-D:

3.3 mg (0.00793 mmol, 1.00 eq) **1** and 13 mmol (0.0821 mmol, 10.35 eq) TEMPO-D are dissolved in 0.5 mL THF-d_8 and 2 µL HMDSO are added as internal standard. The sample is filled in a J-Young NMR tube and stirred for 1.5 h at RT before photolysis ($\lambda_{exc.}$ > 305 nm).

(a) (b)

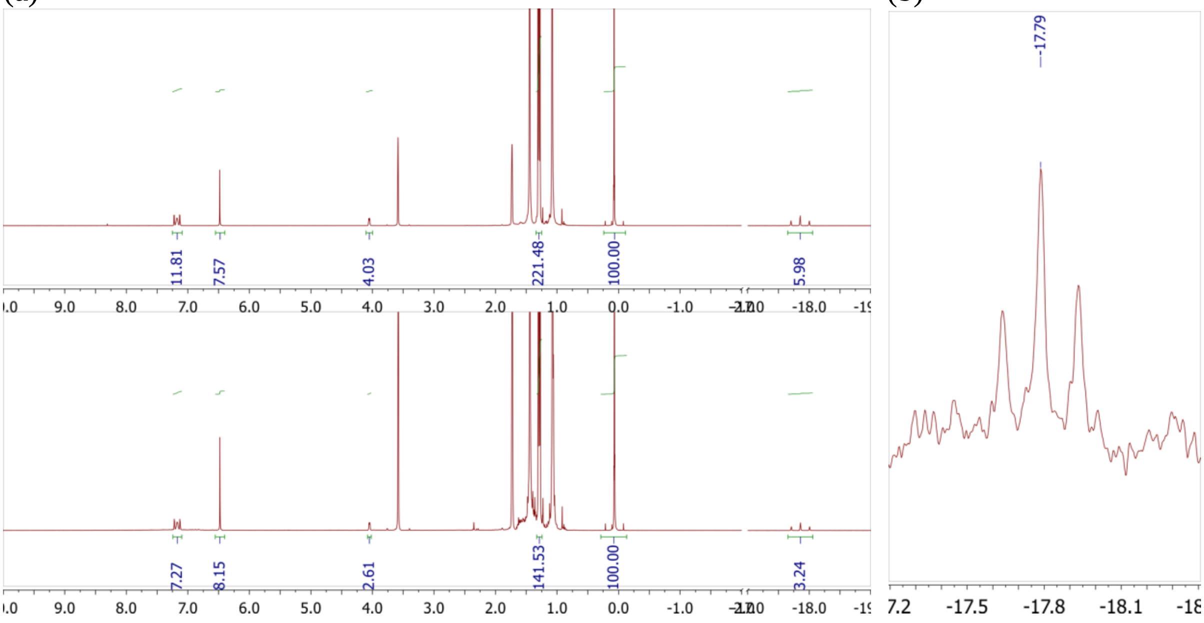

Supplementary Figure 19. (a) ^{1}H NMR spectrum of the photolysis ($\lambda_{exc.}$ > 305 nm) of **1** in the presence of TEMPO-D; bottom: after stirring at RT for 1.5 h showing 67% deuterium labeling of the PCH position. Top: after photolysis for 3.5 h showing 67% deuterium labeling of the PCH position and 15% deuterium labeling of the Ni-H position. Deuteration is determined by comparison of the integrals with the integral of the *t*Bu groups. (b) ^{2}H NMR spectrum after 3.5 h of photolysis.

Rate dependence of abnormal CO_2 insertion on added **7**:

7 is filled into a J-Young NMR tube and 0.5 mL of a 9.69 mM solution of **1** in THF-d_8 containing HMDSO as internal standard are filled into a J-Young NMR tube. The solution is degassed by three pump-freeze-thaw cycles, 1 atm CO_2 pressure (≥99.9993% purity, purification by passing through P_4O_{10}, Drierite and cooling to -40°C) is applied and the sample is photolyzed ($\lambda_{exc.}$ > 305 nm).

(a)

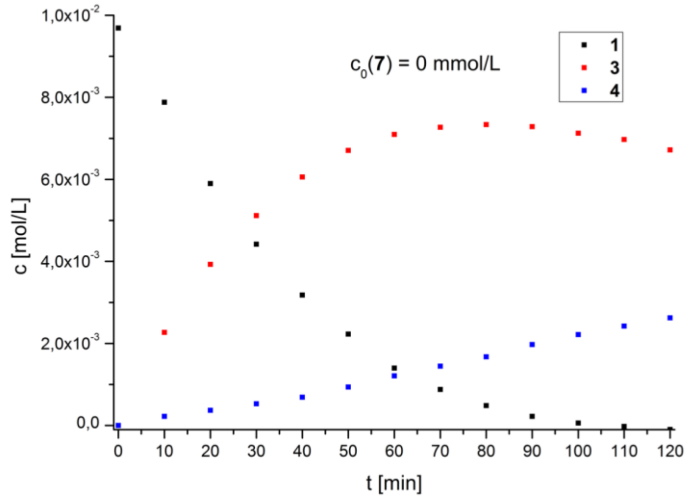
c₀(7) = 0 mmol/L
1
3
4
c [mol/L]
t [min]

(b)

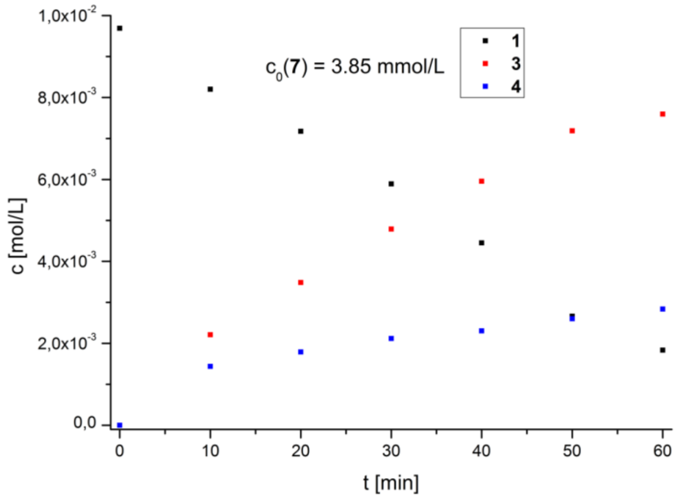
c₀(7) = 3.85 mmol/L
1
3
4
c [mol/L]
t [min]

(c)

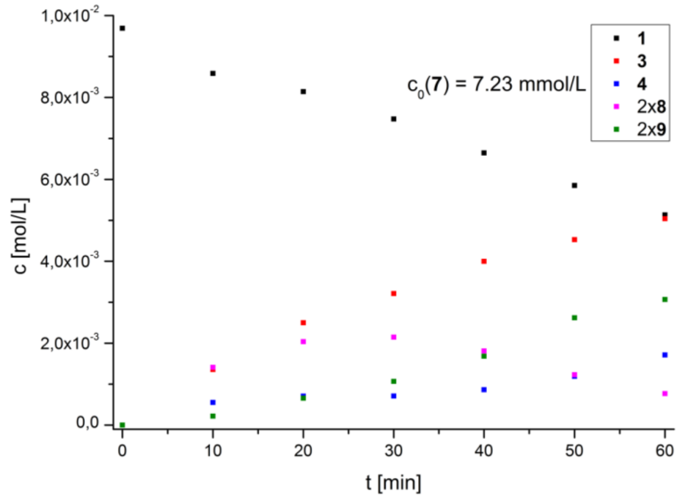
c₀(7) = 7.23 mmol/L
1
3
4
2x8
2x9
c [mol/L]
t [min]

(d)

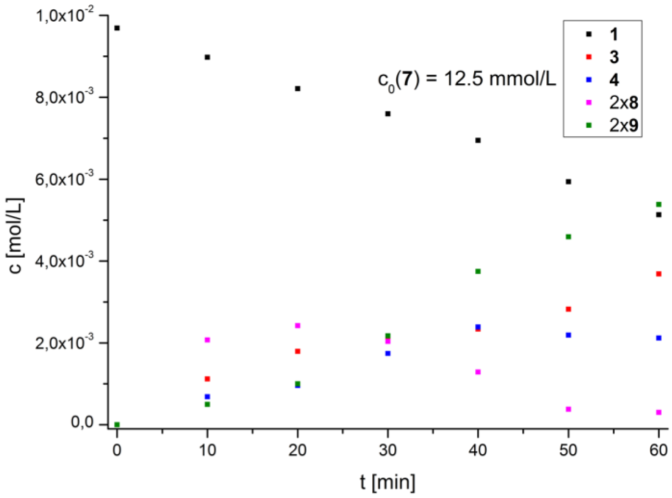

Supplementary Figure 20. Plot of concentration *vs*. time for the photochemical reaction of **1** with CO_2. (a) c_0(**7**) = 0 mmol/L, (b) c_0(**7**) = 3.85 mmol/L, (c) c_0(**7**) = 7.23 mmol/L and (d) c_0(**7**) = 12.5 mmol/L. Concentrations are determined by $^{1}H\{^{31}P\}$ NMR spectra by integration *vs*. the internal standard.

(a)

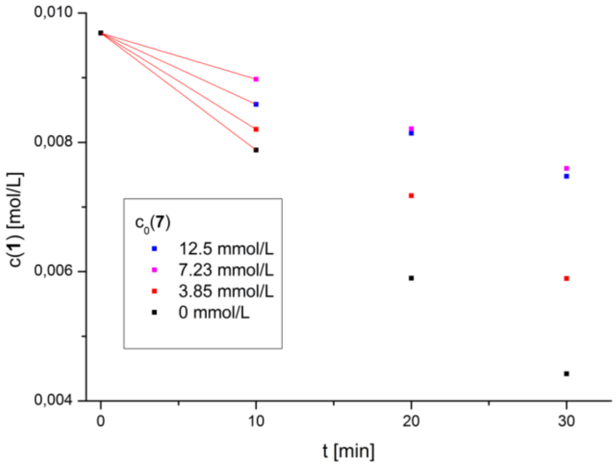

(b)

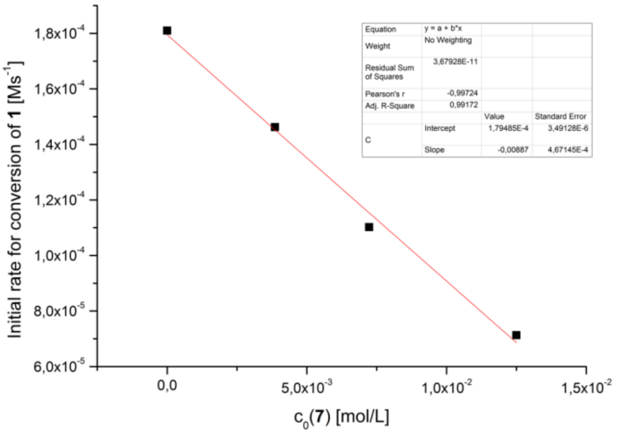

Supplementary Figure 21. (a) Plot of concentration *vs*. time and (b) plot of initial rate of **1** conversion *vs*. c_0(**7**) for the photochemical reaction of **1** with CO_2. Concentrations are determined by $^1H\{^{31}P\}$ NMR spectra by integration *vs*. the internal standard.

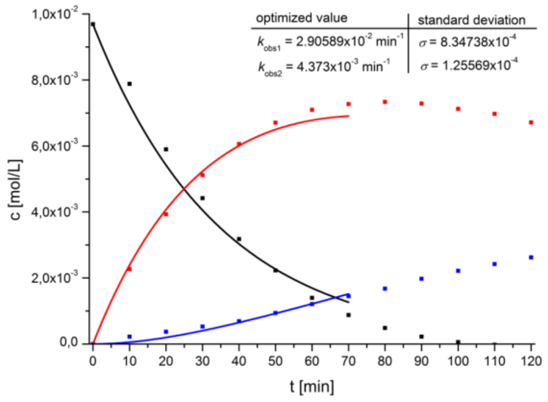

Supplementary Figure 22. Plot of c *vs*. time for the photochemical reaction of **1** with CO_2 (c_0(**7**) = 0 mmol/L) and global fit (black: **1**; red: **3**; blue: **4**).

Rate dependence of abnormal CO_2 insertion on photon flux:

0.5 mL of a 9.69 mM solution of **1** in THF-d_8 containing HMDSO as internal standard are filled into a J-Young NMR tube. The solution is degassed by three pump-freeze-thaw cycles, 1 atm CO_2 pressure (≥99.9993% purity, purification by passing through P_4O_{10}, Drierite and cooling to -40°C) is applied and the sample is photolyzed ($\lambda_{exc.}$ > 305 nm) with different lamp intensity.

(a)

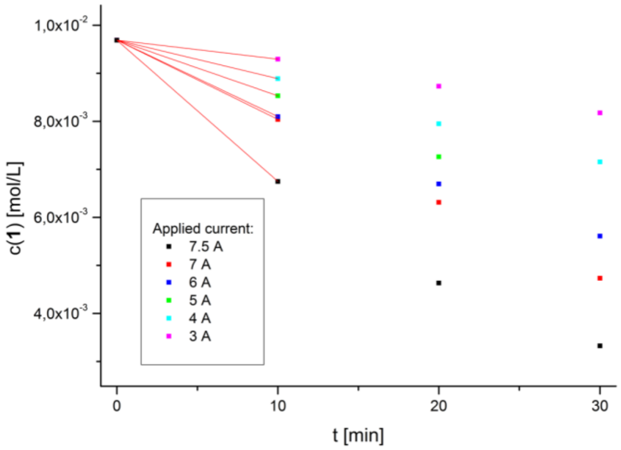

(b)

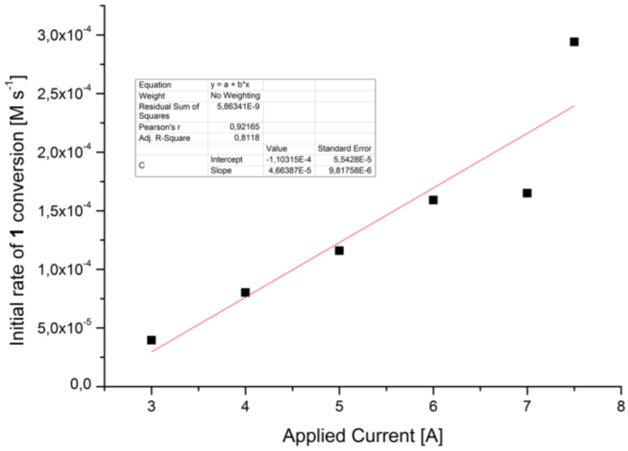

Supplementary Figure 23. (a) Plot of concentration *vs*. time and (b) plot of initial rate of **1** conversion *vs*. applied current for the photochemical reaction of **1** with CO_2 at different lamp intensities. Concentrations are determined by $^{1}H\{^{31}P\}$ NMR spectra by integration *vs*. the internal standard.

Rate dependence of abnormal CO_2 insertion on CO_2 pressure:

0.3 mL of a 9.69 mM solution of **1** in THF-d_8 containing HMDSO as internal standard are filled into a Medium Wall Precision Pressure/Vacuum Valve NMR tube. The solution is degassed by three pump-freeze-thaw cycles, CO_2 pressure (≥99.9993% purity, purification by passing through P_4O_{10}, Drierite and cooling to -40°C) is applied and the sample is photolyzed ($\lambda_{exc.}$ > 305 nm).

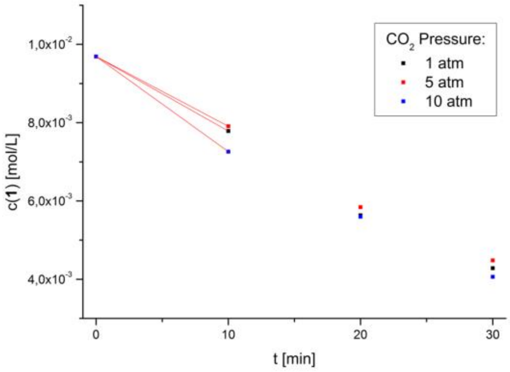

Supplementary Figure 24. Plot of concentration *vs*. time for the photochemical reaction of **1** with CO_2 at different CO_2 pressures. Concentrations are determined by $^{1}H\{^{31}P\}$ NMR spectra by integration *vs*. the internal standard.

Isotopic labeling studies:

0.5 mL of a 9.69 mM solution of **1-D** in THF-d_8 containing HMDSO as internal standard are filled into a J-Young NMR tube. The solution is degassed by three pump-freeze-thaw cycles, 1 atm CO_2 pressure (≥99.9993% purity, purification by passing through P_4O_{10}, Drierite and cooling to -40°C) is applied and the sample is photolyzed ($\lambda_{exc.}$ > 305 nm).

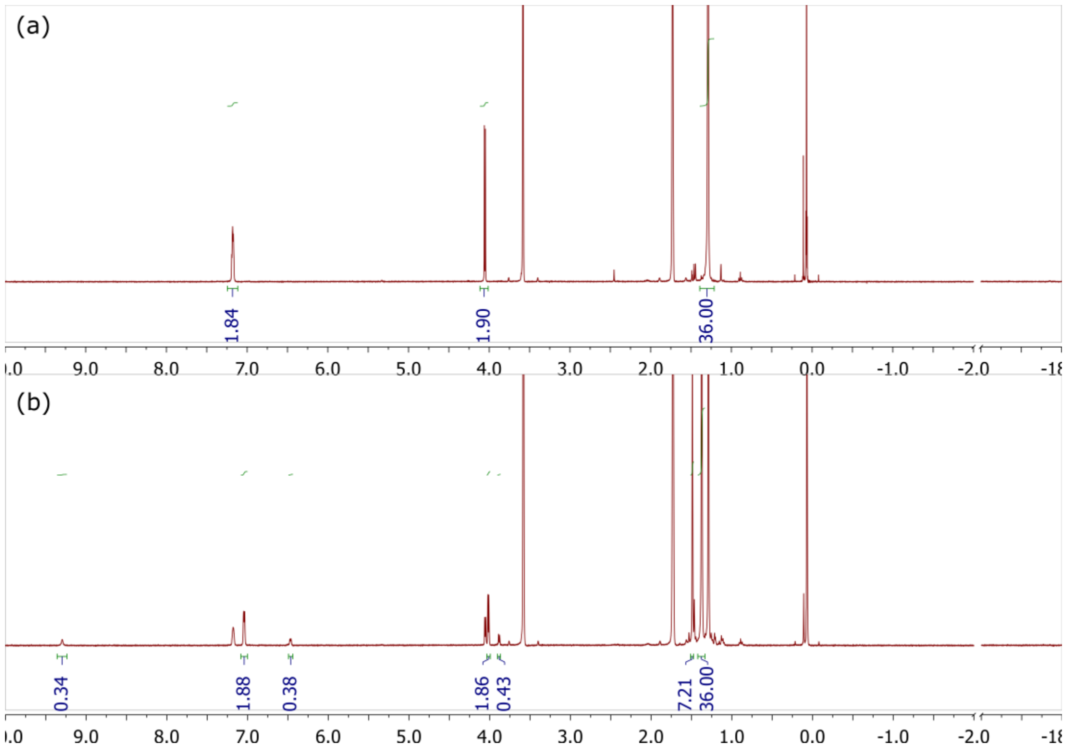

Supplementary Figure 25. $^{1}H\{^{31}P\}$ NMR spectrum of the photolysis ($\lambda_{exc.} > 305$ nm) of **1-D** under 1 atm CO_2; (a) before photolysis showing complete deuteration of the Ni-D position. (b) After 1h photolysis showing 72% deuterium labeling of the OH position of **3-D** and **4-D**. Deuteration is determined by comparison of the integrals with the integral of the *t*Bu groups.

Deprotonation of **3**:

3 mg (6.5 µmol, 1.00 eq) **3** and 1.3 mg NaHMDS are dissolved in 0.5 mL of THF-d_8.

$^{31}P\{^1H\}$ NMR (162 MHz, THF-d_8) δ: 57.5 ppm. ^1H NMR (400 MHz, THF-d_8) δ: 7.03 (ABXX'B'A', $N = |^3J_{A\text{-}X} + {}^4J_{A\text{-}X'}|$ = 18.6 Hz, $^3J_{A\text{-}B}$ = 5.3 Hz, 2H, NC*H*), 3.80 (d, $^3J_{H\text{-}H}$ = 5.2 Hz, 2H, PC*H*), 1.50 ($A_{18}XX'A'_{18}$, $N = |^3J_{A\text{-}X} + {}^5J_{A\text{-}X'}|$ = 6.5 Hz, 36H, P*t*Bu) ppm.

(a)

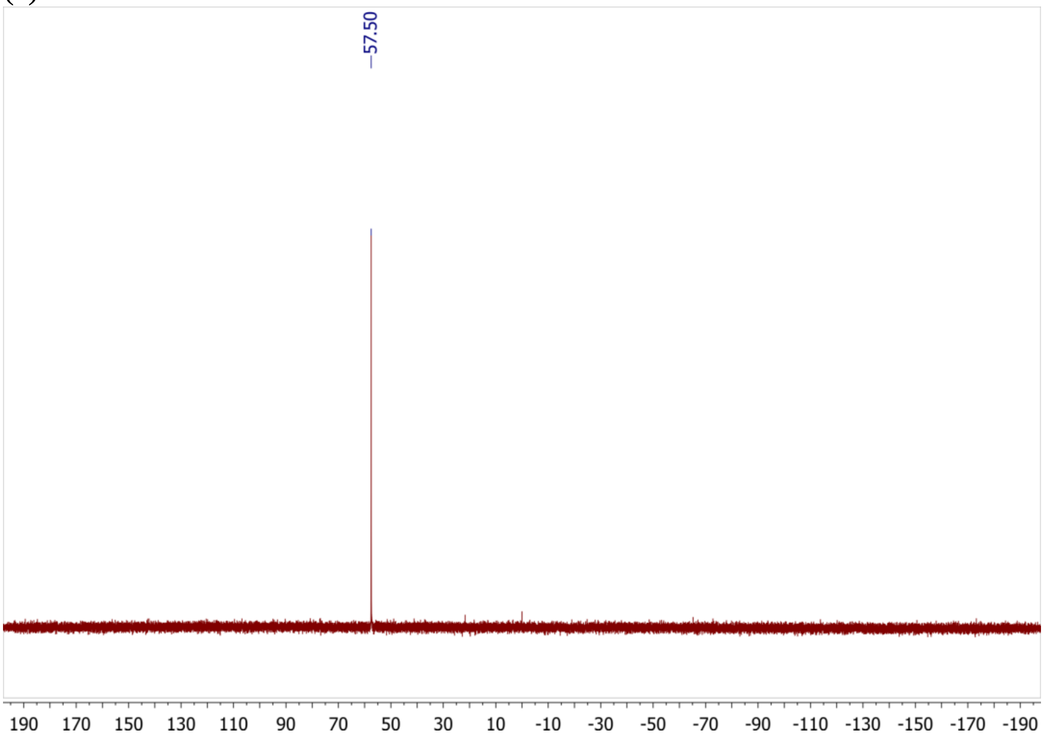

(b)

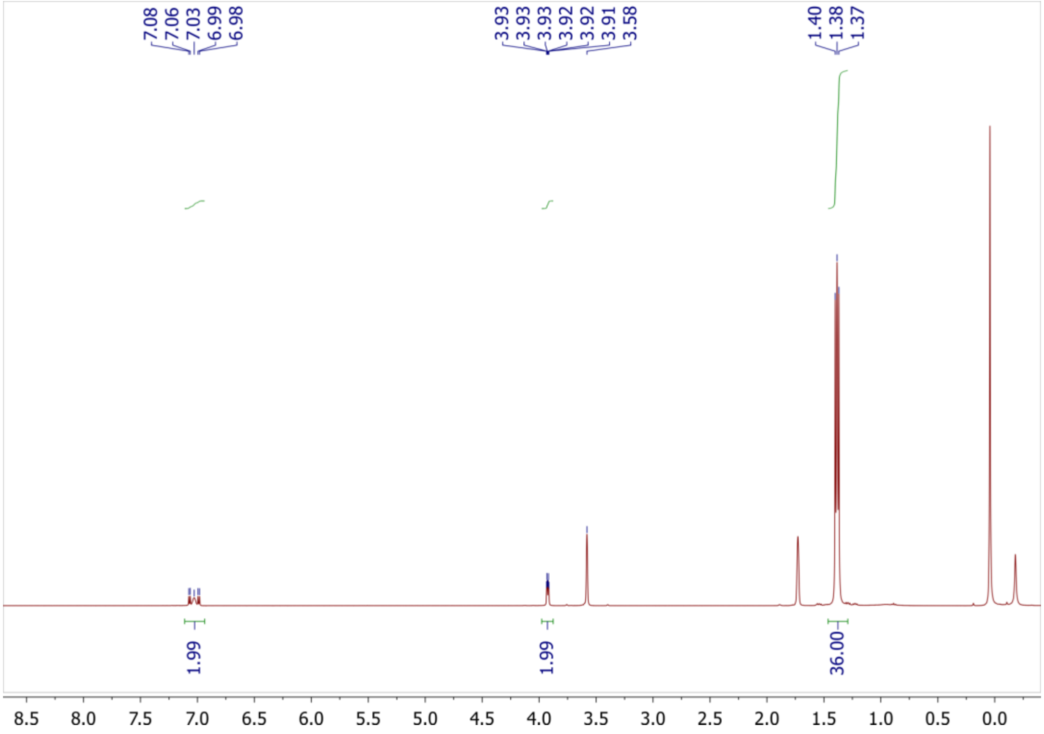

Supplementary Figure 26. (a) $^{31}P\{^{1}H\}$ NMR and (b) ^{1}H NMR spectra of **3** and NaHMDS in THF-d_8.

3 mg (6.5 µmol, 1.00 eq) **3** are dissolved in 2 mL THF-d_8 and samples of the solution are measured by $^{31}P\{^1H\}$ NMR spectroscopy in the presence of 1 eq of DBU/TBD/NaHMDS and in the absence of base.

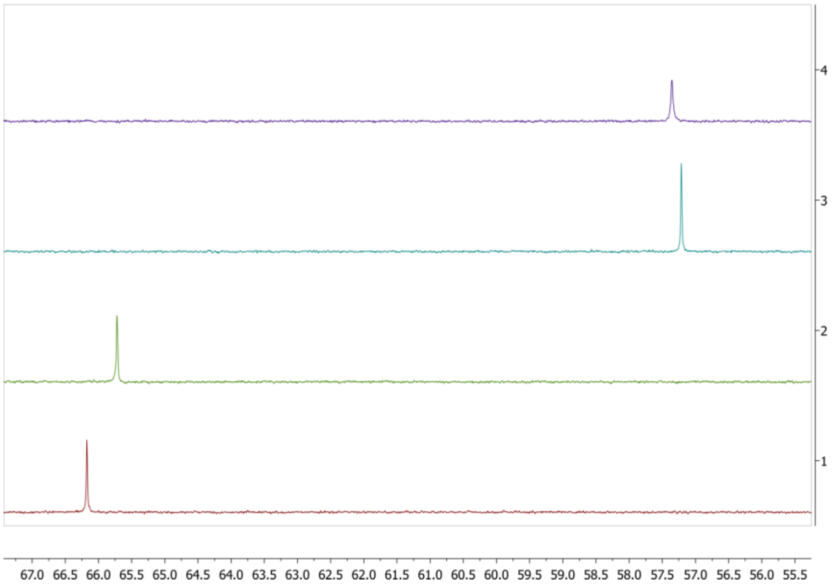

Supplementary Figure 27. $^{31}P\{^1H\}$ NMR spectrum of the reaction of **3** without base (bottom), with 1 eq DBU (lower middle), with 1 eq TBD (upper middle) and 1 eq NaHMDS (top) in THF-d_8.

pK_{ip}^{THF}(**3**) and pK_{α}^{THF}(**3**) estimation:

The ΔG of proton transfer from **3** to THF is defined by Supplementary Equation 1:

$$\Delta G = -\ln(K_{\mathbf{3}+THF})\cdot RT \qquad (1)$$

With the equilibrium constant $K_{\mathbf{3}+\mathrm{THF}}$ for the protonation of THF by **3**, which can be determined from the pK_A of **3** and $[H(THF)_x]^+$. Since ion pairing plays an important role in unpolar solvents, deprotonation of **3** with base is assumed to give the ion pair [H(base)][**3-H**].*(4)* Therefore the experimentally observable equilibrium constant K_{eq} gives pK_{ip}^{THF}(**3**), the acidity constant of the

ion pair. To account for the dissociation equilibrium of the ion pair [H(base)][**3-H**] to the completely solvated ions, a correction according to the Fuoss equation has to be done.*(5-6)*

By assumption of complete deprotonation of **3** in the presence of 1 eq NaHMDS, K_{eq} for the reaction of **3** with 1 eq DBU is estimated according to Supplementary Equation 2 (δ(**3**) = 66.17, δ(**3-H**) = 57.35, δ(**3**+DBU) = 65.72):

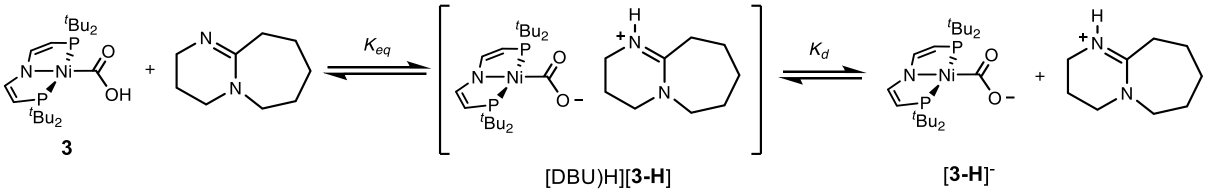

$$K_{eq} = c(\mathbf{3\text{-}H})^2 / c(\mathbf{3})^2 = \chi(\mathbf{3\text{-}H})^2 / \chi(\mathbf{3})^2 \quad (2)$$

$$\delta(\mathbf{3}\text{+DBU}) = \chi(\mathbf{3})\,\delta(\mathbf{3}) + \chi(\mathbf{3\text{-}H})\,\delta(\mathbf{3\text{-}H}) \quad (3)$$

with

$$\chi(\mathbf{3}) + \chi(\mathbf{3\text{-}H}) = 1 \quad (4)$$

$$\chi(\mathbf{3\text{-}H}) = 0.051 \quad (5)$$

and

$$K_{eq} = 0.0028 \quad (6)$$

The pK_{ip}^{THF}(**3**) can then be estimated by Supplementary Equation 5 using pK_{ip}^{THF}(DBU) from literature:*(7)*

$$pK_{ip}^{THF}(\mathbf{3}) = pK_{ip}^{THF}(\text{DBU}) - \log(K_{eq}) \quad (7)$$

$$pK_{ip}^{THF}(\mathbf{3}) = 18.0 + 2.6 = 20.6 \quad (8)$$

With pK_{ip}^{THF}(**3**) and $pK_a([H(THF)_x]^+) = 0$ the equilibrium constant $K_{ip,\mathbf{3}+THF}$ for proton transfer from **3** to THF giving the ion pair $[H(THF)_x]$[**3-H**] can be estimated by Supplementary Equation 9:*(8)*

$$\mathrm{p}K_{\mathrm{ip},\mathbf{3}+\mathrm{THF}} = \mathrm{p}K_{\mathrm{ip}}^{\mathrm{THF}}(\mathbf{3}) - \mathrm{p}K_{\mathrm{a}}([\mathrm{H(THF)_x}]^{+}) \quad (9)$$

$$\mathrm{p}K_{\mathrm{ip},\mathbf{3}+\mathrm{THF}} = 20.6 \quad (10)$$

$$K_{\mathrm{ip},\mathbf{3}+\mathrm{THF}} = 10^{-20.6} \quad (11)$$

With equilibrium constant $K_{\mathrm{ip},\mathbf{3}+\mathrm{THF}}$ a free energy of +28.1 kcal/mol can be estimated for the protonation of THF by **3** giving ion-pair [H(THF)$_x$][**3-H**].

Upon correcting for ion-pairing in THF solution the dissociation constant K_d of [H(DBU)][**3-H**] has to be considered in Supplementary Equation 12:*(4-6*)

$$\mathrm{p}K_{\alpha}^{\mathrm{THF}}(\mathbf{3}) = \mathrm{p}K_{\alpha}^{\mathrm{THF}}(\mathrm{DBU}) - \log(K_{\mathrm{eq}}) + \Delta \mathrm{p}K_{\mathrm{d}} \quad (12)$$

$K_\mathrm{d} = 2 \cdot 10^{-6}$ is calculated using the Fuoss model of ion-pairs with a inter-ion distance of a = 5.5 Å estimated based on the crystal structure of **3** and the crystal structure of protonated DBU from literature.*(5-6,9)*

$$\mathrm{p}K_{\alpha}^{\mathrm{THF}}(\mathbf{3}) = \mathrm{p}K_{\alpha}^{\mathrm{THF}}(\mathrm{DBU}) - \log(K_{\mathrm{eq}}) + \Delta \mathrm{p}K_{\mathrm{d}} \quad (13)$$

$$\mathrm{p}K_{\alpha}^{\mathrm{THF}}(\mathbf{3}) = 16.8 + 2.6 + 5.6 = 25.0 \quad (14)$$

With $\mathrm{p}K_{\alpha}^{\mathrm{THF}}(\mathbf{3})$ and $\mathrm{p}K_{\mathrm{a}}([\mathrm{H(THF)_x}]^{+}) = 0$ the equilibrium constant $K_{\mathbf{3}+\mathrm{THF}}$ for proton transfer from **3** to THF can be estimated. The inter-ion distance a = 5.7 Å is estimated based on the crystal structure of **3** and the crystal structure of [H(THF)$_2$][$CHB_{11}H_5Br_6$] from literature:(10)

$$\mathrm{p}K_{\mathbf{3}+\mathrm{THF}} = \mathrm{p}K_{\alpha}^{\mathrm{THF}}(\mathbf{3}) - \mathrm{p}K_{\mathrm{a}}([\mathrm{H(THF)_x}]^{+}) - \Delta \mathrm{p}K_{\mathrm{d}} \quad (15)$$

$$\mathrm{p}K_{\mathbf{3}+\mathrm{THF}} = 25.0 - 5.4 \quad (16)$$

$$K_{\mathbf{3}+\mathrm{THF}} = 10^{-19.6} \quad (17)$$

With equilibrium constant K_{3+THF} a free energy of +26.7 kcal/mol can be estimated for the protonation of THF by **3** giving solvated ions.

EPR Spectroscopic Characterization:

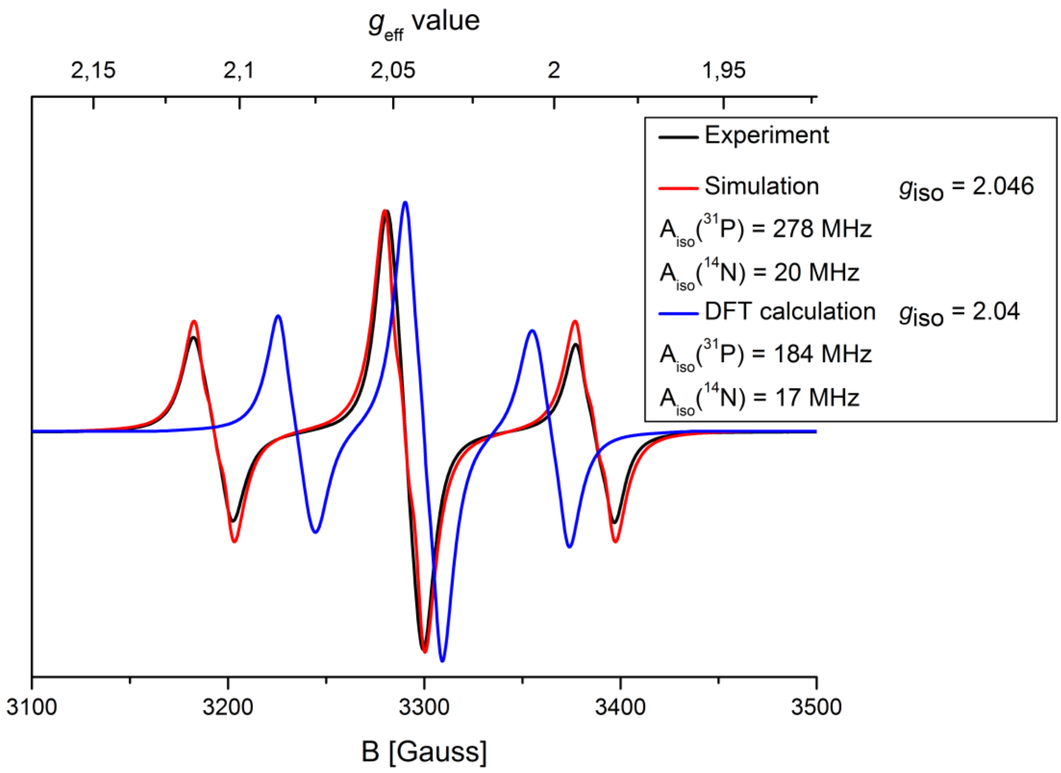

Supplementary Figure 28. X-Band EPR spectrum of **5** in THF (RT, c(**5**) = 17 mmol, ν = 9.435505GHz). For EPR spectrum of **5** from photolysis of **1** under CO_2, see Supplementary Figure 29.

0.5 mL of a 9.69x10^{-3} m solution (7.3 mmol) of **1** in THF-d_8 containing 10 µL hexamethyldisiloxane as internal standard are filled into a J-Young NMR tube. The solution is degassed by 3 pump-freeze-thaw cycles and 1 atm CO_2 pressure (≥99.9993% purity, purification by passing through P_4O_{10}, Drierite and cooling to -40°C) is applied and the sample is photolyzed ($\lambda_{exc.}$ > 305 nm) for 2 hours.

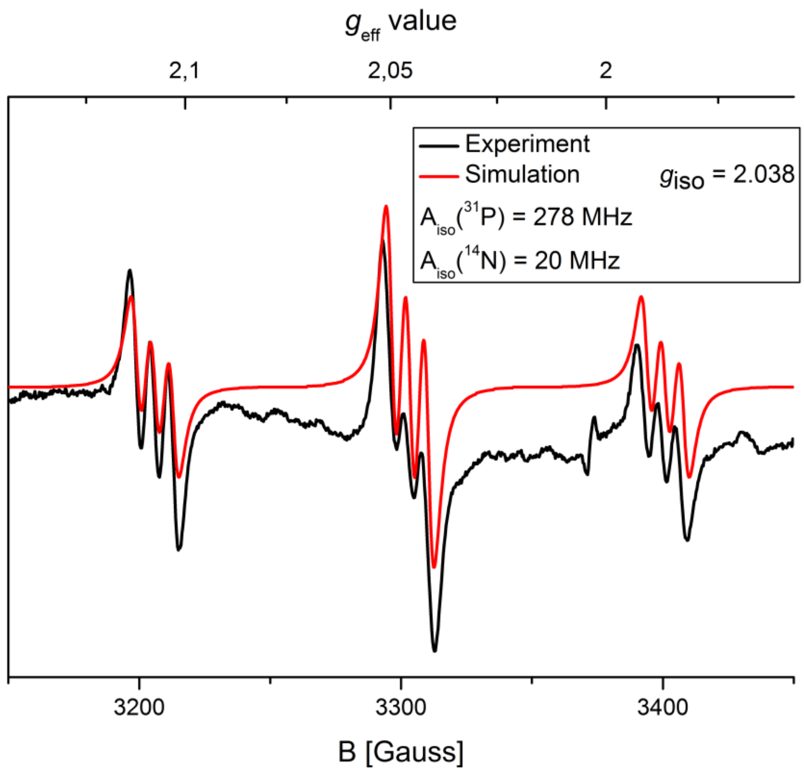

Supplementary Figure 29. X-Band EPR spectrum of a sample of **1** photolyzed under CO_2 pressure in THF-d_8 showing formation of 5 (RT, ν = 9.456767 GHz).

4.4 mg **1** are dissolved in 0.5 mL THF-d_8 and 3 µL HMDSO are added as internal standard. The sample is filled in a J-Young NMR tube and the sample is photolyzed ($\lambda_{exc.}$ > 305 nm). 57% conversion of **1** is observed by ^{1}H NMR spectroscopy. 0.2 mL of the sample are filled into a quartz tube and EPR is measured.

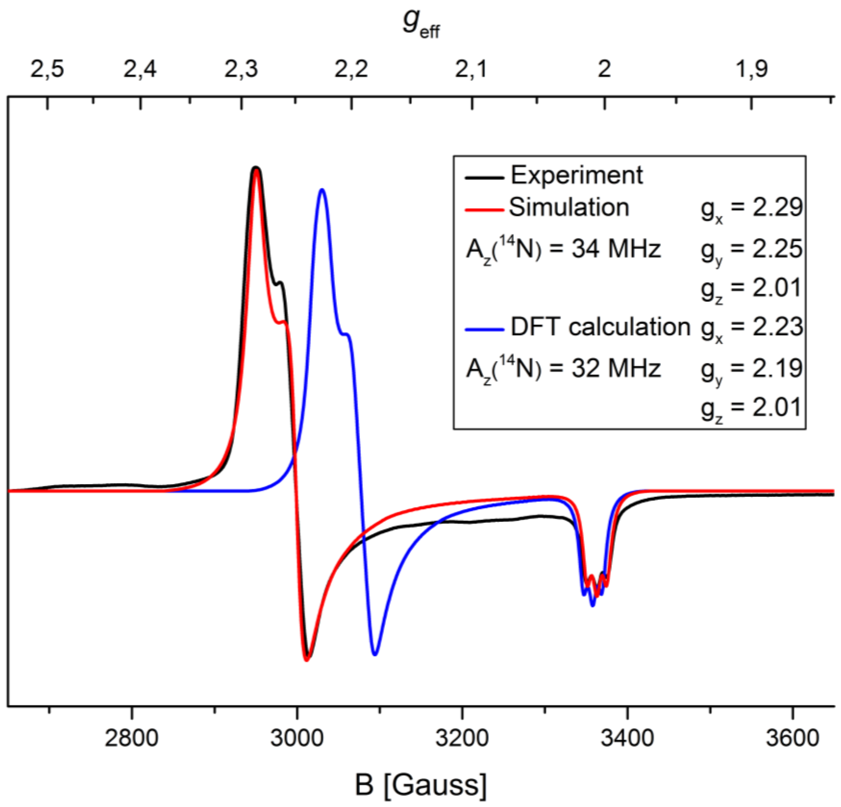

Supplementary Figure 30. X-Band EPR spectrum of a sample of **1** photolyzed under Ar in THF-d_8 showing formation of 7 (152 K, c(7) = 12.1 mmol/L, ν = 9.44016 GHz).

UV/Vis Spectroscopic Characterization:

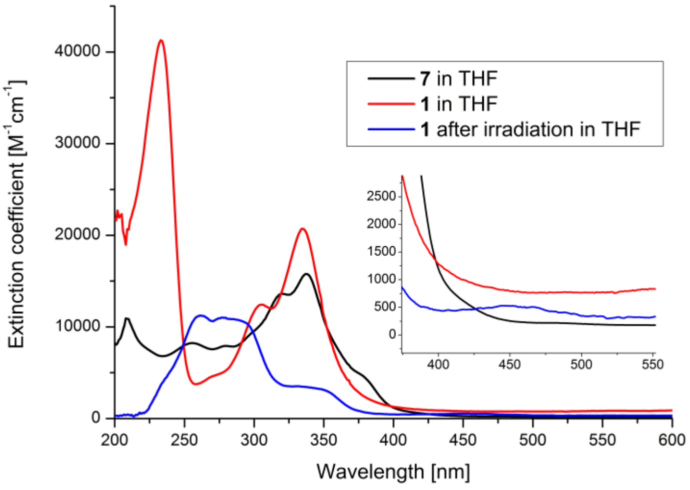

Supplementary Figure 31**.** UV/Vis spectra of **1** and **7** in THF and UV/Vis spectrum of **1** after 16 h of photolysis ($\lambda_{exc.}$ > 305 nm) in THF.

Infrared Spectroscopic Characterization:

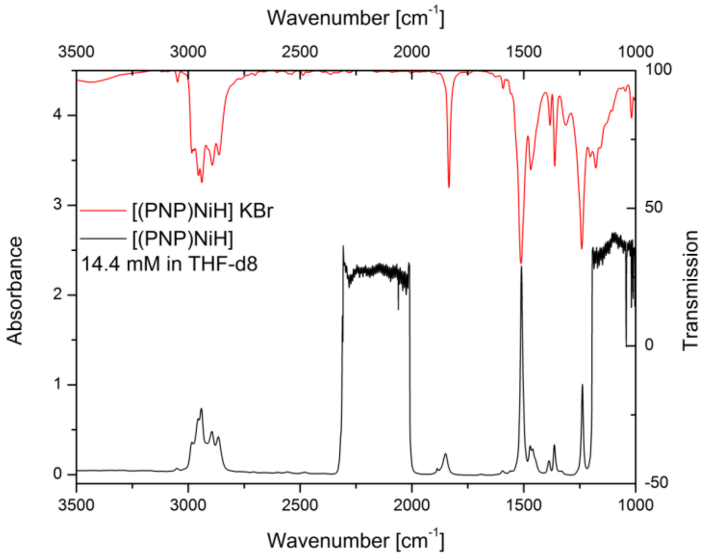

Supplementary Figure 32. Infrared spectra of **1** as KBr pellet (red) and as solution in THF-d_8 (black).

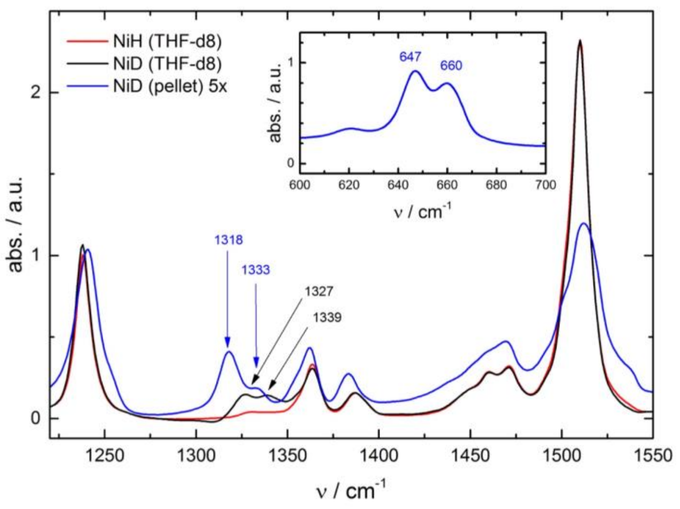

Supplementary Figure 33. Infrared spectra of **1** and **1-D** as solution in THF-d_8 (black).

UV-pump-UV/Vis-probe:

UV/Vis pump-probe experiments were performed with a 1 kHz Ti:sapphire regenerative amplifier system (Clark-MXR, CPA-1000) producing 150 fs pulses at 775 nm. Pump pulses were generated by second harmonic generation resulting in 387 nm pulses, which were focused to a diameter of about 200 μm at the sample. To exclude multi-photon processes the pump pulse energies were set below 1 μJ. For probing a white-light continuum was generated by focusing a small portion of the 775 nm pulse in a CaF_2 crystal of 4 mm thickness. The white-light was split into a reference and a probe beam, with the probe being superimposed with the pump pulse in the sample cell. The spectra of reference and probe continua were each recorded at wavelengths 350-730 nm with a 256-element linear diode array attached to a spectrograph. A translation stage was used to adjust the time delay between pump and probe pulses covering a time range of up to 1 ns. The relative plane of polarization between pump and probe was set to the magic angle of 54.7°. The measured time-dependent transient spectra were corrected for a wavelength dependent temporal shift introduced by group delay dispersion within the white-light-probe continuum. Experiments were performed with a hermetically sealed quartz glass cell of 2 mm optical path length filled under argon atmosphere. A magnetic stirrer was included to avoid accumulation of photoproducts in the laser focus. Sample concentrations were set to 6 mM in THF solution.

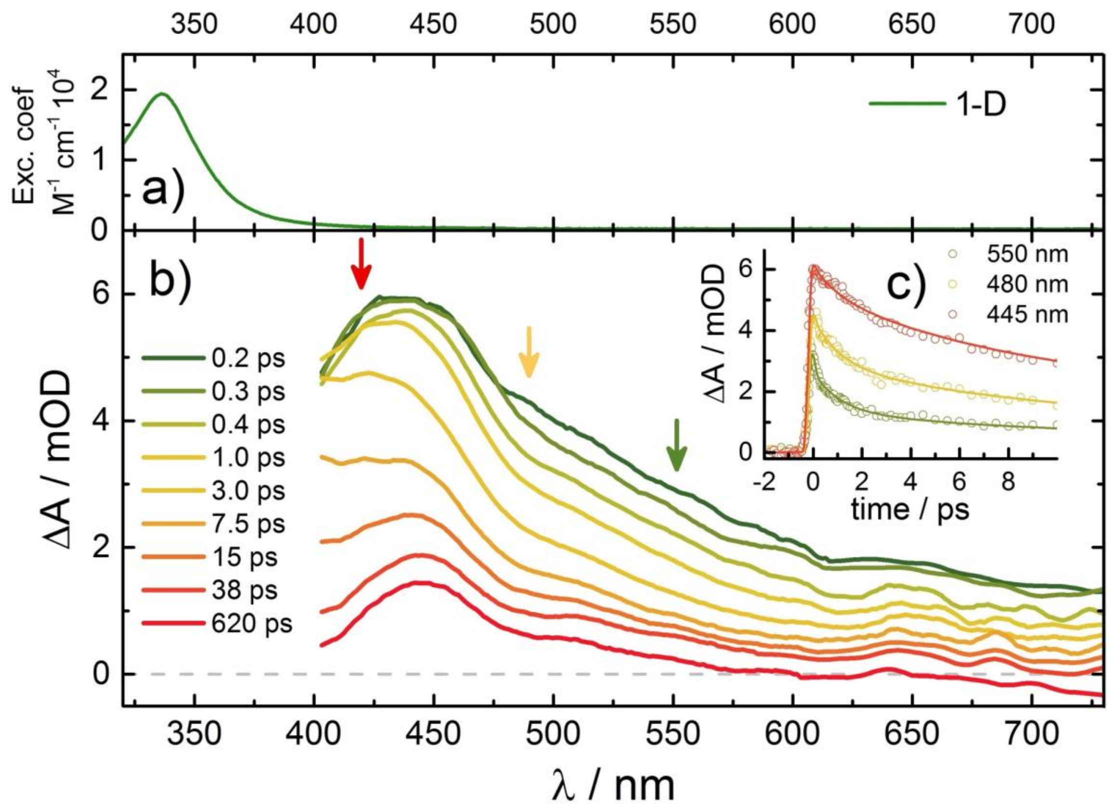

Supplementary Figure 34. (a) Transient difference spectra generated by 385 nm excitation of a 6 mM solution of **1-D** in THF for selected pump-probe delays. (b) Stationary absorption spectrum of **1-D** in THF- solution. (c) Time traces at 445 (red arrow), 480 (yellow arrow) and 550 nm (green arrow) as well as global tri-exponential fits with time constants of $\tau_1 = 1.1 \pm 0.2$ ps, $\tau_2 = 10 \pm 1$ ps and $\tau_3 >> 1$ ns (solid lines).

UV-pump-IR-probe:

UV-pump-mid-IR-probe experiments were performed with a 1 kHz Ti:sapphire oscillator/regenerative amplifier system (Coherent, Libra) producing 100 fs pulses at 800 nm. Part of the output energy was used to generate pump pulses at 400 nm by second harmonic generation. Pulse energies of about 0.4 µJ focused to a diameter of about 200 µm were used to excite the sample. Tuneable mid-IR probe pulses were generated by difference frequency mixing of idler and signal pulses from a home-build two stage optical parametric amplifier (OPA)*(11)* pumped by 0.5 mJ of the regenerative amplifier output. The mid-IR beam was split into a reference and a probe beam. The probe pulse passed a translation stage and was

superimposed with the pump pulse in the sample cell. To eliminate over-all molecular rotational effects to the signal the relative plane of polarization between pump and probe was set to the magic angle of 54.7°. Probe and reference pulses were directed to a polychromator and separately detected by a liquid-nitrogen cooled HgCdTe-detector of 2 x 32 pixels. To minimize CO_2 and water absorptions the mid-IR beam path was purged with dry nitrogen. The hermetically sealed stainless steel sample cell equipped with two CaF_2 windows of 1 mm thickness and a magnetic stirrer had an optical path length of 0.6 mm. The cell was filled under argon atmosphere with 11 mM sample solutions in THF-d_8.

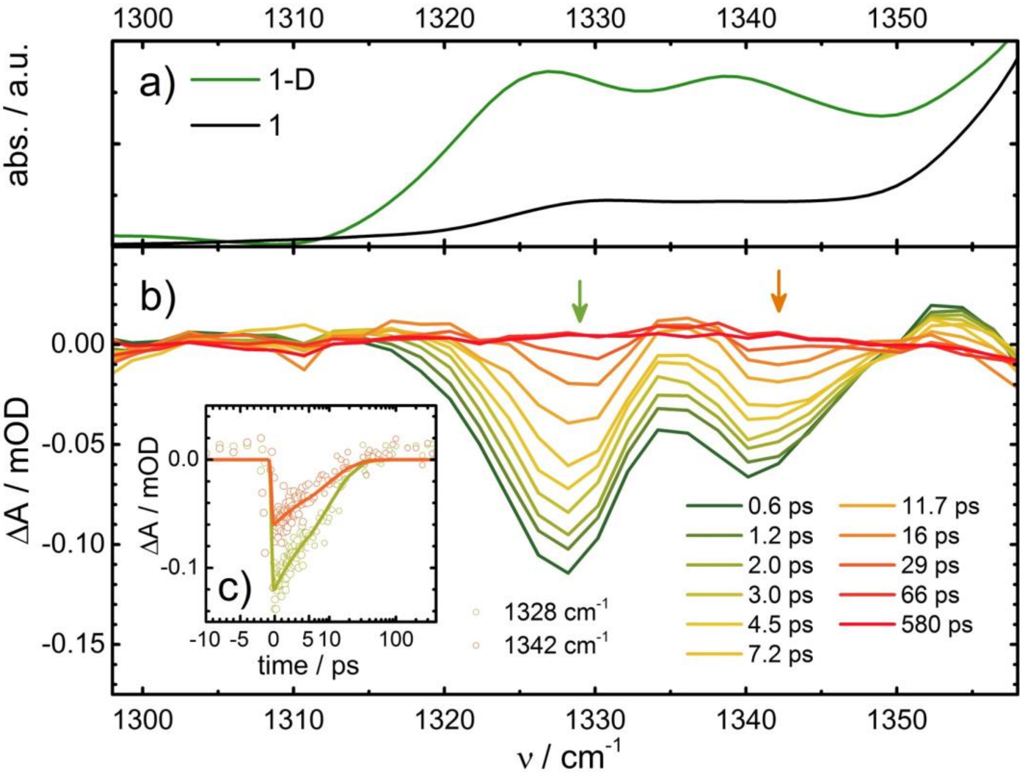

Supplementary Figure 35. (a) Transient difference spectra generated by 400 nm excitation of 15 mM solution of **1-D** in THF-d_8 (1298-1358 cm^{-1}) for selected pump-probe delays. (b) FTIR spectrum of **1** and **1-D** in THF-d_8 solution. (c): Time traces at 1328 (green) and 1342 cm^{-1} (orange) with mono-exponential fit (time constant: τ = 10 ± 2 ps).

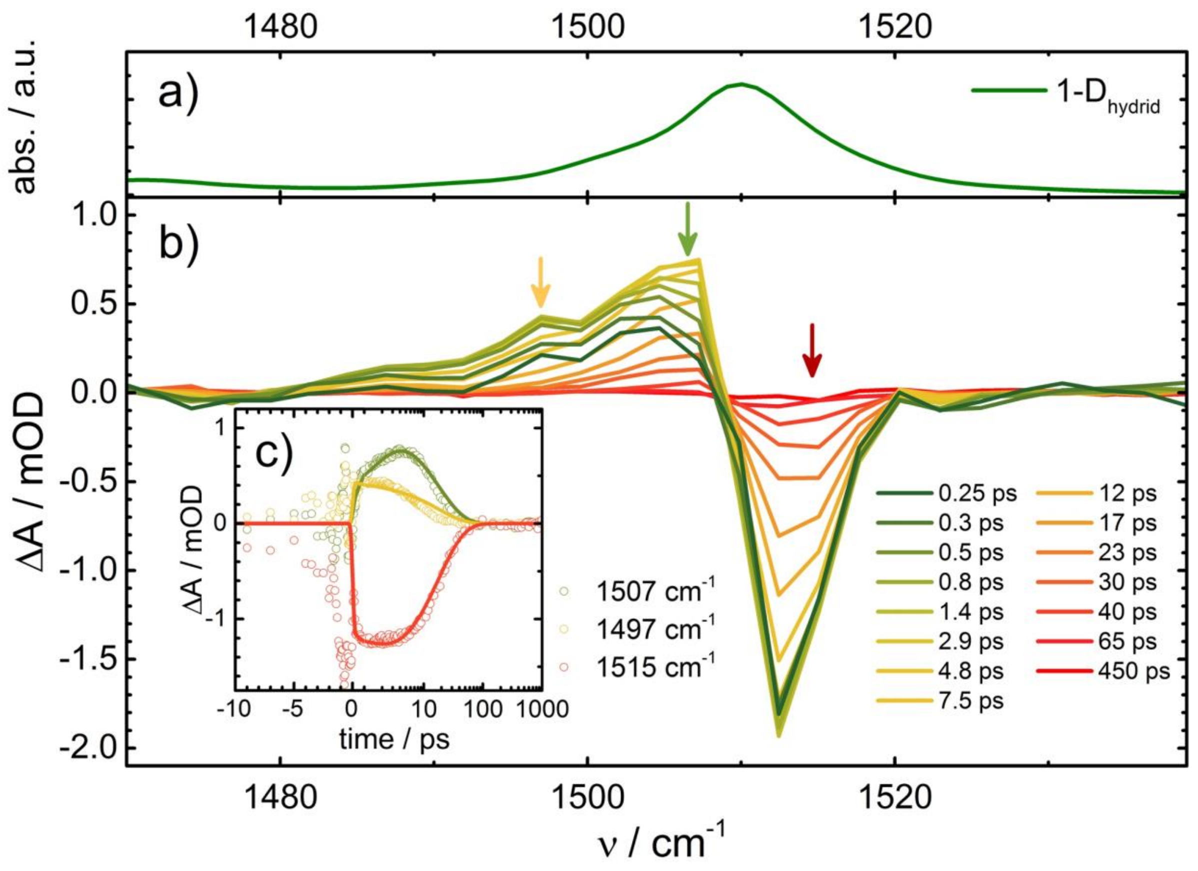

Supplementary Figure 36. (a) Transient difference spectra generated by 400 nm excitation of 3 mM solution of **1-D** in THF-d_8 (1298-1358 cm^{-1}) for selected pump-probe delays. (b) FTIR spectrum of **1-D** in THF-d_8 solution. (c): Time traces at 1507 (green), 1497 (yellow) and 1515 cm^{-1} (red) with bi-exponential fits (time constants: $\tau_1 = 1.7 \pm 0.5$ ps, $\tau_2 = 18 \pm 5$ps).

Actinometry and Quantum Yield Determination:

The photon flux of the lamp with a band gap filter (410 nm) was determined using potassium ferrioxalate as chemical actinometer. Photochemical decomposition of $K_3[Fe(C_2O_4)_3]$ by photolysis with 406.7 nm wavelength is described in the literature with a quantum yield of 1.188±0.002(5) and was used as reference.*(12)* Photochemical decomposition of potassium ferrioxalate was quantified using excess of 2,2'-bipyridine and measuring the characteristic absorption at 510 nm of the formed $[Fe(bipy)_3]^{2+}$ by UV/Vis spectroscopy. In a representative experiment 2 mL of 0.006 M solution of $K_3[Fe(C_2O_4)_3]$ was filled in a UV/Vis cuvette and photolyzed for a certain time with a certain lamp intensity. A 1 mL aliquot was taken, 1 mL of

a 0.0055 M solution of 2,2'-phenanthroline was added and the reaction was quenched using 1 mL of a Na_2OCCH_3 solution at pH =3.5 (pH was adjusted using H_2SO_4). The sample was then diluted to a total volume of 10 mL and left in the dark for 1 hour to equilibrate. Afterwards a UV/Vis spectrum was recorded.

The photon flux of the lamp was determined using the following equation:

$$I = (A \cdot V_2 \cdot V_3) / (\varepsilon \cdot \Phi_{406.7} \cdot t \cdot V_1) \qquad (18)$$

Where I is the photon flux, A is the absorbance of the photolyzed sample corrected for the blank, V_2 is the volume of the photolyzed actinometer solution (V_2 = 0.002 L), V_3 is the total volume of the diluted sample (V_3 = 10 mL), ε is the extinction coefficient of $[Fe(bipy)_3]^{2+}$ at 510 nm ($\varepsilon = 1.11 \cdot 10^4$ $M^{-1}cm^{-1}$)*(11)*, d is the path length of the cuvette (d = 1 cm), $\Phi_{406.7}$ is the quantum yield of photochemical $K_3[Fe(C_2O_4)_3]$ decomposition, t is the time of photolysis (t = 0.25 min) and V_1 is the volume of the aliquot taken from the photolyzed sample (V_1 = 1 mL).

A linear relationship between the photon flux of the lamp and the applied current was found using the average of 3 measurements per data point.

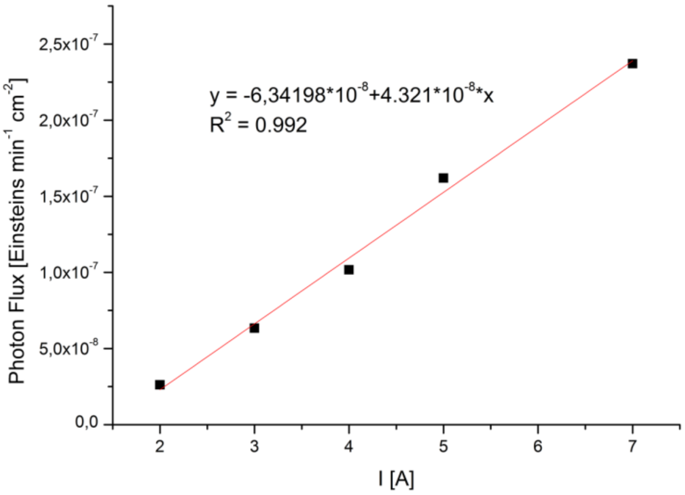

Supplementary Figure 37. Plot of photon flux determined by ferrioxalate actinometry *vs*. applied current.

Photon flux determined by actinometry of potassium ferrioxalate *vs*. current of Hg(Xe) lamp.

The Quantum yield of the photochemical conversion of **1** under CO_2 atmosphere was determined using the following experimental procedure:

10 mg **1** were dissolved in 5 mL of THF. An aliquot of the stock solution was filled in a J-Young nmr tube and a capillary containing a solution of PPh_3 in toluene was added as internal standard. A $^{31}P\{^1H\}$ NMR was recorded. The remaining solution of **1** was degassed 3 times and put under 1 atm of CO_2 (≥99.9993% purity, purification by passing through P_4O_{10}, Drierite and cooling to -40°C). An Argon filled UV/Vis cuvette was purged with CO_2 for 5 minutes and 2 mL of the CO_2 saturated **1** solution was added giving 2 cm² surface area of photolysis. A UV/Vis spectrum was recorded to determine the absorption at 410 nm (A = 1.617). The cuvette was photolysed with 410 nm wavelength using a bandpass (FWHM = 10±2 nm) filter for 3 hours with an applied current of 7 A and therefore a photon flux of 4.782×10^{-7} mol min^{-1}. Afterwards an aliquot of the photolyzed solution was taken and a $^{31}P\{^1H\}$ NMR spectrum containing the PPh_3 solution as internal standard was measured to determine the conversion of **1** by comparison with the spectrum recorded before photolysis. 46% conversion (2.456×10^{-8} mol/min) could be observed giving a quantum yield of 5.3% by the following equations:

$$I_{Abs} = I\,(1\text{-}10^{-A}) = 4.666 \cdot 10^{-7} \text{ mol/min} \qquad (19)$$

$$\Phi = [\mathit{conversion\ of\ }\mathbf{1}] \,/\, I_{Abs} = 0.0526 \qquad (20)$$

Structure optimizations and free energies:

All calculations were performed within the ORCA program package.*(13-14)* The molecular structures were optimized using the PBE*(15)* functional, Grimme's dispersion correction with Becke-Johnson damping (D3(BJ)) *(16-17)* and the Resolution of Identity (RI-*J*) *(18-21)* approach to minimize computational costs and Ahlrichs' revised all-electron def2-SVP basis set and the corresponding def2/J auxiliary basis set.*(21-24)* Tight convergence criteria in the SCF procedure and optimization and a fine integration grid (Grid 5) were applied throughout. No symmetry restrains were imposed and the optimized structures were defined as minima (no negative eigenvalue) and transition states (one negative eigenvalue) by vibrational analyses at the D3(BJ)-RI-*J*-PBE/def2-SVP level of theory. All transition states were verified by distortion of the structures along the reaction mode followed by full optimizations. The energies of the optimized structures were evaluated by means of single point calculations applying the TPSS functional*(25)* (see next section for further details), Becke-Johnson damping and Ahlrichs' all electron def2-TZVP basis. Solvent effects have been accounted for by the conductor-like screening model (COSMO, ε = 7.25 for THF) *(26)* using the outlying charge corrected values.

According to Grimme's quasi-RRHO approach, low energy frequencies below 35 cm^{-1} have been treated as free rotors instead of harmonic vibrations in the calculation of the vibrational entropy.*(27)* As ideal gas standard conditions (1atm, 298.15 K) are applied in the calculation of the free energies by gas statistical mechanics, the free energies have been corrected to standard solution conditions (1 mol/l, 298.15 K) by:

$$G_{sol} = G_{gas} + RT\ln\frac{RT}{p} \qquad (21)$$

$$G_{sol} = G_{gas} + RT\ln(24.47) \qquad (22)$$

$$G_{sol} = G_{gas} + 1.89\ \text{kcal/mol} \qquad (23)$$

Therefore, a correction of 1.89 kcal/mol results which has been added in the steps that involve coordination or release of CO_2. The G value of the THF molecule has been further corrected by

applying the actual concentration in the pure solvent which is (ρ = 0.889 g/ml (25°C), c = 12.3 mol/l):

$$G'_{sol} = G_{sol} + RT\ln c \qquad (24)$$

$$G'_{sol} = G_{solv} + 1.49\ \text{kcal/mol} \qquad (25)$$

That gives an overall correction factor of 3.38 kcal/mol for THF.

The total free energies have thus been obtained by addition of the single point SCF energies with the free energy contributions from the thermal analyses at the D3(BJ)-RI-*J*-PBE/def2-SVP level and the above mentioned thermal corrections.

DLPNO-CCSD(T) benchmark studies:

To assess the quality of the DFT calculations, domain-based local pair natural orbital based coupled cluster (DLPNO-CCSD(T)) single point calculations *(28-32)* have been performed for selected molecules employing a truncated model system in which the *tert*-butyl groups of the pincer ligand have been replaced by methyl groups. The model complexes were fully optimized on the D3(BJ)-RI-*J*-PBE/def2-TZVP level and resemble strongly the evaluated structures of the full system. In the DLPNO-CCSD(T) calculations, we applied the all-electron correlation consistent cc-pVTZ and cc-pVQZ basis sets *(33-36)* in combination with the cc-pVTZ/C and cc-pVQZ/C density fitting basis sets *(37-39)* and tight SCF convergence criteria. The complete basis set (CBS) limit has been estimated for the Hartree-Fock energy from the SCF energy of the cc-pVQZ basis (E_{SCF}^{QZ}) by *(40)*:

$$E_{SCF}^{CBS} = E_{SCF}^{QZ} - Ae^{-\alpha\sqrt{4}} \qquad (26)$$

Where α is 5.46 *(41)* and A has been extrapolated from the SCF energies obtained with the cc-pVTZ (E_{SCF}^{TZ}) and cc-pVQZ bases:

$$A = \frac{E_{SCF}^{QZ} - E_{SCF}^{TZ}}{e^{-\alpha\sqrt{4}} - e^{-\alpha\sqrt{3}}} \qquad (27)$$

3 and 4 represent the cardinal numbers of the basis sets cc-pVTZ and cc-pVQZ, respectively. The correlation energy has been extrapolated to the complete basis set limit by *(42)*:

$$E_{corr}^{CBS} = \frac{4^{\beta} E_{corr}^{QZ} - 3^{\beta} E_{corr}^{TZ}}{4^{\beta} - 3^{\beta}} \tag{28}$$

applying a value of 3.05 for the exponent β.*(41)* The resulting ΔE values have been compared with the single point energies of several DFT functionals at the D3(BJ)-Func/def2-TZVP level (without solvent correction).

EPR and TD-DFT-calculations:

EPR data were evaluated applying the PBE0 *(43)* functional including spin-orbit coupling within the Breit-Pauli approximation as implemented in ORCA (SOMF(1X) keyword) using the geometry from the optimization as described above. Relativistic effects were treated at the all-electron level with the ZORA method *(44)* in conjunction with the corresponding recontracted def2-TZVPP basis sets *(45)* employing the model potential approach of van Wüllen.*(46)* Picture change effects *(47)* were also included as a correction within the scalar relativistic framework. The density-fitting procedure was used within the RIJCOSX approximation *(48)* in conjunction with the corresponding def2-Coulomb fit basis sets *(49)* and large integration grids (Grid6 and GridX6 in ORCA convention). Grimme's dispersion correction with Becke-Johnson damping (D3(BJ)) was employed as well.

The UV/Vis spectra of selected compounds were obtained at the same level but employing the B3LYP functional *(50)* within the time-dependent DFT framework as implemented in ORCA. In contrast to the EPR calculations, spin-orbit coupling has been neglected but solvent effects were included by application of the COSMO model (THF, ε= 7.25, n= 1.407).

It is well known that the PBE0 and the B3LYP functionals perform well for the calculation of EPR and UV/Vis data, respectively *(51)*. Indeed, we found that the results are in good agreement with the experimental g-values of [Ni(PNP)] (**7**) and the experimental UV/Vis spectrum of [NiH(PNP)] (**1**) (see below).

DLPNO-CCSD(T) benchmark studies:

The formation energies of the complexes [Ni(CO_2H)(PNP^{Me})] (**3Me**), [Ni(O_2CH)(PNP^{Me})] (**2Me**) and the proposed intermediate [Ni^0(PN^{H}P^{Me})] (**AMe**) from [NiH(PNP^{Me})] (**1Me**) have been chosen as benchmark tests (Supplementary Figure 38):

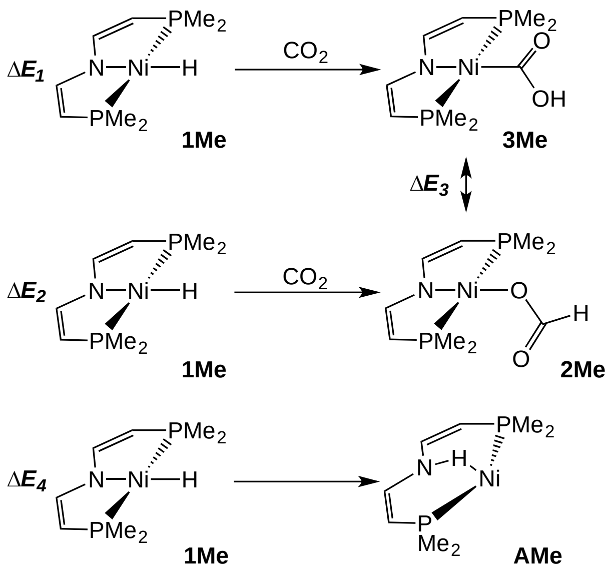

Supplementary Figure 38. Complexes chosen as benchmark tests for formation energies.

Supplementary Table 1. SCF and correlation energies of the DLPNO-CCSD(T) calculations, the CBS extrapolation values and the resulting reaction energies ΔE_1 - ΔE_4.

	E_{SCF} (H)	E_{corr} (H)	ΔE (kcal/mol)
**CO_2**			
cc-pVTZ	-187.7042751	-0.620633567	
cc-pVQZ	-187.7185582	-0.663322647	
CBS	-187.7228618	-0.693712554	
**[Ni^{II}(PNP^{Me})H]**			
cc-pVTZ	-2555.730922	-2.890950518	
cc-pVQZ	-2555.767913	-3.104108016	
CBS	-2555.7790580	-3.255852597	

**[Ni^{II}(PNP^{Me})(COOH)]**			**ΔE_1: -5.63**
cc-pVTZ	-2743.435691	-3.517035835	
cc-pVQZ	-2743.486522	-3.774984175	
CBS	-2743.5018381	-3.958614886	
**[Ni^{II}(PNP^{Me})(OCHO)]**			**ΔE_2: -8.55**<br>**ΔE_3: -2.93**
cc-pVTZ	-2743.484466	-3.475184964	
cc-pVQZ	-2743.535468	-3.731684268	
CBS	-2743.5508347	-3.914283425	
**[Ni^0(PN^{H}P^{Me})]**			**ΔE_4: 30.18**
cc-pVTZ	-2555.617757	-2.953192804	
cc-pVQZ	-2555.653925	-3.168623954	
CBS	-2555.6648222	-3.321987124	

Supplementary Table 2. Deviation of the at DFT level calculated energy differences from the corresponding DLPNO-CCSD(T) values (ΔE^{DFT} - $\Delta E^{CCSD(T)}$) in kcal/mol.

	PBE	PBE0	TPSS	TPSSh	M06L	M06
ΔΔE_1	-3.28	-4.38	-1.49	-1.94	7.82	4.36
ΔΔE_2	-0.98	-5.94	-2.63	-4.21	1.66	1.66
ΔΔE_3	2.31	-1.55	-1.14	-2.27	-6.17	-2.70
ΔΔE_4	-5.57	-0.57	-0.43	1.04	-0.89	-6.57

Computed electronic absorption spectra of selected compounds:

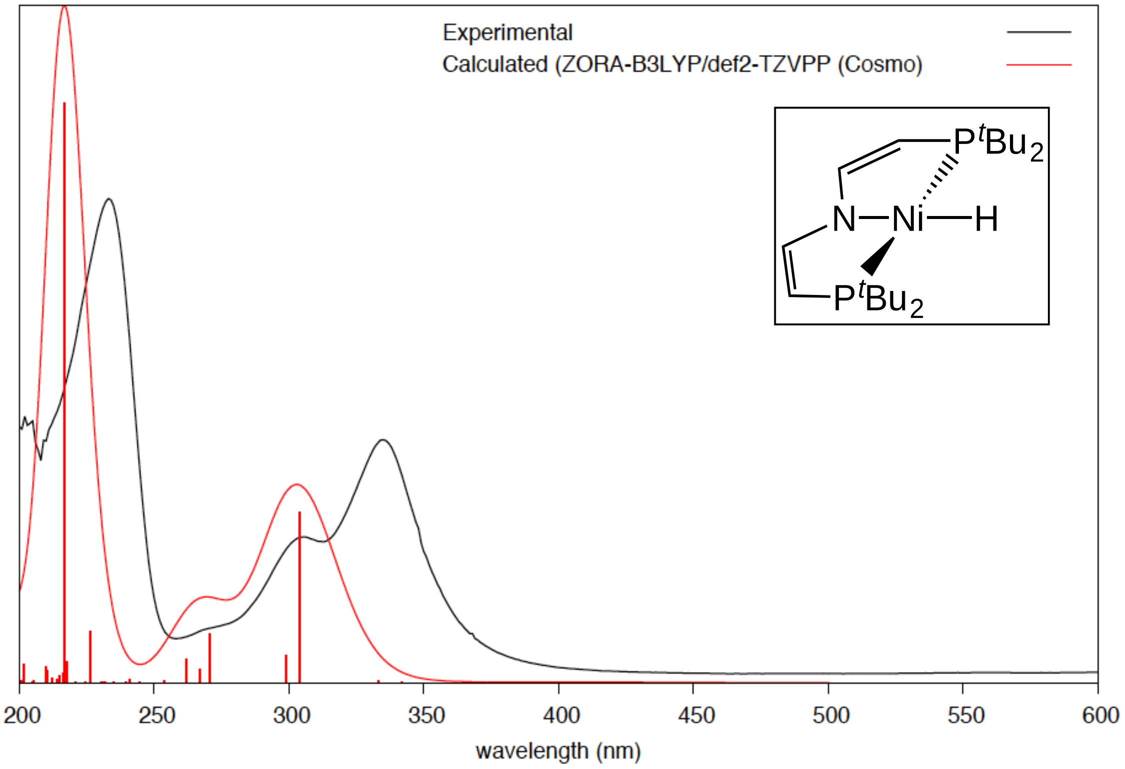

Supplementary Figure 39. Calculated *vs*. experimental UV/vis-spectrum of [NiH(PNP)] (**1**) in THF.

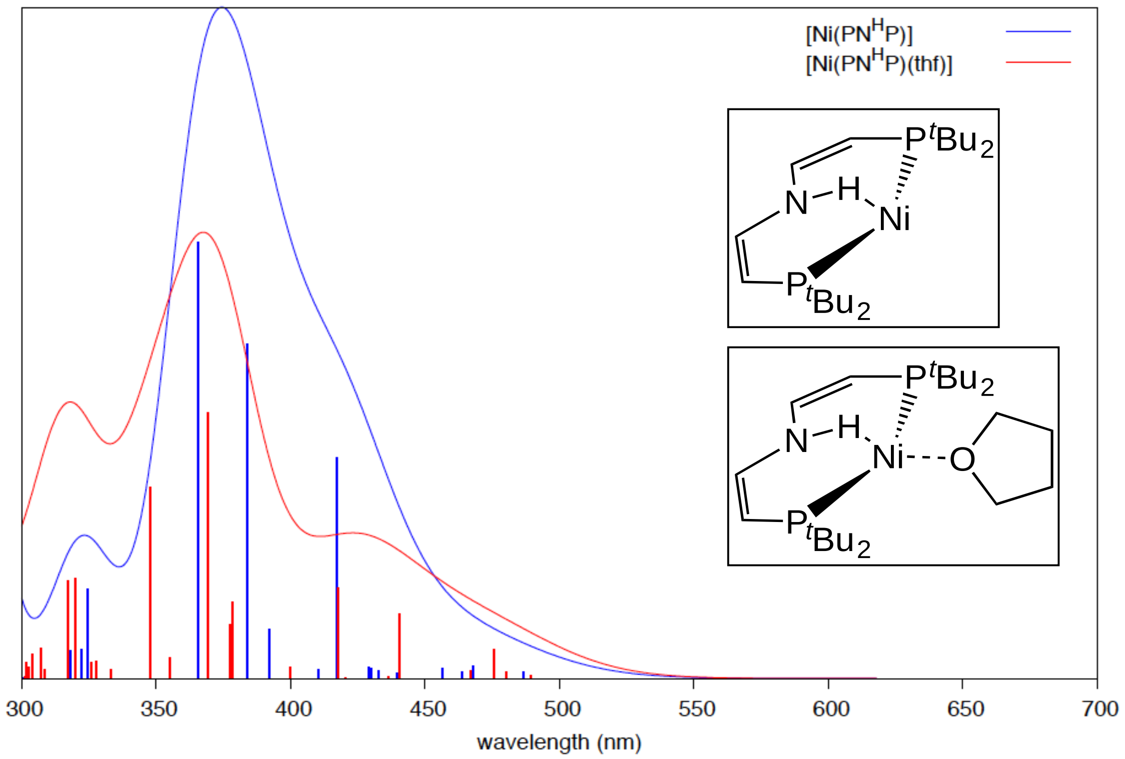

Supplementary Figure 40. Calculated UV/vis-spectrum of [Ni(PN^{H}P)] (**A**) and its THF adduct in THF.

Calculated EPR data of [Ni(CO){N(CHCHP*t*Bu$_2$)$_2$}] (**5**):

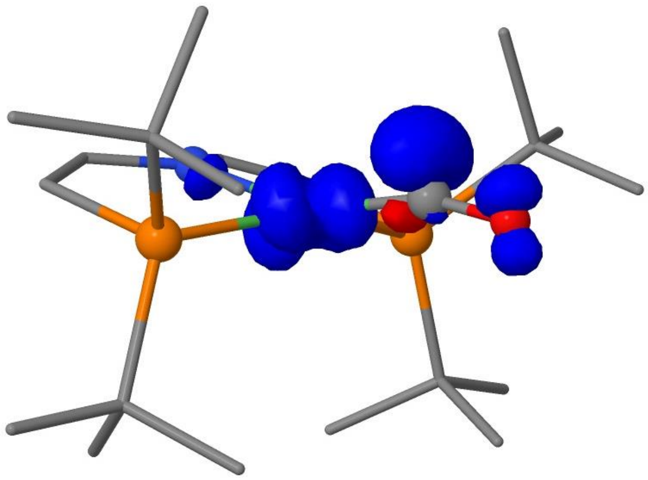

Supplementary Figure 41. Spin density plot of [Ni(CO){N(CHCHP*t*Bu$_2$)$_2$}] (**5**) (Ni 63%, P 4% each, N 3%, C$_{CO}$ 12%, O$_{CO}$ 5%).

Supplementary Table 3. Calculated EPR data of [Ni(CO){N(CHCHP*t*Bu$_2$)$_2$}] (**5**).

g-tensor	g_1 = 2.03	g_2 = 2.03	g_3 = 2.06	g_{iso} = 2.04
A-tensor (^{14}N)	A_1 = 16 MHz	A_2 = 16 MHz	A_3 = 21 MHz	A_{iso} = 17 MHz
A-tensor (^{31}P)	A_1 = 159 MHz	A_2 = 174 MHz	A_3 = 217 MHz	A_{iso} = 184 MHz
A-tensor ($^{13}C_{CO}$)	A_1 = 78 MHz	A_2 = 84 MHz	A_3 = 117 MHz	A_{iso} = 93 MHz

Calculated EPR data of [Ni{N(CHCHP*t*Bu$_2$)$_2$}] (**7**):

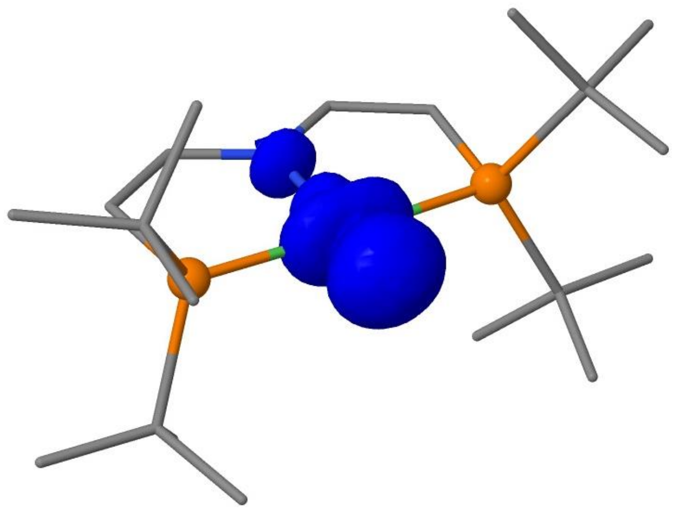

Supplementary Figure 42. Spin density plot of [Ni{N(CHCHPtBu$_2$)$_2$}] (**7**) (Ni 91%, N 4%, P 1% each).

Supplementary Table 4. Calculated EPR data of [Ni{N(CHCHP*t*Bu$_2$)$_2$}] (**7**).

g-tensor	g_1 = 2.01	g_2 = 2.19	g_3 = 2.23	
A-tensor (^{14}N)	A_1 = 25 MHz	A_2 = 26 MHz	A_3 = 32 MHz	A_{iso} = 27 MHz
A-tensor (^{31}P)	A_1 = -5 MHz	A_2 = -17 MHz	A_3 = -29 MHz	A_{iso} = -17 MHz

Relaxed surface scans of the thermal Ni-H *trans*-elimination step:

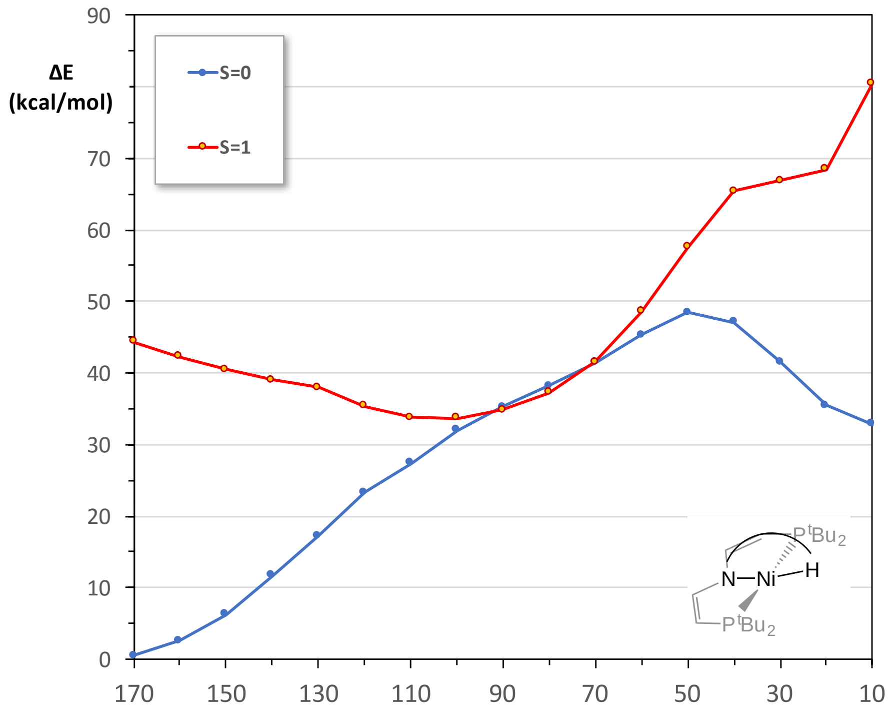

Supplementary Figure 43. Relaxed surface scans of the N-Ni-H angle in [Ni^{II}(PNP^{tBu})H] at the singlet and the triplet hyper surface (D3(BJ)-RI-*J*-PBE/def2-SVP, optimization followed by D3(BJ)-TPSS/def2-TZVP (Cosmo: THF) single point calculations. The scans predict the *trans*-elimination to occur at the singlet hyper surface.

Structures and XYZ coordinates of calculated compounds:

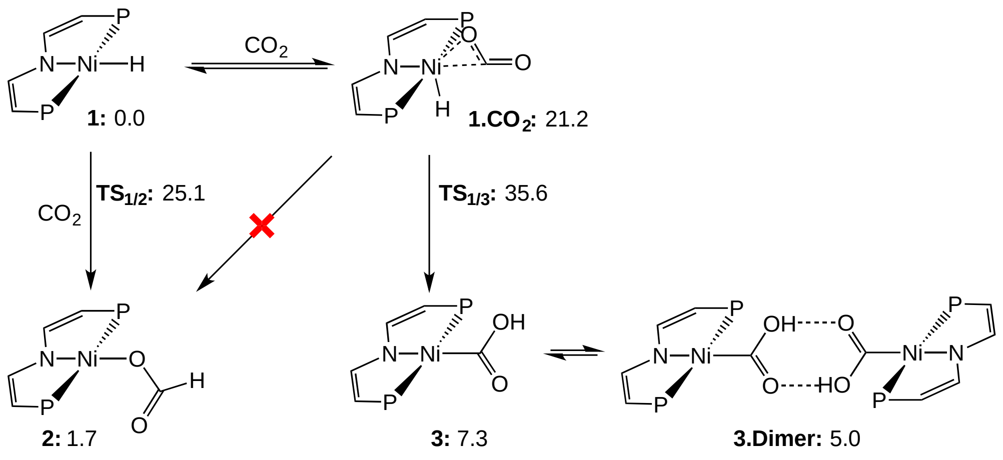

Supplementary Figure 44. Thermal pathway for normal and abnormal CO_2 inversion into the Ni-H bond of **1**.

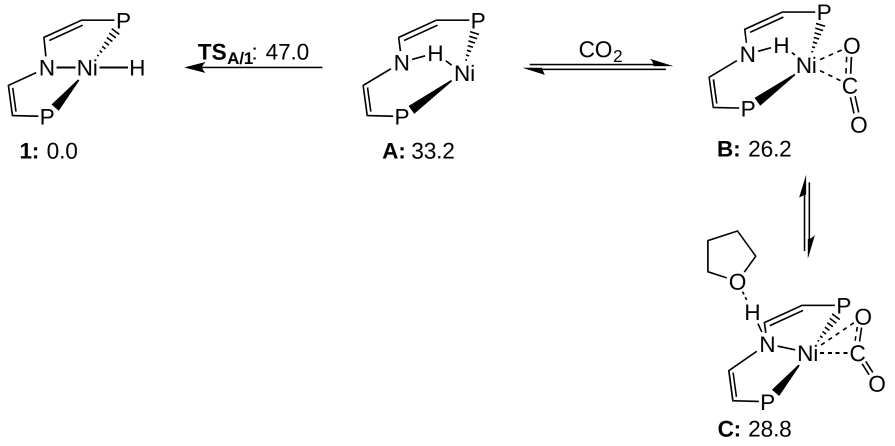

Supplementary Figure 45. Photochemical pathway for normal and abnormal CO_2 inversion into the Ni-H bond of **1**.

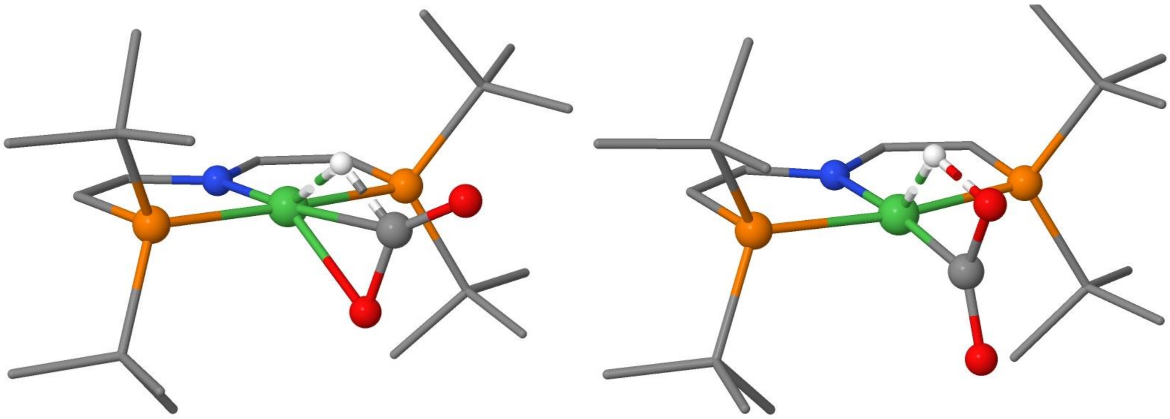

Supplementary Figure 46. Structure of $\mathbf{TS_{1/2}}$ (left side) and $\mathbf{TS_{1/3}}$ (right side). All hydrogens except the migrating one are omitted for clarity.

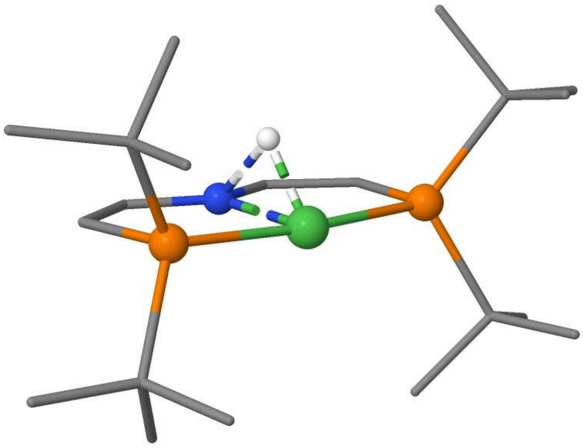

Supplementary Figure 47. Structure of $\mathbf{TS_{A/1}}$. All hydrogens except the migrating one are omitted for clarity.

Supplementary Table 5. XYZ coordinates of calculated CO_2.

CO_2

C	-0.87905240001302	0.29925186565008	-0.00000000421998
O	0.29527088310737	0.29925187217496	0.00000000210999
O	-2.05337568309434	0.29925187217496	0.00000000210999

Supplementary Table 6. XYZ coordinates of calculated [NiH{N(CHCHP*t*Bu$_2$)$_2$}] (**1**).

Ni	0.02809968245013	-0.14587166578865	0.40637692538800
C	3.11430145721658	-0.80126537168452	1.81653795558187
P	2.19333854537938	-0.01489289088503	0.34891884946977
N	0.02955348258105	1.80201017342828	0.50328740901957
P	-2.13701551918039	-0.01192150898513	0.35320148023033
C	-3.05615342004375	-0.79659894518311	1.82284093634148
C	2.92595215108416	-0.50963833391363	-1.34138927964244
C	-2.87347139588730	-0.50631182921490	-1.33551188334129
H	0.02707349223810	-1.65238985940330	0.42205175759088
C	-2.36201675087442	1.77501615736620	0.51996257584470
H	-3.32881103064259	2.29673216386757	0.55475596615833
C	-1.16833589404682	2.46034981247719	0.56244571177445
H	-1.14334635089205	3.56620349861313	0.64737152108571
C	1.22845510941972	2.45867703393261	0.56035402945764
H	1.20515692313300	3.56455901033012	0.64538996067088
C	2.42110388537818	1.77168131276383	0.51572074557192
H	3.38869183681261	2.29202919691486	0.54880571027260
C	4.48650317080292	-0.15819805379692	2.06879083324761
H	4.93900748362790	-0.60274053567040	2.98142712317502
H	5.19359170327244	-0.32070187329790	1.23365407667262
H	4.39477346433084	0.93247559152291	2.23991163430724
C	3.24437853343582	-2.31936997955791	1.62945291887061
H	2.26870912098846	-2.77628854181619	1.36715365904129
H	3.98143521544848	-2.58478942335967	0.84603668267798
H	3.59297695359636	-2.77960513784425	2.57888839097848
C	2.19031639323884	-0.51714418280585	3.01764146237212
H	2.66399233174863	-0.88815991884933	3.95203764153048
H	1.99721343772834	0.56876014036490	3.13287333852680
H	1.21452373317543	-1.02711965782485	2.89015913263135
C	4.45234248324383	-0.36590991610845	-1.40484815640486
H	4.96872967147777	-1.10584351744876	-0.76238026672146
H	4.79897783239378	-0.53704511137057	-2.44710446539793
H	4.78408543716334	0.64921886931614	-1.10658176223104
C	2.48998437456998	-1.94333100617105	-1.68945556567302
H	1.39329206343244	-2.05245230737838	-1.57427765824953
H	2.76241453624515	-2.16940522065267	-2.74269754012096
H	2.97364514985954	-2.70305194581500	-1.04782760511870
C	2.27089444704555	0.46046588344456	-2.34406717197929
H	2.57476663429360	1.50889178311915	-2.15915962369173
H	2.56796984837767	0.18006509169853	-3.37703729508937
H	1.16403899529247	0.41608475038867	-2.27739984549082

C	-2.44177937547305	-1.94149031872253	-1.68279160442546
H	-1.34520516433821	-2.05337127320331	-1.56913328904121
H	-2.92647225832516	-2.69928473558963	-1.03963782993647
H	-2.71639675241098	-2.16791911570967	-2.73538693511202
C	-4.39957458515670	-0.35866687195162	-1.39687428090662
H	-4.74816181537437	-0.52955704311397	-2.43852018060888
H	-4.91694937509189	-1.09686150992523	-0.75320308660663
H	-4.72828132253094	0.65750160240518	-1.09877843806811
C	-2.21736289165175	0.46112065892374	-2.34010062880656
H	-2.51848351680661	1.51046978328713	-2.15596089458806
H	-1.11053244367650	0.41415446147742	-2.27477665816721
H	-2.51642170677906	0.18029316707772	-3.37238212919356
C	-3.18828032929483	-2.31460794413977	1.63636755703571
H	-2.21362351886713	-2.77261843676643	1.37220813715011
H	-3.53551243678360	-2.77427320798529	2.58657759650305
H	-3.92716135705448	-2.57937907552857	0.85444270179374
C	-4.42713844227952	-0.15189755584188	2.07751320992339
H	-5.13610175119867	-0.31417792377198	1.24393627262678
H	-4.87823461469332	-0.59539961094781	2.99135206902365
H	-4.33389844108263	0.93878178340481	2.24779610462864
C	-2.12957743783066	-0.51316782017040	3.02210603915355
H	-1.15448175586451	-1.02404065838217	2.89288304590494
H	-1.93525565145252	0.57257306012175	3.13680165520544
H	-2.60180547489807	-0.88361994966969	3.95745755717450

Supplementary Table 7. XYZ coordinates of calculated $NiH(CO_2)\{N(CHCHP\textit{t}Bu_2)_2\}]$ (**1.CO$_2$**).

Ni	0.03051042835581	-0.97026603037993	-0.18850267313338
C	2.83264941543225	-2.82001070841375	0.82927992672183
P	2.23380541738194	-1.11826433113662	0.19813728217130
N	0.04506206803705	0.45866061102970	1.23636525495860
P	-2.19200233073432	-1.02801251144156	0.16445617228404
C	-2.76767909685779	-2.64535863297979	1.01271743438005
C	3.37719916421850	-0.40793534712833	-1.17016101232439
C	-3.35701644956669	-0.50825639877884	-1.26196009779462
H	0.03134081873768	-2.24957737594520	0.46810577936956
C	-2.33724338590695	0.29379127528758	1.37916705073184
H	-3.29345191459141	0.61228860108280	1.81758152872784
C	-1.14518264732359	0.84645302694885	1.77900842402520
H	-1.11971589665882	1.60495489938294	2.58967676218434
C	1.21756595102415	0.70740375615037	1.88547009079848
H	1.19925563599835	1.43358061574980	2.72535269434533
C	2.38255107911075	0.05919796131457	1.55126710480055
H	3.32937447198979	0.27470711291746	2.06624504653453
C	4.34490000568603	-2.85955066811515	1.09974546126689
H	4.59404722704670	-3.81391185054940	1.61077724480982
H	4.93815034748870	-2.82651644903888	0.16651602184494
H	4.67669794621162	-2.03369259529149	1.76038390449811
C	2.43897768025606	-3.90662494965285	-0.18595820061525
H	1.35875669143290	-3.87528577006420	-0.42717224865260

H	2.98740257364522	-3.80948575460165	-1.14139098297899
H	2.67582601868909	-4.90557881849960	0.23793999652014
C	2.08974676791015	-3.06696738448117	2.15330597253714
H	2.37064225846484	-4.06693304744487	2.54560595737544
H	2.33628748864137	-2.30327262265166	2.91663613240784
H	0.99161825736167	-3.05958226220431	2.00791507431520
C	4.77149104313537	-0.05514830880101	-0.62009733667651
H	5.36812181268229	-0.93807146414817	-0.33149681655130
H	5.33352261918460	0.47739657942877	-1.41694377173598
H	4.70774249280470	0.62926603920821	0.24834186454173
C	3.50232301349682	-1.37795754416531	-2.35538721996843
H	2.51930262262227	-1.70572746270425	-2.74359955043638
H	4.03672733393093	-0.86775988759849	-3.18493334602142
H	4.09138260016605	-2.27898407630446	-2.09366740574356
C	2.70324329610060	0.90497322572956	-1.61821898806582
H	2.55632405442089	1.59150107954313	-0.76043734974944
H	3.36774874714630	1.41176872239074	-2.35001432275498
H	1.72746682859724	0.72140844280184	-2.10048864613722
C	-3.48836925098223	-1.63178947908761	-2.30293774717054
H	-2.50806664827225	-2.02034808836780	-2.64034182274231
H	-4.08221926411158	-2.48430732535335	-1.91741680154920
H	-4.02112461035130	-1.23932895576439	-3.19504636110738
C	-4.74755653790412	-0.09860067924659	-0.74148167392450
H	-5.33749509002171	0.29000770872987	-1.59911361794725
H	-5.31802783958308	-0.93511291904698	-0.30183262630433
H	-4.68224255859092	0.71722248334864	0.00470127333916
C	-2.70595555712170	0.73883984319116	-1.89336404459507
H	-2.53422175836100	1.52775309936759	-1.13334132400473
H	-1.74265765281963	0.50119073140687	-2.37790962477490
H	-3.39634443274013	1.14948025764742	-2.66056502236511
C	-2.32556982143851	-3.84628957051393	0.15937756312024
H	-1.23756033892443	-3.82290126353016	-0.04561353638882
H	-2.56081474296321	-4.78709158132093	0.70102977166458
H	-2.83968232722844	-3.88202347100081	-0.81931382519135
C	-4.28123869905192	-2.70149950209873	1.26801992368666
H	-4.86166898904308	-2.81120323403419	0.33229447830083
H	-4.50605760186523	-3.58903949462076	1.89741714342155
H	-4.64808639925765	-1.80862849985442	1.81262188390121
C	-2.03766782406716	-2.67505257436815	2.36740296032268
H	-0.94613109143767	-2.53415847511500	2.23953336948887
H	-2.40001391796800	-1.87842701048378	3.04530180307105
H	-2.20580115343824	-3.65950105716444	2.85271268660267
C	0.01410891088605	-1.76331770321531	-1.93932525246257
O	0.06591347277370	-0.57381514016439	-2.33007970409463
O	-0.03261609188576	-2.89822941578515	-2.36469431510731

Supplementary Table 8. XYZ coordinates of calculated $\mathbf{TS_{1/2}}$.

Ni	0.00663136343294	-0.11023873410191	0.13430786496576
C	3.17325425375183	-0.99627647005632	1.46483052533750
P	2.25020155912111	0.01047438874800	0.12576488727613

N	0.03403453047410	1.73330615094893	0.58657724447416
P	-2.21597600025955	0.05921888805955	0.20386575434513
C	-3.10751713432711	-0.94678566687205	1.55956119519654
C	3.04639149764665	-0.11463211044105	-1.60519044385250
C	-3.05431132539882	-0.04402148003821	-1.51076446743945
H	-0.15455423486295	-1.81773337179106	0.70395865879432
C	-2.35796132977512	1.78320009087347	0.69452975660091
H	-3.30083981364244	2.31601896076515	0.87495338665687
C	-1.14272910686409	2.40215032616769	0.80930054458708
H	-1.05758423239169	3.46947973124672	1.09251757228776
C	1.23221669561946	2.37840155688296	0.76102580918317
H	1.17936288731872	3.44940230591823	1.03822868538866
C	2.42907116803523	1.73417344871569	0.60949239582868
H	3.38676953468955	2.25487855768969	0.73925283822169
C	4.41821812042459	-0.23490563531957	1.95268572376010
H	4.91991574912413	-0.83736256293961	2.73988935040414
H	5.15563359149018	-0.06291992218172	1.14542235688227
H	4.15216816922864	0.74363037239300	2.39749665952031
C	3.56362274576724	-2.39962592805036	0.97828649568632
H	2.70203424186550	-2.95935175955730	0.56450530293403
H	4.37171834480530	-2.36579156818806	0.22175238783240
H	3.95081895569896	-2.97839140640800	1.84405447972652
C	2.16691946258098	-1.11445978353360	2.62723243141090
H	2.67500221825458	-1.56320252052507	3.50744557319612
H	1.77035299456639	-0.12316555027375	2.92940394698063
H	1.31385045433593	-1.76721774975687	2.35737401413954
C	4.53149804337729	0.27738423131250	-1.55493380375644
H	5.14189647529284	-0.47078365493708	-1.01227877990389
H	4.92603748757822	0.33141506308053	-2.59240897716964
H	4.69004557771307	1.27007400897860	-1.08781576949821
C	2.87793218859233	-1.51290683470732	-2.22468087381584
H	1.81315273281859	-1.80914286132410	-2.28626684528766
H	3.29210683131153	-1.48912622785512	-3.25559853233334
H	3.42446823132819	-2.29537289292985	-1.66851814915103
C	2.26246597870512	0.90334248741008	-2.45721523261893
H	2.37088037139844	1.93666968667123	-2.07335277935706
H	2.64467534117361	0.87437728894131	-3.49943365952904
H	1.18225510583171	0.65582806821599	-2.48216983704074
C	-3.37570505853178	-1.49159778632014	-1.90986782342051
H	-2.48026447925701	-2.13780827529354	-1.83859896978953
H	-4.19464433440475	-1.91954354466978	-1.29885641542418
H	-3.71817668796785	-1.50124573714522	-2.96680404954055
C	-4.32456418569197	0.82221335219560	-1.55619026154330
H	-4.74745456672491	0.77627888857788	-2.58257239077423
H	-5.10931647745255	0.47284413080324	-0.85899217511562
H	-4.10377946811925	1.88410987999473	-1.33269655808095
C	-2.00800978335060	0.54170427999900	-2.48037205680131
H	-1.67271871441215	1.54910248823413	-2.15900050836223
H	-1.12798623781099	-0.12429512863275	-2.55481519320927
H	-2.46297093102237	0.64034442157680	-3.48919917169633
C	-2.95960146650702	-2.46287712218881	1.35196642666185

H	-1.90076880277888	-2.78346473825732	1.34647431721629
H	-3.45668790745087	-2.98595162326482	2.19636844826575
H	-3.43002567656361	-2.81785588602285	0.41740028197345
C	-4.59436396036696	-0.56527645897395	1.63963050449356
H	-5.16196198756710	-0.91542649281193	0.75554915515926
H	-5.04625961458320	-1.04750970736701	2.53291165687812
H	-4.74177859810835	0.52817531220824	1.74372537559961
C	-2.39286158827621	-0.55202962307955	2.86681794681305
H	-1.31241131728101	-0.80003164560882	2.82380574944274
H	-2.48540060859838	0.53147671827213	3.07719927645583
H	-2.84093930534832	-1.11525453583585	3.71254960310724
C	0.08614456004678	-2.50701254467941	-0.28760442181394
O	-0.17455520397008	-1.93313226010648	-1.38201499137663
O	0.52114051626832	-3.61056577283484	0.01862447401792

Supplementary Table 9. XYZ coordinates of calculated **TS$_{1/3}$**.

Ni	0.02182316862817	-0.16926573457393	0.61555282908539
C	3.15622485047868	-1.53269933611623	1.62761774758944
P	2.26224235933706	-0.07505243038200	0.78208768598178
N	0.01061216002558	1.37192303238355	1.85811466213702
P	-2.22048429243903	-0.10453487988107	0.77894713800499
C	-3.09582919476175	-1.57291398951240	1.62492286642319
C	3.11539116457824	0.55260609485063	-0.80788548869081
C	-3.08092423952959	0.51113987016914	-0.81169125013161
H	0.03936659079940	-1.65527631118427	0.92471805564079
C	-2.37470517859694	1.27233387517219	1.93821691769469
H	-3.33596142196905	1.64643504494839	2.31589368106177
C	-1.18589079094393	1.83498427341008	2.32693696821164
H	-1.16609191947207	2.68093188127713	3.04477700182351
C	1.20018497222675	1.85012530817070	2.32908325262574
H	1.16841278682965	2.69561322299588	3.04704037846480
C	2.39685885300467	1.30278134358848	1.94239392213959
H	3.35256592920363	1.68929408862344	2.32161534702605
C	4.59525000559855	-1.15753378311230	2.01623928422732
H	5.03173032146781	-1.98034978989641	2.62194089570258
H	5.24451484611464	-1.02552122113204	1.12909676315066
H	4.64056821103542	-0.23501729362853	2.62806748992152
C	3.15672442833528	-2.79825208268667	0.75352375857855
H	2.14398231034999	-3.05548094432325	0.38872917050704
H	3.82903493341803	-2.70329455602585	-0.11881292854519
H	3.52947774255781	-3.64827324480183	1.36419510163386
C	2.33062556473153	-1.79702092055631	2.90245291983307
H	2.80676089821621	-2.61528943284689	3.48273131592573
H	2.26914238436567	-0.89837734077992	3.54788579313807
H	1.29752689121420	-2.11335585876741	2.65427576245932
C	4.40634048715238	1.32442829335118	-0.49120642055654
H	5.18079429753878	0.68691698913469	-0.02383132782384
H	4.82867799834325	1.71871005484845	-1.44020221030956
H	4.21426756855945	2.18813066047566	0.17456809031935
C	3.40337927883726	-0.58601807621372	-1.79739639033040

H	2.50840112405016	-1.20801149498866	-1.98959625038252
H	3.71416789927255	-0.14341283515505	-2.76792339885841
H	4.23219712793545	-1.23397511617590	-1.45317891336845
C	2.07858278448743	1.51862819425184	-1.41798548070941
H	1.75056151153873	2.28329788793943	-0.68399943848848
H	2.53600664452147	2.04978974173634	-2.27991912266274
H	1.19423834566087	0.96478576676981	-1.78658131412343
C	-3.35288672889730	-0.63238307680231	-1.79997703069655
H	-2.44878580587121	-1.24115456504016	-1.99189217594901
H	-4.17188453783740	-1.29207027157046	-1.45444839917528
H	-3.67077783072453	-0.19549630190746	-2.77079992922833
C	-4.38204103522347	1.26630744707122	-0.49653929468537
H	-4.80903712202927	1.65375156710557	-1.44627641151225
H	-5.14837887543415	0.61964073378108	-0.02842188124522
H	-4.20156501757614	2.13351601569410	0.16795633643993
C	-2.05672189880194	1.49053899749895	-1.42184404100879
H	-1.74027135697897	2.26052509617362	-0.68833345337082
H	-1.16427622728381	0.94870863872306	-1.78875751167108
H	-2.52041773108132	2.01433725067869	-2.28494019936931
C	-3.06849156326761	-2.84143778709322	0.75561427880129
H	-2.04842914198050	-3.08381273077719	0.40120394722267
H	-3.43382638100995	-3.69509050826229	1.36569375246315
H	-3.73366588711620	-2.75981832072908	-0.12352926320105
C	-4.54353731850001	-1.22065507636636	2.00236398448649
H	-5.18953774031065	-1.10679205573609	1.11033542640758
H	-4.96907185627278	-2.04663054827119	2.61156673192805
H	-4.60917742457630	-0.29458479779810	2.60701380920408
C	-2.27412639511718	-1.81788848072966	2.90611113751699
H	-1.23304839703617	-2.11345807983714	2.66587894312013
H	-2.23545585357467	-0.91662130718620	3.54953649946559
H	-2.73767125915020	-2.64436293018417	3.48494564170695
C	0.03172966652579	-1.51576507682236	-0.75539237337824
O	0.02770741600920	-1.16658716799190	-1.93137769184963
O	0.04329239041433	-2.68724864497735	-0.21961168674808

Supplementary Table 10. XYZ coordinates of calculated [Ni(OCOH){N(CHCHP*t*Bu$_2$)$_2$}] (**2**).

Ni	0.02814000775858	-0.20689911724315	0.34419013714083
C	3.10201777548776	-0.84963137079605	1.85206680111836
P	2.26826143403330	-0.03175630551942	0.34543924128492
N	0.03087215885337	1.69757714230192	0.55885986184157
P	-2.21149507750473	-0.02633238265320	0.35303148704996
C	-3.04248125608939	-0.84105640649259	1.86284870797389
C	3.15486872529872	-0.38321417053772	-1.30864407310645
C	-3.10343511006628	-0.37711249407149	-1.29826543737874
C	-2.36574202112403	1.74959184283203	0.59809423681780
H	-3.31227951207615	2.29894733142509	0.68711751289000
C	-1.15385194055878	2.38248424892009	0.66623233340225
H	-1.08097181445791	3.47797438419761	0.81419239194250
C	1.21757941329190	2.37954918507463	0.66320669019903
H	1.14777492597693	3.47514143045819	0.81185654307624

C	2.42765228926433	1.74363594153257	0.59138200896970
H	3.37585566323731	2.29046626992973	0.67817139419211
C	4.59767725015020	-0.51182577775603	1.93590085770298
H	5.00584958697653	-0.89264882416047	2.89708571207897
H	5.17862196564571	-0.98848001273707	1.12205910990004
H	4.78216160866644	0.58095740357282	1.90686348838649
C	2.88700837038869	-2.37274979380390	1.84118154808059
H	1.81365186103083	-2.62297386241031	1.74150887156586
H	3.43464765955860	-2.87087644050916	1.01999319553401
H	3.26373080073816	-2.79553862272881	2.79718391884646
C	2.36199465439037	-0.25443826235126	3.06641640838665
H	2.75860448840892	-0.71145723842589	3.99772119612102
H	2.48983395817061	0.84384405756298	3.13016887311753
H	1.27439166279005	-0.46805092455160	3.01552486981673
C	4.43226448078173	0.46309575719689	-1.44020682005387
H	5.18641179397330	0.22723498805619	-0.66532566337834
H	4.89470732787063	0.25947534891622	-2.42975490713634
H	4.20863875549749	1.54649242079708	-1.39291370115894
C	3.48300052567494	-1.87151033339947	-1.49378522844190
H	2.58562619520190	-2.50956496304251	-1.39001880473955
H	3.87160713263703	-2.02367823665994	-2.52336338731099
H	4.26621707656715	-2.22122334499393	-0.79327980597259
C	2.13424132380915	0.05427254227036	-2.37877355606688
H	1.79459404264094	1.09742919276669	-2.21419520305243
H	2.61207378831025	0.00572872231074	-3.38082535325132
H	1.25469009021066	-0.61913304527378	-2.37815622809520
C	-3.43591986390985	-1.86473907875007	-1.48093917647996
H	-2.54023963077883	-2.50522861097760	-1.37760248676724
H	-4.21895754109710	-2.21128544614550	-0.77866495668883
H	-3.82655275348807	-2.01724283298369	-2.50969451996831
C	-4.37903259216640	0.47233170302198	-1.42714341518452
H	-4.84500578506760	0.26857728894301	-2.41500362202183
H	-5.13141085807626	0.23945900151433	-0.64963269181908
H	-4.15245050876093	1.55520794258215	-1.38193308253555
C	-2.08464238376517	0.05666417472365	-2.37165971529323
H	-1.74134238643019	1.09888685909714	-2.20873880867205
H	-1.20707147136132	-0.61934097777167	-2.37333808955504
H	-2.56561374758555	0.00898772422840	-3.37225261040850
C	-2.83399750839213	-2.36506963368040	1.85122922603848
H	-1.76227287008163	-2.61979350439310	1.74554466447423
H	-3.20691950822803	-2.78588654603588	2.80959124797446
H	-3.38866360144057	-2.86108329069240	1.03349994596720
C	-4.53633764296161	-0.49676078277011	1.95267512949407
H	-5.12253930624135	-0.97044553232519	1.14087907697009
H	-4.94243804696449	-0.87625697489127	2.91525825306492
H	-4.71604563827298	0.59684488836464	1.92485011419205
C	-2.29503522797403	-0.24893539810338	3.07417347144332
H	-1.20891774266382	-0.46910863505124	3.02000943547807
H	-2.41626376623192	0.85014989698158	3.13715692460750
H	-2.69135527485439	-0.70260317449924	4.00724103188108
O	0.02474497028200	-2.12652736542299	0.42813040514383

C	0.02384333189816	-2.87492459975389	-0.63340514514972
H	0.02265776898826	-3.96989774815132	-0.35513516894695
O	0.02442885421059	-2.52847357706203	-1.81713954553144

Supplementary Table 11. XYZ coordinates of calculated [Ni(CO_2H){N(CHCHP*t*Bu_2)$_2$}] (**3**).

Ni	0.02789527602887	-0.26375584000046	0.30332968889305
C	3.11525607107786	-0.85055864107036	1.82894427578300
P	2.25482096450506	-0.09382889394652	0.30167013729009
N	0.02966312225331	1.69502043699367	0.50202323852795
P	-2.19874514681044	-0.09023255691138	0.30611427011884
C	-3.05741287060828	-0.84499590909726	1.83532747636227
C	3.13988056055649	-0.47004299020951	-1.35204938370952
C	-3.08752690728226	-0.46567229669936	-1.34575792629406
C	-2.35792895006566	1.69634351063702	0.51043199582757
H	-3.31525675577057	2.22999656149163	0.57975159235303
C	-1.15927083765541	2.36092795496415	0.58174704478946
H	-1.12292288535453	3.46227423055622	0.70870368576195
C	1.21982056557293	2.35896256168962	0.57977657792124
H	1.18550436069190	3.46034789153996	0.70695576202815
C	2.41726049961643	1.69242268252577	0.50628074061690
H	3.37557855375830	2.22449603121269	0.57403896430311
C	4.44179730355246	-0.14318350383800	2.14502160353560
H	4.86029450426637	-0.56511664103807	3.08384919518442
H	5.19937766102671	-0.29072991994226	1.35084118938684
H	4.30258320238960	0.94446497923357	2.30009723396283
C	3.34158756048882	-2.36405495728892	1.68752890563391
H	2.42460653731659	-2.89611616392811	1.37138718464681
H	4.16208810806314	-2.59991697513555	0.98406053543176
H	3.63466914945169	-2.77135214033223	2.67885691967943
C	2.10518292062090	-0.59454777111914	2.96680377474626
H	2.56429848130848	-0.88689649631481	3.93555554225300
H	1.82124744983417	0.47566495516892	3.03099546211562
H	1.19013771425852	-1.20064769204417	2.82160808492779
C	4.52051134197586	0.20004505110230	-1.42815233304127
H	5.23359192091099	-0.22973702095427	-0.69824892494308
H	4.94937995346664	0.04011600962711	-2.44081457215771
H	4.45992833315683	1.29299065403324	-1.25987450195051
C	3.27334375455023	-1.97872877366221	-1.61616908836299
H	2.33264947647964	-2.52896239410343	-1.42376326883495
H	3.55048635932933	-2.13610994739330	-2.68066544904070
H	4.06877155817410	-2.43778008953790	-1.00087785212563
C	2.21290735755720	0.15843920576011	-2.41115187026068
H	2.04431314653000	1.23704832467104	-2.21947761345239
H	2.66365902119433	0.04455405318373	-3.41990458361604
H	1.21847196378074	-0.33305908138429	-2.42016434275789
C	-3.22359231701681	-1.97426099181993	-1.60912215568942
H	-2.28326254123168	-2.52570516106618	-1.41841737288628
H	-4.01842860478220	-2.43205358200498	-0.99213042105748
H	-3.50303483615059	-2.13161029336081	-2.67302333661456
C	-4.46734500692707	0.20634719184508	-1.41956376501963

H	-4.89815158979265	0.04686108866983	-2.43147221882672
H	-5.17980047431001	-0.22233186217713	-0.68839221438017
H	-4.40495915150521	1.29923911505609	-1.25156999456578
C	-2.16159168629190	0.16108656100264	-2.40679504652844
H	-1.99120400784849	1.23955540553966	-2.21593092617708
H	-1.16784326755091	-0.33177015059739	-2.41725276026441
H	-2.61426735492614	0.04731753495547	-3.41469770289816
C	-3.28599693642459	-2.35825824217958	1.69507791078756
H	-2.37022456307602	-2.89166243419406	1.37769248457183
H	-3.57799359528768	-2.76466069467388	2.68709209068177
H	-4.10795848637018	-2.59338294016464	0.99306147065936
C	-4.38244554109113	-0.13567637416982	2.15336576970645
H	-5.14155787751939	-0.28255021160337	1.36053148759659
H	-4.79992067223210	-0.55662796479746	3.09308860644057
H	-4.24148624313535	0.95184785400413	2.30771947689960
C	-2.04499407161865	-0.58972409838815	2.97125907828496
H	-1.13099368709710	-1.19708913036840	2.82478702574487
H	-1.75953359979371	0.48014588056467	3.03438602354973
H	-2.50277128621604	-0.88095495040507	3.94098004427931
C	0.02646124164739	-2.12412091053618	0.27125478853351
O	0.02517953286526	-2.77439158644104	-0.95765700369648
H	0.02524500110063	-2.06458445963934	-1.63371162540510
O	0.02665690238496	-2.82811432148934	1.27059838474017

Supplementary Table 12. XYZ coordinates of calculated [Ni(CO_2H){N(CHCHP*t*Bu_2)$_2$}]$_2$ (**3.Dimer**).

Ni	-0.03904546005435	-3.83726519501590	0.24404474649917
C	2.65301429334508	-3.25843854131319	-1.91319007993983
P	2.13403012022377	-3.97927427673564	-0.22108431422140
N	-0.04927629711060	-5.78875767263182	0.10377658465183
P	-2.19902832151036	-4.02081883122728	0.74096676398827
C	-3.44409268854510	-3.31767846438032	-0.52468521372485
C	3.33672800982068	-3.53402382260644	1.19366672531790
C	-2.64265343630259	-3.59926817329043	2.55299444255885
C	-2.37901384089922	-5.81311805658230	0.62667013514654
H	-3.32018092866299	-6.35541696619774	0.78896944663196
C	-1.21571166481900	-6.46819178138219	0.30977350165519
H	-1.18892055391089	-7.57271417077982	0.21252226824933
C	1.10741035946872	-6.44575899766297	-0.20527360957208
H	1.06271249110812	-7.55120279799357	-0.28378614948999
C	2.28211088752416	-5.76769333396297	-0.41258929786569
H	3.21570130836926	-6.29285307999676	-0.65530680131947
C	3.82559500569193	-4.04177799739461	-2.52553050764075
H	4.05689180503990	-3.61798372676473	-3.52639651473029
H	4.74720120035298	-3.97728871299728	-1.91569054797877
H	3.57215439363776	-5.11069727374176	-2.66437346444178
C	3.00282111584918	-1.76458413516293	-1.82968301309460
H	2.21500617506668	-1.18119946765308	-1.31806225665544
H	3.97191753795049	-1.59018305357260	-1.32240542533290
H	3.09713388399606	-1.35884674148441	-2.85991776715437

C	1.39851038035547	-3.44803512513075	-2.79158597263545
H	1.64252846865844	-3.17925298719111	-3.84217349104389
H	1.05254692238439	-4.50210324356492	-2.78135339854345
H	0.57369179785367	-2.79555353942113	-2.44525111051262
C	4.77409376866489	-3.97043007243630	0.87358493938124
H	5.21942627387314	-3.36650853500925	0.05874612831398
H	5.40941359649989	-3.82688924619514	1.77418589267013
H	4.83450850581541	-5.04054192764914	0.59205498690385
C	3.29790124653209	-2.03488816289586	1.53171196399241
H	2.27192612503297	-1.69596317153166	1.76554501829327
H	3.92715963387823	-1.85416361190865	2.42972893854858
H	3.69901875447415	-1.40232157992142	0.71944640945195
C	2.80229910597561	-4.32842888887889	2.40178377217920
H	2.81182860940878	-5.41966409680580	2.21152870642844
H	3.43689721834774	-4.11924294604579	3.28914041094280
H	1.76172844447709	-4.03246088902598	2.64519971095279
C	-3.01314896152944	-2.12027470601939	2.73685257409375
H	-2.25104548735861	-1.44701173385975	2.30247816096009
H	-4.00146569035108	-1.87974517813076	2.29903629858271
H	-3.07387854981583	-1.89738649195998	3.82386424236732
C	-3.77543696452626	-4.49717267061019	3.07635359864481
H	-3.97733093422522	-4.24055058792272	4.13856804727273
H	-4.72031627029877	-4.36128243401434	2.51576238656171
H	-3.49725819594796	-5.56799372352511	3.03506002046467
C	-1.34629832567818	-3.90666382004607	3.33057964723912
H	-0.98588975247868	-4.93606556587476	3.12772994417890
H	-0.54601225354479	-3.18998937539791	3.06233456678141
H	-1.54144601842002	-3.82135599399836	4.42153954754446
C	-3.40483764445479	-1.78182364038368	-0.59706956368110
H	-2.38892331800744	-1.41386273234708	-0.82843346297782
H	-4.07707505137893	-1.44704838096772	-1.41630106184039
H	-3.75354199577774	-1.29663324832505	0.33224090012442
C	-4.87370267275113	-3.79882375289746	-0.23437031552681
H	-5.28176433475017	-3.35439843104849	0.69493976647768
H	-5.54013117260161	-3.48743872114894	-1.06753647400744
H	-4.93647550720839	-4.90216912696289	-0.15414137672275
C	-2.95779465969017	-3.88372644880391	-1.87400779232165
H	-1.93065825537016	-3.53447724889416	-2.10298015742647
H	-2.95424396935502	-4.99155383456419	-1.87788958954726
H	-3.63009369281264	-3.52991564896544	-2.68440292290969
C	-0.04778286840758	-1.98852150818251	0.28993444685911
O	0.03451287303685	-1.38918903724328	1.47831400631506
H	0.06418980517744	-0.35060853356748	1.36947887539161
O	-0.13288204474695	-1.30368538324619	-0.76378637215978
Ni	-0.04977341090012	3.69177334895307	0.30502800179233
C	3.11536544500500	3.14659235485125	1.69734545528582
P	2.16631557323671	3.87322500718592	0.20661307852336
N	-0.05903261836691	5.64003100850368	0.46293096963634
P	-2.26944751032277	3.83284877352483	0.35319836011212
C	-3.14672110677331	3.08787301083040	1.87676063195996
C	2.97763455360567	3.46406617205289	-1.47332799987650

C	-3.13330283322628	3.42125636392025	-1.30269441889683
C	-2.44583069561984	5.61954014171563	0.53838209373075
H	-3.40662461458292	6.14491222789748	0.62173788272586
C	-1.25200252749244	6.29608130004831	0.56429165491289
H	-1.22221618588520	7.40002092324150	0.66752431441525
C	1.12645493042665	6.31804901812444	0.48334765763970
H	1.08304797107642	7.42153215344924	0.58633561422181
C	2.32746178824840	5.66237574340169	0.38109708020704
H	3.28257999704092	6.20466906430348	0.39037052461466
C	4.39260171186432	3.94678929205233	2.00154017197909
H	4.88308228976746	3.51178541993272	2.89879852327389
H	5.12621842719175	3.91579753747191	1.17327268365167
H	4.16394123041192	5.00673067220989	2.22569689994891
C	3.45579300136096	1.66180811347271	1.49891508353441
H	2.57035097190747	1.06894939492066	1.20310271065779
H	4.25819705618372	1.51386989506563	0.74974085311246
H	3.82545553646630	1.24448872738887	2.46016927078683
C	2.12725327204872	3.29979189237234	2.87240368457206
H	2.63978541126451	3.02716234052350	3.82030829336327
H	1.77092563198186	4.34625939026632	2.96683330026756
H	1.25296445853944	2.63295839458912	2.74198317668625
C	4.45014778707590	3.89818078886335	-1.51113034538458
H	5.08238166155005	3.27352486646689	-0.84991296125009
H	4.84048132909766	3.78155612617401	-2.54526765771736
H	4.58179154285263	4.96052054929646	-1.22430936220252
C	2.85228959791786	1.97210225828477	-1.82280254370823
H	1.79923938574421	1.63644711894635	-1.79964244510630
H	3.23800951527352	1.81222485837714	-2.85277823521840
H	3.44069534012269	1.32300303124632	-1.14924607516455
C	2.16095873156916	4.28021584470768	-2.49457640670252
H	2.21988626196044	5.36775446830776	-2.29284226462098
H	2.55330154086901	4.08805540460342	-3.51585937875722
H	1.09174650316935	3.98675659198612	-2.47619475054312
C	-3.50224487751512	1.93488445772454	-1.41987666781343
H	-2.65166280381137	1.27635618744636	-1.16386404086555
H	-4.36967179800825	1.66948550184839	-0.78512523043749
H	-3.78835030963914	1.71752809547622	-2.47142131185857
C	-4.38077186743640	4.29387214907363	-1.51393740518625
H	-4.83191695289035	4.04878396964979	-2.49952076767706
H	-5.15457866090103	4.11980920755054	-0.74098237403290
H	-4.12766487685311	5.37178676144032	-1.51681242520666
C	-2.07250803223732	3.77221329983238	-2.36610855440376
H	-1.69563181131548	4.80784720953001	-2.23921453841265
H	-1.21574190446676	3.07264793336285	-2.31168755136640
H	-2.52483251896977	3.69392249886970	-3.37843361613476
C	-3.14651550721386	1.54988930717155	1.87192154159636
H	-2.12935683048139	1.13863573144714	1.74061531510826
H	-3.52447552102614	1.19226999941735	2.85386241587801
H	-3.80531771742031	1.12458004487634	1.09386515816076
C	-4.58475898425908	3.61148301810357	2.00937994418431
H	-5.23835877156707	3.23986389619537	1.19544191285657

H	-5.01732544871257	3.25322939436296	2.96834945644440
H	-4.62796596481791	4.71843487181849	2.01796282852668
C	-2.29049735852595	3.57729599909919	3.06215900403321
H	-1.26240194536487	3.16742527054944	3.00251297041533
H	-2.22272520139611	4.68298932012123	3.09060929611208
H	-2.74627895002527	3.22894276739174	4.01350448194353
C	-0.04909285304394	1.84362133960623	0.26148973282211
O	-0.24656002253063	1.24301863782988	-0.91197295941501
H	-0.19811815720760	0.20400817757800	-0.81142657655855
O	0.11883370138765	1.16132887614683	1.30716575722610

Supplementary Table 13. XYZ coordinates of calculated **TS$_{A/1}$**.

Ni	0.03462925107536	-0.25200153207802	0.27571775173265
C	3.16524934639677	-0.92414007465492	1.84917843047221
P	2.20877292921399	-0.15019153905621	0.39234121114148
N	0.04335736816963	1.83387296450424	0.58698535434604
P	-2.14738108529249	-0.10370504391437	0.28689350742384
C	-3.13653580111247	-0.85977056716990	1.73467643625855
C	3.02193807619454	-0.50928538759389	-1.30310006489380
C	-2.94587185931060	-0.46666144081111	-1.41573107723369
H	0.02443511086937	0.52582706809620	1.47221916668368
C	-2.33808714906594	1.69836767558610	0.50792723955351
H	-3.31492031688055	2.20350777079732	0.54630055696275
C	-1.17952181489246	2.43713610786412	0.60371154082562
H	-1.21684623360281	3.54254905242895	0.70244990465210
C	1.27589268392353	2.41307936795697	0.65052228590017
H	1.33173264724657	3.51734586885318	0.75293585311301
C	2.42312590018956	1.65192130149024	0.59711372620914
H	3.40656827633339	2.13596479518510	0.69615744872423
C	4.67412218070668	-0.64946690499526	1.80304870932441
H	5.15271439579942	-1.02341708720973	2.73423662219877
H	5.16443081186862	-1.16507440989132	0.95378032633601
H	4.89703835114971	0.43396182497365	1.72831526378749
C	2.88530020048965	-2.43605526836629	1.89437648912118
H	1.79478402225115	-2.63533311480868	1.85696750633767
H	3.35883349029039	-2.98058074562468	1.05645858649399
H	3.28730605719520	-2.86073720977862	2.83950030649536
C	2.55205115489750	-0.27685368702410	3.10538286322966
H	3.00971545973737	-0.72320707233792	4.01400441765220
H	2.71835229173731	0.81807255930247	3.12815244326862
H	1.45760619436047	-0.45596955233667	3.14930725643423
C	4.31536122819261	0.27484563260257	-1.55780597140774
H	5.12711630671596	-0.01069186354016	-0.86192085017413
H	4.67606266219640	0.07527339276384	-2.59043332039918
H	4.14780936258779	1.36596487230163	-1.46601503338244
C	3.24621954949788	-2.01731275940419	-1.47671673637015
H	2.33437808707505	-2.58924188797517	-1.20678903217211
H	3.48956272581823	-2.23974208987785	-2.53811453581827
H	4.08880206350911	-2.38875066150103	-0.86073826883379
C	1.93567720410202	-0.04562073792518	-2.29424002619056

H	1.67670327105042	1.02272109893456	-2.14771288350903
H	2.28802997747222	-0.17682073195449	-3.34061296339908
H	1.01014982045502	-0.65059546338237	-2.16933808094456
C	-2.60090110261092	-1.91206848717609	-1.81445445837991
H	-1.51566788649318	-2.10237032576425	-1.67764539586583
H	-3.14872616985859	-2.66040908871961	-1.21164194617379
H	-2.86104713533955	-2.08193005499858	-2.88145332138055
C	-4.45722754875040	-0.22236507286383	-1.47978645572027
H	-4.81137044749373	-0.32693411896606	-2.52874422999402
H	-5.02323019561378	-0.95381273479433	-0.87011304763269
H	-4.72538545073370	0.79828894745622	-1.13980141834910
C	-2.21724139741036	0.49415295339715	-2.37511349390748
H	-2.47388110882601	1.55148282012352	-2.17087260034385
H	-1.11581341384356	0.39640439428929	-2.27306043123499
H	-2.49009728739164	0.25621234792072	-3.42538890125307
C	-3.33065460858128	-2.36618865462661	1.50847894803870
H	-2.37271329050343	-2.85907389007302	1.24351889915644
H	-3.71189371783701	-2.83686628813215	2.44019136587942
H	-4.06730480467486	-2.57846558502157	0.70842446082467
C	-4.48435660510165	-0.16805347582251	1.98719085998562
H	-5.18622144311724	-0.28062958027634	1.13983174183488
H	-4.96814301135661	-0.61613563233432	2.88212592062813
H	-4.35232973659763	0.91327572619125	2.18812748896223
C	-2.22494497271572	-0.64629157074558	2.95897365262113
H	-1.25237977203799	-1.16216496736825	2.82463024730388
H	-2.02027706008216	0.43125006734350	3.12556216962347
H	-2.71840923164059	-1.04875704946749	3.86983388542707

Supplementary Table 14. XYZ coordinates of calculated [Ni{HN(CHCHP*t*$Bu_2)_2$] (**A**).

Ni	0.02386819092766	-0.64904472438021	0.20344860072750
C	3.06232177970429	-0.91029219286979	1.73743623620688
P	2.10728758409440	-0.15377856829421	0.25578278378733
N	0.03113858336631	1.94541897994919	1.18443970990136
P	-2.06207509944329	-0.17828184139048	0.33641733438075
C	-3.01278202810514	-0.91066393267579	1.83289213350983
C	3.00828698514202	-0.45061511564341	-1.40909367445588
C	-2.96285956214769	-0.49373874671290	-1.32665321429264
H	0.03478444250135	0.85770539940885	1.17953373232552
C	-2.26611676090355	1.63166098437710	0.55010034452640
H	-3.24645914600349	2.10228257788777	0.37699069798462
C	-1.20914256287284	2.44405607223407	0.86040062846712
H	-1.31129751617562	3.54595647014961	0.86114326196416
C	1.26811980486924	2.45416725617184	0.85667440102734
H	1.36884904057989	3.55594798437650	0.87987882199184
C	2.32017348853589	1.64847149995615	0.51834932803548
H	3.29833287714659	2.12060660347973	0.33385647129263
C	4.40460637468574	-0.23942328571241	2.05361652691560
H	4.82444660716086	-0.66819359686628	2.98981136680882
H	5.15517486521677	-0.39141283192181	1.25543188041843
H	4.28265694399882	0.85038912054375	2.21360524350295

C	3.23879536179141	-2.41851742557017	1.51028646406047
H	2.27927913679186	-2.89110909340453	1.21292553350239
H	3.99216799028567	-2.63977835040569	0.72816236681422
H	3.58341577341340	-2.90424514292646	2.44881238703441
C	2.08686919020497	-0.68520284583167	2.90975727686050
H	2.46906280107369	-1.17668308446687	3.83059552941428
H	1.95331459618402	0.39495274286569	3.12352802036595
H	1.08669788027965	-1.09979172894934	2.65851718001980
C	4.52791615480507	-0.25915207034900	-1.39001804381447
H	5.03673262891348	-1.02413518535291	-0.77137578463489
H	4.93274905050492	-0.34957052482752	-2.42187844460211
H	4.81782386891100	0.74180247062263	-1.01077914434197
C	2.63216166129870	-1.87401371050838	-1.86060946958955
H	1.53351763360027	-2.01631939637678	-1.77198444744750
H	2.92941392091560	-2.03288310400043	-2.91959522918395
H	3.12116162645372	-2.65593823485443	-1.24912135300078
C	2.35841854580411	0.55125502842153	-2.38172062548293
H	2.62657935211491	1.59853248707457	-2.14199985319912
H	2.68769557593991	0.33264052272218	-3.42041620097909
H	1.25187889164296	0.47143036291163	-2.33992885265081
C	-3.07215006542677	-2.01324365199234	-1.52398825690447
H	-2.10070210831973	-2.50071173505890	-1.29420568312120
H	-3.85169523629260	-2.46725546214865	-0.88098791185573
H	-3.33401308348056	-2.24202869063609	-2.57944412454165
C	-4.32470102007787	0.18617084603386	-1.50499250999365
H	-4.70007196267967	0.00290290570277	-2.53552531688275
H	-5.08761505291241	-0.19911228968963	-0.80287070413209
H	-4.25621014502516	1.28450708847043	-1.37587958877850
C	-1.96037773683420	0.06393655292268	-2.35639927304681
H	-1.83437509723835	1.16051464558205	-2.25212641373153
H	-0.96273881604486	-0.39974933693849	-2.19173378719109
H	-2.30440008879638	-0.15718557790795	-3.39012510580199
C	-2.77626243284961	-2.42983923700029	1.87252073016686
H	-1.69215174274322	-2.65806622084968	1.80198858605141
H	-3.15697718342758	-2.84535217701273	2.83066953923258
H	-3.28951210202973	-2.96203039886439	1.04965112783819
C	-4.51032020971437	-0.58791026131357	1.85887177276261
H	-5.06275875705672	-1.10918797176600	1.05241144477118
H	-4.95089451950445	-0.92006681926341	2.82438641059668
H	-4.70030900664413	0.50037387221272	1.76461989510596
C	-2.31411940662952	-0.27964393983792	3.05212393472427
H	-1.21812410771964	-0.44998145143501	3.00168756297064
H	-2.48531115380260	0.81346116818614	3.10602663686681
H	-2.69697849795745	-0.74268568625690	3.98673011072332

Supplementary Table 15. XYZ coordinates of calculated [Ni(CO_2){HN(CHCHP*t*Bu_2)$_2$}] (**B**).

C	3.07694350914579	-0.93654730382362	1.72970931786411
P	2.17112244220794	-0.21260275405738	0.20331725892809
N	0.03094788572177	1.87904236975474	1.16801438701461
P	-2.13234309110068	-0.22106349015415	0.28741660438745

C	-3.01012863032984	-0.92232860502615	1.83628103084798
C	3.16211910281409	-0.44564698269741	-1.41733442119447
C	-3.12323510755318	-0.49221263841891	-1.32977943928266
H	0.03311666327825	0.81666421840024	1.23136017029500
C	-2.27306997634772	1.59126006392663	0.51493918973533
H	-3.24156510008422	2.08359129113002	0.34368632813266
C	-1.20709292179984	2.38360259945916	0.82731335460016
H	-1.29126358488256	3.48607796961042	0.82867747792898
C	1.26478082105514	2.38684320473112	0.80899646673450
H	1.35171660788302	3.48893710076531	0.82446607752402
C	2.31902201538809	1.59608469932761	0.46155692125695
H	3.28544030697832	2.08635261829107	0.26648837598017
C	4.29016302789154	-0.09591989644156	2.15446125376219
H	4.73674209378688	-0.54252904722560	3.06877157147671
H	5.08082383868636	-0.06385349022120	1.38133854768231
H	3.99985618419440	0.94507729746079	2.39835678485467
C	3.48995261330912	-2.39466035030037	1.47600825970692
H	2.63451510937036	-3.00544268236753	1.12778131760380
H	4.31982442804627	-2.47359381483117	0.74687057514599
H	3.84931547697586	-2.83658397426234	2.43009607900639
C	2.01510857163183	-0.90167022080090	2.84762590870746
H	2.47004012195435	-1.26019642072832	3.79632872663906
H	1.63889022528926	0.12683363925358	3.02787361272880
H	1.16072589071392	-1.56068236189008	2.59777713085161
C	4.64171551670031	-0.05353595012238	-1.31177301350943
H	5.20659380831120	-0.74028943663848	-0.65186767772252
H	5.10747074811946	-0.11079138245119	-2.31921632058139
H	4.78684168970200	0.98196813318880	-0.94360477337328
C	3.02716454766673	-1.90553967963369	-1.88575441688064
H	1.96561802338521	-2.20466071264630	-1.98208599566106
H	3.50483988555340	-2.00813991158503	-2.88372805849688
H	3.52657159009602	-2.61878881670115	-1.20481446482364
C	2.44412864871004	0.45620177245974	-2.44099110400095
H	2.52136122520712	1.53090433449308	-2.18381116882903
H	2.89558253292338	0.30411220185256	-3.44447031091517
H	1.36705056606731	0.19631466277684	-2.50765444599119
C	-3.49745354957411	-1.97609263017343	-1.47525461514757
H	-2.62113879824440	-2.63639785997703	-1.32485156320258
H	-4.30434496723558	-2.27327059359063	-0.77754207783685
H	-3.87330715353922	-2.15122229422886	-2.50607524326717
C	-4.37651128928598	0.38541404540161	-1.46183225247058
H	-4.88276366888369	0.14586474489959	-2.42149796891165
H	-5.10801915460725	0.21332534113390	-0.64958507735498
H	-4.12915118534541	1.46461726781685	-1.48731489819807
C	-2.11171663642506	-0.11847576565020	-2.43369010511996
H	-1.74501559377885	0.92233881410407	-2.31627644234053
H	-1.24315629187443	-0.80696301010831	-2.41595024443480
H	-2.60220606427128	-0.19758180100936	-3.42810555652356
C	-2.94757259197332	-2.45920632785237	1.84056396627291
H	-1.90973729571763	-2.82821239958368	1.72933814554904
H	-3.33764602589684	-2.82945315654299	2.81298748605373

H	-3.56657102711241	-2.91102328892122	1.04434856567728
C	-4.45997195509396	-0.43817999096337	1.96999233020295
H	-5.11316342074226	-0.85844307310367	1.18026876482394
H	-4.87116871547958	-0.77339391061623	2.94654852414441
H	-4.53621789002687	0.66730056432641	1.93866669086372
C	-2.16915454487420	-0.39787877138049	3.01674868190238
H	-1.11679023011391	-0.73813519844084	2.93561903063355
H	-2.17708713755130	0.70862726398593	3.07650381918999
H	-2.58340447046532	-0.79701331479058	3.96690936548337
Ni	0.01977763733321	-0.69167920057194	0.05794773878442
C	0.03209434920078	-2.57336289739223	-0.09774274529932
O	0.23006045942995	-3.16624528580543	0.95972306857028
O	-0.16909849451778	-2.77156361482228	-1.30015185617799

Supplementary Table 16. XYZ coordinates of calculated [Ni(CO_2){HN(CHCHP*t*Bu$_2$)$_2$]·thf (**C**).

C	-0.56164361866490	-2.21700693817498	2.73164394388992
P	0.97013689145665	-2.40610252708745	1.59033657875014
N	3.16899159075220	-0.46286453960120	2.08157645326350
P	2.90134520889423	0.70320414955439	-0.64244041177926
C	2.46502520412638	2.56989788024755	-0.68333195360142
C	0.96938581425983	-4.09927185655611	0.69618209194251
C	3.51549428335388	0.08063649680775	-2.34816255812514
H	2.68976924077378	0.28031041306609	2.65705174957027
C	4.43934779328301	0.67980989042615	0.36709597763048
H	5.38716242268725	1.12929830939045	0.02943702472545
C	4.39995951382102	0.05951322410651	1.56483221699977
H	5.30116126308501	-0.06887275812672	2.19602097273062
C	3.28937936454645	-1.66453140997513	2.85519225223863
H	4.20122274743235	-1.76961631888904	3.47619005360079
C	2.33919893617388	-2.62127079968649	2.80028728370529
H	2.44101496757498	-3.51723346244485	3.43535694310844
C	-0.54420221663261	-3.13343949658152	3.96375372582449
H	-1.43687856837252	-2.91681330816566	4.59027655216245
H	-0.57583072834208	-4.20774943010757	3.70558375350739
H	0.35005239758709	-2.95521436385489	4.59467376535409
C	-1.84736655547342	-2.41743067889066	1.91439151024937
H	-1.85123592106935	-1.78099628127836	1.00742359028040
H	-1.99619863779618	-3.47359654846029	1.61671806067326
H	-2.72108359669441	-2.12293782254362	2.53517869800266
C	-0.49025121949261	-0.74966360333701	3.19367766871550
H	-1.31511946344655	-0.54767575363016	3.91221006707826
H	0.46805962130567	-0.53549804609691	3.70709819845671
H	-0.59052889691296	-0.06159204278123	2.32988788941776
C	0.61438032704327	-5.29571867186393	1.58887770501474
H	-0.44533910056925	-5.27813383426637	1.90940238511664
H	0.76390181878367	-6.23571172794122	1.01448285211211
H	1.25311449154772	-5.36091142512864	2.49328465547512
C	0.00691856083693	-4.01658484468377	-0.50272193885169
H	0.18324595694158	-3.11163814168999	-1.11838271345776

H	0.14807510328501	-4.91517423303468	-1.14125015424306
H	-1.05281714019419	-4.00033861831100	-0.18595003110281
C	2.40687889321992	-4.25779701982389	0.16638963445213
H	3.14669577138368	-4.37148096856022	0.98375980855820
H	2.46238455789309	-5.16198366196841	-0.47650792824311
H	2.70207478510504	-3.38194791816093	-0.44279219591868
C	2.51561735162369	0.47673562597811	-3.44634918432689
H	1.47796576674662	0.21437395187293	-3.15733387968193
H	2.57130913112097	1.55469376015421	-3.69407621606742
H	2.76187722372116	-0.08571177804641	-4.37264117804510
C	4.93210626599139	0.55343954888623	-2.70693036767251
H	5.20350289123362	0.15534227401385	-3.70848272930668
H	5.01855466163687	1.65570273576637	-2.75455992826397
H	5.68957140082120	0.17507280517298	-1.99206798087898
C	3.50247665066898	-1.45202562613229	-2.20362352683788
H	4.14620878221497	-1.79010448620065	-1.36558478728150
H	2.46918126916290	-1.80653498643309	-2.02049103577280
H	3.88252481298368	-1.91661489935340	-3.13931114010580
C	1.14648518353548	2.80003291144339	-1.44586834131272
H	0.32578319978558	2.18449116802357	-1.02846588645141
H	0.86105735459035	3.86926009461171	-1.33881021424068
H	1.23357694708573	2.58843473658885	-2.52611892219952
C	3.57987925074153	3.45461779525088	-1.25523668298536
H	3.72637962911273	3.28600737713904	-2.34008760795673
H	3.30560502833835	4.52432733719428	-1.12581393906684
H	4.55063688206545	3.29833800797232	-0.74280890357494
C	2.21099110830861	2.91098435729096	0.79656322382630
H	1.40997112718526	2.25737680588304	1.19699653486530
H	3.11488999280697	2.78746858681267	1.42623837768667
H	1.87121281304621	3.96571444307437	0.88127882712630
Ni	1.62303085308549	-0.58342789822575	0.57392921591911
C	0.12438455500949	-0.39578423438451	-0.57080194808738
O	-0.55119766879555	0.40282555348552	0.12567368310741
O	-0.00032711801243	-0.93632008514733	-1.67269453353113
C	2.51290130141757	0.88956301791478	5.30601418329385
O	2.23143703153894	1.37856323290302	3.98409606223121
C	1.15209918556950	2.34186286458751	4.02837910974243
C	0.79321354729306	2.50709228482727	5.51071316983964
C	1.24967952391797	1.17207045214004	6.11698136729942
H	2.76392215187691	-0.18872831102986	5.22851104261053
H	3.39548432831928	1.42649631614947	5.72799568644159
H	1.49299080318139	3.28621900372085	3.55739516532403
H	0.30590718040536	1.94208485888414	3.43048465885316
H	1.36388545053977	3.34652692351590	5.95947849982078
H	-0.28390157691904	2.71280729468600	5.66289366679695
H	1.43860611329875	1.22297865251891	7.20688613920704
H	0.49399104025270	0.38136147459408	5.93178023237225

Supplementary Table 17. XYZ coordinates of calculated [Ni{N(CHCHP*t*Bu$_2$)$_2$}] (**7**).

Ni	0.02806394508694	-0.14897416341798	0.35926881447098

C	3.15726597492617	-0.80176328788488	1.81173051096575
P	2.22634099694931	-0.04454579914719	0.32941141652220
N	0.02981760597430	1.78834171784627	0.53945939786692
P	-2.17008023680426	-0.04092451861640	0.33384457828215
C	-3.09929877101212	-0.79582071121174	1.81839720611388
C	3.00911286409530	-0.50800263400742	-1.34837643087055
C	-2.95659519581510	-0.50376802983327	-1.34232545852809
C	-2.36171189729480	1.74719484392766	0.52041982856325
H	-3.32561839149214	2.27499246611488	0.55596553687717
C	-1.16910095969529	2.43504439676006	0.59700208710479
H	-1.15645799319832	3.54019404904214	0.70506233155304
C	1.22991293192018	2.43302407019671	0.59508058281666
H	1.21931255385186	3.53817808893438	0.70331322317057
C	2.42123521772690	1.74318495787457	0.51639414285029
H	3.38608505148644	2.26936034409491	0.55032366273558
C	4.52953002947706	-0.16029622293787	2.06299295767365
H	4.97354395073551	-0.58408153628004	2.98988548635195
H	5.24317421925274	-0.34487378242475	1.23824513029552
H	4.44062210764206	0.93449761066529	2.20740361101049
C	3.28477719303780	-2.32250042715353	1.64426471746515
H	2.30916781396213	-2.78185633156516	1.38221744082339
H	4.02163199775517	-2.59898345111036	0.86439425459617
H	3.62838201979641	-2.77677390495008	2.59847478463888
C	2.23095957376364	-0.49853495418203	3.00616537779589
H	2.69231606291474	-0.87037989262624	3.94644298811700
H	2.05236651918377	0.59065213886283	3.11150418782723
H	1.24522682994042	-0.99108092864375	2.87913143155877
C	4.53198893583588	-0.33909568980918	-1.40263660121797
H	5.05671403828422	-1.07428844304062	-0.76125494472118
H	4.89128707054688	-0.49593232297540	-2.44309468872833
H	4.84391188126366	0.67903700077067	-1.09347226000145
C	2.59651052643136	-1.94783975204691	-1.70096337000778
H	1.49728659008263	-2.06700017531100	-1.60187794541161
H	2.87859974495069	-2.17527984651117	-2.75152128492119
H	3.08037589353765	-2.70240565273144	-1.05286933926715
C	2.33827377559715	0.44965591600974	-2.35177357843448
H	2.62605462812139	1.50294313125214	-2.16882508608588
H	2.63277415750491	0.17239501104057	-3.38648456562775
H	1.23194554052840	0.38739469261037	-2.27564165655753
C	-2.54595499797546	-1.94393370084742	-1.69587807745885
H	-1.44665135450476	-2.06412123049499	-1.59890403597919
H	-3.02928079130413	-2.69813021310376	-1.04697082751398
H	-2.83024979269629	-2.17097688191331	-2.74592406085297
C	-4.47941247918097	-0.33346380344241	-1.39366638066437
H	-4.84079992358340	-0.48944237131636	-2.43353008379284
H	-5.00360826217668	-1.06851898723355	-0.75169574057431
H	-4.78986131567816	0.68478559823007	-1.08339965725700
C	-2.28679491144440	0.45339604470869	-2.34689362347399
H	-2.57337640204170	1.50690914535057	-2.16334915606240
H	-1.18038415729243	0.39019812237775	-2.27267457104792
H	-2.58335233246350	0.17643622228896	-3.38109567359280

C	-3.22683393243357	-2.31682879786506	1.65340543142989
H	-2.25148140472845	-2.77637600147533	1.39073359709454
H	-3.56910426863698	-2.76973273749607	2.60874472274766
H	-3.96465502014362	-2.59460600007705	0.87492239104448
C	-4.47129472132410	-0.15406074641839	2.07032774417771
H	-5.18604155707809	-0.34028461330477	1.24690845640635
H	-4.91398340797626	-0.57615400687169	2.99862461084035
H	-4.38233828292794	0.94101690477502	2.21256791776123
C	-2.17161541275262	-0.49070034954834	3.01127694571498
H	-1.18573616452653	-0.98277383481611	2.88355977487278
H	-1.99352698350535	0.59870660017924	3.11512774343192
H	-2.63155692047625	-0.86174433927053	3.95256107508237

Supplementary Table 18. XYZ coordinates of calculated [Ni(CO){N(CHCHP*t*Bu_2)$_2$} (**5**).

Ni	0.02833780383172	-0.31848864451536	0.79349837398762
C	3.41393156006784	-0.80193481984163	1.74588470282729
P	2.26006790092156	-0.07368671737996	0.40939677071137
N	0.03000294171802	1.69449751677761	0.85221938217523
P	-2.20376400284898	-0.07008454273141	0.41416535093728
C	-3.35633263248763	-0.79719964804776	1.75231485949848
C	2.84394508377017	-0.50645607634926	-1.36172083214643
C	-2.79125033561249	-0.50122024562027	-1.35617236759000
C	-2.35504329252069	1.71553217820672	0.60223860408041
H	-3.30213581687949	2.27042401157711	0.54381018315942
C	-1.15628059120797	2.36669777636210	0.76943445250360
H	-1.11890782690941	3.47519867278764	0.82091775193662
C	1.21712390230133	2.36483840780330	0.76644483518560
H	1.18160546473674	3.47340470773965	0.81783442426941
C	2.41446530722760	1.71178071347791	0.59647038264511
H	3.36227874442105	2.26518611144541	0.53568748918711
C	4.77740112384080	-0.09196794061641	1.79446234509764
H	5.36872037638500	-0.50214767143758	2.64148498397363
H	5.37235471492687	-0.23719489024472	0.87429453734088
H	4.66227284039087	0.99566195050222	1.96932362748437
C	3.60382748516540	-2.31434120760137	1.55151025298093
H	2.63798425213190	-2.84965263457590	1.46943159684917
H	4.21166583455913	-2.54738921077874	0.65503630568810
H	4.14276457428581	-2.73138557759970	2.42911829649274
C	2.67885149650591	-0.53668535190739	3.07567827917984
H	3.32315252465144	-0.85679986130668	3.92246302758106
H	2.45369024607852	0.54190992809671	3.20141433577694
H	1.72261750708061	-1.09149469655426	3.13592537369345
C	4.35739023263925	-0.37602815744269	-1.57645343032914
H	4.93028979147581	-1.13761208965869	-1.01269819305902
H	4.59152782160191	-0.52085710570617	-2.65376955159584
H	4.72956556276985	0.62778593212914	-1.28901975067987
C	2.36222858748355	-1.93007657777423	-1.69239836505639
H	1.26530136529030	-2.01646143366473	-1.55503744377724
H	2.59874260763479	-2.16836916845297	-2.75167654299354
H	2.84202352450735	-2.70014887883511	-1.05955662451011

C	2.11068850166505	0.49237387924820	-2.27431946547176
H	2.46216429650938	1.52982386959336	-2.11448881403053
H	2.28162922507630	0.21820548141888	-3.33704527758732
H	1.01788315784943	0.47731902949803	-2.08683796593562
C	-2.31095816120221	-1.92491607622084	-1.68861332283577
H	-1.21377941568484	-2.01186022794829	-1.55361145818607
H	-2.78975445184750	-2.69506840639887	-1.05510725469573
H	-2.54984950224307	-2.16257511465308	-2.74750031152001
C	-4.30499046967949	-0.36973281596848	-1.56814640977508
H	-4.54109720166098	-0.51378016447195	-2.64513798165227
H	-4.87738918902396	-1.13129562636029	-1.00385247934564
H	-4.67603478832553	0.63414796176377	-1.27947940629006
C	-2.05899042492858	0.49778812404587	-2.26937437468322
H	-2.41000386966545	1.53525452545268	-2.10866500510373
H	-0.96594668788436	0.48242233552478	-2.08328141965149
H	-2.23140366344736	0.22408395141879	-3.33198247405994
C	-3.54895531394928	-2.30920067467635	1.55745733519711
H	-2.58409184175306	-2.84598270497023	1.47350074010171
H	-4.08708088697183	-2.72585869021615	2.43574554186214
H	-4.15864298356584	-2.54081255706922	0.66187280060283
C	-4.71854946133945	-0.08503399270920	1.80370445992298
H	-5.31527448196402	-0.22849160782598	0.88440794642043
H	-5.30915031213451	-0.49488615807599	2.65138682823939
H	-4.60129341063454	1.00226034531224	1.97924208261888
C	-2.61848824750240	-0.53382329136386	3.08094511923986
H	-1.66302421056579	-1.09016422766797	3.13924743536308
H	-2.39139206194301	0.54434840381490	3.20684782865076
H	-3.26180762651078	-0.85334705929893	3.92869823103171
C	0.02757543547260	-1.99300252418837	1.33419731019742
O	0.02663232792064	-3.16899676526998	1.34640123787025

Crystallographic Details:

CCDC 1561988 (**1**), CCDC 1561989 (**3**), CCDC 1561990 (**4**), CCDC 1561991 (**5**), CCDC 1561992 (**6**), CCDC 1561993 (**7**), CCDC 1561994 (**9**), CCDC 1574302 (**10**) and CCDC 1574303 (**11**) contain the supplementary crystallographic data for this paper. This data can be obtained free of charge via http://www.ccdc.cam.ac.uk/ products/csd/request/ (or from Cambridge Crystallographic Data Centre, 12 Union Road, Cambridge, CB2 1EZ, UK. Fax: +44-1223- 336-033; e-mail: deposit@ccdc.cam.ac.uk).

Suitable single crystals for X-ray structure determination were selected from the mother liquor under an inert gas atmosphere and transferred in protective perfluoro polyether oil on a microscope slide. The selected and mounted crystals were transferred to the cold gas stream on the diffractometer. The diffraction data were obtained at 100 K on a Bruker D8 three-circle diffractometer, equipped with a PHOTON 100 CMOS detector and an INCOATEC microfocus source with Quazar mirror optics (Mo-Kα radiation, λ= 0.71073 Å).

The data obtained were integrated with SAINT and a semi-empirical absorption correction from equivalents with SADABS was applied. The structure was solved and refined using the Bruker SHELX 2014 software package.*(52-55)* All non-hydrogen atoms were refined with anisotropic displacement parameters. All C-H hydrogen atoms were refined isotropically on calculated positions by using a riding model with their U_{iso} values constrained to 1.5 U_{eq} of their pivot atoms for terminal sp3 carbon atoms and 1.2 times for all other atoms.

X-ray Single-Crystal Structure Analysis of **1**:

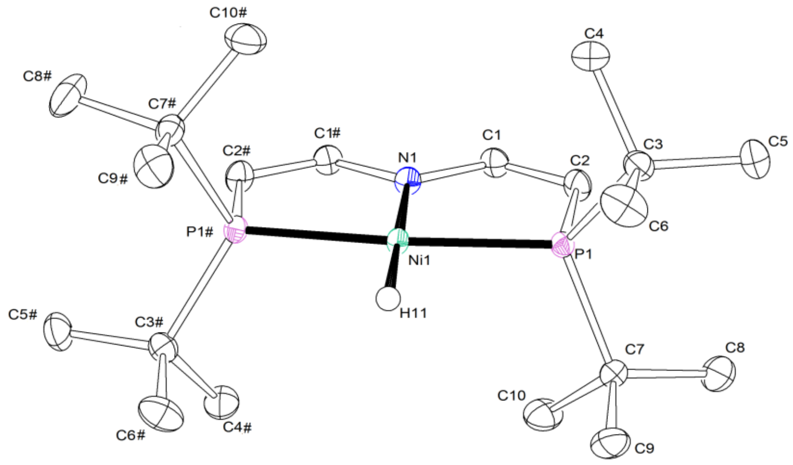

Supplementary Figure 48**.** Thermal ellipsoid plot of **1** with the anisotropic displacement parameters at the 50% probability level. All C-H hydrogen atoms are omitted for clarity. The Ni-H hydrogen atom was found from residual density map and isotropically refined. The asymmetric unit contains only a half complex molecule.

Supplementary Table 19. Crystal data and structure refinement for **1**.

Identification code	SF_SF_010216	
Empirical formula	$C_{20}H_{41}NNiP_2$	
Formula weight	416.19	
Temperature	104(2) K	
Wavelength	0.71073 Å	
Crystal system	Monoclinic	
Space group	C2/c	
Unit cell dimensions	a = 21.4284(12) Å	α = 90°
	b = 7.7159(4) Å	β = 101.926(2)°
	c = 14.0489(8) Å	γ = 90°
Volume	2272.7(2) $Å^3$	
Z	4	
Density (calculated)	1.216 Mg/m^3	
Absorption coefficient	0.997 mm^{-1}	
F(000)	904	
Crystal size	0.202 x 0.126 x 0.103 mm^3	
Crystal shape and color	Block, clear light yellow	
Theta range for data collection	2.813 to 33.217°	
Index ranges	-31<=h<=32, -11<=k<=11, -21<=l<=21	
Reflections collected	61848	
Independent reflections	4349 [R(int) = 0.0355]	
Completeness to theta = 25.242°	99.9 %	

Max. and min. transmission	0.7465 and 0.7109
Refinement method	Full-matrix least-squares on F^2
Data / restraints / parameters	4349 / 0 / 118
Goodness-of-fit on F^2	1.050
Final R indices [I>2sigma(I)]	R1 = 0.0239, wR2 = 0.0535
R indices (all data)	R1 = 0.0340, wR2 = 0.0574
Largest diff. peak and hole	0.379 and -0.484 eÅ^{-3}

Supplementary Table 20. Bond lengths [Å] and angles [°] for **1**.

Ni(1)-N(1)	1.9176(10)	N(1)-Ni(1)-P(1)#1	86.632(7)
Ni(1)-P(1)#1	2.1880(2)	N(1)-Ni(1)-P(1)	86.632(7)
Ni(1)-P(1)	2.1880(2)	P(1)#1-Ni(1)-P(1)	173.263(13)
Ni(1)-H(11)	1.66(3)	N(1)-Ni(1)-H(11)	180.0
N(1)-C(1)	1.3646(10)	P(1)#1-Ni(1)-H(11)	93.368(10)
N(1)-C(1)#1	1.3646(10)	P(1)-Ni(1)-H(11)	93.368(7)
P(1)-C(2)	1.7933(9)	C(1)-N(1)-C(1)#1	121.42(10)
P(1)-C(3)	1.8733(9)	C(1)-N(1)-Ni(1)	119.29(5)
P(1)-C(7)	1.8757(9)	C(1)#1-N(1)-Ni(1)	119.29(5)
C(1)-C(2)	1.3587(13)	C(2)-P(1)-C(3)	106.12(4)
C(3)-C(5)	1.5280(13)	C(2)-P(1)-C(7)	104.98(4)
C(3)-C(6)	1.5339(13)	C(3)-P(1)-C(7)	113.37(4)
C(3)-C(4)	1.5379(13)	C(2)-P(1)-Ni(1)	99.29(3)
C(7)-C(9)	1.5276(14)	C(3)-P(1)-Ni(1)	117.83(3)
C(7)-C(8)	1.5303(14)	C(7)-P(1)-Ni(1)	113.04(3)
C(7)-C(10)	1.5371(14)	C(2)-C(1)-N(1)	120.98(8)
		C(1)-C(2)-P(1)	113.76(7)
		C(5)-C(3)-C(6)	110.31(8)
		C(5)-C(3)-C(4)	108.92(8)
		C(6)-C(3)-C(4)	107.84(8)
		C(5)-C(3)-P(1)	114.11(7)
		C(6)-C(3)-P(1)	110.77(6)
		C(4)-C(3)-P(1)	104.55(6)
		C(9)-C(7)-C(8)	110.02(9)
		C(9)-C(7)-C(10)	108.55(8)
		C(8)-C(7)-C(10)	108.56(9)
		C(9)-C(7)-P(1)	111.46(7)
		C(8)-C(7)-P(1)	113.94(7)
		C(10)-C(7)-P(1)	103.98(6)

Symmetry transformations used to generate equivalent atoms:
#1 -x+1,y,-z+1/2

Supplementary Table 21. Torsion angles [°] for **1**.

C(1)#1-N(1)-C(1)-C(2)	-179.01(10)
Ni(1)-N(1)-C(1)-C(2)	0.99(10)
N(1)-C(1)-C(2)-P(1)	0.78(11)

C(3)-P(1)-C(2)-C(1)	-124.45(7)
C(7)-P(1)-C(2)-C(1)	115.23(7)
Ni(1)-P(1)-C(2)-C(1)	-1.79(7)
C(2)-P(1)-C(3)-C(5)	-60.55(8)
C(7)-P(1)-C(3)-C(5)	54.16(8)
Ni(1)-P(1)-C(3)-C(5)	-170.59(6)
C(2)-P(1)-C(3)-C(6)	174.25(7)
C(7)-P(1)-C(3)-C(6)	-71.04(8)
Ni(1)-P(1)-C(3)-C(6)	64.21(8)
C(2)-P(1)-C(3)-C(4)	58.34(7)
C(7)-P(1)-C(3)-C(4)	173.05(6)
Ni(1)-P(1)-C(3)-C(4)	-51.69(7)
C(2)-P(1)-C(7)-C(9)	178.20(7)
C(3)-P(1)-C(7)-C(9)	62.81(8)
Ni(1)-P(1)-C(7)-C(9)	-74.62(7)
C(2)-P(1)-C(7)-C(8)	52.95(9)
C(3)-P(1)-C(7)-C(8)	-62.44(9)
Ni(1)-P(1)-C(7)-C(8)	160.13(7)
C(2)-P(1)-C(7)-C(10)	-65.05(7)
C(3)-P(1)-C(7)-C(10)	179.56(6)
Ni(1)-P(1)-C(7)-C(10)	42.13(7)

Symmetry transformations used to generate equivalent atoms:
#1 -x+1,y,-z+1/2

X-ray Single-Crystal Structure Analysis of **3**:

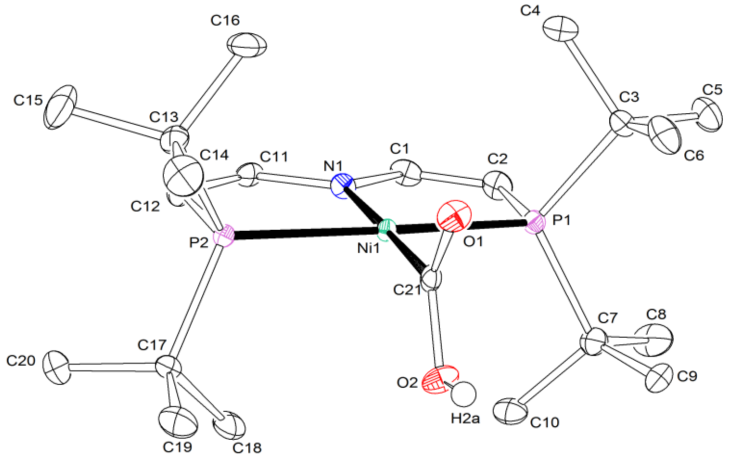

Supplementary Figure 49. Thermal ellipsoid plot of **3** with the anisotropic displacement parameters at the 50% probability level. All C-H hydrogen atoms are omitted for clarity. The O-H hydrogen atom was found from residual density map and constrained to 1.5 U_{eq} of the connected O-atom.

Supplementary Table 22. Crystal data and structure refinement for **3**.

Identification code	mo_SF_SF_290816_0m_a	
Empirical formula	$C_{21}H_{41}NNiO_2P_2$	
Formula weight	460.20	
Temperature	100(2) K	
Wavelength	0.71073 Å	
Crystal system	Triclinic	
Space group	P-1	
Unit cell dimensions	a = 8.4481(5) Å	α = 103.507(2)°
	b = 11.4423(6) Å	β = 92.683(2)°
	c = 13.6806(7) Å	γ = 110.468(2)°
Volume	1192.64(11) $Å^3$	
Z	2	
Density (calculated)	1.281 Mg/m^3	
Absorption coefficient	0.963 mm^{-1}	
F(000)	496	
Crystal size	0.136 x 0.117 x 0.111 mm^3	
Crystal shape and color	Block, clear light yellow	
Theta range for data collection	2.156 to 28.396°	
Index ranges	-11<=h<=11, -15<=k<=15, -18<=l<=17	
Reflections collected	46494	
Independent reflections	5980 [R(int) = 0.1041]	
Completeness to theta = 25.242°	100.0 %	
Max. and min. transmission	0.7457 and 0.6905	
Refinement method	Full-matrix least-squares on F^2	
Data / restraints / parameters	5980 / 0 / 258	
Goodness-of-fit on F^2	1.015	
Final R indices [I>2sigma(I)]	R1 = 0.0425, wR2 = 0.0684	
R indices (all data)	R1 = 0.0787, wR2 = 0.0773	
Largest diff. peak and hole	0.467 and -0.415 $eÅ^{-3}$	

Supplementary Table 23. Bond lengths [Å] and angles [°] for **3**.

Ni(1)-C(21)	1.854(2)	C(21)-Ni(1)-N(1)	179.00(9)
Ni(1)-N(1)	1.9155(17)	C(21)-Ni(1)-P(2)	93.35(7)
Ni(1)-P(2)	2.2185(7)	N(1)-Ni(1)-P(2)	85.80(6)
Ni(1)-P(1)	2.2191(6)	C(21)-Ni(1)-P(1)	95.32(7)
O(1)-C(21)	1.274(3)	N(1)-Ni(1)-P(1)	85.51(6)
P(1)-C(2)	1.785(2)	P(2)-Ni(1)-P(1)	171.08(2)
P(1)-C(7)	1.872(2)	C(2)-P(1)-C(7)	105.30(11)
P(1)-C(3)	1.879(2)	C(2)-P(1)-C(3)	104.74(10)
N(1)-C(11)	1.364(3)	C(7)-P(1)-C(3)	112.97(10)
N(1)-C(1)	1.366(3)	C(2)-P(1)-Ni(1)	99.19(8)
C(1)-C(2)	1.340(3)	C(7)-P(1)-Ni(1)	114.62(7)
P(2)-C(12)	1.787(2)	C(3)-P(1)-Ni(1)	117.58(8)
P(2)-C(13)	1.875(2)	C(11)-N(1)-C(1)	120.41(19)
P(2)-C(17)	1.879(2)	C(11)-N(1)-Ni(1)	119.62(15)

O(2)-C(21)	1.299(3)		C(1)-N(1)-Ni(1)	119.89(15)
O(2)-H(2A)	0.80(2)		C(12)-P(2)-C(13)	105.17(10)
C(3)-C(5)	1.529(3)		C(12)-P(2)-C(17)	104.30(10)
C(3)-C(6)	1.529(3)		C(13)-P(2)-C(17)	114.49(10)
C(3)-C(4)	1.536(3)		C(12)-P(2)-Ni(1)	98.94(8)
C(16)-C(13)	1.537(3)		C(13)-P(2)-Ni(1)	115.86(7)
C(15)-C(13)	1.535(3)		C(17)-P(2)-Ni(1)	115.35(7)
C(20)-C(17)	1.529(3)		C(1)-C(2)-P(1)	113.99(17)
C(14)-C(13)	1.530(3)		C(5)-C(3)-C(6)	109.56(19)
C(19)-C(17)	1.531(3)		C(5)-C(3)-C(4)	109.35(19)
C(18)-C(17)	1.537(3)		C(6)-C(3)-C(4)	107.6(2)
C(9)-C(7)	1.530(3)		C(5)-C(3)-P(1)	112.65(16)
C(7)-C(10)	1.534(3)		C(6)-C(3)-P(1)	113.22(16)
C(7)-C(8)	1.537(3)		C(4)-C(3)-P(1)	104.13(15)
C(12)-C(11)	1.341(3)		O(1)-C(21)-O(2)	119.50(19)
			O(1)-C(21)-Ni(1)	122.09(16)
			O(2)-C(21)-Ni(1)	118.41(16)
			C(20)-C(17)-C(19)	108.96(19)
			C(20)-C(17)-C(18)	108.99(19)
			C(19)-C(17)-C(18)	108.54(19)
			C(20)-C(17)-P(2)	111.74(15)
			C(19)-C(17)-P(2)	113.85(16)
			C(18)-C(17)-P(2)	104.55(15)
			C(9)-C(7)-C(10)	109.30(19)
			C(9)-C(7)-C(8)	109.6(2)
			C(10)-C(7)-C(8)	108.13(19)
			C(9)-C(7)-P(1)	112.23(16)
			C(10)-C(7)-P(1)	104.49(15)
			C(8)-C(7)-P(1)	112.87(16)
			C(14)-C(13)-C(15)	108.8(2)
			C(14)-C(13)-C(16)	108.67(19)
			C(15)-C(13)-C(16)	109.2(2)
			C(14)-C(13)-P(2)	113.82(16)
			C(15)-C(13)-P(2)	112.75(16)
			C(16)-C(13)-P(2)	103.37(15)
			C(11)-C(12)-P(2)	114.06(17)
			C(12)-C(11)-N(1)	121.5(2)

Supplementary Table 24**.** Torsion angles [°] for **3**.

C(11)-N(1)-C(1)-C(2)	-178.5(2)
Ni(1)-N(1)-C(1)-C(2)	-1.7(3)
N(1)-C(1)-C(2)-P(1)	0.0(3)
C(7)-P(1)-C(2)-C(1)	120.06(19)
C(3)-P(1)-C(2)-C(1)	-120.59(18)
Ni(1)-P(1)-C(2)-C(1)	1.25(19)
C(2)-P(1)-C(3)-C(5)	-59.62(19)
C(7)-P(1)-C(3)-C(5)	54.45(19)
Ni(1)-P(1)-C(3)-C(5)	-168.51(14)

C(2)-P(1)-C(3)-C(6)	175.36(17)
C(7)-P(1)-C(3)-C(6)	-70.57(19)
Ni(1)-P(1)-C(3)-C(6)	66.47(18)
C(2)-P(1)-C(3)-C(4)	58.76(18)
C(7)-P(1)-C(3)-C(4)	172.83(15)
Ni(1)-P(1)-C(3)-C(4)	-50.13(17)
P(2)-Ni(1)-C(21)-O(1)	-90.40(17)
P(1)-Ni(1)-C(21)-O(1)	91.71(17)
P(2)-Ni(1)-C(21)-O(2)	89.62(16)
P(1)-Ni(1)-C(21)-O(2)	-88.28(16)
C(12)-P(2)-C(17)-C(20)	-44.64(19)
C(13)-P(2)-C(17)-C(20)	69.75(19)
Ni(1)-P(2)-C(17)-C(20)	-151.99(14)
C(12)-P(2)-C(17)-C(19)	-168.60(17)
C(13)-P(2)-C(17)-C(19)	-54.21(19)
Ni(1)-P(2)-C(17)-C(19)	84.05(17)
C(12)-P(2)-C(17)-C(18)	73.11(17)
C(13)-P(2)-C(17)-C(18)	-172.50(14)
Ni(1)-P(2)-C(17)-C(18)	-34.24(16)
C(2)-P(1)-C(7)-C(9)	164.71(16)
C(3)-P(1)-C(7)-C(9)	50.98(19)
Ni(1)-P(1)-C(7)-C(9)	-87.38(16)
C(2)-P(1)-C(7)-C(10)	-76.97(16)
C(3)-P(1)-C(7)-C(10)	169.30(14)
Ni(1)-P(1)-C(7)-C(10)	30.94(16)
C(2)-P(1)-C(7)-C(8)	40.3(2)
C(3)-P(1)-C(7)-C(8)	-73.4(2)
Ni(1)-P(1)-C(7)-C(8)	148.19(16)
C(12)-P(2)-C(13)-C(14)	166.94(17)
C(17)-P(2)-C(13)-C(14)	53.06(19)
Ni(1)-P(2)-C(13)-C(14)	-84.98(17)
C(12)-P(2)-C(13)-C(15)	42.4(2)
C(17)-P(2)-C(13)-C(15)	-71.5(2)
Ni(1)-P(2)-C(13)-C(15)	150.47(15)
C(12)-P(2)-C(13)-C(16)	-75.38(17)
C(17)-P(2)-C(13)-C(16)	170.74(14)
Ni(1)-P(2)-C(13)-C(16)	32.70(17)
C(13)-P(2)-C(12)-C(11)	121.89(18)
C(17)-P(2)-C(12)-C(11)	-117.28(18)
Ni(1)-P(2)-C(12)-C(11)	1.89(18)
P(2)-C(12)-C(11)-N(1)	-1.7(3)
C(1)-N(1)-C(11)-C(12)	177.1(2)
Ni(1)-N(1)-C(11)-C(12)	0.3(3)

Supplementary Table 25. Hydrogen bonds for **3** [Å and °].

D-H...A	d(D-H)	d(H...A)	d(D...A)	<(DHA)
O(2)-H(2A)...O(1)#1	0.80	1.85	2.636(2)	167.0

Symmetry transformations used to generate equivalent atoms:
#1 -x+1,-y+1,-z+1

X-ray Single-Crystal Structure Analysis of **4**:

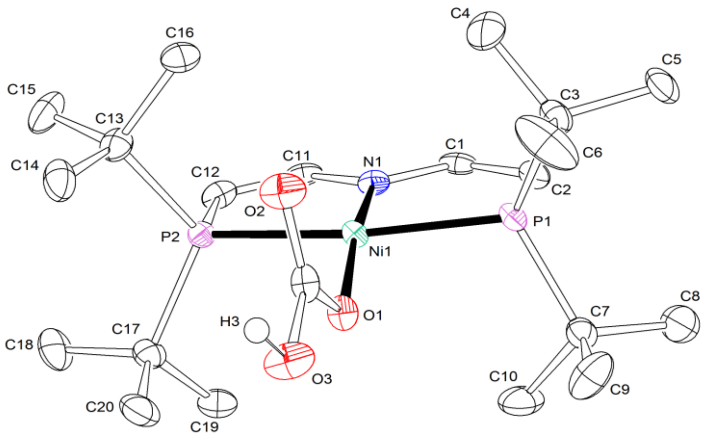

Supplementary Figure 50. Thermal ellipsoid plot of **4** with the anisotropic displacement parameters at the 50% probability level. All C-H hydrogen atoms are omitted for clarity. The O-H hydrogen atom was found from residual density map and isotropically refined.

Supplementary Table 26. Crystal data and structure refinement for **4**.

Identification code	mo_SF_SF_130417_2_0ma_a	
Empirical formula	$C_{21}H_{41}NNiO_3P_2$	
Formula weight	476.20	
Temperature	100(2) K	
Wavelength	0.71073 Å	
Crystal system	Monoclinic	
Space group	$P2_1/n$	
Unit cell dimensions	a = 14.9312(6) Å	α = 90°
	b = 11.4998(5) Å	β = 116.777(2)°
	c = 16.4761(7) Å	γ = 90°
Volume	2525.67(19) $Å^3$	
Z	4	
Density (calculated)	1.252 Mg/m^3	
Absorption coefficient	0.914 mm^{-1}	

F(000)	1024
Crystal size	0.174 x 0.088 x 0.034 mm^3
Crystal shape and color	Plate, clear orange
Theta range for data collection	2.248 to 28.398°
Index ranges	-19<=h<=19, -15<=k<=15, -22<=l<=21
Reflections collected	122428
Independent reflections	6296 [R(int) = 0.1642]
Completeness to theta = 25.242°	100.0 %
Refinement method	Full-matrix least-squares on F^2
Data / restraints / parameters	6296 / 0 / 269
Goodness-of-fit on F^2	1.081
Final R indices [I>2sigma(I)]	R1 = 0.0492, wR2 = 0.0873
R indices (all data)	R1 = 0.0799, wR2 = 0.0968
Largest diff. peak and hole	0.572 and -0.508 $eÅ^{-3}$

Supplementary Table 27. Bond lengths [Å] and angles [°] for **4**.

Ni(1)-O(1)	1.8736(17)	O(1)-Ni(1)-N(1)	169.00(9)
Ni(1)-N(1)	1.876(2)	O(1)-Ni(1)-P(1)	92.75(6)
Ni(1)-P(1)	2.2289(7)	N(1)-Ni(1)-P(1)	85.34(7)
Ni(1)-P(2)	2.2614(7)	O(1)-Ni(1)-P(2)	96.99(6)
P(1)-C(2)	1.776(3)	N(1)-Ni(1)-P(2)	85.18(7)
P(1)-C(7)	1.874(3)	P(1)-Ni(1)-P(2)	170.25(3)
P(1)-C(3)	1.881(3)	C(2)-P(1)-C(7)	106.17(12)
P(2)-C(12)	1.776(3)	C(2)-P(1)-C(3)	105.96(12)
P(2)-C(17)	1.873(3)	C(7)-P(1)-C(3)	114.35(13)
P(2)-C(13)	1.882(3)	C(2)-P(1)-Ni(1)	99.31(9)
O(1)-C(21)	1.271(3)	C(7)-P(1)-Ni(1)	114.56(9)
O(2)-C(21)	1.246(3)	C(3)-P(1)-Ni(1)	114.50(9)
O(3)-C(21)	1.327(3)	C(12)-P(2)-C(17)	105.91(13)
O(3)-H(3)	0.98(4)	C(12)-P(2)-C(13)	104.50(13)
N(1)-C(1)	1.369(3)	C(17)-P(2)-C(13)	113.09(13)
N(1)-C(11)	1.379(3)	C(12)-P(2)-Ni(1)	98.49(10)
C(1)-C(2)	1.339(4)	C(17)-P(2)-Ni(1)	114.28(9)
C(3)-C(5)	1.522(4)	C(13)-P(2)-Ni(1)	118.05(9)
C(3)-C(6)	1.524(4)	C(21)-O(1)-Ni(1)	123.97(17)
C(3)-C(4)	1.535(4)	C(21)-O(3)-H(3)	107(2)
C(7)-C(8)	1.520(4)	C(1)-N(1)-C(11)	119.0(2)
C(7)-C(9)	1.523(4)	C(1)-N(1)-Ni(1)	120.37(18)
C(7)-C(10)	1.534(4)	C(11)-N(1)-Ni(1)	120.31(18)
C(11)-C(12)	1.332(4)	C(2)-C(1)-N(1)	122.0(2)
C(13)-C(14)	1.526(4)	C(1)-C(2)-P(1)	112.8(2)
C(13)-C(15)	1.538(4)	C(5)-C(3)-C(6)	109.8(3)
C(13)-C(16)	1.538(4)	C(5)-C(3)-C(4)	109.1(2)
C(17)-C(20)	1.529(4)	C(6)-C(3)-C(4)	108.8(3)
C(17)-C(18)	1.530(4)	C(5)-C(3)-P(1)	111.92(18)
C(17)-C(19)	1.540(4)	C(6)-C(3)-P(1)	112.9(2)
		C(4)-C(3)-P(1)	103.98(19)

C(8)-C(7)-C(9)	110.4(3)
C(8)-C(7)-C(10)	109.3(3)
C(9)-C(7)-C(10)	108.7(2)
C(8)-C(7)-P(1)	111.9(2)
C(9)-C(7)-P(1)	112.2(2)
C(10)-C(7)-P(1)	104.12(19)
C(12)-C(11)-N(1)	122.1(2)
C(11)-C(12)-P(2)	113.6(2)
C(14)-C(13)-C(15)	109.6(2)
C(14)-C(13)-C(16)	109.7(2)
C(15)-C(13)-C(16)	107.9(2)
C(14)-C(13)-P(2)	113.4(2)
C(15)-C(13)-P(2)	111.54(19)
C(16)-C(13)-P(2)	104.46(19)
C(20)-C(17)-C(18)	109.5(2)
C(20)-C(17)-C(19)	109.1(2)
C(18)-C(17)-C(19)	109.2(2)
C(20)-C(17)-P(2)	111.57(18)
C(18)-C(17)-P(2)	112.3(2)
C(19)-C(17)-P(2)	105.12(19)
O(2)-C(21)-O(1)	124.7(2)
O(2)-C(21)-O(3)	121.5(2)
O(1)-C(21)-O(3)	113.7(2)

Supplementary Table 28. Torsion angles [°] for **4**.

N(1)-Ni(1)-O(1)-C(21)	-175.6(4)
P(1)-Ni(1)-O(1)-C(21)	104.69(19)
P(2)-Ni(1)-O(1)-C(21)	-74.83(19)
O(1)-Ni(1)-N(1)-C(1)	-76.7(5)
P(1)-Ni(1)-N(1)-C(1)	3.68(18)
P(2)-Ni(1)-N(1)-C(1)	-178.62(19)
O(1)-Ni(1)-N(1)-C(11)	96.9(5)
P(1)-Ni(1)-N(1)-C(11)	177.34(19)
P(2)-Ni(1)-N(1)-C(11)	-4.96(18)
C(11)-N(1)-C(1)-C(2)	-176.4(2)
Ni(1)-N(1)-C(1)-C(2)	-2.6(3)
N(1)-C(1)-C(2)-P(1)	-0.8(3)
C(7)-P(1)-C(2)-C(1)	122.1(2)
C(3)-P(1)-C(2)-C(1)	-115.9(2)
Ni(1)-P(1)-C(2)-C(1)	3.0(2)
C(2)-P(1)-C(3)-C(5)	-53.1(2)
C(7)-P(1)-C(3)-C(5)	63.5(2)
Ni(1)-P(1)-C(3)-C(5)	-161.47(18)
C(2)-P(1)-C(3)-C(6)	-177.6(2)
C(7)-P(1)-C(3)-C(6)	-61.1(3)
Ni(1)-P(1)-C(3)-C(6)	74.0(3)

C(2)-P(1)-C(3)-C(4)	64.6(2)
C(7)-P(1)-C(3)-C(4)	-178.83(19)
Ni(1)-P(1)-C(3)-C(4)	-43.8(2)
C(2)-P(1)-C(7)-C(8)	42.6(2)
C(3)-P(1)-C(7)-C(8)	-73.9(2)
Ni(1)-P(1)-C(7)-C(8)	151.1(2)
C(2)-P(1)-C(7)-C(9)	167.3(2)
C(3)-P(1)-C(7)-C(9)	50.8(2)
Ni(1)-P(1)-C(7)-C(9)	-84.2(2)
C(2)-P(1)-C(7)-C(10)	-75.3(2)
C(3)-P(1)-C(7)-C(10)	168.24(19)
Ni(1)-P(1)-C(7)-C(10)	33.2(2)
C(1)-N(1)-C(11)-C(12)	177.8(2)
Ni(1)-N(1)-C(11)-C(12)	4.1(3)
N(1)-C(11)-C(12)-P(2)	0.4(3)
C(17)-P(2)-C(12)-C(11)	-121.8(2)
C(13)-P(2)-C(12)-C(11)	118.5(2)
Ni(1)-P(2)-C(12)-C(11)	-3.4(2)
C(12)-P(2)-C(13)-C(14)	161.3(2)
C(17)-P(2)-C(13)-C(14)	46.6(2)
Ni(1)-P(2)-C(13)-C(14)	-90.6(2)
C(12)-P(2)-C(13)-C(15)	37.0(2)
C(17)-P(2)-C(13)-C(15)	-77.7(2)
Ni(1)-P(2)-C(13)-C(15)	145.08(19)
C(12)-P(2)-C(13)-C(16)	-79.3(2)
C(17)-P(2)-C(13)-C(16)	166.01(18)
Ni(1)-P(2)-C(13)-C(16)	28.8(2)
C(12)-P(2)-C(17)-C(20)	172.1(2)
C(13)-P(2)-C(17)-C(20)	-74.0(2)
Ni(1)-P(2)-C(17)-C(20)	64.8(2)
C(12)-P(2)-C(17)-C(18)	-64.5(2)
C(13)-P(2)-C(17)-C(18)	49.3(2)
Ni(1)-P(2)-C(17)-C(18)	-171.82(18)
C(12)-P(2)-C(17)-C(19)	54.0(2)
C(13)-P(2)-C(17)-C(19)	167.91(18)
Ni(1)-P(2)-C(17)-C(19)	-53.2(2)
Ni(1)-O(1)-C(21)-O(2)	-8.5(4)
Ni(1)-O(1)-C(21)-O(3)	171.48(16)

Supplementary Table 29. Hydrogen bonds for **4** [Å and °].

D-H...A	d(D-H)	d(H...A)	d(D...A)	<(DHA)
O(3)-H(3)...O(2)#1	0.98(4)	1.61(4)	2.582(3)	172(4)

Symmetry transformations used to generate equivalent atoms:
#1 -x+1,-y+1,-z+2

X-ray Single-Crystal Structure Analysis of **5**:

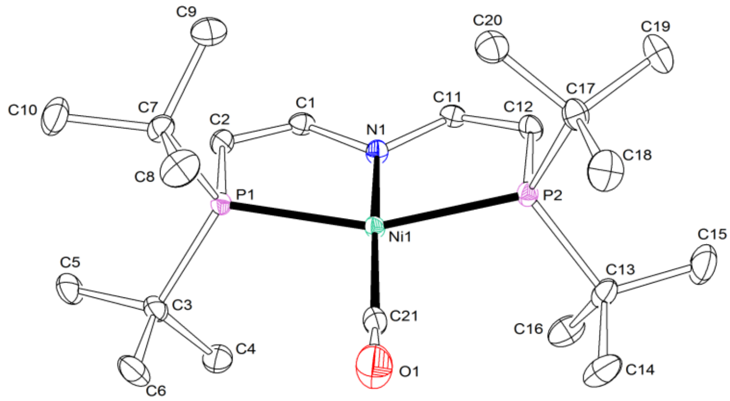

Supplementary Figure 51**.** Thermal ellipsoid plot of **5** with the anisotropic displacement parameters at the 50% probability level. All C-H hydrogen atoms are omitted for clarity.

Supplementary Table 30. Crystal data and structure refinement for **5**.

Identification code	mo_SF_SF_240517_0m_a	
Empirical formula	$C_{21}H_{40}NNiOP_2$	
Formula weight	443.19	
Temperature	100(2) K	
Wavelength	0.71073 Å	
Crystal system	Monoclinic	
Space group	$P2_1/c$	
Unit cell dimensions	a = 7.8351(5) Å	α = 90°
	b = 26.8613(15) Å	β = 107.270(2)°
	c = 11.7832(6) Å	γ = 90°
Volume	2368.1(2) Å^3	
Z	4	
Density (calculated)	1.243 Mg/m^3	
Absorption coefficient	0.964 mm^{-1}	
F(000)	956	
Crystal size	0.289 x 0.192 x 0.041 mm^3	
Crystal shape and color	plate, clear dark green-red	
Theta range for data collection	2.361 to 30.571°	
Index ranges	-11<=h<=11, -38<=k<=38, -16<=l<=16	
Reflections collected	76684	
Independent reflections	7257 [R(int) = 0.0579]	
Completeness to theta = 25.242°	100.0 %	
Refinement method	Full-matrix least-squares on F^2	
Data / restraints / parameters	7257 / 0 / 247	
Goodness-of-fit on F^2	1.137	
Final R indices [I>2sigma(I)]	R1 = 0.0403, wR2 = 0.0743	
R indices (all data)	R1 = 0.0537, wR2 = 0.0774	

Largest diff. peak and hole	0.555 and -0.518 eÅ^{-3}

Supplementary Table 31. Bond lengths [Å] and angles [°] for **5**.

Ni(1)-C(21)	1.7849(19)	C(21)-Ni(1)-N(1)	167.33(8)
Ni(1)-N(1)	1.9687(14)	C(21)-Ni(1)-P(2)	97.05(6)
Ni(1)-P(2)	2.2892(5)	N(1)-Ni(1)-P(2)	84.32(4)
Ni(1)-P(1)	2.2919(5)	C(21)-Ni(1)-P(1)	98.32(6)
O(1)-C(21)	1.143(2)	N(1)-Ni(1)-P(1)	83.74(4)
P(1)-C(2)	1.7872(18)	P(2)-Ni(1)-P(1)	159.246(19)
P(1)-C(7)	1.8757(19)	C(2)-P(1)-C(7)	107.18(8)
P(1)-C(3)	1.8774(18)	C(2)-P(1)-C(3)	104.56(8)
N(1)-C(1)	1.367(2)	C(7)-P(1)-C(3)	112.46(8)
N(1)-C(11)	1.369(2)	C(2)-P(1)-Ni(1)	99.36(6)
C(1)-C(2)	1.349(3)	C(7)-P(1)-Ni(1)	117.17(6)
P(2)-C(12)	1.7873(17)	C(3)-P(1)-Ni(1)	114.10(6)
P(2)-C(13)	1.8788(19)	C(1)-N(1)-C(11)	118.74(15)
P(2)-C(17)	1.8807(18)	C(1)-N(1)-Ni(1)	120.13(12)
C(3)-C(6)	1.531(3)	C(11)-N(1)-Ni(1)	119.73(11)
C(3)-C(4)	1.535(3)	C(2)-C(1)-N(1)	122.31(16)
C(3)-C(5)	1.537(2)	C(12)-P(2)-C(13)	104.78(8)
C(8)-C(7)	1.532(3)	C(12)-P(2)-C(17)	104.95(8)
C(7)-C(10)	1.531(3)	C(13)-P(2)-C(17)	112.35(8)
C(7)-C(9)	1.537(3)	C(12)-P(2)-Ni(1)	98.94(6)
C(11)-C(12)	1.351(2)	C(13)-P(2)-Ni(1)	115.42(6)
C(13)-C(15)	1.531(3)	C(17)-P(2)-Ni(1)	117.83(6)
C(13)-C(14)	1.533(3)	C(1)-C(2)-P(1)	114.23(13)
C(13)-C(16)	1.535(3)	C(6)-C(3)-C(4)	109.05(16)
C(17)-C(18)	1.534(3)	C(6)-C(3)-C(5)	109.91(15)
C(17)-C(19)	1.534(3)	C(4)-C(3)-C(5)	107.60(15)
C(17)-C(20)	1.538(3)	C(6)-C(3)-P(1)	111.57(12)
		C(4)-C(3)-P(1)	104.73(12)
		C(5)-C(3)-P(1)	113.69(13)
		C(10)-C(7)-C(8)	110.18(17)
		C(10)-C(7)-C(9)	108.91(16)
		C(8)-C(7)-C(9)	108.28(16)
		C(10)-C(7)-P(1)	115.22(13)
		C(8)-C(7)-P(1)	108.75(13)
		C(9)-C(7)-P(1)	105.20(13)
		C(12)-C(11)-N(1)	122.13(16)
		C(11)-C(12)-P(2)	114.83(13)
		C(15)-C(13)-C(14)	109.16(17)
		C(15)-C(13)-C(16)	109.09(17)
		C(14)-C(13)-C(16)	108.09(17)
		C(15)-C(13)-P(2)	112.93(14)
		C(14)-C(13)-P(2)	112.01(13)
		C(16)-C(13)-P(2)	105.36(13)
		C(18)-C(17)-C(19)	109.88(16)
		C(18)-C(17)-C(20)	108.51(16)

C(19)-C(17)-C(20)	108.17(16)
C(18)-C(17)-P(2)	111.69(13)
C(19)-C(17)-P(2)	113.78(13)
C(20)-C(17)-P(2)	104.51(12)
O(1)-C(21)-Ni(1)	162.8(2)

Supplementary Table 32. Torsion angles [°] for **5**.

C(11)-N(1)-C(1)-C(2)	-166.21(17)
Ni(1)-N(1)-C(1)-C(2)	0.3(2)
N(1)-C(1)-C(2)-P(1)	3.6(2)
C(7)-P(1)-C(2)-C(1)	117.56(15)
C(3)-P(1)-C(2)-C(1)	-122.87(14)
Ni(1)-P(1)-C(2)-C(1)	-4.81(15)
C(2)-P(1)-C(3)-C(6)	-172.05(14)
C(7)-P(1)-C(3)-C(6)	-56.10(16)
Ni(1)-P(1)-C(3)-C(6)	80.47(14)
C(2)-P(1)-C(3)-C(4)	70.12(14)
C(7)-P(1)-C(3)-C(4)	-173.93(12)
Ni(1)-P(1)-C(3)-C(4)	-37.36(14)
C(2)-P(1)-C(3)-C(5)	-47.07(16)
C(7)-P(1)-C(3)-C(5)	68.89(16)
Ni(1)-P(1)-C(3)-C(5)	-154.54(12)
C(2)-P(1)-C(7)-C(10)	70.77(16)
C(3)-P(1)-C(7)-C(10)	-43.59(17)
Ni(1)-P(1)-C(7)-C(10)	-178.74(12)
C(2)-P(1)-C(7)-C(8)	-164.99(13)
C(3)-P(1)-C(7)-C(8)	80.64(15)
Ni(1)-P(1)-C(7)-C(8)	-54.50(15)
C(2)-P(1)-C(7)-C(9)	-49.17(14)
C(3)-P(1)-C(7)-C(9)	-163.54(12)
Ni(1)-P(1)-C(7)-C(9)	61.32(14)
C(1)-N(1)-C(11)-C(12)	167.69(17)
Ni(1)-N(1)-C(11)-C(12)	1.2(2)
N(1)-C(11)-C(12)-P(2)	-2.6(2)
C(13)-P(2)-C(12)-C(11)	121.80(15)
C(17)-P(2)-C(12)-C(11)	-119.69(14)
Ni(1)-P(2)-C(12)-C(11)	2.40(15)
C(12)-P(2)-C(13)-C(15)	63.66(16)
C(17)-P(2)-C(13)-C(15)	-49.72(17)
Ni(1)-P(2)-C(13)-C(15)	171.31(13)
C(12)-P(2)-C(13)-C(14)	-172.61(14)
C(17)-P(2)-C(13)-C(14)	74.01(16)
Ni(1)-P(2)-C(13)-C(14)	-64.96(15)
C(12)-P(2)-C(13)-C(16)	-55.32(15)
C(17)-P(2)-C(13)-C(16)	-168.70(13)
Ni(1)-P(2)-C(13)-C(16)	52.33(14)
C(12)-P(2)-C(17)-C(18)	-164.53(13)
C(13)-P(2)-C(17)-C(18)	-51.25(16)

Ni(1)-P(2)-C(17)-C(18)	86.64(14)
C(12)-P(2)-C(17)-C(19)	-39.42(16)
C(13)-P(2)-C(17)-C(19)	73.86(16)
Ni(1)-P(2)-C(17)-C(19)	-148.25(12)
C(12)-P(2)-C(17)-C(20)	78.36(13)
C(13)-P(2)-C(17)-C(20)	-168.36(12)
Ni(1)-P(2)-C(17)-C(20)	-30.47(14)
N(1)-Ni(1)-C(21)-O(1)	177.3(4)
P(2)-Ni(1)-C(21)-O(1)	81.8(6)
P(1)-Ni(1)-C(21)-O(1)	-84.2(6)

X-ray Single-Crystal Structure Analysis of **6**:

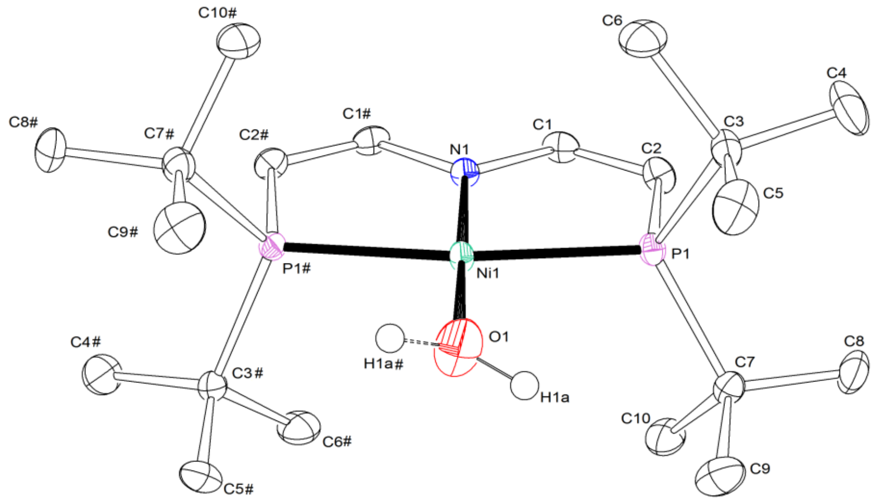

Supplementary Figure 52**.** Thermal ellipsoid plot of **6** with the anisotropic displacement parameters at the 50% probability level. All C-H hydrogen atoms are omitted for clarity. The O-H hydrogen was refined isotropically on calculated position by using a riding model with the Uiso value constrained to 1.2 Ueq. No hydrogen bond acceptor could be found. The asymmetric unit contains only a half complex molecule.

Supplementary Table 33. Crystal data and structure refinement for **6**.

Identification code	mo_SF_SF_250517_2_0m_a
Empirical formula	$C_{20}H_{41}NNiOP_2$
Formula weight	432.19
Temperature	100(2) K
Wavelength	0.71073 Å
Crystal system	Monoclinic

Space group	C2/c
Unit cell dimensions	a = 21.4757(14) Å α = 90°
	b = 7.8122(6) Å β = 102.380(3)°
	c = 13.9003(10) Å γ = 90°
Volume	2277.9(3) $Å^3$
Z	4
Density (calculated)	1.260 Mg/m^3
Absorption coefficient	1.001 mm^{-1}
F(000)	936
Crystal size	0.245 x 0.188 x 0.086 mm^3
Theta range for data collection	2.782 to 26.480°
Crystal shape and color	Block clear, intense red
Index ranges	-26<=h<=26, -9<=k<=9, -17<=l<=17
Reflections collected	21536
Independent reflections	2362 [R(int) = 0.1001]
Completeness to theta = 25.242°	100.0 %
Refinement method	Full-matrix least-squares on F^2
Data / restraints / parameters	2362 / 0 / 121
Goodness-of-fit on F^2	1.012
Final R indices [I>2sigma(I)]	R1 = 0.0381, wR2 = 0.0723
R indices (all data)	R1 = 0.0640, wR2 = 0.0795
Largest diff. peak and hole	0.648 and -0.284 $eÅ^{-3}$

Supplementary Table 34. Bond lengths [Å] and angles [°] for **6**.

Ni(1)-O(1)	1.845(3)	O(1)-Ni(1)-P(1)#1	94.970(19)
Ni(1)-N(1)	1.896(3)	N(1)-Ni(1)-P(1)#1	85.030(18)
Ni(1)-P(1)#1	2.2195(6)	O(1)-Ni(1)-P(1)	94.969(18)
Ni(1)-P(1)	2.2195(6)	N(1)-Ni(1)-P(1)	85.031(19)
P(1)-C(2)	1.784(2)	P(1)#1-Ni(1)-P(1)	170.06(4)
P(1)-C(7)	1.873(2)	C(2)-P(1)-C(7)	106.76(11)
P(1)-C(3)	1.874(2)	C(2)-P(1)-C(3)	105.31(11)
O(1)-H(1A)	0.8400	C(7)-P(1)-C(3)	113.56(11)
N(1)-C(1)	1.373(3)	C(2)-P(1)-Ni(1)	100.21(8)
N(1)-C(1)#1	1.373(3)	C(7)-P(1)-Ni(1)	117.47(8)
C(1)-C(2)	1.348(3)	C(3)-P(1)-Ni(1)	111.72(7)
C(3)-C(4)	1.521(3)	C(1)-N(1)-C(1)#1	118.8(3)
C(3)-C(5)	1.531(3)	C(1)-N(1)-Ni(1)	120.61(14)
C(3)-C(6)	1.535(3)	C(1)#1-N(1)-Ni(1)	120.62(14)
C(10)-C(7)	1.534(3)	C(2)-C(1)-N(1)	121.6(2)
C(9)-C(7)	1.539(3)	C(1)-C(2)-P(1)	112.45(18)
C(8)-C(7)	1.521(3)	C(4)-C(3)-C(5)	110.1(2)
		C(4)-C(3)-C(6)	108.5(2)
		C(5)-C(3)-C(6)	108.2(2)
		C(4)-C(3)-P(1)	113.57(16)
		C(5)-C(3)-P(1)	111.62(16)
		C(6)-C(3)-P(1)	104.49(16)

C(8)-C(7)-C(10)	109.4(2)
C(8)-C(7)-C(9)	109.9(2)
C(10)-C(7)-C(9)	107.7(2)
C(8)-C(7)-P(1)	113.54(16)
C(10)-C(7)-P(1)	104.72(16)
C(9)-C(7)-P(1)	111.32(17)

Symmetry transformations used to generate equivalent atoms:
#1 -x+1,y,-z+3/2

Supplementary Table 35. Torsion angles [°] for **6**.

P(1)#1-Ni(1)-N(1)-C(1)	-178.20(11)
P(1)-Ni(1)-N(1)-C(1)	1.80(11)
P(1)#1-Ni(1)-N(1)-C(1)#1	1.80(11)
P(1)-Ni(1)-N(1)-C(1)#1	-178.20(11)
C(1)#1-N(1)-C(1)-C(2)	179.2(3)
Ni(1)-N(1)-C(1)-C(2)	-0.8(3)
N(1)-C(1)-C(2)-P(1)	-1.2(3)
C(7)-P(1)-C(2)-C(1)	125.11(18)
C(3)-P(1)-C(2)-C(1)	-113.87(18)
Ni(1)-P(1)-C(2)-C(1)	2.17(18)
C(2)-P(1)-C(3)-C(4)	-52.4(2)
C(7)-P(1)-C(3)-C(4)	64.1(2)
Ni(1)-P(1)-C(3)-C(4)	-160.26(17)
C(2)-P(1)-C(3)-C(5)	-177.63(17)
C(7)-P(1)-C(3)-C(5)	-61.17(19)
Ni(1)-P(1)-C(3)-C(5)	74.50(17)
C(2)-P(1)-C(3)-C(6)	65.69(18)
C(7)-P(1)-C(3)-C(6)	-177.85(16)
Ni(1)-P(1)-C(3)-C(6)	-42.18(18)
C(2)-P(1)-C(7)-C(8)	61.2(2)
C(3)-P(1)-C(7)-C(8)	-54.4(2)
Ni(1)-P(1)-C(7)-C(8)	172.65(14)
C(2)-P(1)-C(7)-C(10)	-58.05(18)
C(3)-P(1)-C(7)-C(10)	-173.65(16)
Ni(1)-P(1)-C(7)-C(10)	53.37(18)
C(2)-P(1)-C(7)-C(9)	-174.12(17)
C(3)-P(1)-C(7)-C(9)	70.28(19)
Ni(1)-P(1)-C(7)-C(9)	-62.70(18)

Symmetry transformations used to generate equivalent atoms:
#1 -x+1,y,-z+3/2

X-ray Single-Crystal Structure Analysis of **7**:

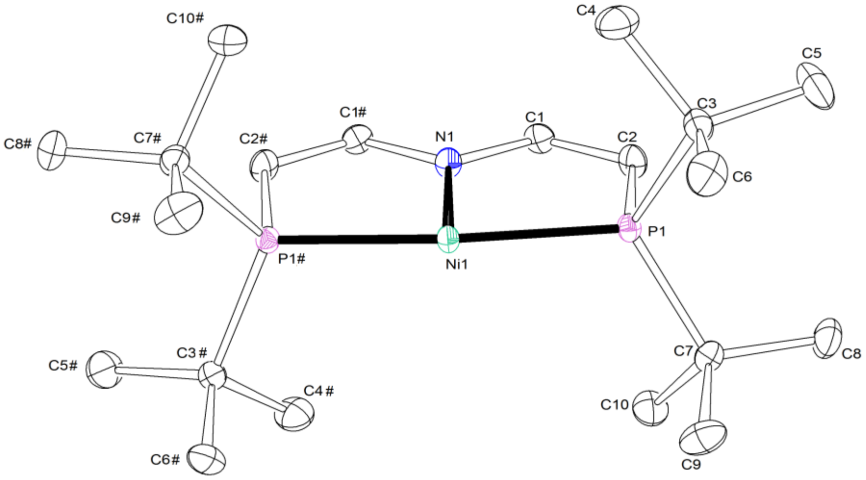

Supplementary Figure 53**.** Thermal ellipsoid plot of **7** with the anisotropic displacement parameters at the 50% probability level. All C-H hydrogen atoms are omitted for clarity. The asymmetric unit contains only a half complex molecule.

Supplementary Table 36. Crystal data and structure refinement for **7**.

Identification code	mo_SF_SF_030417_0m_a	
Empirical formula	$C_{20}H_{40}NNiP_2$	
Formula weight	415.18	
Temperature	100(2) K	
Wavelength	0.71073 Å	
Crystal system	Monoclinic	
Space group	C2/c	
Unit cell dimensions	a = 21.5733(10) Å	α = 90°
	b = 7.6788(3) Å	β = 102.388(2)°
	c = 14.0606(7) Å	γ = 90°
Volume	2275.01(18) $Å^3$	
Z	4	
Density (calculated)	1.212 Mg/m^3	
Absorption coefficient	0.996 mm^{-1}	
F(000)	900	
Crystal size	0.192 x 0.137 x 0.099 mm^3	
Crystal shape and color	Block clear, dark orange	
Theta range for data collection	2.823 to 34.851°	
Index ranges	-34<=h<=34, -12<=k<=12, -22<=l<=21	
Reflections collected	54032	
Independent reflections	4966 [R(int) = 0.0677]	
Completeness to theta = 25.242°	100.0 %	
Refinement method	Full-matrix least-squares on F^2	
Data / restraints / parameters	4966 / 0 / 116	

Goodness-of-fit on F^2	1.043
Final R indices [I>2sigma(I)]	R1 = 0.0339, wR2 = 0.0648
R indices (all data)	R1 = 0.0579, wR2 = 0.0710
Largest diff. peak and hole	0.476 and -0.295 eÅ^{-3}

Supplementary Table 37. Bond lengths [Å] and angles [°] for **7**.

Ni(1)-N(1)	1.9337(12)	N(1)-Ni(1)-P(1)#1	86.783(8)
Ni(1)-P(1)#1	2.2125(3)	N(1)-Ni(1)-P(1)	86.782(8)
Ni(1)-P(1)	2.2125(3)	P(1)#1-Ni(1)-P(1)	173.565(16)
P(1)-C(2)	1.7935(11)	C(2)-P(1)-C(7)	105.77(5)
P(1)-C(7)	1.8729(11)	C(2)-P(1)-C(3)	104.61(5)
P(1)-C(3)	1.8771(11)	C(7)-P(1)-C(3)	113.18(5)
N(1)-C(1)	1.3586(12)	C(2)-P(1)-Ni(1)	98.28(4)
N(1)-C(1)#1	1.3586(12)	C(7)-P(1)-Ni(1)	118.70(4)
C(1)-C(2)	1.3588(15)	C(3)-P(1)-Ni(1)	113.65(4)
C(3)-C(6)	1.5273(16)	C(1)-N(1)-C(1)#1	122.43(13)
C(3)-C(5)	1.5292(16)	C(1)-N(1)-Ni(1)	118.79(6)
C(3)-C(4)	1.5358(17)	C(1)#1-N(1)-Ni(1)	118.79(6)
C(10)-C(7)	1.5377(16)	N(1)-C(1)-C(2)	121.26(10)
C(9)-C(7)	1.5321(15)	C(1)-C(2)-P(1)	114.83(8)
C(8)-C(7)	1.5272(16)	C(6)-C(3)-C(5)	110.08(10)
		C(6)-C(3)-C(4)	108.47(10)
		C(5)-C(3)-C(4)	108.42(10)
		C(6)-C(3)-P(1)	111.13(8)
		C(5)-C(3)-P(1)	114.38(8)
		C(4)-C(3)-P(1)	104.02(8)
		C(8)-C(7)-C(9)	110.34(10)
		C(8)-C(7)-C(10)	108.75(9)
		C(9)-C(7)-C(10)	107.70(10)
		C(8)-C(7)-P(1)	114.65(8)
		C(9)-C(7)-P(1)	110.32(8)
		C(10)-C(7)-P(1)	104.71(7)

Symmetry transformations used to generate equivalent atoms:
#1 -x+1,y,-z+3/2

Supplementary Table 38. Torsion angles [°] for **7**.

C(1)#1-N(1)-C(1)-C(2)	178.97(12)
Ni(1)-N(1)-C(1)-C(2)	-1.04(12)
N(1)-C(1)-C(2)-P(1)	-0.91(14)
C(7)-P(1)-C(2)-C(1)	125.04(9)
C(3)-P(1)-C(2)-C(1)	-115.20(9)
Ni(1)-P(1)-C(2)-C(1)	1.99(9)
C(2)-P(1)-C(3)-C(6)	-179.17(8)
C(7)-P(1)-C(3)-C(6)	-64.51(9)
Ni(1)-P(1)-C(3)-C(6)	74.77(8)

C(2)-P(1)-C(3)-C(5)	-53.77(10)
C(7)-P(1)-C(3)-C(5)	60.89(11)
Ni(1)-P(1)-C(3)-C(5)	-159.83(8)
C(2)-P(1)-C(3)-C(4)	64.31(8)
C(7)-P(1)-C(3)-C(4)	178.96(7)
Ni(1)-P(1)-C(3)-C(4)	-41.76(8)
C(2)-P(1)-C(7)-C(8)	61.35(9)
C(3)-P(1)-C(7)-C(8)	-52.61(9)
Ni(1)-P(1)-C(7)-C(8)	170.33(7)
C(2)-P(1)-C(7)-C(9)	-173.36(8)
C(3)-P(1)-C(7)-C(9)	72.68(9)
Ni(1)-P(1)-C(7)-C(9)	-64.38(9)
C(2)-P(1)-C(7)-C(10)	-57.73(8)
C(3)-P(1)-C(7)-C(10)	-171.70(7)
Ni(1)-P(1)-C(7)-C(10)	51.24(8)

Symmetry transformations used to generate equivalent atoms:
#1 -x+1,y,-z+3/2

X-ray Single-Crystal Structure Analysis of **9**:

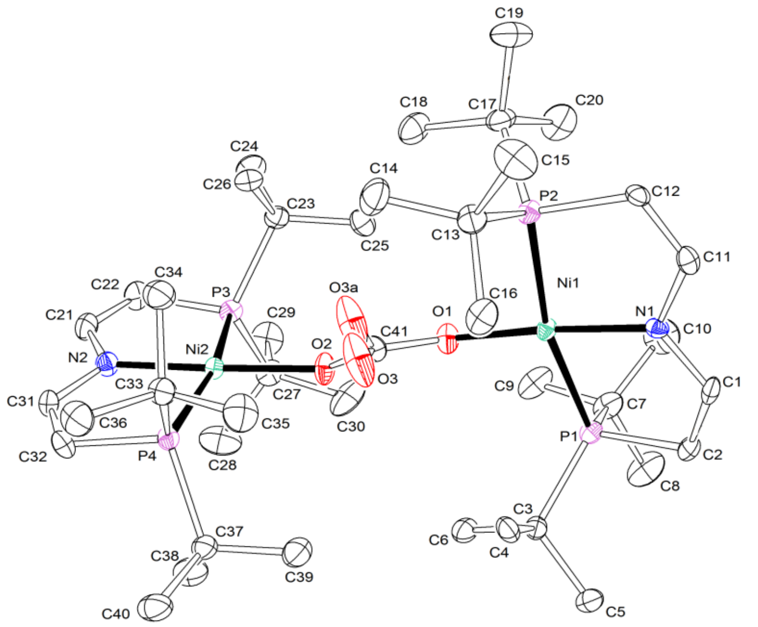

Supplementary Figure 54**.** Thermal ellipsoid plot of **9** with the anisotropic displacement parameters at the 50% probability level. All C-H hydrogen atoms are omitted for clarity. Disorder of O3 and O3a was refined with fixed population of 0.5 for both sites using PART and EADP commands.

Supplementary Table 39. Crystal data and structure refinement for **9**.

Identification code	mo_SF_SF_090617_0m_a	
Empirical formula	$C_{41}H_{80}N_2Ni_2O_3P_4$	
Formula weight	890.37	
Temperature	100(2) K	
Wavelength	0.71073 Å	
Crystal system	Triclinic	
Space group	P-1	
Unit cell dimensions	a = 12.0918(7) Å	α = 77.041(4)°
	b = 13.3064(8) Å	β = 87.050(4)°
	c = 16.5140(9) Å	γ = 66.641(4)°
Volume	2375.1(2) $Å^3$	
Z	2	
Density (calculated)	1.245 Mg/m^3	
Absorption coefficient	0.963 mm^{-1}	
F(000)	960	
Crystal size	0.152 x 0.081 x 0.039 mm^3	
Crystal shape and color	Plate clear, orange	
Theta range for data collection	2.274 to 26.461°	
Index ranges	-15<=h<=15, -16<=k<=16, -20<=l<=20	
Reflections collected	47157	
Independent reflections	9744 [R(int) = 0.1590]	
Completeness to theta = 25.242°	99.9 %	
Refinement method	Full-matrix least-squares on F^2	
Data / restraints / parameters	9744 / 0 / 496	
Goodness-of-fit on F^2	1.003	
Final R indices [I>2sigma(I)]	R1 = 0.0514, wR2 = 0.0850	
R indices (all data)	R1 = 0.1181, wR2 = 0.1029	
Largest diff. peak and hole	0.567 and -0.584 $eÅ^{-3}$	

Supplementary Table **40**. Bond lengths [Å] and angles [°] for **9**.

Ni(1)-O(1)	1.888(3)	O(1)-Ni(1)-N(1)	172.21(11)
Ni(1)-N(1)	1.904(3)	O(1)-Ni(1)-P(1)	88.97(8)
Ni(1)-P(1)	2.2267(11)	N(1)-Ni(1)-P(1)	83.70(9)
Ni(1)-P(2)	2.2871(11)	O(1)-Ni(1)-P(2)	103.29(8)
O(1)-C(41)	1.313(4)	N(1)-Ni(1)-P(2)	83.88(9)
P(1)-C(2)	1.777(4)	P(1)-Ni(1)-P(2)	167.21(4)
P(1)-C(3)	1.866(4)	C(41)-O(1)-Ni(1)	128.6(2)
P(1)-C(7)	1.877(4)	C(2)-P(1)-C(3)	105.74(17)
N(1)-C(1)	1.369(4)	C(2)-P(1)-C(7)	105.69(18)
N(1)-C(11)	1.374(4)	C(3)-P(1)-C(7)	114.01(18)
C(1)-C(2)	1.334(5)	C(2)-P(1)-Ni(1)	100.71(13)
Ni(2)-O(2)	1.882(2)	C(3)-P(1)-Ni(1)	114.90(12)
Ni(2)-N(2)	1.895(3)	C(7)-P(1)-Ni(1)	114.00(12)
Ni(2)-P(3)	2.2238(11)	C(1)-N(1)-C(11)	118.2(3)
Ni(2)-P(4)	2.2823(11)	C(1)-N(1)-Ni(1)	120.7(2)

O(2)-C(41)	1.298(4)
P(2)-C(12)	1.779(4)
P(2)-C(17)	1.859(4)
P(2)-C(13)	1.887(4)
N(2)-C(21)	1.366(5)
N(2)-C(31)	1.371(5)
C(3)-C(6)	1.529(5)
C(3)-C(5)	1.532(5)
C(3)-C(4)	1.535(5)
O(3)-C(41)	1.23(2)
P(3)-C(22)	1.779(4)
P(3)-C(23)	1.862(4)
P(3)-C(27)	1.872(4)
P(4)-C(32)	1.783(4)
P(4)-C(37)	1.868(4)
P(4)-C(33)	1.891(4)
C(7)-C(9)	1.525(5)
C(7)-C(8)	1.531(5)
C(7)-C(10)	1.541(6)
C(11)-C(12)	1.329(5)
C(14)-C(13)	1.527(5)
C(13)-C(16)	1.529(5)
C(13)-C(15)	1.539(5)
C(18)-C(17)	1.531(5)
C(17)-C(19)	1.535(5)
C(17)-C(20)	1.536(5)
C(21)-C(22)	1.329(5)
C(23)-C(25)	1.532(5)
C(23)-C(24)	1.533(5)
C(23)-C(26)	1.540(5)
C(27)-C(30)	1.525(5)
C(27)-C(29)	1.526(5)
C(27)-C(28)	1.540(6)
C(31)-C(32)	1.327(5)
C(33)-C(35)	1.526(5)
C(33)-C(34)	1.533(5)
C(33)-C(36)	1.534(5)
C(37)-C(40)	1.524(5)
C(37)-C(39)	1.526(5)
C(37)-C(38)	1.542(5)
O(3A)-C(41)	1.26(2)

C(11)-N(1)-Ni(1)	120.9(2)
C(2)-C(1)-N(1)	122.2(3)
O(2)-Ni(2)-N(2)	170.92(12)
O(2)-Ni(2)-P(3)	87.21(8)
N(2)-Ni(2)-P(3)	84.04(10)
O(2)-Ni(2)-P(4)	104.31(8)
N(2)-Ni(2)-P(4)	84.26(10)
P(3)-Ni(2)-P(4)	167.81(4)
C(41)-O(2)-Ni(2)	132.3(2)
C(12)-P(2)-C(17)	105.25(18)
C(12)-P(2)-C(13)	102.17(17)
C(17)-P(2)-C(13)	113.77(17)
C(12)-P(2)-Ni(1)	98.25(12)
C(17)-P(2)-Ni(1)	115.18(12)
C(13)-P(2)-Ni(1)	118.75(13)
C(21)-N(2)-C(31)	118.2(3)
C(21)-N(2)-Ni(2)	120.7(3)
C(31)-N(2)-Ni(2)	121.0(3)
C(1)-C(2)-P(1)	111.8(3)
C(6)-C(3)-C(5)	110.2(3)
C(6)-C(3)-C(4)	109.6(3)
C(5)-C(3)-C(4)	108.6(3)
C(6)-C(3)-P(1)	112.2(3)
C(5)-C(3)-P(1)	112.4(3)
C(4)-C(3)-P(1)	103.6(2)
C(22)-P(3)-C(23)	106.63(18)
C(22)-P(3)-C(27)	105.80(18)
C(23)-P(3)-C(27)	114.16(18)
C(22)-P(3)-Ni(2)	100.53(13)
C(23)-P(3)-Ni(2)	113.77(12)
C(27)-P(3)-Ni(2)	114.30(12)
C(32)-P(4)-C(37)	104.34(18)
C(32)-P(4)-C(33)	102.81(18)
C(37)-P(4)-C(33)	113.04(17)
C(32)-P(4)-Ni(2)	98.38(14)
C(37)-P(4)-Ni(2)	112.29(12)
C(33)-P(4)-Ni(2)	122.45(13)
C(9)-C(7)-C(8)	109.8(3)
C(9)-C(7)-C(10)	108.6(3)
C(8)-C(7)-C(10)	109.3(3)
C(9)-C(7)-P(1)	111.6(3)
C(8)-C(7)-P(1)	112.6(3)
C(10)-C(7)-P(1)	104.8(3)
C(12)-C(11)-N(1)	121.6(3)
C(11)-C(12)-P(2)	114.1(3)
C(14)-C(13)-C(16)	109.3(3)
C(14)-C(13)-C(15)	108.9(3)
C(16)-C(13)-C(15)	108.4(3)
C(14)-C(13)-P(2)	113.6(3)
C(16)-C(13)-P(2)	104.0(2)
C(15)-C(13)-P(2)	112.4(3)

C(18)-C(17)-C(19)	110.2(3)
C(18)-C(17)-C(20)	108.3(3)
C(19)-C(17)-C(20)	109.1(4)
C(18)-C(17)-P(2)	110.6(3)
C(19)-C(17)-P(2)	113.2(3)
C(20)-C(17)-P(2)	105.1(3)
C(22)-C(21)-N(2)	122.6(4)
C(21)-C(22)-P(3)	111.7(3)
C(25)-C(23)-C(24)	109.7(3)
C(25)-C(23)-C(26)	110.0(3)
C(24)-C(23)-C(26)	108.4(3)
C(25)-C(23)-P(3)	112.5(3)
C(24)-C(23)-P(3)	112.1(3)
C(26)-C(23)-P(3)	103.9(3)
C(30)-C(27)-C(29)	110.2(3)
C(30)-C(27)-C(28)	108.4(4)
C(29)-C(27)-C(28)	108.1(3)
C(30)-C(27)-P(3)	111.5(3)
C(29)-C(27)-P(3)	113.3(3)
C(28)-C(27)-P(3)	105.0(3)
C(32)-C(31)-N(2)	122.1(4)
C(31)-C(32)-P(4)	113.9(3)
C(35)-C(33)-C(34)	109.7(3)
C(35)-C(33)-C(36)	108.5(3)
C(34)-C(33)-C(36)	108.6(3)
C(35)-C(33)-P(4)	114.1(3)
C(34)-C(33)-P(4)	104.1(3)
C(36)-C(33)-P(4)	111.7(3)
C(40)-C(37)-C(39)	110.5(3)
C(40)-C(37)-C(38)	108.9(3)
C(39)-C(37)-C(38)	108.5(3)
C(40)-C(37)-P(4)	113.1(3)
C(39)-C(37)-P(4)	110.7(3)
C(38)-C(37)-P(4)	105.0(3)
O(3)-C(41)-O(2)	120.7(11)
O(3A)-C(41)-O(2)	122.3(11)
O(3)-C(41)-O(1)	122.2(11)
O(3A)-C(41)-O(1)	121.5(11)
O(2)-C(41)-O(1)	114.3(3)

Supplementary Table 41. Torsion angles [°] for **9**.

P(1)-Ni(1)-O(1)-C(41)	123.3(3)
P(2)-Ni(1)-O(1)-C(41)	-60.4(3)
C(11)-N(1)-C(1)-C(2)	-170.9(3)
Ni(1)-N(1)-C(1)-C(2)	5.5(5)
P(3)-Ni(2)-O(2)-C(41)	132.3(3)
P(4)-Ni(2)-O(2)-C(41)	-51.7(3)
P(3)-Ni(2)-N(2)-C(21)	-5.7(3)

P(4)-Ni(2)-N(2)-C(21)	177.8(3)
P(3)-Ni(2)-N(2)-C(31)	171.0(3)
P(4)-Ni(2)-N(2)-C(31)	-5.5(3)
N(1)-C(1)-C(2)-P(1)	2.6(5)
C(3)-P(1)-C(2)-C(1)	-127.5(3)
C(7)-P(1)-C(2)-C(1)	111.3(3)
Ni(1)-P(1)-C(2)-C(1)	-7.6(3)
C(2)-P(1)-C(3)-C(6)	-163.6(2)
C(7)-P(1)-C(3)-C(6)	-47.9(3)
Ni(1)-P(1)-C(3)-C(6)	86.3(3)
C(2)-P(1)-C(3)-C(5)	-38.8(3)
C(7)-P(1)-C(3)-C(5)	76.9(3)
Ni(1)-P(1)-C(3)-C(5)	-148.9(2)
C(2)-P(1)-C(3)-C(4)	78.3(3)
C(7)-P(1)-C(3)-C(4)	-166.1(2)
Ni(1)-P(1)-C(3)-C(4)	-31.8(3)
C(2)-P(1)-C(7)-C(9)	-173.5(3)
C(3)-P(1)-C(7)-C(9)	70.8(3)
Ni(1)-P(1)-C(7)-C(9)	-63.8(3)
C(2)-P(1)-C(7)-C(8)	62.5(3)
C(3)-P(1)-C(7)-C(8)	-53.2(3)
Ni(1)-P(1)-C(7)-C(8)	172.2(3)
C(2)-P(1)-C(7)-C(10)	-56.2(3)
C(3)-P(1)-C(7)-C(10)	-171.9(3)
Ni(1)-P(1)-C(7)-C(10)	53.5(3)
C(1)-N(1)-C(11)-C(12)	-178.6(3)
Ni(1)-N(1)-C(11)-C(12)	4.9(5)
N(1)-C(11)-C(12)-P(2)	4.7(5)
C(17)-P(2)-C(12)-C(11)	-128.5(3)
C(13)-P(2)-C(12)-C(11)	112.4(3)
Ni(1)-P(2)-C(12)-C(11)	-9.5(3)
C(12)-P(2)-C(13)-C(14)	156.4(3)
C(17)-P(2)-C(13)-C(14)	43.6(3)
Ni(1)-P(2)-C(13)-C(14)	-97.0(3)
C(12)-P(2)-C(13)-C(16)	-84.9(3)
C(17)-P(2)-C(13)-C(16)	162.3(2)
Ni(1)-P(2)-C(13)-C(16)	21.7(3)
C(12)-P(2)-C(13)-C(15)	32.2(3)
C(17)-P(2)-C(13)-C(15)	-80.7(3)
Ni(1)-P(2)-C(13)-C(15)	138.8(2)
C(12)-P(2)-C(17)-C(18)	177.7(3)
C(13)-P(2)-C(17)-C(18)	-71.3(3)
Ni(1)-P(2)-C(17)-C(18)	70.7(3)
C(12)-P(2)-C(17)-C(19)	-58.0(3)
C(13)-P(2)-C(17)-C(19)	53.0(3)
Ni(1)-P(2)-C(17)-C(19)	-165.0(3)
C(12)-P(2)-C(17)-C(20)	61.0(3)
C(13)-P(2)-C(17)-C(20)	172.0(3)
Ni(1)-P(2)-C(17)-C(20)	-46.0(3)
C(31)-N(2)-C(21)-C(22)	-174.0(4)
Ni(2)-N(2)-C(21)-C(22)	2.8(5)

N(2)-C(21)-C(22)-P(3)	3.3(5)
C(23)-P(3)-C(22)-C(21)	-125.3(3)
C(27)-P(3)-C(22)-C(21)	112.8(3)
Ni(2)-P(3)-C(22)-C(21)	-6.3(3)
C(22)-P(3)-C(23)-C(25)	-164.4(3)
C(27)-P(3)-C(23)-C(25)	-47.9(3)
Ni(2)-P(3)-C(23)-C(25)	85.8(3)
C(22)-P(3)-C(23)-C(24)	-40.1(3)
C(27)-P(3)-C(23)-C(24)	76.3(3)
Ni(2)-P(3)-C(23)-C(24)	-150.0(2)
C(22)-P(3)-C(23)-C(26)	76.7(3)
C(27)-P(3)-C(23)-C(26)	-166.8(2)
Ni(2)-P(3)-C(23)-C(26)	-33.1(3)
C(22)-P(3)-C(27)-C(30)	-174.3(3)
C(23)-P(3)-C(27)-C(30)	68.8(3)
Ni(2)-P(3)-C(27)-C(30)	-64.7(3)
C(22)-P(3)-C(27)-C(29)	60.7(3)
C(23)-P(3)-C(27)-C(29)	-56.3(3)
Ni(2)-P(3)-C(27)-C(29)	170.3(3)
C(22)-P(3)-C(27)-C(28)	-57.1(3)
C(23)-P(3)-C(27)-C(28)	-174.0(3)
Ni(2)-P(3)-C(27)-C(28)	52.5(3)
C(21)-N(2)-C(31)-C(32)	-179.7(4)
Ni(2)-N(2)-C(31)-C(32)	3.5(5)
N(2)-C(31)-C(32)-P(4)	2.0(5)
C(37)-P(4)-C(32)-C(31)	-120.8(3)
C(33)-P(4)-C(32)-C(31)	121.0(3)
Ni(2)-P(4)-C(32)-C(31)	-5.1(3)
C(32)-P(4)-C(33)-C(35)	154.7(3)
C(37)-P(4)-C(33)-C(35)	42.8(3)
Ni(2)-P(4)-C(33)-C(35)	-96.5(3)
C(32)-P(4)-C(33)-C(34)	-85.7(3)
C(37)-P(4)-C(33)-C(34)	162.4(2)
Ni(2)-P(4)-C(33)-C(34)	23.1(3)
C(32)-P(4)-C(33)-C(36)	31.3(3)
C(37)-P(4)-C(33)-C(36)	-80.6(3)
Ni(2)-P(4)-C(33)-C(36)	140.1(2)
C(32)-P(4)-C(37)-C(40)	-58.6(3)
C(33)-P(4)-C(37)-C(40)	52.3(3)
Ni(2)-P(4)-C(37)-C(40)	-164.1(3)
C(32)-P(4)-C(37)-C(39)	176.8(3)
C(33)-P(4)-C(37)-C(39)	-72.2(3)
Ni(2)-P(4)-C(37)-C(39)	71.3(3)
C(32)-P(4)-C(37)-C(38)	60.0(3)
C(33)-P(4)-C(37)-C(38)	170.9(3)
Ni(2)-P(4)-C(37)-C(38)	-45.5(3)
Ni(2)-O(2)-C(41)-O(3)	34.6(9)
Ni(2)-O(2)-C(41)-O(3A)	0.9(9)
Ni(2)-O(2)-C(41)-O(1)	-163.9(2)
Ni(1)-O(1)-C(41)-O(3)	-5.2(8)
Ni(1)-O(1)-C(41)-O(3A)	28.6(9)

Ni(1)-O(1)-C(41)-O(2)	-166.5(2)

X-ray Single-Crystal Structure Analysis of **10**:

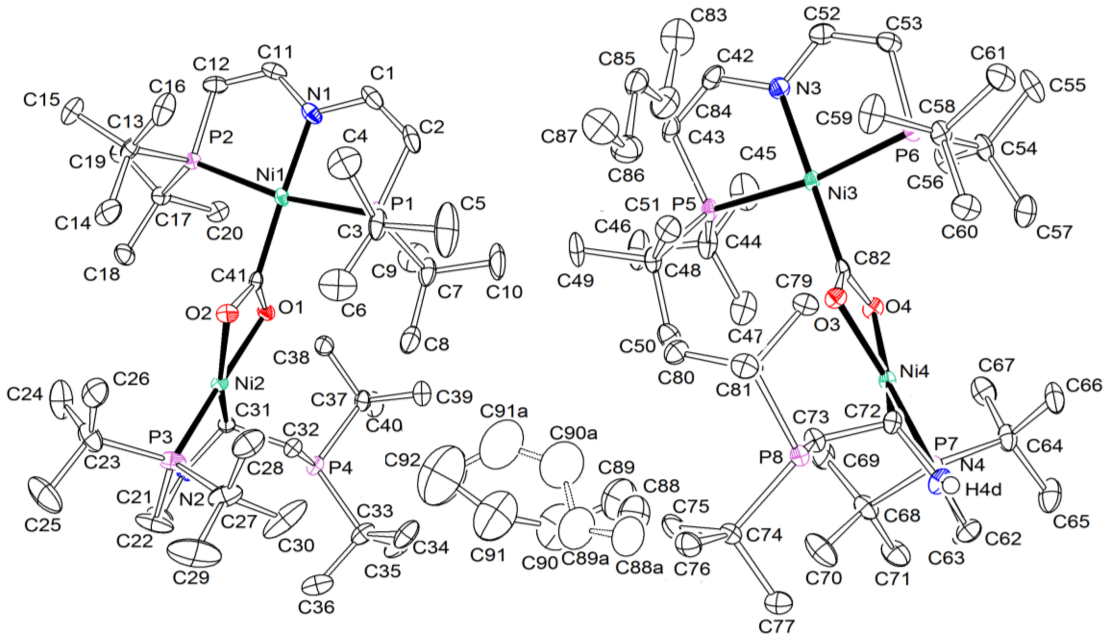

Supplementary Figure 55. Thermal ellipsoid plot of **10** with the anisotropic displacement parameters at the 50% probability level. All C-H hydrogen atoms are omitted for clarity. The N-H hydrogen atom was found from residual density map and isotropically refined. The asymmetric unit contains two complex molecules and two pentane solvent molecules. One disordered pentane solvent molecule was refined with population of 0.584(4) on the main domain using some restraints and constraints (SADI, RIGU, EADP).

Supplementary Table 42. Crystal data and structure refinement for **10**.

Identification code	SF_SF_120617	
Empirical formula	$C_{41}H_{82}N_2Ni_2O_2P_4 * C_5H_{12}$	
Formula weight	948.53	
Temperature	100(2) K	
Wavelength	0.71073 Å	
Crystal system	Triclinic	
Space group	P-1	
Unit cell dimensions	a = 11.5412(5) Å	α = 93.693(2)°
	b = 19.3558(8) Å	β = 97.673(2)°
	c = 24.1966(10) Å	γ = 96.435(2)°
Volume	5305.8(4) $Å^3$	
Z	4	
Density (calculated)	1.187 Mg/m^3	

Absorption coefficient	0.865 mm^{-1}
F(000)	2064
Crystal size	0.276 x 0.144 x 0.087 mm^3
Crystal shape and color	Block, clear orange
Theta range for data collection	2.194 to 28.368°
Index ranges	-15<=h<=14, -25<=k<=25, -32<=l<=32
Reflections collected	183629
Independent reflections	26493 [R(int) = 0.0898]
Completeness to theta = 25.242°	99.9 %
Refinement method	Full-matrix least-squares on F2
Data / restraints / parameters	26493 / 70 / 1083
Goodness-of-fit on F^2	1.048
Final R indices [I>2sigma(I)]	R1 = 0.0481, wR2 = 0.0975
R indices (all data)	R1 = 0.0776, wR2 = 0.1082
Largest diff. peak and hole	1.222 and -1.068 eÅ^{-3}

Supplementary Table 43. Bond lengths [Å] and angles [°] for **10**.

Ni(1)-C(41)	1.875(3)	C(41)-Ni(1)-N(1)	171.21(10)
Ni(1)-N(1)	1.924(2)	C(41)-Ni(1)-P(1)	96.03(8)
Ni(1)-P(1)	2.2112(8)	N(1)-Ni(1)-P(1)	85.40(7)
Ni(1)-P(2)	2.2120(7)	C(41)-Ni(1)-P(2)	94.62(8)
Ni(2)-C(31)	1.919(2)	N(1)-Ni(1)-P(2)	84.69(7)
Ni(2)-O(1)	1.9500(18)	P(1)-Ni(1)-P(2)	168.59(3)
Ni(2)-O(2)	2.0056(18)	C(31)-Ni(2)-O(1)	92.56(9)
Ni(2)-P(3)	2.1305(8)	C(31)-Ni(2)-O(2)	158.51(9)
Ni(2)-C(41)	2.356(3)	O(1)-Ni(2)-O(2)	66.09(7)
Ni(3)-C(82)	1.869(3)	C(31)-Ni(2)-P(3)	94.72(8)
Ni(3)-N(3)	1.925(2)	O(1)-Ni(2)-P(3)	171.82(6)
Ni(3)-P(5)	2.2214(7)	O(2)-Ni(2)-P(3)	106.75(6)
Ni(3)-P(6)	2.2247(7)	C(31)-Ni(2)-C(41)	125.63(10)
Ni(4)-C(72)	1.921(3)	O(1)-Ni(2)-C(41)	33.27(8)
Ni(4)-O(3)	1.9608(18)	O(2)-Ni(2)-C(41)	32.91(8)
Ni(4)-O(4)	2.0062(18)	P(3)-Ni(2)-C(41)	139.65(7)
Ni(4)-P(7)	2.1379(7)	C(82)-Ni(3)-N(3)	175.85(10)
Ni(4)-C(82)	2.362(3)	C(82)-Ni(3)-P(5)	96.16(8)
P(1)-C(2)	1.788(3)	N(3)-Ni(3)-P(5)	84.93(7)
P(1)-C(7)	1.867(3)	C(82)-Ni(3)-P(6)	94.05(8)
P(1)-C(3)	1.878(3)	N(3)-Ni(3)-P(6)	85.01(7)
P(2)-C(12)	1.778(3)	P(5)-Ni(3)-P(6)	169.65(3)
P(2)-C(17)	1.871(3)	C(72)-Ni(4)-O(3)	92.60(9)
P(2)-C(13)	1.872(3)	C(72)-Ni(4)-O(4)	157.83(9)
P(3)-C(22)	1.768(3)	O(3)-Ni(4)-O(4)	66.05(7)
P(3)-C(27)	1.878(3)	C(72)-Ni(4)-P(7)	95.57(8)
P(3)-C(23)	1.885(3)	O(3)-Ni(4)-P(7)	170.37(6)
P(4)-C(32)	1.862(3)	O(4)-Ni(4)-P(7)	106.27(6)
P(4)-C(37)	1.885(3)	C(72)-Ni(4)-C(82)	125.33(10)

P(4)-C(33)	1.891(3)
P(5)-C(43)	1.786(3)
P(5)-C(48)	1.871(3)
P(5)-C(44)	1.880(3)
P(6)-C(53)	1.784(3)
P(6)-C(54)	1.877(3)
P(6)-C(58)	1.880(3)
P(7)-C(63)	1.766(3)
P(7)-C(64)	1.878(3)
P(7)-C(68)	1.885(3)
P(8)-C(73)	1.865(3)
P(8)-C(74)	1.888(3)
P(8)-C(78)	1.889(3)
O(1)-C(41)	1.292(3)
O(2)-C(41)	1.280(3)
O(3)-C(82)	1.291(3)
O(4)-C(82)	1.285(3)
N(1)-C(11)	1.363(4)
N(1)-C(1)	1.366(4)
N(2)-C(21)	1.336(4)
N(2)-C(31)	1.448(3)
N(2)-H(2A)	0.87(3)
N(3)-C(52)	1.356(4)
N(3)-C(42)	1.372(3)
N(4)-C(62)	1.336(4)
N(4)-C(72)	1.451(3)
N(4)-H(4D)	0.80(3)
C(1)-C(2)	1.344(4)
C(3)-C(5)	1.514(4)
C(3)-C(4)	1.521(4)
C(3)-C(6)	1.537(5)
C(7)-C(8)	1.517(5)
C(7)-C(10)	1.527(4)
C(7)-C(9)	1.544(5)
C(11)-C(12)	1.346(4)
C(13)-C(14)	1.526(4)
C(13)-C(16)	1.531(4)
C(13)-C(15)	1.535(4)
C(17)-C(18)	1.530(4)
C(17)-C(19)	1.531(4)
C(17)-C(20)	1.535(4)
C(21)-C(22)	1.347(4)
C(23)-C(26)	1.528(4)
C(23)-C(25)	1.532(5)
C(23)-C(24)	1.536(5)
C(27)-C(29)	1.523(5)
C(27)-C(28)	1.528(5)
C(27)-C(30)	1.533(5)
C(31)-C(32)	1.543(4)
C(33)-C(35)	1.534(4)
C(33)-C(34)	1.534(4)

O(3)-Ni(4)-C(82)	33.14(8)
O(4)-Ni(4)-C(82)	32.95(8)
P(7)-Ni(4)-C(82)	139.10(7)
C(2)-P(1)-C(7)	104.72(14)
C(2)-P(1)-C(3)	105.51(14)
C(7)-P(1)-C(3)	112.91(14)
C(2)-P(1)-Ni(1)	99.55(11)
C(7)-P(1)-Ni(1)	116.87(10)
C(3)-P(1)-Ni(1)	114.99(9)
C(12)-P(2)-C(17)	104.98(12)
C(12)-P(2)-C(13)	105.09(13)
C(17)-P(2)-C(13)	113.32(13)
C(12)-P(2)-Ni(1)	100.19(10)
C(17)-P(2)-Ni(1)	119.40(9)
C(13)-P(2)-Ni(1)	111.62(9)
C(22)-P(3)-C(27)	106.17(16)
C(22)-P(3)-C(23)	102.86(17)
C(27)-P(3)-C(23)	111.72(14)
C(22)-P(3)-Ni(2)	114.57(11)
C(27)-P(3)-Ni(2)	109.33(12)
C(23)-P(3)-Ni(2)	111.97(10)
C(32)-P(4)-C(37)	103.30(12)
C(32)-P(4)-C(33)	100.50(12)
C(37)-P(4)-C(33)	110.33(13)
C(43)-P(5)-C(48)	104.85(13)
C(43)-P(5)-C(44)	104.25(14)
C(48)-P(5)-C(44)	113.34(13)
C(43)-P(5)-Ni(3)	99.68(10)
C(48)-P(5)-Ni(3)	116.30(8)
C(44)-P(5)-Ni(3)	115.84(9)
C(53)-P(6)-C(54)	105.42(13)
C(53)-P(6)-C(58)	103.61(12)
C(54)-P(6)-C(58)	113.49(12)
C(53)-P(6)-Ni(3)	99.23(9)
C(54)-P(6)-Ni(3)	115.34(9)
C(58)-P(6)-Ni(3)	117.02(9)
C(63)-P(7)-C(64)	103.65(14)
C(63)-P(7)-C(68)	105.58(14)
C(64)-P(7)-C(68)	111.43(13)
C(63)-P(7)-Ni(4)	114.96(10)
C(64)-P(7)-Ni(4)	111.15(10)
C(68)-P(7)-Ni(4)	109.85(10)
C(73)-P(8)-C(74)	100.07(12)
C(73)-P(8)-C(78)	103.79(12)
C(74)-P(8)-C(78)	109.99(12)
C(41)-O(1)-Ni(2)	90.87(15)
C(41)-O(2)-Ni(2)	88.75(15)
C(82)-O(3)-Ni(4)	90.77(15)
C(82)-O(4)-Ni(4)	88.94(14)
C(11)-N(1)-C(1)	119.7(2)
C(11)-N(1)-Ni(1)	120.32(18)

C(33)-C(36)	1.535(5)
C(37)-C(39)	1.524(4)
C(37)-C(38)	1.538(4)
C(37)-C(40)	1.539(4)
C(42)-C(43)	1.336(4)
C(44)-C(46)	1.529(4)
C(44)-C(47)	1.530(4)
C(44)-C(45)	1.531(4)
C(48)-C(50)	1.522(4)
C(48)-C(51)	1.534(4)
C(48)-C(49)	1.537(4)
C(52)-C(53)	1.339(4)
C(54)-C(57)	1.522(4)
C(54)-C(55)	1.531(4)
C(54)-C(56)	1.537(4)
C(58)-C(60)	1.526(4)
C(58)-C(61)	1.532(4)
C(58)-C(59)	1.537(4)
C(62)-C(63)	1.345(4)
C(64)-C(67)	1.530(4)
C(64)-C(66)	1.536(4)
C(64)-C(65)	1.537(4)
C(68)-C(69)	1.522(4)
C(68)-C(71)	1.531(4)
C(68)-C(70)	1.537(4)
C(72)-C(73)	1.531(4)
C(74)-C(76)	1.524(4)
C(74)-C(77)	1.538(4)
C(74)-C(75)	1.542(4)
C(78)-C(80)	1.526(4)
C(78)-C(81)	1.534(4)
C(78)-C(79)	1.541(4)
C(83)-C(84)	1.519(5)
C(84)-C(85)	1.506(4)
C(85)-C(86)	1.511(4)
C(86)-C(87)	1.521(5)
C(88)-C(89)	1.444(10)
C(89)-C(90)	1.473(10)
C(90)-C(91)	1.414(10)
C(91)-C(92)	1.414(9)
C(88A)-C(89A)	1.407(13)
C(89A)-C(90A)	1.467(11)
C(90A)-C(91A)	1.307(11)
C(91A)-C(92)	1.375(11)

C(1)-N(1)-Ni(1)	119.9(2)
C(21)-N(2)-C(31)	126.1(2)
C(21)-N(2)-H(2A)	116(2)
C(31)-N(2)-H(2A)	118(2)
C(52)-N(3)-C(42)	119.8(2)
C(52)-N(3)-Ni(3)	120.18(18)
C(42)-N(3)-Ni(3)	119.99(19)
C(62)-N(4)-C(72)	128.5(2)
C(62)-N(4)-H(4D)	115(2)
C(72)-N(4)-H(4D)	117(2)
C(2)-C(1)-N(1)	121.2(3)
C(1)-C(2)-P(1)	113.8(2)
C(5)-C(3)-C(4)	109.2(3)
C(5)-C(3)-C(6)	109.0(3)
C(4)-C(3)-C(6)	108.3(3)
C(5)-C(3)-P(1)	113.6(2)
C(4)-C(3)-P(1)	105.2(2)
C(6)-C(3)-P(1)	111.2(2)
C(8)-C(7)-C(10)	109.6(3)
C(8)-C(7)-C(9)	109.2(3)
C(10)-C(7)-C(9)	109.1(3)
C(8)-C(7)-P(1)	112.7(2)
C(10)-C(7)-P(1)	112.4(2)
C(9)-C(7)-P(1)	103.5(2)
C(12)-C(11)-N(1)	121.1(2)
C(11)-C(12)-P(2)	113.3(2)
C(14)-C(13)-C(16)	108.2(3)
C(14)-C(13)-C(15)	110.2(3)
C(16)-C(13)-C(15)	108.7(2)
C(14)-C(13)-P(2)	111.85(19)
C(16)-C(13)-P(2)	104.44(19)
C(15)-C(13)-P(2)	113.17(19)
C(18)-C(17)-C(19)	109.3(2)
C(18)-C(17)-C(20)	109.0(2)
C(19)-C(17)-C(20)	108.6(2)
C(18)-C(17)-P(2)	112.10(18)
C(19)-C(17)-P(2)	112.94(18)
C(20)-C(17)-P(2)	104.72(18)
N(2)-C(21)-C(22)	128.1(3)
C(21)-C(22)-P(3)	122.3(2)
C(26)-C(23)-C(25)	109.8(3)
C(26)-C(23)-C(24)	108.3(3)
C(25)-C(23)-C(24)	108.3(3)
C(26)-C(23)-P(3)	111.3(2)
C(25)-C(23)-P(3)	114.3(3)
C(24)-C(23)-P(3)	104.6(2)
C(29)-C(27)-C(28)	109.3(3)
C(29)-C(27)-C(30)	109.6(3)
C(28)-C(27)-C(30)	107.2(3)
C(29)-C(27)-P(3)	113.7(3)
C(28)-C(27)-P(3)	111.5(2)

C(30)-C(27)-P(3)	105.2(2)
N(2)-C(31)-C(32)	109.5(2)
N(2)-C(31)-Ni(2)	124.68(18)
C(32)-C(31)-Ni(2)	106.50(16)
C(31)-C(32)-P(4)	114.08(17)
C(35)-C(33)-C(34)	108.7(3)
C(35)-C(33)-C(36)	108.4(3)
C(34)-C(33)-C(36)	108.9(3)
C(35)-C(33)-P(4)	109.5(2)
C(34)-C(33)-P(4)	117.1(2)
C(36)-C(33)-P(4)	103.9(2)
C(39)-C(37)-C(38)	109.3(2)
C(39)-C(37)-C(40)	109.1(2)
C(38)-C(37)-C(40)	107.6(2)
C(39)-C(37)-P(4)	116.35(19)
C(38)-C(37)-P(4)	104.96(18)
C(40)-C(37)-P(4)	109.25(19)
O(2)-C(41)-O(1)	114.0(2)
O(2)-C(41)-Ni(1)	129.29(19)
O(1)-C(41)-Ni(1)	116.70(18)
O(2)-C(41)-Ni(2)	58.34(13)
O(1)-C(41)-Ni(2)	55.86(12)
Ni(1)-C(41)-Ni(2)	170.77(13)
C(43)-C(42)-N(3)	121.3(3)
C(42)-C(43)-P(5)	113.8(2)
C(46)-C(44)-C(47)	109.2(3)
C(46)-C(44)-C(45)	109.4(3)
C(47)-C(44)-C(45)	108.2(3)
C(46)-C(44)-P(5)	113.2(2)
C(47)-C(44)-P(5)	111.71(19)
C(45)-C(44)-P(5)	105.0(2)
C(50)-C(48)-C(51)	109.6(2)
C(50)-C(48)-C(49)	109.5(2)
C(51)-C(48)-C(49)	108.3(2)
C(50)-C(48)-P(5)	112.69(19)
C(51)-C(48)-P(5)	103.70(18)
C(49)-C(48)-P(5)	112.83(18)
C(53)-C(52)-N(3)	121.4(3)
C(52)-C(53)-P(6)	114.0(2)
C(57)-C(54)-C(55)	109.9(2)
C(57)-C(54)-C(56)	109.1(2)
C(55)-C(54)-C(56)	108.8(2)
C(57)-C(54)-P(6)	112.03(19)
C(55)-C(54)-P(6)	112.5(2)
C(56)-C(54)-P(6)	104.28(18)
C(60)-C(58)-C(61)	108.8(2)
C(60)-C(58)-C(59)	108.0(2)
C(61)-C(58)-C(59)	109.4(2)
C(60)-C(58)-P(6)	113.95(19)
C(61)-C(58)-P(6)	112.42(19)
C(59)-C(58)-P(6)	103.95(18)

N(4)-C(62)-C(63)	129.2(3)
C(62)-C(63)-P(7)	122.4(2)
C(67)-C(64)-C(66)	109.4(3)
C(67)-C(64)-C(65)	109.9(2)
C(66)-C(64)-C(65)	107.2(2)
C(67)-C(64)-P(7)	110.9(2)
C(66)-C(64)-P(7)	104.32(19)
C(65)-C(64)-P(7)	114.7(2)
C(69)-C(68)-C(71)	109.8(2)
C(69)-C(68)-C(70)	108.4(3)
C(71)-C(68)-C(70)	108.8(2)
C(69)-C(68)-P(7)	110.2(2)
C(71)-C(68)-P(7)	113.9(2)
C(70)-C(68)-P(7)	105.57(19)
N(4)-C(72)-C(73)	109.2(2)
N(4)-C(72)-Ni(4)	125.61(18)
C(73)-C(72)-Ni(4)	106.91(17)
C(72)-C(73)-P(8)	114.13(17)
C(76)-C(74)-C(77)	108.5(2)
C(76)-C(74)-C(75)	108.2(2)
C(77)-C(74)-C(75)	109.0(2)
C(76)-C(74)-P(8)	110.71(18)
C(77)-C(74)-P(8)	104.03(18)
C(75)-C(74)-P(8)	116.11(18)
C(80)-C(78)-C(81)	109.5(2)
C(80)-C(78)-C(79)	109.0(2)
C(81)-C(78)-C(79)	108.2(2)
C(80)-C(78)-P(8)	116.17(18)
C(81)-C(78)-P(8)	108.68(18)
C(79)-C(78)-P(8)	104.97(18)
O(4)-C(82)-O(3)	114.1(2)
O(4)-C(82)-Ni(3)	126.55(19)
O(3)-C(82)-Ni(3)	119.31(19)
O(4)-C(82)-Ni(4)	58.11(12)
O(3)-C(82)-Ni(4)	56.10(12)
Ni(3)-C(82)-Ni(4)	173.86(13)
C(85)-C(84)-C(83)	113.6(3)
C(84)-C(85)-C(86)	114.7(3)
C(85)-C(86)-C(87)	113.6(3)
C(88)-C(89)-C(90)	116.3(9)
C(91)-C(90)-C(89)	122.3(9)
C(90)-C(91)-C(92)	132.9(10)
C(88A)-C(89A)-C(90A)	123.2(13)
C(91A)-C(90A)-C(89A)	121.8(13)
C(90A)-C(91A)-C(92)	134.0(14)

Supplementary Table 44. Torsion angles [°] for **10**.

C(11)-N(1)-C(1)-C(2)	-179.5(3)
Ni(1)-N(1)-C(1)-C(2)	-3.2(4)
N(1)-C(1)-C(2)-P(1)	-0.3(4)
C(7)-P(1)-C(2)-C(1)	124.0(2)
C(3)-P(1)-C(2)-C(1)	-116.7(2)
Ni(1)-P(1)-C(2)-C(1)	2.8(2)
C(2)-P(1)-C(3)-C(5)	-65.5(3)
C(7)-P(1)-C(3)-C(5)	48.3(3)
Ni(1)-P(1)-C(3)-C(5)	-174.1(3)
C(2)-P(1)-C(3)-C(4)	54.0(2)
C(7)-P(1)-C(3)-C(4)	167.8(2)
Ni(1)-P(1)-C(3)-C(4)	-54.6(2)
C(2)-P(1)-C(3)-C(6)	171.1(2)
C(7)-P(1)-C(3)-C(6)	-75.1(3)
Ni(1)-P(1)-C(3)-C(6)	62.5(2)
C(2)-P(1)-C(7)-C(8)	172.1(2)
C(3)-P(1)-C(7)-C(8)	57.9(2)
Ni(1)-P(1)-C(7)-C(8)	-78.9(2)
C(2)-P(1)-C(7)-C(10)	47.6(3)
C(3)-P(1)-C(7)-C(10)	-66.7(3)
Ni(1)-P(1)-C(7)-C(10)	156.6(2)
C(2)-P(1)-C(7)-C(9)	-70.0(2)
C(3)-P(1)-C(7)-C(9)	175.7(2)
Ni(1)-P(1)-C(7)-C(9)	38.9(2)
C(1)-N(1)-C(11)-C(12)	-179.9(3)
Ni(1)-N(1)-C(11)-C(12)	3.8(4)
N(1)-C(11)-C(12)-P(2)	1.2(3)
C(17)-P(2)-C(12)-C(11)	-128.9(2)
C(13)-P(2)-C(12)-C(11)	111.4(2)
Ni(1)-P(2)-C(12)-C(11)	-4.5(2)
C(12)-P(2)-C(13)-C(14)	-174.5(2)
C(17)-P(2)-C(13)-C(14)	71.4(2)
Ni(1)-P(2)-C(13)-C(14)	-66.9(2)
C(12)-P(2)-C(13)-C(16)	-57.8(2)
C(17)-P(2)-C(13)-C(16)	-171.85(18)
Ni(1)-P(2)-C(13)-C(16)	49.9(2)
C(12)-P(2)-C(13)-C(15)	60.3(2)
C(17)-P(2)-C(13)-C(15)	-53.8(2)
Ni(1)-P(2)-C(13)-C(15)	167.98(18)
C(12)-P(2)-C(17)-C(18)	-165.9(2)
C(13)-P(2)-C(17)-C(18)	-51.7(2)
Ni(1)-P(2)-C(17)-C(18)	83.0(2)
C(12)-P(2)-C(17)-C(19)	-41.8(2)
C(13)-P(2)-C(17)-C(19)	72.3(2)
Ni(1)-P(2)-C(17)-C(19)	-152.98(17)
C(12)-P(2)-C(17)-C(20)	76.2(2)
C(13)-P(2)-C(17)-C(20)	-169.72(17)
Ni(1)-P(2)-C(17)-C(20)	-35.0(2)
C(31)-N(2)-C(21)-C(22)	17.2(5)
N(2)-C(21)-C(22)-P(3)	9.6(6)

C(27)-P(3)-C(22)-C(21)	-132.5(3)
C(23)-P(3)-C(22)-C(21)	110.0(3)
Ni(2)-P(3)-C(22)-C(21)	-11.8(4)
C(22)-P(3)-C(23)-C(26)	166.0(2)
C(27)-P(3)-C(23)-C(26)	52.5(3)
Ni(2)-P(3)-C(23)-C(26)	-70.5(2)
C(22)-P(3)-C(23)-C(25)	41.0(3)
C(27)-P(3)-C(23)-C(25)	-72.5(3)
Ni(2)-P(3)-C(23)-C(25)	164.5(2)
C(22)-P(3)-C(23)-C(24)	-77.3(3)
C(27)-P(3)-C(23)-C(24)	169.2(2)
Ni(2)-P(3)-C(23)-C(24)	46.2(2)
C(22)-P(3)-C(27)-C(29)	-58.1(3)
C(23)-P(3)-C(27)-C(29)	53.3(3)
Ni(2)-P(3)-C(27)-C(29)	177.8(3)
C(22)-P(3)-C(27)-C(28)	177.7(2)
C(23)-P(3)-C(27)-C(28)	-70.9(3)
Ni(2)-P(3)-C(27)-C(28)	53.6(3)
C(22)-P(3)-C(27)-C(30)	61.8(3)
C(23)-P(3)-C(27)-C(30)	173.2(3)
Ni(2)-P(3)-C(27)-C(30)	-62.3(3)
C(21)-N(2)-C(31)-C(32)	87.6(3)
C(21)-N(2)-C(31)-Ni(2)	-40.1(4)
N(2)-C(31)-C(32)-P(4)	60.4(2)
Ni(2)-C(31)-C(32)-P(4)	-162.37(13)
C(37)-P(4)-C(32)-C(31)	103.4(2)
C(33)-P(4)-C(32)-C(31)	-142.5(2)
C(32)-P(4)-C(33)-C(35)	-164.8(2)
C(37)-P(4)-C(33)-C(35)	-56.2(3)
C(32)-P(4)-C(33)-C(34)	-40.5(3)
C(37)-P(4)-C(33)-C(34)	68.1(3)
C(32)-P(4)-C(33)-C(36)	79.6(2)
C(37)-P(4)-C(33)-C(36)	-171.83(18)
C(32)-P(4)-C(37)-C(39)	66.3(2)
C(33)-P(4)-C(37)-C(39)	-40.4(2)
C(32)-P(4)-C(37)-C(38)	-54.6(2)
C(33)-P(4)-C(37)-C(38)	-161.32(18)
C(32)-P(4)-C(37)-C(40)	-169.73(19)
C(33)-P(4)-C(37)-C(40)	83.6(2)
Ni(2)-O(2)-C(41)-O(1)	-5.0(2)
Ni(2)-O(2)-C(41)-Ni(1)	173.60(19)
Ni(2)-O(1)-C(41)-O(2)	5.2(2)
Ni(2)-O(1)-C(41)-Ni(1)	-173.64(13)
P(1)-Ni(1)-C(41)-O(2)	87.0(2)
P(2)-Ni(1)-C(41)-O(2)	-88.9(2)
P(1)-Ni(1)-C(41)-O(1)	-94.35(18)
P(2)-Ni(1)-C(41)-O(1)	89.75(18)
C(52)-N(3)-C(42)-C(43)	178.0(3)
Ni(3)-N(3)-C(42)-C(43)	-1.8(4)
N(3)-C(42)-C(43)-P(5)	-2.9(4)
C(48)-P(5)-C(43)-C(42)	125.8(2)

C(44)-P(5)-C(43)-C(42)	-114.9(2)
Ni(3)-P(5)-C(43)-C(42)	5.1(2)
C(43)-P(5)-C(44)-C(46)	-61.6(3)
C(48)-P(5)-C(44)-C(46)	51.9(3)
Ni(3)-P(5)-C(44)-C(46)	-170.0(2)
C(43)-P(5)-C(44)-C(47)	174.7(2)
C(48)-P(5)-C(44)-C(47)	-71.8(2)
Ni(3)-P(5)-C(44)-C(47)	66.3(2)
C(43)-P(5)-C(44)-C(45)	57.7(2)
C(48)-P(5)-C(44)-C(45)	171.1(2)
Ni(3)-P(5)-C(44)-C(45)	-50.7(2)
C(43)-P(5)-C(48)-C(50)	168.45(19)
C(44)-P(5)-C(48)-C(50)	55.4(2)
Ni(3)-P(5)-C(48)-C(50)	-82.58(19)
C(43)-P(5)-C(48)-C(51)	-73.1(2)
C(44)-P(5)-C(48)-C(51)	173.79(18)
Ni(3)-P(5)-C(48)-C(51)	35.8(2)
C(43)-P(5)-C(48)-C(49)	43.8(2)
C(44)-P(5)-C(48)-C(49)	-69.2(2)
Ni(3)-P(5)-C(48)-C(49)	152.80(18)
C(42)-N(3)-C(52)-C(53)	-179.2(3)
Ni(3)-N(3)-C(52)-C(53)	0.6(4)
N(3)-C(52)-C(53)-P(6)	-3.6(4)
C(54)-P(6)-C(53)-C(52)	123.9(2)
C(58)-P(6)-C(53)-C(52)	-116.6(2)
Ni(3)-P(6)-C(53)-C(52)	4.3(2)
C(53)-P(6)-C(54)-C(57)	161.5(2)
C(58)-P(6)-C(54)-C(57)	48.8(2)
Ni(3)-P(6)-C(54)-C(57)	-90.1(2)
C(53)-P(6)-C(54)-C(55)	37.1(2)
C(58)-P(6)-C(54)-C(55)	-75.6(2)
Ni(3)-P(6)-C(54)-C(55)	145.48(17)
C(53)-P(6)-C(54)-C(56)	-80.7(2)
C(58)-P(6)-C(54)-C(56)	166.62(18)
Ni(3)-P(6)-C(54)-C(56)	27.7(2)
C(53)-P(6)-C(58)-C(60)	-179.0(2)
C(54)-P(6)-C(58)-C(60)	-65.2(2)
Ni(3)-P(6)-C(58)-C(60)	73.1(2)
C(53)-P(6)-C(58)-C(61)	-54.5(2)
C(54)-P(6)-C(58)-C(61)	59.3(2)
Ni(3)-P(6)-C(58)-C(61)	-162.50(17)
C(53)-P(6)-C(58)-C(59)	63.7(2)
C(54)-P(6)-C(58)-C(59)	177.5(2)
Ni(3)-P(6)-C(58)-C(59)	-44.3(2)
C(72)-N(4)-C(62)-C(63)	8.4(5)
N(4)-C(62)-C(63)-P(7)	5.2(5)
C(64)-P(7)-C(63)-C(62)	119.3(3)
C(68)-P(7)-C(63)-C(62)	-123.5(3)
Ni(4)-P(7)-C(63)-C(62)	-2.2(3)
C(63)-P(7)-C(64)-C(67)	169.8(2)
C(68)-P(7)-C(64)-C(67)	56.7(2)

Ni(4)-P(7)-C(64)-C(67)	-66.1(2)
C(63)-P(7)-C(64)-C(66)	-72.4(2)
C(68)-P(7)-C(64)-C(66)	174.45(19)
Ni(4)-P(7)-C(64)-C(66)	51.6(2)
C(63)-P(7)-C(64)-C(65)	44.6(2)
C(68)-P(7)-C(64)-C(65)	-68.5(2)
Ni(4)-P(7)-C(64)-C(65)	168.58(19)
C(63)-P(7)-C(68)-C(69)	165.3(2)
C(64)-P(7)-C(68)-C(69)	-82.8(2)
Ni(4)-P(7)-C(68)-C(69)	40.8(2)
C(63)-P(7)-C(68)-C(71)	-70.8(2)
C(64)-P(7)-C(68)-C(71)	41.1(3)
Ni(4)-P(7)-C(68)-C(71)	164.73(19)
C(63)-P(7)-C(68)-C(70)	48.5(2)
C(64)-P(7)-C(68)-C(70)	160.4(2)
Ni(4)-P(7)-C(68)-C(70)	-76.0(2)
C(62)-N(4)-C(72)-C(73)	104.3(3)
C(62)-N(4)-C(72)-Ni(4)	-24.7(4)
N(4)-C(72)-C(73)-P(8)	60.2(3)
Ni(4)-C(72)-C(73)-P(8)	-161.14(13)
C(74)-P(8)-C(73)-C(72)	-142.30(19)
C(78)-P(8)-C(73)-C(72)	104.1(2)
C(73)-P(8)-C(74)-C(76)	-162.98(19)
C(78)-P(8)-C(74)-C(76)	-54.2(2)
C(73)-P(8)-C(74)-C(77)	80.64(19)
C(78)-P(8)-C(74)-C(77)	-170.56(17)
C(73)-P(8)-C(74)-C(75)	-39.1(2)
C(78)-P(8)-C(74)-C(75)	69.7(2)
C(73)-P(8)-C(78)-C(80)	64.1(2)
C(74)-P(8)-C(78)-C(80)	-42.3(2)
C(73)-P(8)-C(78)-C(81)	-172.00(18)
C(74)-P(8)-C(78)-C(81)	81.7(2)
C(73)-P(8)-C(78)-C(79)	-56.38(19)
C(74)-P(8)-C(78)-C(79)	-162.70(16)
Ni(4)-O(4)-C(82)-O(3)	-3.3(2)
Ni(4)-O(4)-C(82)-Ni(3)	175.18(17)
Ni(4)-O(3)-C(82)-O(4)	3.4(2)
Ni(4)-O(3)-C(82)-Ni(3)	-175.22(14)
P(5)-Ni(3)-C(82)-O(4)	89.5(2)
P(6)-Ni(3)-C(82)-O(4)	-88.8(2)
P(5)-Ni(3)-C(82)-O(3)	-92.09(19)
P(6)-Ni(3)-C(82)-O(3)	89.64(19)
C(83)-C(84)-C(85)-C(86)	-176.9(3)
C(84)-C(85)-C(86)-C(87)	176.7(3)
C(88)-C(89)-C(90)-C(91)	56.3(15)
C(89)-C(90)-C(91)-C(92)	105.5(15)
C(88A)-C(89A)-C(90A)-C(91A)	-135.4(19)
C(89A)-C(90A)-C(91A)-C(92)	-30(3)

X-ray Single-Crystal Structure Analysis of **11**:

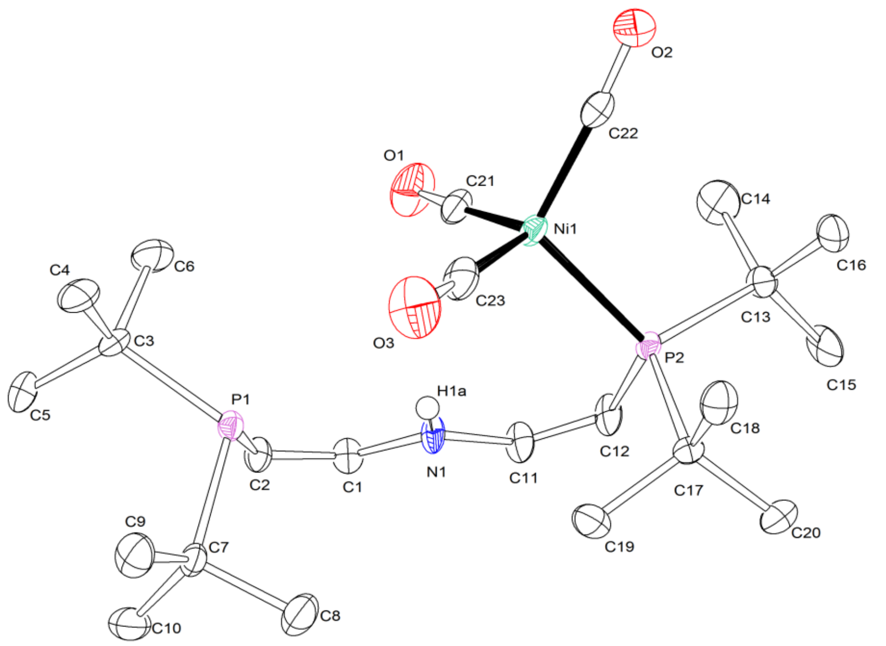

Supplementary Figure 56**.** Thermal ellipsoid plot of **11** with the anisotropic displacement parameters at the 50% probability level. All C-H hydrogen atoms are omitted for clarity. The N-H hydrogen atom was found from residual density map and isotropically refined. The asymmetric unit contains one complex molecule.

Supplementary Table 45. Crystal data and structure refinement for **11**.

Identification code	SF_SF_300817	
Empirical formula	$C_{23}H_{41}NNiO_3P_2$	
Formula weight	500.22	
Temperature	100(2) K	
Wavelength	0.71073 Å	
Crystal system	Monoclinic	
Space group	$P2_1/c$	
Unit cell dimensions	a = 8.7840(11) Å	α = 90°
	b = 16.0680(19) Å	β = 91.080(4)°
	c = 19.419(2) Å	γ = 90°
Volume	2740.3(6) Å^3	
Z	4	
Density (calculated)	1.212 Mg/m^3	
Absorption coefficient	0.846 mm^{-1}	
F(000)	1072	

Crystal size	0.302 x 0.121 x 0.067 mm^3
Crystal shape and color	Block, clear light yellow
Theta range for data collection	2.319 to 26.442°
Index ranges	-10<=h<=10, -20<=k<=20, -23<=l<=24
Reflections collected	62264
Independent reflections	5624 [R(int) = 0.1224]
Completeness to theta = 25.242°	100.0 %
Refinement method	Full-matrix least-squares on F2
Data / restraints / parameters	5624 / 0 / 287
Goodness-of-fit on F^2	1.128
Final R indices [I>2sigma(I)]	R1 = 0.0499, wR2 = 0.0925
R indices (all data)	R1 = 0.0712, wR2 = 0.0992
Largest diff. peak and hole	0.481 and -0.524 eÅ^{-3}

Supplementary Table 46. Bond lengths [Å] and angles [°] for **11**.

Ni(1)-C(23)	1.792(3)	C(23)-Ni(1)-C(21)	114.16(15)
Ni(1)-C(21)	1.793(3)	C(23)-Ni(1)-C(22)	108.30(15)
Ni(1)-C(22)	1.794(3)	C(21)-Ni(1)-C(22)	108.00(15)
Ni(1)-P(2)	2.2702(8)	C(23)-Ni(1)-P(2)	110.85(11)
O(1)-C(21)	1.139(4)	C(21)-Ni(1)-P(2)	105.28(11)
P(1)-C(2)	1.807(3)	C(22)-Ni(1)-P(2)	110.17(10)
P(1)-C(3)	1.877(3)	C(2)-P(1)-C(3)	102.20(14)
P(1)-C(7)	1.887(3)	C(2)-P(1)-C(7)	100.44(14)
N(1)-C(11)	1.368(4)	C(3)-P(1)-C(7)	110.70(13)
N(1)-C(1)	1.381(4)	C(11)-N(1)-C(1)	122.5(3)
N(1)-H(1A)	0.99(4)	C(11)-N(1)-H(1A)	121(2)
C(1)-C(2)	1.337(4)	C(1)-N(1)-H(1A)	116(2)
P(2)-C(12)	1.798(3)	C(2)-C(1)-N(1)	125.8(3)
P(2)-C(17)	1.892(3)	C(12)-P(2)-C(17)	103.55(14)
P(2)-C(13)	1.900(3)	C(12)-P(2)-C(13)	100.02(14)
O(2)-C(22)	1.141(4)	C(17)-P(2)-C(13)	111.19(13)
C(3)-C(5)	1.528(4)	C(12)-P(2)-Ni(1)	115.86(10)
C(3)-C(4)	1.532(4)	C(17)-P(2)-Ni(1)	113.41(10)
C(3)-C(6)	1.540(4)	C(13)-P(2)-Ni(1)	111.82(10)
O(3)-C(23)	1.140(4)	C(1)-C(2)-P(1)	122.1(2)
C(7)-C(9)	1.523(4)	C(5)-C(3)-C(4)	109.0(3)
C(7)-C(10)	1.528(4)	C(5)-C(3)-C(6)	109.3(3)
C(7)-C(8)	1.535(4)	C(4)-C(3)-C(6)	108.1(3)
C(11)-C(12)	1.341(4)	C(5)-C(3)-P(1)	116.4(2)
C(13)-C(16)	1.525(4)	C(4)-C(3)-P(1)	109.2(2)
C(13)-C(14)	1.536(4)	C(6)-C(3)-P(1)	104.6(2)
C(13)-C(15)	1.537(4)	C(9)-C(7)-C(10)	109.6(3)
C(17)-C(18)	1.526(4)	C(9)-C(7)-C(8)	108.3(3)
C(17)-C(20)	1.530(4)	C(10)-C(7)-C(8)	108.1(3)
C(17)-C(19)	1.536(4)	C(9)-C(7)-P(1)	110.0(2)
		C(10)-C(7)-P(1)	116.8(2)

C(8)-C(7)-P(1)	103.7(2)
C(12)-C(11)-N(1)	128.6(3)
C(11)-C(12)-P(2)	129.2(2)
C(16)-C(13)-C(14)	109.6(3)
C(16)-C(13)-C(15)	108.0(3)
C(14)-C(13)-C(15)	106.4(3)
C(16)-C(13)-P(2)	110.6(2)
C(14)-C(13)-P(2)	105.5(2)
C(15)-C(13)-P(2)	116.5(2)
C(18)-C(17)-C(20)	109.6(3)
C(18)-C(17)-C(19)	108.2(3)
C(20)-C(17)-C(19)	108.5(2)
C(18)-C(17)-P(2)	110.5(2)
C(20)-C(17)-P(2)	113.7(2)
C(19)-C(17)-P(2)	106.2(2)
O(1)-C(21)-Ni(1)	177.7(3)
O(2)-C(22)-Ni(1)	176.5(3)
O(3)-C(23)-Ni(1)	176.3(3)

Supplementary Table 47. Torsion angles [°] for **11**.

C(11)-N(1)-C(1)-C(2)	176.3(3)
N(1)-C(1)-C(2)-P(1)	-2.3(5)
C(3)-P(1)-C(2)-C(1)	139.1(3)
C(7)-P(1)-C(2)-C(1)	-106.9(3)
C(2)-P(1)-C(3)-C(5)	61.3(3)
C(7)-P(1)-C(3)-C(5)	-44.9(3)
C(2)-P(1)-C(3)-C(4)	-174.8(2)
C(7)-P(1)-C(3)-C(4)	78.9(2)
C(2)-P(1)-C(3)-C(6)	-59.3(2)
C(7)-P(1)-C(3)-C(6)	-165.6(2)
C(2)-P(1)-C(7)-C(9)	-165.9(2)
C(3)-P(1)-C(7)-C(9)	-58.5(2)
C(2)-P(1)-C(7)-C(10)	-40.1(2)
C(3)-P(1)-C(7)-C(10)	67.3(3)
C(2)-P(1)-C(7)-C(8)	78.6(2)
C(3)-P(1)-C(7)-C(8)	-174.0(2)
C(1)-N(1)-C(11)-C(12)	172.7(3)
N(1)-C(11)-C(12)-P(2)	-4.9(6)
C(17)-P(2)-C(12)-C(11)	93.9(3)
C(13)-P(2)-C(12)-C(11)	-151.2(3)
Ni(1)-P(2)-C(12)-C(11)	-30.9(4)
C(12)-P(2)-C(17)-C(18)	-173.4(2)
C(13)-P(2)-C(17)-C(18)	80.0(2)
Ni(1)-P(2)-C(17)-C(18)	-47.0(2)
C(12)-P(2)-C(17)-C(20)	62.9(2)

C(13)-P(2)-C(17)-C(20)	-43.7(3)
Ni(1)-P(2)-C(17)-C(20)	-170.73(19)
C(12)-P(2)-C(17)-C(19)	-56.3(2)
C(13)-P(2)-C(17)-C(19)	-162.9(2)
Ni(1)-P(2)-C(17)-C(19)	70.1(2)

Supplementary References

1. Schneck, F., Finger, M. Tromp, M. & Schneider, S. Chemical non-innocence of an aliphatic PNP pincer ligand. *Chem. Eur. J.* **23**, 33–37 (2017).

2. Ozinskas, A. J.& Bobst, A. M. Formation of *N*-hydroxy-amines of spin labeled nucleosides for ^{1}H-NMR analysis. *Helv. Chim. Acta* **63**, 1407–1411 (1980).

3. Hoops, S. *et al.* COPASI─a COmplex PAthway SImulator. *Bioinformatics* **22**, 3067–3074 (2006).

4. Kaufman, M. J., Gronert, S. & Streitwieser, A. Carbon Acidity. 73. Conductimetric Study of Lithium and Cesium Salts of Hydrocarbon Acids. A Scale of Free Ion Acidities in Tetrahydrofuran. Revision of the Ion Pair Scales. *J. Am. Chem. Soc.* **110**, 2829–2835 (1988).

5. Fuoss, R. M. Ionic Association. III. The Equilibrium between Ion Pairs and Free Ions. *J. Am. Chem. Soc.* **80**, 5059–5061 (1958).

6. Metz, D. J. & Glines, A. Density, Viscosity, and Dielectric Constant of Tetrahydrofuran between -78 and 30°. *J. Phys. Chem.* **71**, 1158 (1967).

7. Leito, I. *et al.* Acid-Base Equilibria in Nonpolar Media. 4. Extension of the Self-Consistent Basicity Scale in THF Medium. Gas-Phase Basicities of Phosphazenes. *J. Org. Chem.* **68**, 9988–9993 (2003).

8. Morris, R. H. *et al.* An Acidity Scale for Phosphorus-Containing Compounds Including Metal Hydrides and Dihydrogen Complexes in THF: Toward the Unification of Acidity Scales. *J. Am. Chem. Soc.* **122**, 9155–9171 (2000).

9. Smith, G. & Lynch, D. E. Crystal structure of three anhydrous salts of the Lewis base 1,8-diazabicyclo[5.4.0]undec-7-ene (DBU) with the ring-substituted benzoic acid analogue 4-aminobenzoic acid, 3,5-dinitrobenzoic acid and 3,5-dinitrosalicyclic acid. *Acta Cryst.* **E72**, 382–386 (2016).

10. Reed, C. A. *et al.* Molecular Structure of the Solvated Proton in Isolated Salts. Short, Strong, Low Barrier (SSLB) H-bonds. *J. Am. Chem. Soc.* **124**, 13869–13876 (2002).

11. Woerner, M. *et al.* Generation, shaping and characterization of intense femtosecond pulses tunable from 3 to 20 µm. *J. Opt. Soc. Am. B* **17**, 2086–2094 (2000).

12. Velapoldi, R. A., Zalewski, E. F., Bowman, W. D. & Demas, J. D. Determination of the quantum yield of the ferrioxalate actinometer with electrically calibrated radiometers. *J. Phys. Chem.* **85**, 2766–2771 (1981).

13. F. Neese, The ORCA program system. *Wiley Interdiscip. Rev. Comput. Mol. Sci.* **2**, 73–78 (2012).

14. F. Neese, Software update: the ORCA program system, version 4.0. *Wiley Interdiscip. Rev. Comput. Mol. Sci.* e1327 (2017).

15. Perdew, J. P., Burke, K. & Ernzerhof, M. Generalized gradient approximation made simple. *Phys. Rev. Lett*. **77**, 3865–3868 (1996).

16. Grimme, S., Antony, J., Ehrlich, S. & Krieg, H. A consistent and accurate ab initio parametrization of density functional dispersion correction (DFT-D) for the 94 elements H-Pu. *J. Chem. Phys.* **132**, 154104 (2010).

17. Grimme, S., Ehrlich, S., Goerigk, L. Effect of the damping function in dispersion corrected density functional theory. *J. Comput. Chem.* **32**, 1456–1465 (2011).

18. Treutler, O. & Ahlrichs, R. Efficient molecular numerical integration schemes. *J. Chem. Phys.* **102**, 346 (1995).

19. Ahlrichs, R. *et al.* Auxiliary basis sets to approximate coulomb potentials. *Chem. Phys. Lett.* **40**, 283–290 (1995).

20. Ahlrichs, R. *et al.* Auxilary basis sets to approximate coulomb potentials. *Chem. Phys. Lett.* **242**, 652–652 (1995).

21. Eichkorn, K., Weigend, F., Treutler, O. & Ahlrichs, R. Auxiliary basis sets for main row atoms and transition metals and their use to approximate coulomb potentials. *Theo. Chem. Acc.* **97**, 119–124 (1997).

22. Preuss, H. *et al.* Energy-adjusted *ab initio* pseudopotentials for the second and third row transition elements. *Theor. Chim. Acta* **77,** 123–141 (1990).

23. Weigend, F. & Ahlrichs, R. Balanced basis sets of split valence, triple zeta valence and quadruple zeta valence quality for H to Rn: Design and assessment of accuracy. *Phys. Chem. Chem. Phys.* **7**, 3297–3305 (2005).

24. Weigend, F., Häser, M., Patzelt, H. & Ahlrichs, R. RI-MP2: optimized auxiliary basis sets and demonstration of efficiency. *Chem. Phys. Lett.*, **294**, 143–152 (1998).

25. Tao, J., Perdew, J. P., Staroverov, V. N. & Scuseria, G. E. Climbing the density functional ladder: nonempirical meta-generalized gradient approximation designed for molecules and solids. *Phys. Rev. Lett.* **91**, 146401 (2003).

26. Klamt, A. & Schüürmann, G. COSMO: A new approach to dielectric screening in solvents with explicit expressions for the screening energy and its gradient. *J. Chem. Soc. Perkin Trans.* **2**, 799–805 (1993).

27. Grimme, S. Supramolecular Binding Thermodynamics by Dispersion-Corrected Density Functional Theory, *Chem. - A Eur. J.* **18**, 9955–9964 (2012).

28. Pinski, P., Riplinger, C., Valeev, E. F. & Neese, F. Sparse maps—A systematic infrastructure for reduced-scaling electronic structure methods. I. An efficient and simple linear scaling local MP2 method that uses an intermediate basis of pair natural orbitals. *J. Chem. Phys.* **143**, 34108 (2015).

29. Riplinger, C., Sandhoefer, B., Hansen, A. & Neese, F. Natural triple excitations in local coupled cluster calculations with pair natural orbitals. *J. Chem. Phys.* **139**, 134101 (2013).

30. Neese, F., Hansen, A. & Liakos, D. G. Efficient and accurate approximations to the local coupled cluster singles doubles method using a truncated pair natural orbital basis. *J. Chem. Phys.* **131**, 64103 (2009).

31. Neese, F. *et al.* A new near-linear scaling, efficient and accurate, open-shell domain-based local pair natural orbital coupled cluster singles and doubles theory. *J. Chem. Phys.* **146**, 164105 (2017).

32. Riplinger, C. & Neese, F. An efficient and near linear scaling pair natural orbital based local coupled cluster method. *J. Chem. Phys.* **138**, 034106 (2013).

33. Dunning, Jr., T. H. Gaussian basis sets for use in correlated molecular calculations. I. The atoms boron through neon and hydrogen, *J. Chem. Phys.* **90**, 1007–1023 (1989).

34. Woon, D. E. & Dunning, Jr., T. H. Gaussian basis sets for use in correlated molecular calculations. III. The atoms aluminum through argon. *J. Chem. Phys.* **98**, 1358–1371 (1993).

35. Balabanov, N. B. & Peterson, K. A. Systematically convergent basis sets for transition metals. I. All-electron correlation consistent basis sets for the 3d elements Sc-Zn. *J. Chem. Phys.* **123**, 064107 (2005).

36. Balabanov, N. B. & Peterson, K. A. Basis set limit electronic excitation energies, ionization potentials, and electron affinities for the *3d* transition metal atoms: Coupled cluster and multireference methods. *J. Chem. Phys.* **125**, 074110 (2006).

37. Weigend, F., Kohn, A. & Hattig, C. Efficient use of the correlation consistent basis sets in resolution of the identity MP2 calculations. *J. Chem. Phys.* **116**, 3175–3183 (2002).

38. Hill, J. G. & Platts, J. A. Auxiliary basis sets for density fitting-MP2 calculations: Nonrelativistic triple-zeta all-electron correlation consistent basis sets for the 3d elements Sc-Zn. *J. Chem. Phys.* **128**, 044104 (2008).

39. Bross, D. H., Hill, J. G., Werner, H. J. & Peterson, K. A. Explicitly correlated composite thermochemistry of transition metal species. *J. Chem. Phys.* **139**, 094302 (2013).

40. Zhong, S., Barnes, E. C. & Petersson, G. A. Uniformly convergent n-tuple-ζ augmented polarized (nZaP) basis sets for complete basis set extrapolations. I. Self-consistent field energies. *J. Chem. Phys.* **129**, 184116 (2008).

41. Neese, F. & Valeev, E. F. Revisiting the Atomic Natural Orbital Approach for Basis Sets: Robust Systematic Basis Sets for Explicitly Correlated and Conventional Correlated ab initio Methods? *J. Chem. Theory Comput.* **7**, 33–43 (2011).

42. Helgaker, T., Klopper, W., Koch, H. & Noga, J. Basis-set convergence of correlated calculations on water. *J. Chem. Phys.* **106**, 9639–9649 (1997).

43. Perdew, J. P., Burke, K. & Ernzerhof, M. Rationale for mixing exact exchange with density functional approximations. *J. Chem. Phys.* **105**, 9982–9985 (1996).

44. van Lenthe, E., van der Avoird, A. & Wormer, P. E. S. Density functional calculations of molecular hyperfine interactions in the zero order regular approximation for relativistic effects. *J. Chem. Phys.* **108**, 4783–4796 (1998).

45. Pantazis, D. A., Chen, X.-Y., Landis, C. R. & Neese, F. All-Electron scalar relativistic basis sets for third-row transition metal atoms. *J. Chem. Theory Comput.* **4**, 908–919 (2008).

46. van Wüllen, C. Molecular density functional calculations in the regular relativistic approximation: Method, application to coinage metal diatomics, hydrides, fluorides and chlorides, and comparison with first-order relativistic calculations. *J. Chem. Phys.* **109**, 392–399 (1998).

47. Autschbach, J. Perspective: Relativistic effects. *J. Chem. Phys.* **136**, 150902 (2012).

48. Neese, F., Wennmohs, F., Hansen, A. & Becker, U. Efficient, approximate and parallel Hartree-Fock and hybrid DFT calculations. A 'chain-of-spheres' algorithm for the Hartree-Fock exchange. *Chem. Phys.* **356**, 98–109 (2009).

49. Weigend, F. Accurate Coulomb-fitting basis sets for H to Rn. *Phys. Chem. Chem. Phys.* **8**, 1057–1065 (2006).

50. Becke, A. D. Density-functional thermochemistry. III. The role of exact exchange. *J. Chem. Phys.* **98**, 5648–5652 (1993).

51. Neese, F. Prediction of molecular properties and molecular spectroscopy with density functional theory: From fundamental theory to exchange-coupling. *Coord. Chem. Rev.* **253**, 526–563 (2009).

52. APEX2 v2014.9-0 (SAINT/SADABS/SHELXT/SHELXL), Bruker AXS Inc., Madison, WI, USA, 2014. Or APEX3 v2016.9-0 (SAINT/SADABS/SHELXT/SHELXL), Bruker AXS Inc., Madison, WI, USA, 2016.

53. Sheldrick, G. M., SHELXT-Integrated space-group ad crystal-structure determination. *Acta Cryst.* **A71**, 3–8 (2015).

54. Sheldrick, G. M., Crystal structure refinement with SHELXL. *Acta Cryst.* **C71**, 3–8 (2015).

55. Sheldrick, G. M., A short history of SHELX. *Acta Cryst.* **A64**, 112–122 (2008).
